# Catalytic Serine Labeling in Nonaqueous, Acidic Media

**DOI:** 10.1002/chem.202404002

**Published:** 2025-02-09

**Authors:** Seiya Ishizawa, Chiamaka P. Uzoewulu, Yume Iwakura, Anuja Koirala, Shinichi Sato, Jun Ohata

**Affiliations:** ^1^ Department of Chemistry North Carolina State University Raleigh North Carolina 27695 United States; ^2^ Frontier Research Institute for Interdisciplinary Sciences Tohoku University Sendai 980-8578 Japan

## Abstract

Chemoselective modification of alkyl alcohols (e. g., serine residues) on proteins has been a daunting challenge especially in aqueous media. Herein, we report chemical modification of alkyl alcohols in protein and cell lysate samples using carboxylic acid‐based bioconjugation media. The acidic medium is not only useful to suppress reactivity of other nucleophiles in proteins, but the medium also serves as a potentially biomolecule‐compatible solvent. The acid‐catalyzed acylation strategy has a unique selectivity paradigm compared to the common active‐serine‐targeted method and would act as a new strategy for studying biological roles of serine residues.

## Graphical abstract

Serine‐targeting protein bioconjugation has been a formidable challenge because of the modest reactivity of the alkyl alcohol group in aqueous media. This work demonstrates a biomolecule‐compatible, nonaqueous approach to achieve alkyl alcohol‐selective labeling through acid‐catalyzed esterification reactions, which offers a unique advantage over the existing serine protease‐targeting method using a fluorophosphonate reagent.

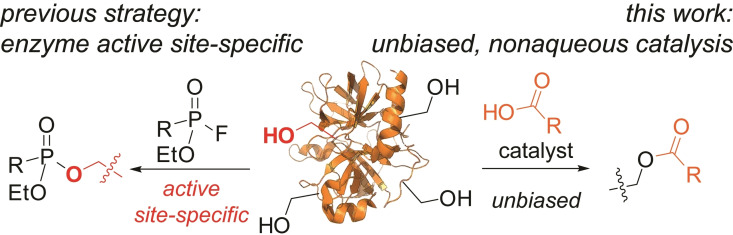

## Introduction

Although selective chemical reactions of alkyl alcohols are ubiquitous processes in synthetic organic chemistry as well as cellular processes of biological systems, chemical tagging of alkyl alcohol‐based amino acid residues such as serine remains a formidable challenge in the field of chemical biology. Existing protein bioconjugation methods often are harnessed by inherent high nucleophilicity of amino acid side chains (e. g., lysine, cysteine, and tyrosine),^[1]^ facilitating a wide range of applications including therapeutic/diagnostic use of bioconjugates[^2^, ^3^] and interrogation of cellular processes through labeling of such target amino acids in living systems.^[4]^ Even if serine is known to undergo post‐translational modification most frequently among the 20 canonical amino acids,^[5]^ chemoselective labeling of serine or tools for the chemical modification of serine to study its biological diversity is simply lacking.^[6]^ The dearth of the serine labeling methods is likely due to the ubiquity of the functional group (i. e., OH group) and its modest nucleophilicity in aqueous media, and common oxophilic reagents used in organic chemistry for alkyl alcohol substrates would be readily quenched by the solvent molecule before reaching the target amino acid. As natural systems take advantage of enzymatic processes for selective serine modification such as phosphorylation even in complex cellular environments,^[7]^ various bioinspired phosphorous‐based approaches have been devised to chemically tag serine residues in aqueous media including fluorophosphonates (active‐site serine modification)^[8]^ and oxathiophospholanes (chemoselective serine modification in peptides and a small protein)^[9]^ although there has not been a general chemoselective modification method applicable to protein and cellular samples. Historically, organic chemists, on the other hand, began utilizing aprotic organic solvents to address the challenges of the alkyl alcohol reactions.[^10^, ^11^] Of course, the majority of aprotic organic solvents are incompatible with proteins because of their denaturation/aggregation,^[12]^ but it is intriguing that serine‐targeting protein bioconjugation in nonaqueous media that is compatible with biomolecules has not been pursued to date even though such an approach would address the challenges of serine labeling.

**Figure 1:**
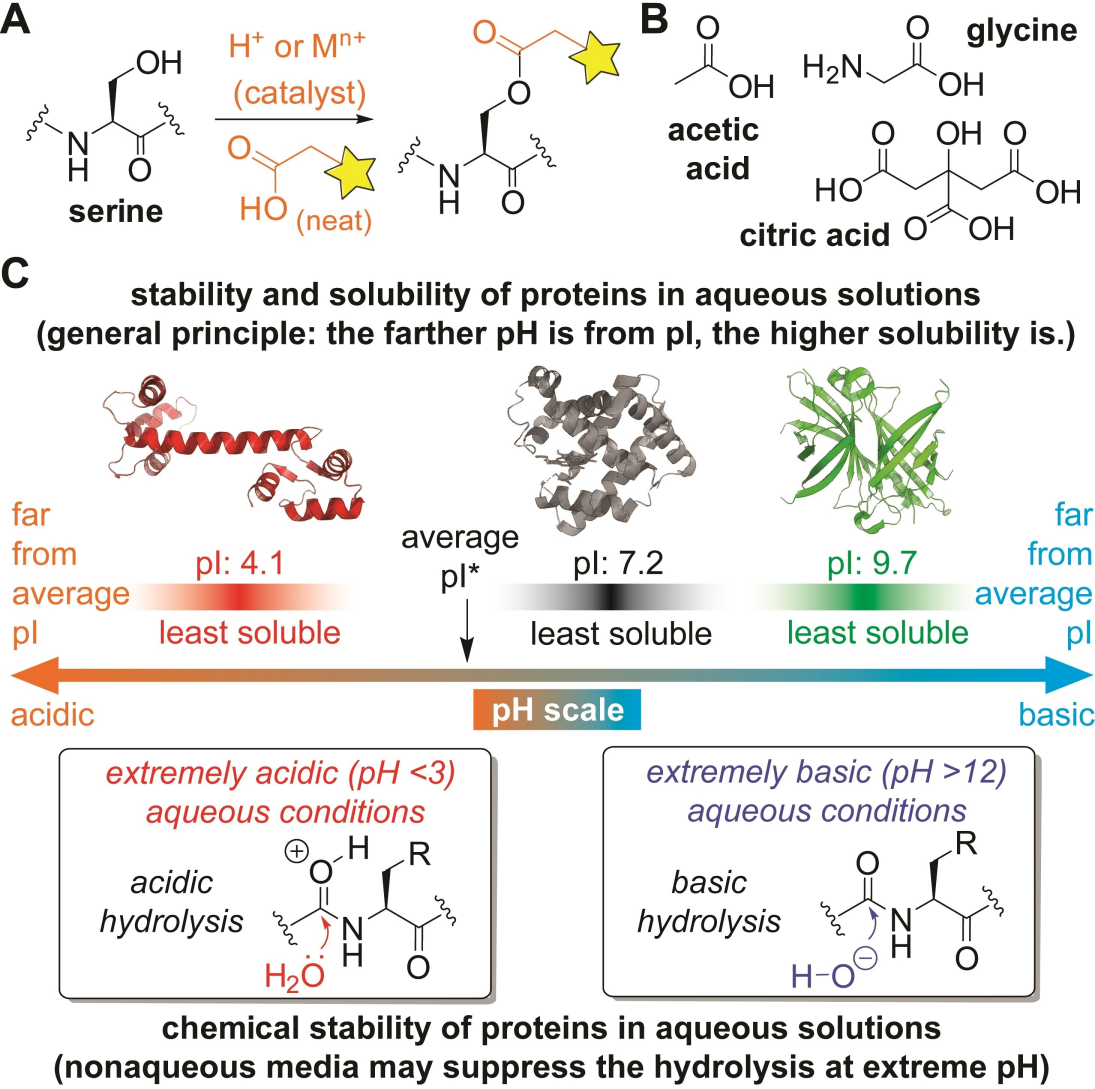
Nonaqueous, acidic media for labeling alkyl alcohol of proteins. (A) General reaction scheme of this work using carboxylic acid‐based molecule as a bioconjugation medium. (B) Chemical structures of common biological buffers containing carboxylic acid groups. (C) Schematic depiction of protein stability/solubility and their hydrolysis susceptibility in aqueous solution at different pH ranges. *Average isoelectric point (pI) for proteins across kingdoms of life based on literature.^[24]^

Recognizing the biocompatibility of various carboxylic acid‐based molecules and protein stabilization by pH controls, this work demonstrates acid‐catalyzed acylation reaction in nonaqueous acid media (Figure 1A,B). Our previous studies demonstrated protein bioconjugation in protein‐compatible nonaqueous media such as ionic liquid[^13^, ^14^, ^15^] and fluoroalcohol solvents.[^16^, ^17^] Notably, the urea‐forming lysine modification process in ionic liquid did not cause any noticeable effects on native protein abilities such as streptavidin and antibody binding capabilities even by introducing the strong base/nucleophile iminophosphorane (p*K*_a_ of its conjugate acid is >20^[18]^).^[13]^ In aqueous media, extremely acidic and basic conditions are often avoided for studying proteins in part due to the potential hydrolysis of amide backbones, though those extreme pH ranges could be helpful for the prevention of protein precipitation in terms of their isoelectric points (Figure 1C).^[19]^ Because a hydrolysis process would not be a favorable chemical event in nonaqueous media with a substantially low concentration of water compared to aqueous solutions, we hypothesized that extreme pH conditions could be potentially protein‐compatible, which has been demonstrated by the iminophosphorane‐based bioconjugation in ionic liquid as an extremely basic process. Carboxylic acid‐based molecules are often biocompatible and used as common buffer components for biochemical studies (e. g., acetate, glycine, and citrate buffers).^[20]^ Acidic conditions are often preferred for structural studies of proteins using NMR to observe exchangeable NH protons too,[^21^, ^22^, ^23^] indicating potential applicability of acidic approaches for various protein substrates. Therefore, we examined alcohol‐selective chemical reactions in nonaqueous carboxylic acid‐based media as an extremely acidic bioconjugation process. In particular, our focus was an acid‐catalyzed acylation approach (i. e., Fischer esterification) that would be not only potentially biomolecule‐compatible due to the abovementioned reasons, but would the acidic media also suppress the reactivity of many intrinsically basic/nucleophilic amino acid side chains through their protonation.

## Results and Discussion

**Figure 2:**
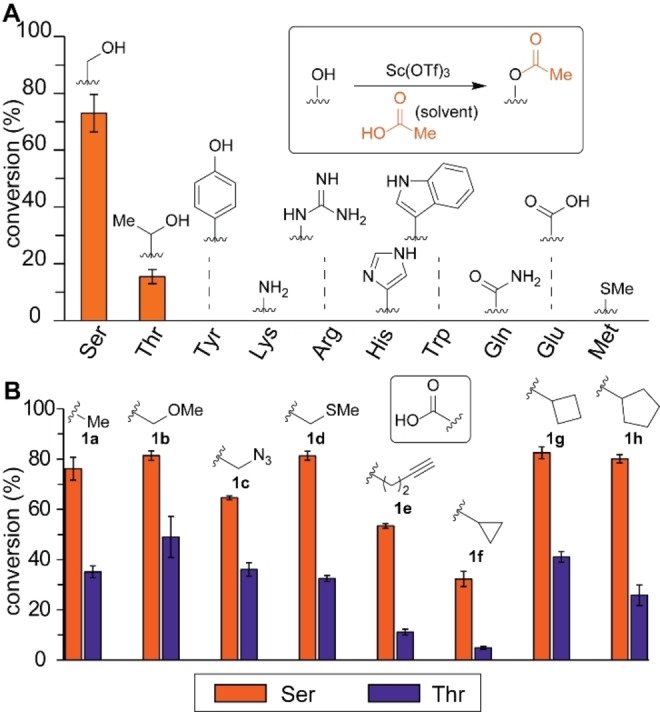
Amino acid screening and carboxylic screening of the acid‐catalyzed acylation reaction. Typical reaction conditions: Amino acid (5 mM) and catalytic amount of scandium (III) triflate (1.25 mM) in acetic acid at 50 °C for 24 h. Error bars represent standard deviation (*n*=3). (A) Schematic illustration of the acylation reaction and a bar graph showing the reaction conversion of liquid‐chromatography (LC)‐based analysis of modification of different *N*‐protected (F‐moc) amino acids. (B) Bar graph showing the reaction conversion of LC‐based analysis of modification of *N*‐protected serine and *N*‐protected threonine with different carboxylic acids.

Catalytic acylation processes in an acidic medium enabled chemoselective labeling toward the alkyl hydroxyl groups over other nucleophilic side chains at an amino‐acid level (Figures 2A, S1–S8). To test our hypothesis, *N*‐protected canonical amino acids were subjected to the acylation processes with acetic acid as a solvent and a catalytic amount of scandium triflate. Scandium triflate was chosen as a catalyst for this amino acid study because the catalyst is known as one of the strongest Lewis acids.^[25]^ The acylation products were observed only for serine and threonine in liquid‐chromatography mass spectrometry (LC‐MS) analysis with a higher selectivity toward serine over threonine (Figure 2B, Figure S5). In contrast, other nucleophilic amino acids including tyrosine, lysine, arginine, histidine, tryptophan, glutamine, glutamic acid, and methionine did not show meaningful product formation (Figure 2A, Figures S6–S8), indicative of the high chemoselectivity to alkyl alcohols through suppression of the reactivities of those typical nucleophiles. Metal Lewis acid catalysts are known to increase electrophilicity of carboxylic acids through coordination events, and the observed serine/threonine reactivity was probably driven through a similar mechanism.[^26^, ^27^]

Screening of a series of carboxylic acid labeling reagents implied that reactivity and selectivity control of the bioconjugation is possible through electronic and steric alterations (Figures 2B, S9–S16). We have tested carboxylic acid compounds to assess both the electronic and steric effects on reactivity and selectivity. While some carboxylic acids are solids under ambient conditions such as pentynoic acid (**1e**), we discovered that the labeling process proceeds efficiently in a 4–5 M solution of the acid in a nonaqueous solvent such as acetonitrile and ionic liquid (Figures S17, S103–105). Whereas overall modification efficiency varied according to the carboxylic acid utilized, we did not observe clear trends in the selective modification of serine over threonine despite the introduction of heteroatoms at the α‐position as well as cyclic structures for electronic and steric perturbations. The acylation product formation was also observed using acids with bioorthogonal handles such as azide and terminal alkyne groups, although the reaction with azidoacetic acid showed an unidentified byproduct in the LC‐MS analysis (Figure S11). The feasibility of the acylation process with 4‐pentynoic acid (**1e**) and subsequent copper‐catalyzed azide‐alkyne cycloaddition reaction of the acylated group was confirmed as well (Figures S18–S26). It is noteworthy that typical reactive acylating reagents (i. e., acyl chlorides and acid anhydrides) were reported to modify even tyrosine residues in addition to serine,[^28^, ^29^] underscoring the importance of the acid‐catalyzed acylation with carboxylic acids employed in the present study through suppression of this unwanted effect.

**Figure 3:**
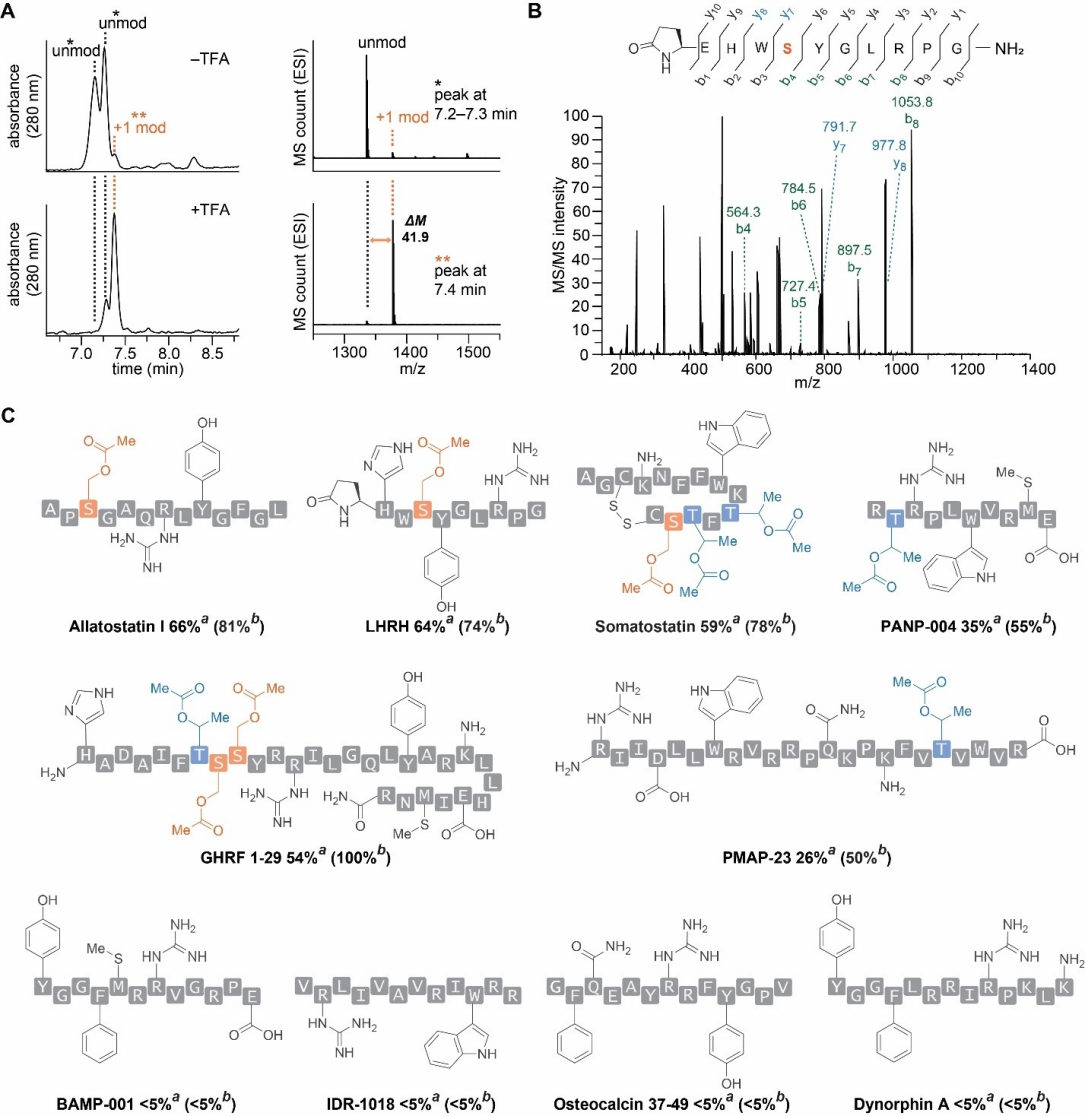
Acid‐catalyzed acylation reaction on peptide substrates. Reaction conditions: peptide (0.05–1.0 mM), trifluoroacetic acid (6 % v/v, 0.78 M or 12 % v/v, 1.6 M) in acetic acid for 24 h at 50 °C. (A) Liquid chromatography‐mass spectrometry (LC‐MS) analysis of the modification of allatostatin I in acetic acid with or without trifluoroacetic acid as a catalyst. (B) Tandem mass spectrometry (MS/MS) analysis of luteinizing hormone‐releasing hormone (LHRH). Observed y and b ions are highlighted in cyan and green, respectively. (C) Amino acid sequence of peptide substrates with chemical structures of representative side chain groups. Serine residues and threonine residues are highlighted in orange and cyan, respectively. ^*a*^Reaction conversions of peptides modified with 6 % v/v, 0.78 M of trifluoro acetic acid. ^*b*^Reaction conversions of peptides modified with 12 % v/v, 1.6 M of trifluoro acetic acid. Reaction conversions were obtained using peaks of starting materials and products in UV chromatograms (280 nm). LHRH: Luteinizing hormone‐releasing hormone human acetate salt. Somatostatin‐14: cyclic somatostatin‐14 with a disulfide bond between the two cysteine residues. GHRF 1–29: Growth hormone releasing factor 1–29. PMAP‐23: Porcine myeloid antibacterial peptide‐23. PANP‐004: BDC2.5 mimotope peptide 1040–63. BAMP‐001: Precursor of bovine adrenomedullary Met‐enkephalin. IDR‐1018: Innate defense regulator 1018.

The high chemoselectivity of the acidic strategy toward the alkyl hydroxyl groups was also observed for various peptide substrates (Figure 3). Trifluoroacetic acid (TFA) was chosen as a catalyst/co‐solvent for peptide acylation based on the results of catalyst screenings with peptide and protein as substrates (Table S1–S2, Figures S27–S39) and its known compatibility with a broad range of peptide substrates (e. g., a general deprotection method of peptides with TFA after solid‐phase synthesis)^[30]^ and matrix for peptide LC‐MS analysis (i. e., volatile acid). Although the use of an excess amount of the acid proved necessary, the acid is likely to act as a catalyst in a similar fashion to traditional Fischer esterification processes. It should be noted that the use of excess amounts of catalysts is quite common in catalytic protein bioconjugation strategies.^[31]^ LC‐MS analysis showed the acylation product formation for serine‐ and threonine‐containing peptides regardless of the presence of other nucleophilic amino acid side chains such as phenol, amine, guanidine, indole, and imidazole (Figure 3A, 3C, S40–S50, Table S3). Serine‐containing peptides showed higher conversions than peptides that contain threonine without serine (e. g., allatostatin I compared to porcine myeloid antibacterial peptide‐23/PMAP‐23), implying for serine selectivity of the acid‐catalyzed acylation method over threonine also at the peptide level. As expected, no detectable product signals were observed for peptides that do not contain any alkyl alcohols such as osteocalcin and dynorphin. The amino acid selectivity trend was not compromised even with higher TFA loading (1.6 M as condition b, compared to condition with 0.8 M in Figure 3), and up to 85 % conversion was observed for serine‐containing peptides. Tandem mass spectrometry (MS/MS) suggested reactions on serine or threonine (Figure 3B, S51–S57).

**Figure 4:**
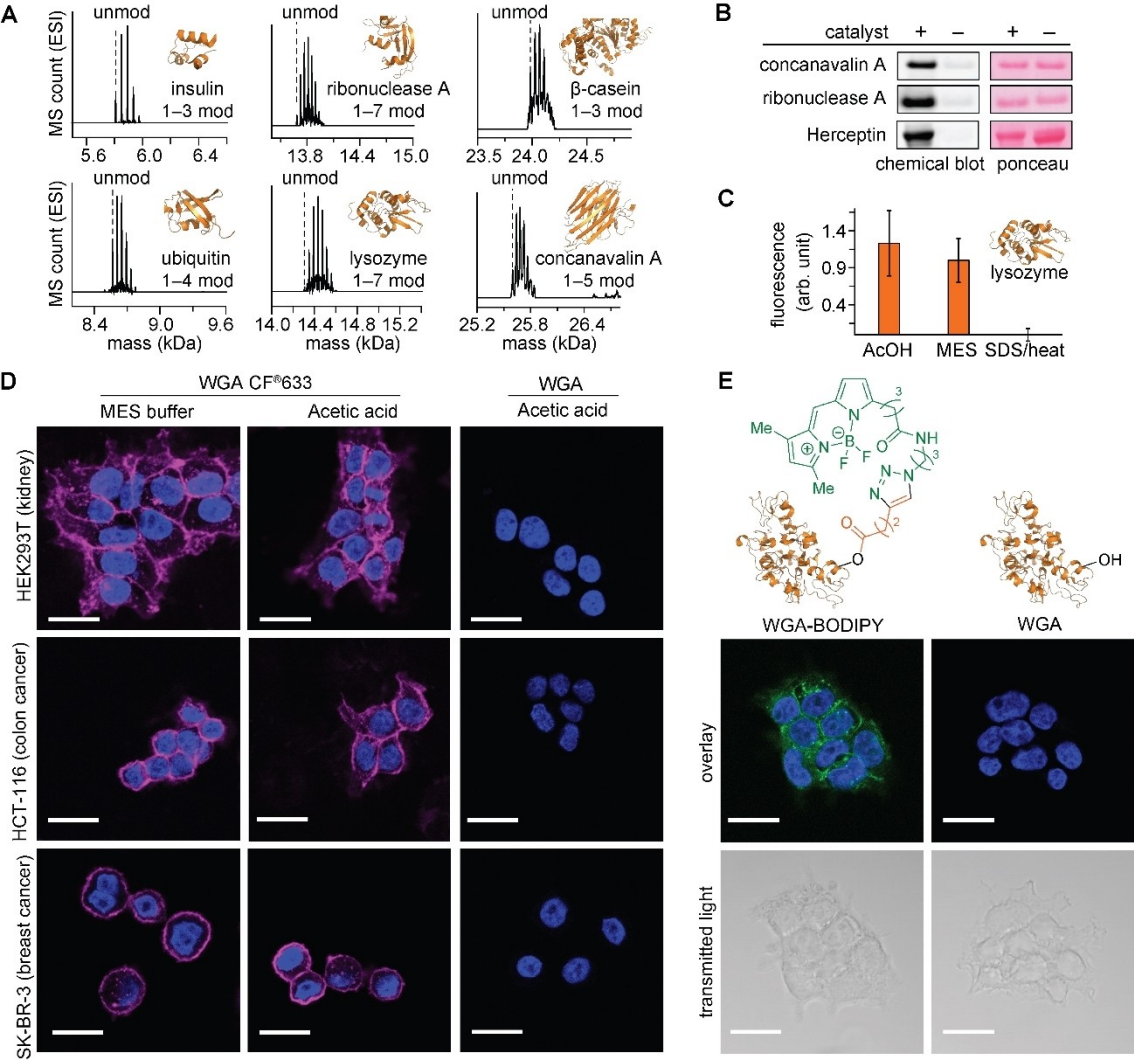
Acid‐catalyzed acylation of proteins and effects of the carboxylic acid media/acid‐catalyzed acylation process on the activity of proteins. (A) Electrospray ionization mass spectrometry (ESI‐MS) analysis of the modification of various proteins with acetic acid in the presence of trifluoroacetic acid (TFA) catalyst. Typical modification conditions: protein (20–100 μM) and TFA (1–12 % v/v, 0.13–1.6 M) in acetic acid at 37 °C or 50 °C for 24 h. (B) Chemical blot analysis (detection of an alkyne tag of a protein on a blot membrane with fluorogenic coumarin azide)^[32]^ of the modification of different proteins treated with an alkyne‐tagged acid **1e**. Catalyst: TFA. Full‐size membrane images are shown in Figure S61. (C) Fluorescence‐based activity assay of lysozyme in aqueous solution after the protein was treated in (1) acetic acid (AcOH) at 37 °C for 24 hours, (2) 2‐(N‐morpholino)ethanesulfonic acid or MES buffer (50 mM, pH 7.4) at 37 °C for 24 hours, or (3) sodium dodecylsulfate (SDS) aqueous solution at 95 °C for 1 min. (D) Confocal microscopy analysis of three different cell lines (HEK 293T, HCT‐116, and SK‐BR‐3) stained with wheat germ agglutinin (WGA)–fluorophore conjugate (WGA CF®633, magenta) treated in MES buffer or acetic acid, and wheat germ agglutinin (WGA) treated in acetic acid. Nuclear stain was performed with DAPI (blue). Scale bars: 20 μm. Each fluorescence channel is shown in Figure S97–S99. (E) Confocal microscopy analysis of HEK293T cells stained with WGA modified with an alkyne‐tagged acid 1 e followed by BODIPY‐azide (left) or unmodified WGA (right). Nuclear stain was performed with DAPI (blue). Scale bars: 20 μm. Each fluorescence channel is shown in Figure S101.

The acid‐catalyzed acylation processes were able to modify a range of polypeptide substrates, enabling the installation of a bioorthogonal handle for imaging purposes (Figure 4). The acylation products were observed for the range of polypeptides (the number of serine residues contained in each are shown in parentheses) such as insulin (3), ubiquitin (3), ribonuclease A (15), lysozyme (10), β‐casein (16), and concanavalin A (31) as shown in Figure 4A and Figures S58–S60. We previously demonstrated that ionic liquids can be conducive for retaining protein structure and activity,[^13^, ^16^] and we employed imidazolium‐based ionic liquid (ethyl‐methylimidazolium tetrafluoroborate) as a solvent for labeling with a solid alkyne‐tagged carboxylic acid. In blot membrane‐based analysis for alkyne handles on proteins,^[32]^ positive fluorescence responses were observed for modification of model proteins concanavalin A, ribonuclease A, and Herceptin, suggesting the successful installation of the alkyne tag through the acylation process (Figures 4B, S61). The modification of concanavalin A and ribonuclease A was also confirmed by mass spectrometry (Figure S62). While the trypsin digestion and subsequent tandem mass spectrometry analysis of modified protein showed some adduct formation on lysine and tyrosine residues to a certain extent (Figures S63–S93), peaks of modified serine peptide fragments were substantially higher in the mass count (Table S4), which also supports our hypothesis about the alkyl alcohol selectivity of the method. Although the intact MS analysis did not indicate the adduct formation of TFA catalyst to model protein and peptide substrates, it should be noted that some TFA adducts were observed in the proteomics study to a lesser extent.

The compatibility of the acid‐catalyzed acylation conditions with protein substrates was demonstrated through enzyme activity assays and immunofluorescence experiments (Figure 4C–E). To estimate the effects of an acidic nonaqueous medium on the activity of an enzyme, lysozyme (a glycosyl hydrolase) was incubated in acetic acid at 37 °C for 24 hours and then subjected to a fluorescence‐based enzyme‐activity assay in aqueous solution.[^33^, ^34^] The acid‐treated lysozyme showed a comparable level of fluorescence intensity as the positive control sample treated in an aqueous buffer (Figure 4C), and thus the retained enzymatic activity in aqueous buffer after the acid treatment was indicated. Lysozyme is often handled in the acidic acetate buffer[^35^, ^36^] as its isoelectric point is 10.7.^[37]^ Perhaps, other classes of proteins that favor acidic buffers (e. g., endonucleases^[38]^ and lysosomal enzymes^[39]^) may also be compatible with acidic nonaqueous media. We also tested the binding activity of a commercial fluorophore conjugate of a glycol‐protein binding protein, wheat germ agglutinin (WGA CF®633) to cancer cell lines after incubation in acetic acid without a catalyst (i. e., non‐acetylating conditions). Confocal microscope imaging demonstrated similar fluorescence intensity and staining pattern of the acid‐treated sample to those of the positive control protein treated in an aqueous buffer while negative control experiments with unconjugated WGA did not exhibit any substantial signals, suggesting that WGA conjugate retained its activity even after extended incubation in the nonaqueous acidic medium (Figures 4D, S97–S99). Encouraged by those results of the retention of protein activities in acidic media without the acylation process, we then evaluated the binding activity of modified WGA after the catalytic bioconjugation in the acidic medium as well as subsequent fluorophore attachment through the copper‐catalyzed azide‐alkyne cycloaddition reaction. The fluorescently labeled protein was characterized by gel fluorescence (Figure S100). The localization patterns of the HEK293T cells stained with the fluorophore conjugate prepared by the acidic labeling and click chemistry proved virtually identical to those by the commercial staining dye (Figures 4E, S101), and thus the acidic modification and alkyl alcohol tagging processes probably did not have substantial negative impacts on the protein activity. The negative control confocal microscope imaging with unconjugated WGA did not show a meaningful fluorescence signal, confirming that the BODIPY dye installed by the acylation process and click chemistry is responsible for the fluorescence response.

**Figure 5:**
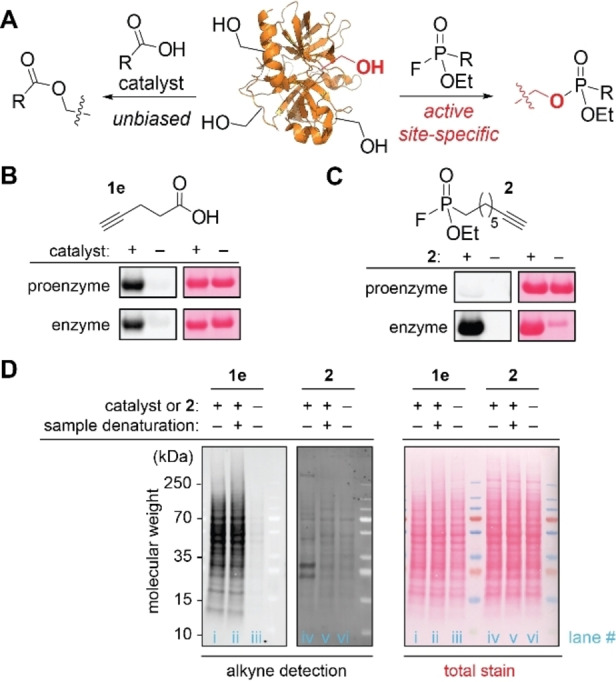
Comparison of the acid‐catalyzed acylation bioconjugation with the active‐site serine modification by fluorophoshonate. (A) Schematic illustration of the difference between the acid‐catalyzed acylation (unbiased) and modification with fluorophosphonate (active site‐specific). (B) Chemical blot analysis (detection of an alkyne tag in proteins on a blot membrane)^[32]^ of the modification of α‐chymotrypsinogen A (proenzyme) and chymotrypsin (enzyme) treated with an alkyne‐tagged acid **1e**. (C) Chemical blot analysis of the modification of α‐chymotrypsinogen A and chymotrypsin treated with fluorophosphonate alkyne **2**. (D) Chemical blot analysis of the modification of HEK293T cell lysate treated with either an alkyne‐tagged acid **1e** (lane i–iii) or fluorophosphonate **2** (lane iv–vi). Sample denaturation: 80 °C for 5 min before the labeling process (lane ii and v). Catalyst: trifluoroacetic acid (TFA).

The acid‐catalyzed acylation can offer general alkyl alcohol labeling without obvious preferences to active serines in enzymatic pockets (Figure 5). The commonly used alcohol‐targeting protein labeling reagents, fluorophosphonates, are known to selectively modify the serine residues of the active site of serine proteases.^[8]^ As the fluorophosphonate‐based labeling is limited to enzyme active sites, we envisioned that the acid‐catalyzed acylation may be able to act as a complementary alkyl alcohol‐targeting method. Incubation of a model serine protease (α‐chymotrypsin) and corresponding proenzyme (α‐chymotrypsinogen A) with fluorophosphonates **2** showed a sole modification toward chymotrypsin whereas the proenzyme remained intact, which is consistent with a previous report.^[40]^ On the other hand, the acid‐catalyzed acylation showed adduct formations in both substrates, indicating the independent nature of the labeling efficiency from the active site status (Figure 5A–C, S103–S105).^[41]^ Finally, the unique selectivity profile of the acid‐catalyzed acylation was also demonstrated at a cell lysate level (Figure 5D). First, the acylation processes in acidic media did not show any noticeable changes to the band shapes and patterns of HEK293T cell lysates in the total stain image from those of the samples in aqueous solutions, and this observation may be indicating a potential utility of nonaqueous acidic media for broad purposes. We then performed the control experiments with fluorophophonate **2**, and the band pattern of cell lysate of HEK293T modified with **2** was similar to the reported gel‐based activity protein profiling results with fluorophosphonate‐fluorophore conjugates (Figure S106).[^42^, ^43^] Compared to the cell lysate modified by **2**, numerous bands showed fluorescence signals for the acylation samples in the presence of the catalyst in the blot analysis, suggesting modification on many types of proteins beyond specific serine proteases. The fluorescence signals with proteins treated with fluorophoshonate **2** were decreased for heat‐denatured cell lysates through the loss of the protease activity as reported in the literature.[^8^, ^44^, ^45^] By contrast, the acid‐catalyzed acylation did not show an apparent difference by the heat denaturation, further confirming that the acid‐catalyzed protein modification is independent of the serine protease activities. These results indicate that the acid‐catalyzed acylation method would serve as a complementary serine‐targeting method to the enzyme‐specific labeling with fluorophosphonates. It is noteworthy that the present method has advantages in several ways including the shelf stability and broad availability of carboxylic acid compounds.

## Conclusions

Though a variety of chemical reactions of serine in natural systems has been known for decades, the challenges to achieve chemical modification of serines have been the longstanding issue of chemists, and the present work demonstrated the catalytic bioconjugation in biomolecule‐compatible, nonaqueous acidic media. With the success of cell lysate labeling described above, the acylation method would be applicable to the profiling of the activity of serine residues that are not located in enzyme active sites.[^46^, ^47^] In contrast to the natural system facilitating reactions of a mildly reactive solvent molecule (i. e., water) through actions of enzymes as catalysts, the nonaqueous strategy promoted the acylation reaction with a solvent molecule through acid catalysis by reaching the extreme pH scale that would not be accessible in aqueous media. As acid catalysis is a common synthetic organic chemistry method,^[48]^ the acidic nonaqueous approach can be extended to a variety of other chemical transformations, which would expand the repertoire of the catalytic bioconjugation strategies.

## Experimental Section

### Protein Modification in Acetic Acid for Figure 4

To acetic acid (typically 30 μL scale) in a 1.7‐mL Eppendorf tube, TFA (1–12% v/v, 0.13–1.6 M final concn from neat solution) and an aqueous solution of a protein (20–100 μM final concn from 2 mM–10 mM stock solution in 50 mM pH 7.4 MES buffer, H_2_O, or acetic acid) were added. Specific conditions of each substrate are described in figure captions of the MS data in the Supporting Information. The mixture was incubated at 37 °C (all proteins and insulin) or 50 °C (ubiquitin) for 24 h. 5 μL of the reaction mixture was diluted with 20 μL of H_2_O (all proteins) or H_2_O/MeCN 1:1 (insulin and ubiquitin), and the diluted solution was centrifuged (15 krcf, rt, 15 min). The resulting supernatant of protein samples was analyzed by LC‐MS with MAbPac column (Thermo Fisher, 088648, particle size 4 μm, diameter 2.1 mm, length 50 mm). The resulting supernatant of insulin and ubiquitin was analyzed by LC‐MS with hypersil gold column (Thermo Fisher, 25005‐259070A, particle size 5 μm, diameter 10 mm, length 250 mm).

### WGA Modification, Purification, and Analysis

To 4‐pentynoic acid in ethylmethylimidazolium tetrafluoroborate or EMIM BF_4_ (4 M, 10 μL scale), TFA (6% v/v, 0.78 M final concn from neat solution) and WGA solution (3 mg/ml final concn from 100 mg/ml stock solution in 100 mM aqueous 4‐pentynoic acid) were added. The reaction mixture was incubated at 37 °C overnight. To the reaction mixture in a 1.7 mL Eppendorf tube, cold acetone (1200 μL, −20 °C) was added in one portion. The mixture was mixed by upside‐down shaking and sat at −80 °C for 1 h or overnight. The precipitates were collected by centrifugation (15,000 rcf, 15 min, 4 °C) and the supernatant was removed. The pellet was further washed by three additional cycles of acetone (600 μL) addition and centrifugation. After removing the supernatant, the pellet was air‐dried on the bench at rt for 15 min. To the dried pellet, H_2_O (30 μL scale), *N*‐methyl morpholine (NMM) buffer (50 mM final concn from 200 mM aqueous stock solution, pH 7.4), sodium ascorbate (2 mM final concn from 100 mM aqueous stock solution), THPTA (0.2 mM final concn from 20 mM aqueous stock solution), BODIPY‐N_3_ (0.2 mM final concn from 20 mM stock solution in DMSO), and CuSO_4_ (1 mM final concn from 100 mM aqueous stock solution) were added. The reaction mixture was incubated at rt for 1 h. The reaction was quenched with EDTA (1 μL, 250 mM). To the reaction mixture in a 1.7 mL Eppendorf tube, cold acetone (1200 μL, −20 °C) was added in one portion. The mixture was mixed by upside‐down shaking and sat at −80 °C for 1 h or overnight. The precipitates were collected by centrifugation (15,000 rcf, 15 min, 4 °C), and the supernatant was removed. The pellet was further washed by one additional cycle of acetone (600 μL) addition and centrifugation. After removing the supernatant, the pellet was air‐dried on the bench at rt for 15 min and reconstituted in H_2_O (30 μL). The resulting solution was used in the *fluorescence imaging experiment* described in Supporting Information. In addition, 3 μL of the resulting solution was diluted with H2O (6 μL) and 4X loading buffer (3 μL). 10 μL of the resulting mixture was subjected to SDS‐PAGE at 140 V for 45 min. The gel was imaged with Amersham ImageQuant 800 by 460 nm excitation with a 525 nm bandpass filter (±20 nm), followed by total staining with Coomassie Blue Brilliant R‐250 staining and destaining processes.

### Modification of Cell Lysate

To a 5 M solution of 4‐pentynoic acid in acetonitrile (50 μL scale) in a 1.7 mL Eppendorf tube, TFA (3% v/v, 0.39 M final concn from neat solution) and HEK293T cell lysate (0.24 mg/mL final concn from 1.69 mg/mL stock solution in a lysis buffer). For some conditions that require the denaturation process (lanes ii and v in Figure 5D), the cell lysate stock solution was preheated at 80 °C for 5 min before use. The mixture was incubated at 37 °C for 6 h. To the reaction mixture in a 1.7 mL Eppendorf tube, cold acetone (1200 μL, −20 °C) was added in one portion. The mixture was mixed by upside‐down shaking and sat at −80 °C for 1 h or overnight. The precipitates were collected by centrifugation (15,000 rcf, 15 min, 4 °C), and the supernatant was removed. The pellet was further washed by one additional cycle of acetone (600 μL) addition and centrifugation. After removing the supernatant, the pellet was air‐dried on the bench at rt for 15 min. To the dried pellet, 1X loading buffer (16 μL, prepared from 4X loading buffer, Thermo NP0007 and water) was added. The samples were heated at 95 °C for 5 min, subjected to SDS‐PAGE at 140 V for 45 min, and transferred to a PVDF membrane through a semi‐dry transfer process. The membrane was subjected to chemical blot (alkyne detection) following the procedures in the *Chemical blotting for the detection of alkyne handles* section of Supporting Information.

The serine phosphatase‐targeting modification in aqueous solutions was performed in 1X PBS (12 μL scale) by the addition of cell lysate (0.24 mg/mL final concn from 1.69 mg/mL stock solution in a lysis buffer) and fluorophosphonate‐alkyne **2** (30 μM final concn from 1.2 mM stock solution in DMSO). For some conditions (lanes ii and v in Figure 5D), the cell lysate stock solution was preheated at 80 °C for 5 min before use. The mixture was incubated at rt for 1 h. After the reaction, 4 μL of 4X loading buffer was added, the samples were heated at 95 °C for 5 min, subjected to SDS‐PAGE at 140 V for 45 min, and transferred to a PVDF membrane through a semi‐dry transfer process. The membrane was subjected to chemical blot (alkyne detection) following the procedures in the *chemical blotting for the detection of alkyne handles* section of Supporting Information.

## Conflict of Interests

The authors declare no conflict of interest.

## Supporting information

As a service to our authors and readers, this journal provides supporting information supplied by the authors. Such materials are peer reviewed and may be re‐organized for online delivery, but are not copy‐edited or typeset. Technical support issues arising from supporting information (other than missing files) should be addressed to the authors.

Supporting Information

## References

[1] O. Boutureira , G. J. L. Bernardes , Chem. Rev. 2015, 115, 2174–2195. 25700113 10.1021/cr500399p

[2] C. Dumontet , J. M. Reichert , P. D. Senter , J. M. Lambert , A. Beck , Nat. Rev. Drug Discov. 2023, 22, 641–661. 37308581 10.1038/s41573-023-00709-2

[3] W. Wei , Z. T. Rosenkrans , J. Liu , G. Huang , Q.-Y. Luo , W. Cai , Chem. Rev. 2020, 120, 3787–3851. 32202104 10.1021/acs.chemrev.9b00738 PMC7265988

[4] N. Burger , E. T. Chouchani , Curr. Opin. Chem. Biol. 2024, 79, 102435. 38382148 10.1016/j.cbpa.2024.102435

[5] E. K. Keenan , D. K. Zachman , M. D. Hirschey , Mol. Cell 2021, 81, 1868–1878. 33798408 10.1016/j.molcel.2021.03.015 PMC8106652

[6] J. N. deGruyter , L. R. Malins , P. S. Baran , Biochemistry 2017, 56, 3863–3873. 28653834 10.1021/acs.biochem.7b00536 PMC5792174

[7] J. A. Ubersax , J. E. Ferrell Jr , Nat. Rev. Mol. Cell Biol. 2007, 8, 530–541. 17585314 10.1038/nrm2203

[8] Y. Liu , M. P. Patricelli , B. F. Cravatt , Proc. Natl. Acad. Sci. U.S.A. 1999, 96, 14694–14699. 10611275 10.1073/pnas.96.26.14694 PMC24710

[9] J. C. Vantourout , S. R. Adusumalli , K. W. Knouse , D. T. Flood , A. Ramirez , N. M. Padial , A. Istrate , K. Maziarz , J. N. deGruyter , R. R. Merchant , J. X. Qiao , M. A. Schmidt , M. J. Deery , M. D. Eastgate , P. E. Dawson , G. J. L. Bernardes , P. S. Baran , J. Am. Chem. Soc. 2020, 142, 17236–17242. 32965106 10.1021/jacs.0c05595 PMC8350984

[10] R. G. Smith , A. Vanterpool , H. J. Kulak , Can. J. Chem. 1969, 47, 2015–2019.

[11] K. C. K. Swamy , N. N. B. Kumar , E. Balaraman , K. V. P. P. Kumar , Chem. Rev. 2009, 109, 2551–2651. 19382806 10.1021/cr800278z

[12] T. Arakawa , Y. Kita , S. N. Timasheff , Biophys. Chem. 2007, 131, 62–70. 17904724 10.1016/j.bpc.2007.09.004

[13] H. M. El-Shaffey , E. J. Gross , Y. D. Hall , J. Ohata , J. Am. Chem. Soc. 2021, 143, 12974–12979. 34387473 10.1021/jacs.1c06092

[14] Z. M. Nizam , A. M. Stowe , J. K. Mckinney , J. Ohata , Chem. Commun. 2023, 59, 12160–12163. 10.1039/d3cc03825d 37743738

[15] B. M. Colella , J. Ohata , Synlett 2023, 10.1055/a-2212-7816.

[16] M. Nuruzzaman , B. M. Colella , C. P. Uzoewulu , A. E. Meo , E. J. Gross , S. Ishizawa , S. Sana , H. Zhang , M. E. Hoff , B. T. W. Medlock , E. C. Joyner , S. Sato , E. A. Ison , Z. Li , J. Ohata , J. Am. Chem. Soc. 2024, 146, 6773–6783. 38421958 10.1021/jacs.3c13447

[17] J. Ohata , M. Nuruzzaman , B. Colella , Z. M. Nizam , I. Cho , J. Zagorski , RSC Chem. Biol. 2024, 5, 963–969. 39234575 10.1039/d4cb00142g PMC11368038

[18] M. G. Núñez , A. J. M. Farley , D. J. Dixon , J. Am. Chem. Soc. 2013, 135, 16348–16351. 24107070 10.1021/ja409121s PMC3931333

[19] I. Cruz-Solis , C. C. Ibarra-Herrera , M. del R. Rocha-Pizaña , D. Luna-Vital , Green Protein Processing Technologies from Plants: Novel Extraction and Purification Methods for Product Development (Eds.: A. J. Hernández-Álvarez , M. Mondor , M. G. Nosworthy ), Springer International Publishing, Cham, 2023, pp. 1–29.

[20] C. Ma , E. Gerhard , D. Lu , J. Yang , Biomaterials 2018, 178, 383–400. 29759730 10.1016/j.biomaterials.2018.05.003 PMC6366999

[21] P. J. Kerber , R. Nuñez , D. R. Jensen , A. L. Zhou , F. C. Peterson , R. B. Hill , B. F. Volkman , B. C. Smith , Methods in Enzymology (Ed.: M. Lloyd ), Academic Press, 2023, pp. 285–310. 10.1016/bs.mie.2023.06.018 PMC10657026 37858532

[22] C. Jorge , B. S. Marques , K. G. Valentine , A. J. Wand , Methods in Enzymology (Ed.: A. J. Wand ), Academic Press, 2019, pp. 77–101. 10.1016/bs.mie.2018.09.040 PMC6358200 30638541

[23] D. C. Rodriguez Camargo , K. J. Korshavn , A. Jussupow , K. Raltchev , D. Goricanec , M. Fleisch , R. Sarkar , K. Xue , M. Aichler , G. Mettenleiter , A. K. Walch , C. Camilloni , F. Hagn , B. Reif , A. Ramamoorthy , eLife 2017, 6, e31226. 29148426 10.7554/eLife.31226 PMC5706959

[24] L. P. Kozlowski , Nucleic Acids Res. 2017, 45, D1112–D1116. 27789699 10.1093/nar/gkw978 PMC5210655

[25] J. R. Gaffen , J. N. Bentley , L. C. Torres , C. Chu , T. Baumgartner , C. B. Caputo , Chem 2019, 5, 1567–1583.

[26] L. A. Wolzak , J. I. van der Vlugt , K. J. van den Berg , J. N. H. Reek , M. Tromp , T. J. Korstanje , ChemCatChem 2020, 12, 5229–5235.

[27] B. N. Atkinson , J. M. J. Williams , Tetrahedron Lett. 2014, 55, 6935–6938.

[28] J. S. Ram , P. H. Maurer , Arch. Biochem. Biophys. 1958, 74, 119–130. 13522230 10.1016/0003-9861(58)90206-6

[29] A. Previero , L.-G. Barry , M.-A. Coletti-Previero , Biochim. Biophys. Acta 1972, 263, 7–13. 5062506 10.1016/0005-2795(72)90154-7

[30] C. A. Guy , G. B. Fields , Methods in Enzymology, Academic Press, 1997, pp. 67–83. 10.1016/s0076-6879(97)89044-1 9353718

[31] S. Ishizawa , K. Fujimura , K. Oisaki , S. Sato , J. Ohata , ChemRxiv 2024, 10.26434/chemrxiv-2024-jxph9. PMC13042321 41930370

[32] J. Ohata , F. Vohidov , Z. T. Ball , Mol. Biosyst. 2015, 11, 2846–2849. 26325302 10.1039/c5mb00510h PMC4605879

[33] N. S. Yadavalli , N. Borodinov , C. K. Choudhury , T. Quiñones-Ruiz , A. M. Laradji , S. Tu , I. K. Lednev , O. Kuksenok , I. Luzinov , S. Minko , ACS Catal. 2017, 7, 8675–8684.

[34] X. Wang , N. S. Yadavalli , A. M. Laradji , S. Minko , Macromolecules 2018, 51, 5039–5047.

[35] H. Ogawa , H. Miyazaki , M. Kimura , J. Invest. Dermatol. 1971, 57, 111–116. 5109987 10.1111/1523-1747.ep12349624

[36] D. da Silva Freitas , J. Abrahão-Neto , Int. J. Pharm. 2010, 392, 111–117. 20307635 10.1016/j.ijpharm.2010.03.036

[37] F. Baron , F. Nau , C. Guérin-Dubiard , S. Bonnassie , M. Gautier , S. C. Andrews , S. Jan , Food Microbiol. 2016, 53, 82–93. 26678134 10.1016/j.fm.2015.09.009

[38] T. Podzimek , J. Matoušek , P. Lipovová , P. Poučková , V. Spiwok , J. Šantrůček , Plant Sci. 2011, 180, 343–351. 21421379 10.1016/j.plantsci.2010.10.006

[39] R. W. Mason , G. D. J. Green , A. J. Barrett , Biochem. J. 1985, 226, 233–241. 3977867 10.1042/bj2260233 PMC1144697

[40] S. T. Freer , J. Kraut , J. D. Robertus , H. T. Wright , Nguyen-Huu-Xuong , Biochemistry 1970, 9, 1997–2009. 5442169 10.1021/bi00811a022

[41] It should be noted that a noticeable level of modification on serine using **2** was not observed at an amino acid level in LC-MS analysis, even in high loading of the reagent, extended reaction time, and elevated reaction temperature (Figure S102), implying that serine modification with fluorophosphonates is not chemoselective to the alkyl hydroxyl group under the tested conditions.

[42] D. A. Bachovchin , J. T. Mohr , A. E. Speers , C. Wang , J. M. Berlin , T. P. Spicer , V. Fernandez-Vega , P. Chase , P. S. Hodder , S. C. Schürer , D. K. Nomura , H. Rosen , G. C. Fu , B. F. Cravatt , Proc. Natl. Acad. Sci. U.S.A. 2011, 108, 6811–6816. 21398589 10.1073/pnas.1015248108 PMC3084096

[43] Y.-L. Wang , S. Liu , Z.-J. Yu , Y. Lei , M.-Y. Huang , Y.-H. Yan , Q. Ma , Y. Zheng , H. Deng , Y. Sun , C. Wu , Y. Yu , Q. Chen , Z. Wang , Y. Wu , G.-B. Li , J. Med. Chem. 2019, 62, 7160–7184. 31269398 10.1021/acs.jmedchem.9b00735

[44] J. Yang , D. Korovesis , S. Ji , J. P. Kahler , R. Vanhoutte , S. H. L. Verhelst , Isr. J. Chem. 2023, 63, e202200094.

[45] C. Wang , D. Abegg , B. G. Dwyer , A. Adibekian , ChemBioChem 2019, 20, 2212–2216. 30968522 10.1002/cbic.201900126

[46] M. J. Suskiewicz , BioEssays 2024, 46, 2300178. 10.1002/bies.202300178 38247183

[47] L. N. Johnson , R. J. Lewis , Chem. Rev. 2001, 101, 2209–2242. 11749371 10.1021/cr000225s

[48] H. Yamamoto , Inventing Reactions (Ed.: L. J. Gooßen ), Springer, Berlin, Heidelberg, 2013, pp. 315–334.

