# Supplementary material for: Catalytic Serine Labeling in Nonaqueous, Acidic Media
Source: Chemistry. 2025 Feb 9;31(12):e202404002. doi: 10.1002/chem.202404002 (PMC11855257; doi:10.1002/chem.202404002)
Supplement: Supplementary file 1 — Supporting Information [file CHEM-31-e202404002-s001.pdf]

Chemistry–A European Journal

Supporting Information

Catalytic Serine Labeling in Nonaqueous, Acidic Media

Seiya Ishizawa, Chiamaka P. Uzoewulu, Yume Iwakura, Anuja Koirala, Shinichi Sato, and Jun Ohata*

Supporting Information

Contents

Supporting figures....................2
General information....................105
Materials and reagents....................105
Cell culture....................105
List of materials....................106
Instrumentation....................107
Experimental procedures....................110
Preparative synthesis of small molecules....................118
Organic synthesis procedures....................118
References for Electronic Supplementary Information....................119

Supporting figures

Background peaks in Liquid-chromatography mass spectrometry (LC-MS) analysis

Background peaks shown in the figures below were observed when carboxylic acids were incubated in 1.7 mL Eppendorf tubes. With 24 h of incubation, the intensities of peaks were increased and more peaks appeared compared to a control experiment without incubation.

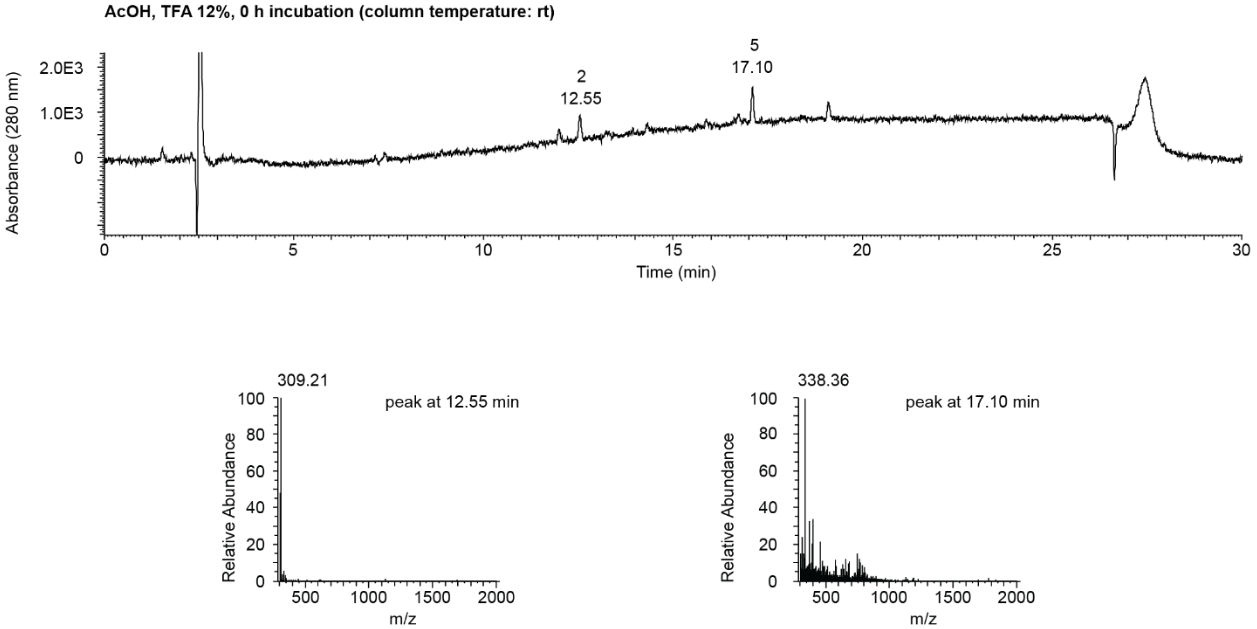

Figure S1. Liquid-chromatography mass spectrometry (LC-MS) analysis of mixtures of acetic acid with TFA (column oven temperature: rt). Conditions: TFA (12% v/v, 1.6 M) was incubated in acetic acid for 0 h (no incubation control). After the incubation, the mixture was analyzed by following the procedure in the *analysis of peptide samples* section. Labels 2 and 5 in the chromatograms indicate representative peaks. 2=12.6 min, 5=17.1 min when LC column oven temperature is at room temperature (rt).

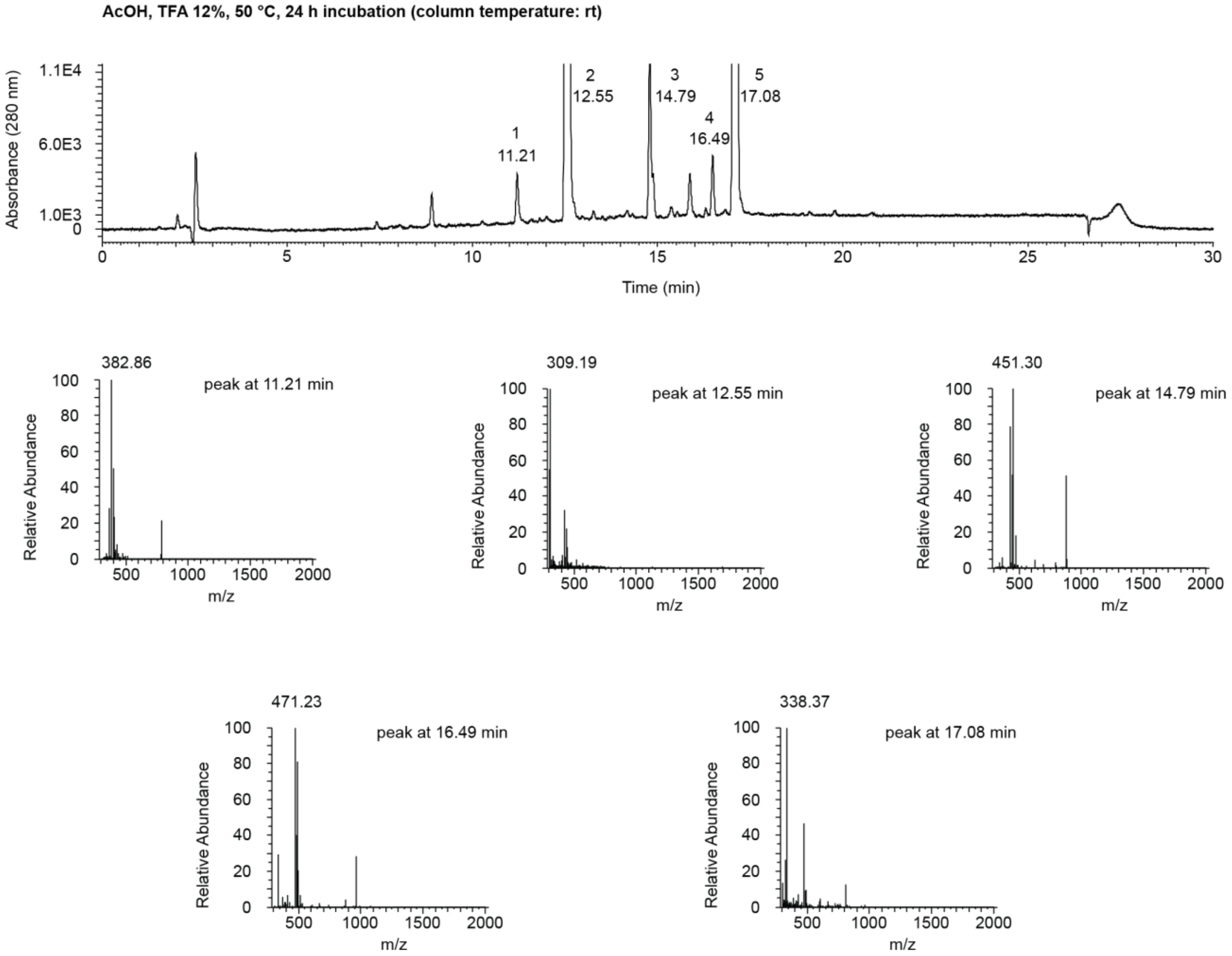

Figure S2. Liquid-chromatography mass spectrometry (LC-MS) analysis of acetic acid treated with TFA (column oven temperature: rt). Conditions: TFA (12% v/v, 1.6 M) was incubated in acetic acid at 50 °C for 24 h. After the incubation, the mixture was analyzed by following the procedure in the *analysis of peptide samples* section. Labels 1, 2, 3, 4, and 5 in the chromatograms indicate representative peaks. 1=11.2 min, 2=12.6 min, 3=14.8 min, 4=16.5 min, 5=17.1 min when LC column oven temperature is at room temperature (rt).

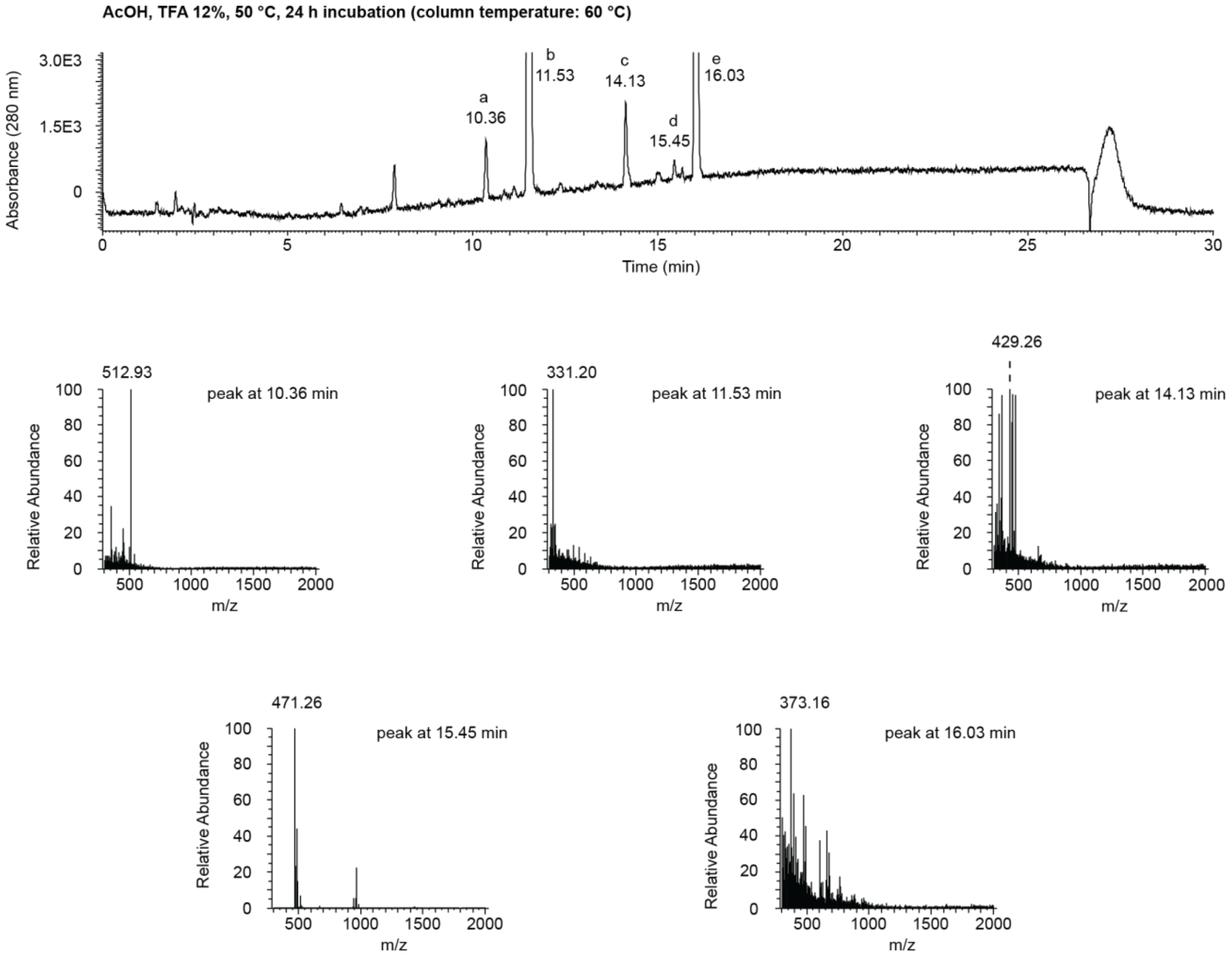

Figure S3. Liquid-chromatography mass spectrometry (LC-MS) analysis of acetic acid treated with TFA (column oven temperature: 60 °C). Conditions: TFA was incubated in acetic acid at 50 °C for 24 h. After the incubation, the mixture was analyzed by following the procedure in the *analysis of peptide samples* section. Labels a, b, c, d, and e in the chromatograms indicate representative peaks. a=10.4 min, b=11.5 min, c=14.1 min, d=15.4 min, e=16.0 min when LC column oven temperature is 60 °C.

Secondary containers for the acid-catalyzed acylation reaction

A 50 mL plastic tube (Fisherbrand 05-539-8) was employed as a secondary container for the acid-catalyzed acylation reactions in 1.7 mL Eppendorf tubes (Axygen MCT-175-C) to avoid the spread of volatile acid. The secondary container was covered with aluminum foil during the reaction (See *Secondary containers for the acid-catalyzed acylation reaction* figure).

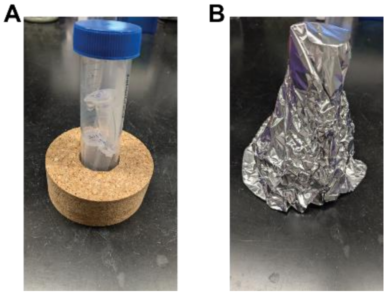

Figure S4. Secondary containers for the acid-catalyzed acylation reaction. (A) 1.7 mL Eppendorf tubes in the 50 mL plastic tube. (B) The 50 mL plastic tube covered with aluminum foil prior to the reaction.

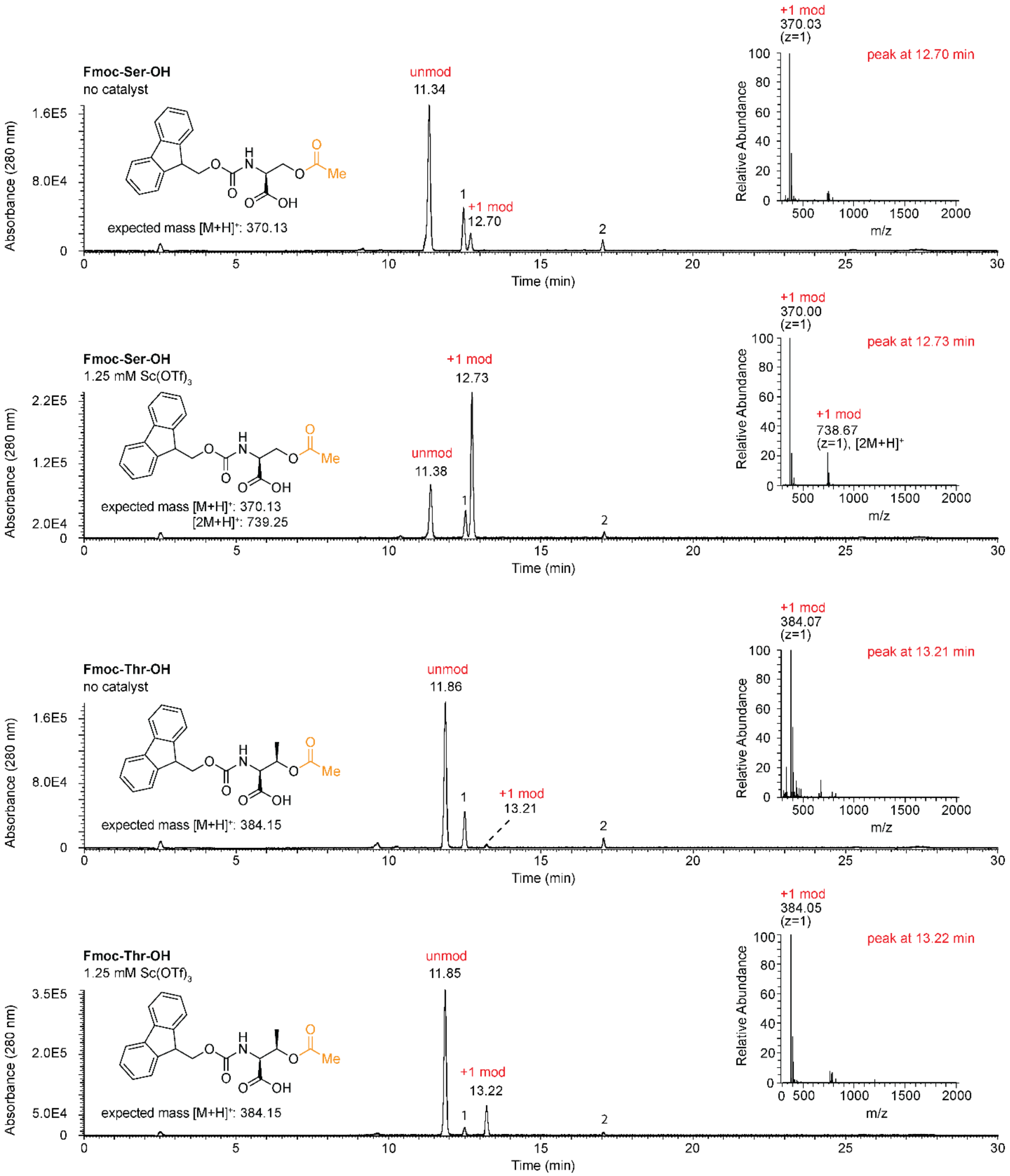

Figure S5. Liquid-chromatography mass spectrometry (LC-MS) analysis of reaction mixtures of modification of Fmoc-Ser-OH and Fmoc-Thr-OH. The reaction without the catalyst (top) and the reaction with the catalyst (bottom) are shown. Typical modification conditions: amino acid (5 mM final concn from 500 mM stock solution in 1,4-dioxane) and scandium (III) triflate (1.25 mM final concn from 125 mM stock solution in 1,4-dioxane) in acetic acid for 24 h at 50 °C. Labels 1 and 2 in the chromatograms indicate peaks observed even in a blank sample shown in the *Liquid-chromatography mass spectrometry (LC-MS) analysis of acetic acid treated with TFA (column oven temperature: rt)* figure. 1=~12.5 min, 2=~17.1 min when LC column oven temperature is at rt.

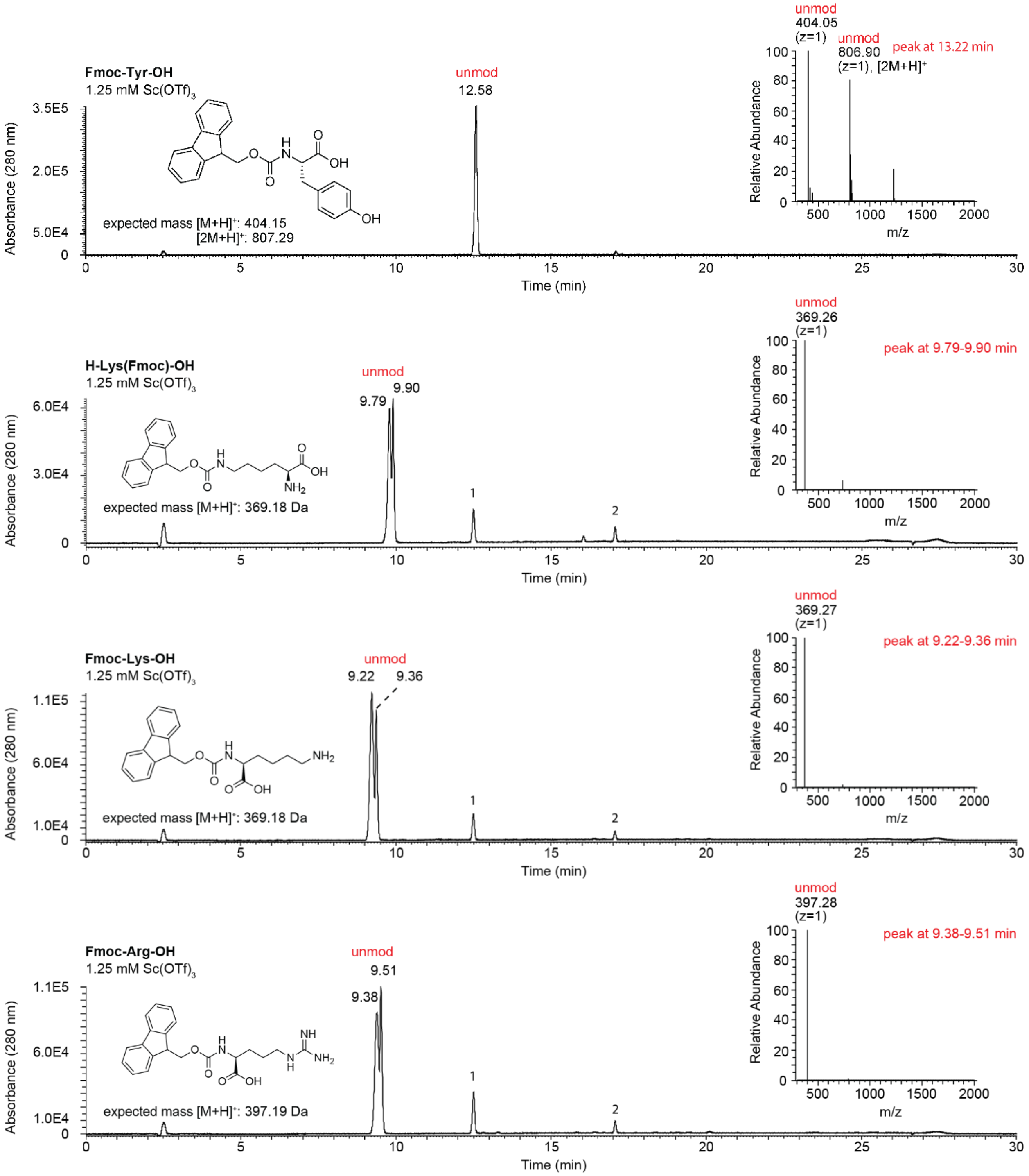

Figure S6. Liquid-chromatography mass spectrometry (LC-MS) analysis of reaction mixtures of modification of Fmoc-Tyr-OH, H-Lys(Fmoc)-OH, Fmoc-Lys-OH, and Fmoc-Arg-OH. No modification was observed. Reaction conditions: amino acid (see below for the concentration) and scandium (III) triflate (1.25 mM final concn from 125 mM stock solution in 1,4-dioxane) in acetic acid for 24 h at 50 °C. Amino acid concentrations: Fmoc-Tyr-OH (5 mM final concn from 500 mM stock solution in 1,4-dioxane), H-Lys(Fmoc)-OH (5 mM final concn from 100 mM stock suspension in acetic acid), Fmoc-Lys-OH (5 mM final concn from 500 mM stock solution in acetic acid), Fmoc-Arg-OH (5 mM final concn from 500 mM stock solution in acetic acid). Labels 1 and 2 in the chromatograms indicate peaks observed even in a blank sample shown in the *Liquid-chromatography mass spectrometry (LC-MS) analysis of acetic acid treated with TFA (column oven temperature: rt)* figure. LC column oven temperature is at rt.

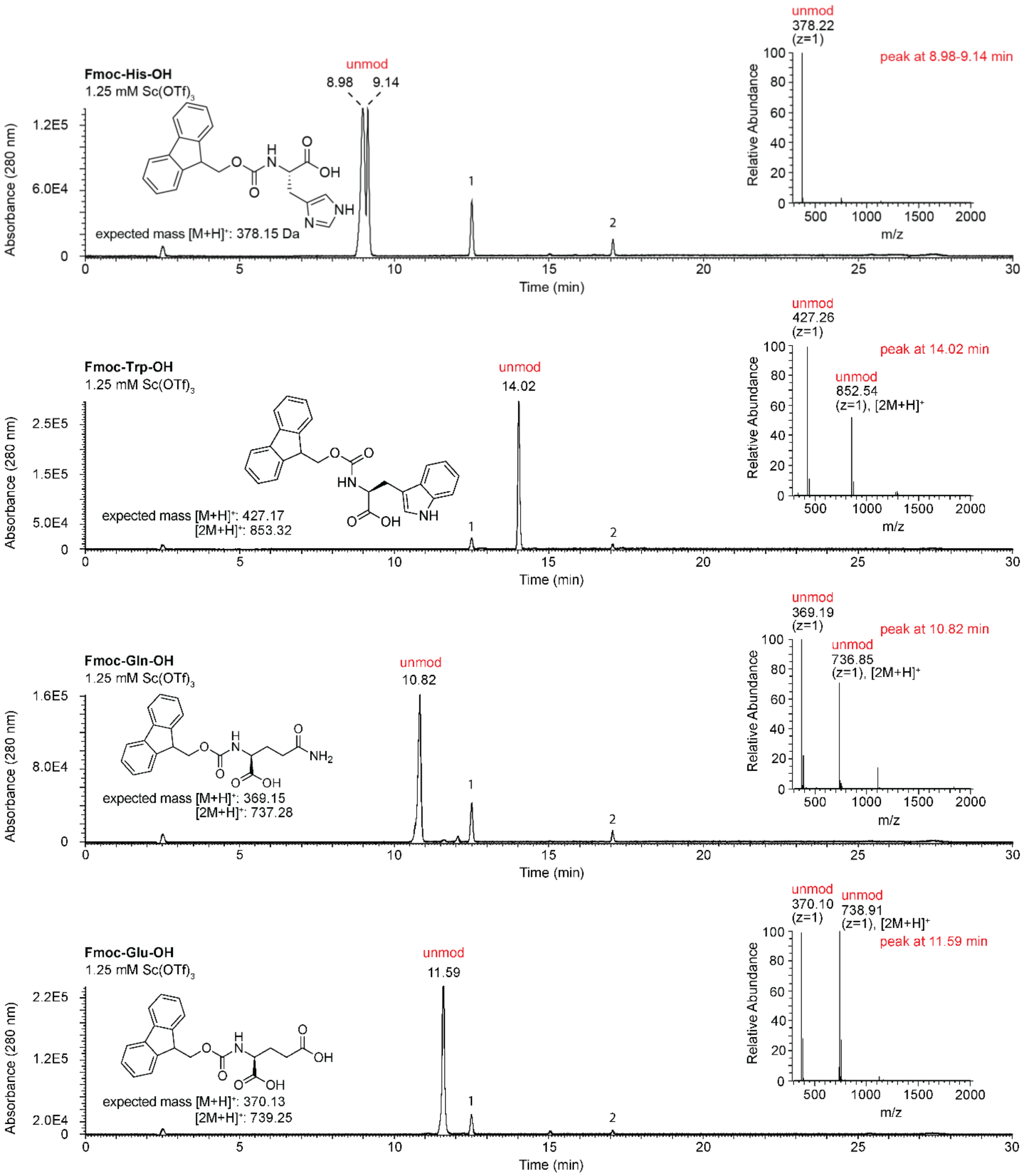

Figure S7. Liquid-chromatography mass spectrometry (LC-MS) analysis of reaction mixtures of modification of Fmoc-His-OH, Fmoc-Trp-OH, Fmoc-Gln-OH, and Fmoc-Glu-OH. No modification was observed. Reaction conditions: amino acid (see below for the concentration) and scandium (III) triflate (1.25 mM final concn from 125 mM stock solution in 1,4-dioxane) in acetic acid for 24 h at 50 °C. Amino acid concentrations: Fmoc-His-OH (5 mM final concn from 500 mM stock suspension in acetonitrile), Fmoc-Trp-OH (5 mM final concn from 500 mM stock solution in 1,4-dioxane), Fmoc-Gln-OH (5 mM final concn from 500 mM stock suspension in acetonitrile), Fmoc-Glu-OH (5 mM final concn from 500 mM stock solution in 1,4-dioxane). Labels 1 and 2 in the chromatograms indicate peaks observed even in a blank sample shown in the *Liquid-chromatography mass spectrometry (LC-MS) analysis of acetic acid treated with TFA (column oven temperature: rt)* figure. 1=~12.5 min, 2=~17.1 min when LC column oven temperature is at rt.

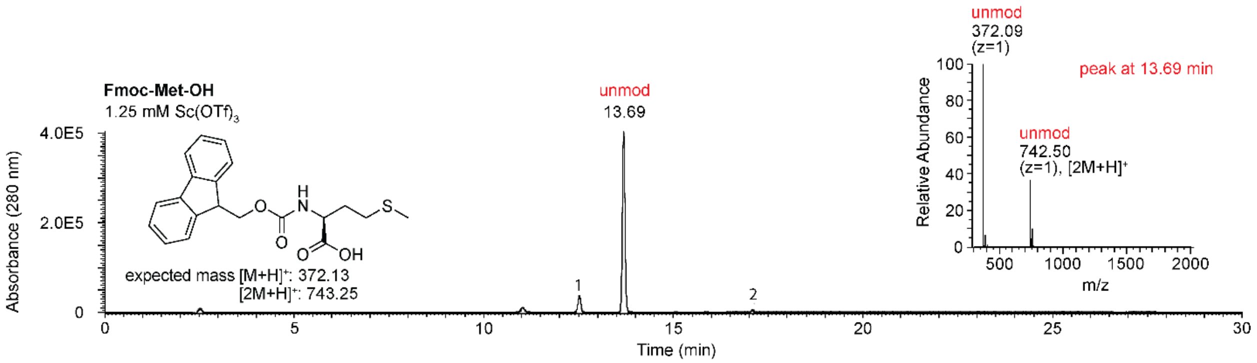

Figure S8. Liquid-chromatography mass spectrometry (LC-MS) analysis of reaction mixtures of modification of Fmoc-Met-OH. No modification was observed. Reaction conditions: amino acid (5 mM final concn from 500 mM stock solution in 1,4-dioxane) and scandium (III) triflate (1.25 mM) in acetic acid for 24 h at 50 °C. Labels 1 and 2 in the chromatograms indicate peaks observed even in a blank sample shown in the *Liquid-chromatography mass spectrometry (LC-MS) analysis of acetic acid treated with TFA (column oven temperature: rt)* figure. 1=~12.5 min, 2=~17.1 min when LC column oven temperature is at rt.

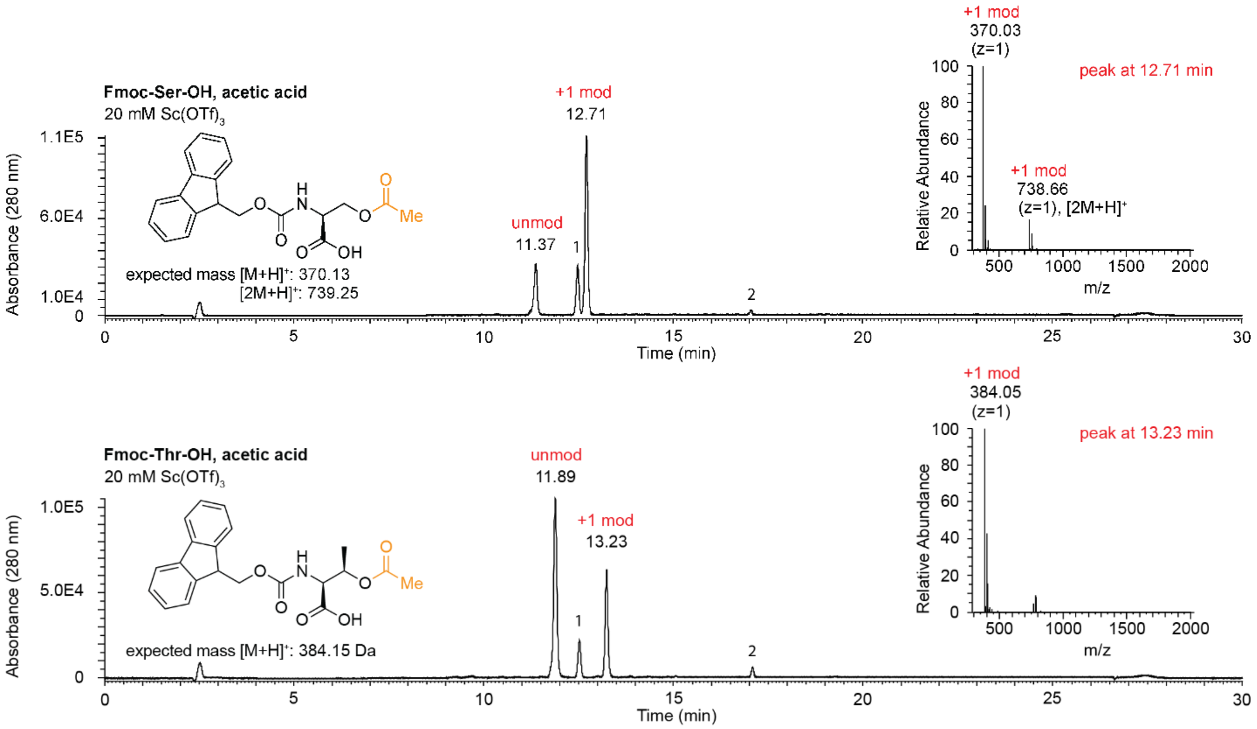

Figure S9. Liquid-chromatography mass spectrometry (LC-MS) analysis of reaction mixtures of modification of Fmoc-Ser-OH and Fmoc-Thr-OH with acetic acid. Reaction conditions: amino acid (5 mM final concn from 500 mM stock solution in 1,4-dioxane), scandium (III) triflate (20 mM final concn from 500 mM stock solution in 1,4-dioxane), and acetic acid (4750 mM final concn from 5 M stock solution in MeCN) were incubated in MeCN for 24 h at 50 °C. Labels 1 and 2 in the chromatograms indicate peaks observed even in a blank sample shown in the *Liquid-chromatography mass spectrometry (LC-MS) analysis of acetic acid treated with TFA (column oven temperature: rt)* figure. 1=~12.5 min, 2=~17.1 min when LC column oven temperature is at rt.

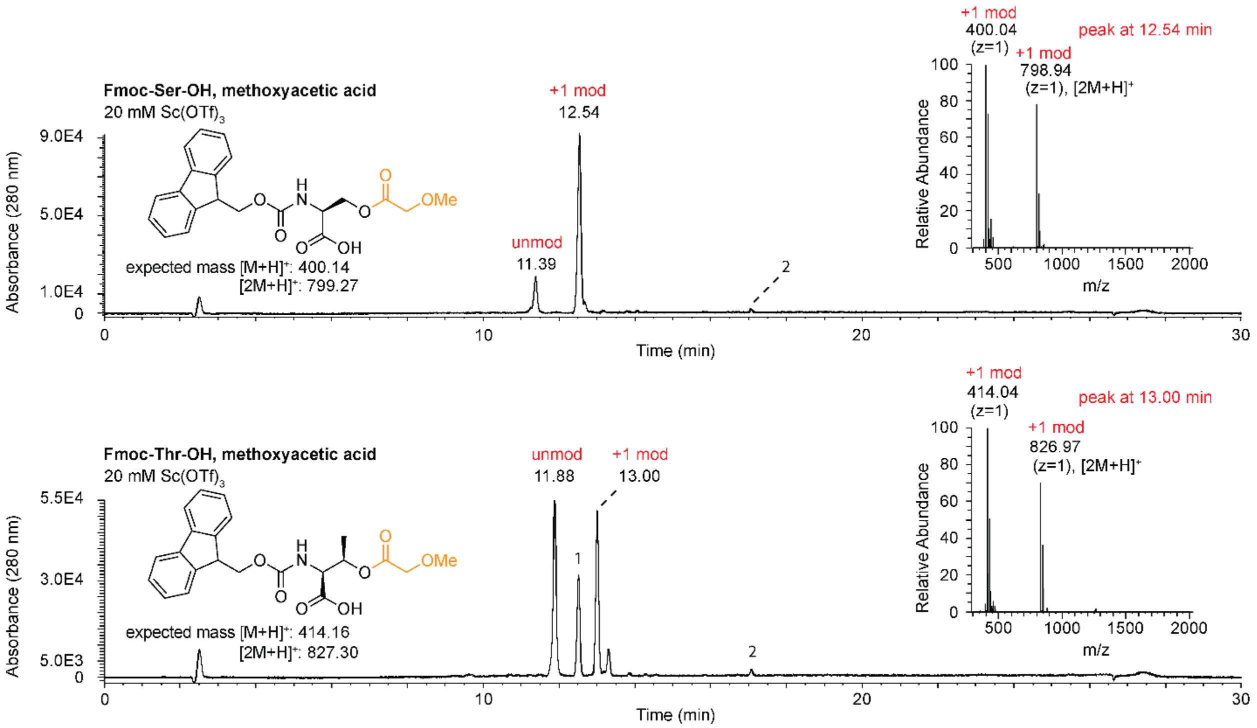

Figure S10. Liquid-chromatography mass spectrometry (LC-MS) analysis of reaction mixtures of modification of Fmoc-Ser-OH, and Fmoc-Thr-OH with methoxyacetic acid **1b**. Reaction conditions: amino acid (5 mM final concn from 500 mM stock solution in 1,4-dioxane), scandium (III) triflate (20 mM final concn from 500 mM stock solution in 1,4-dioxane), and methoxyacetic acid **1b** (4750 mM final concn from 5 M stock solution in MeCN) were incubated in MeCN for 24 h at 50 °C. Labels 1 and 2 in the chromatograms indicate peaks observed even in a blank sample shown in the *Liquid-chromatography mass spectrometry (LC-MS) analysis of acetic acid treated with TFA (column oven temperature: rt)* figure. 1=~12.5 min, 2=~17.1 min when LC column oven temperature is at rt.

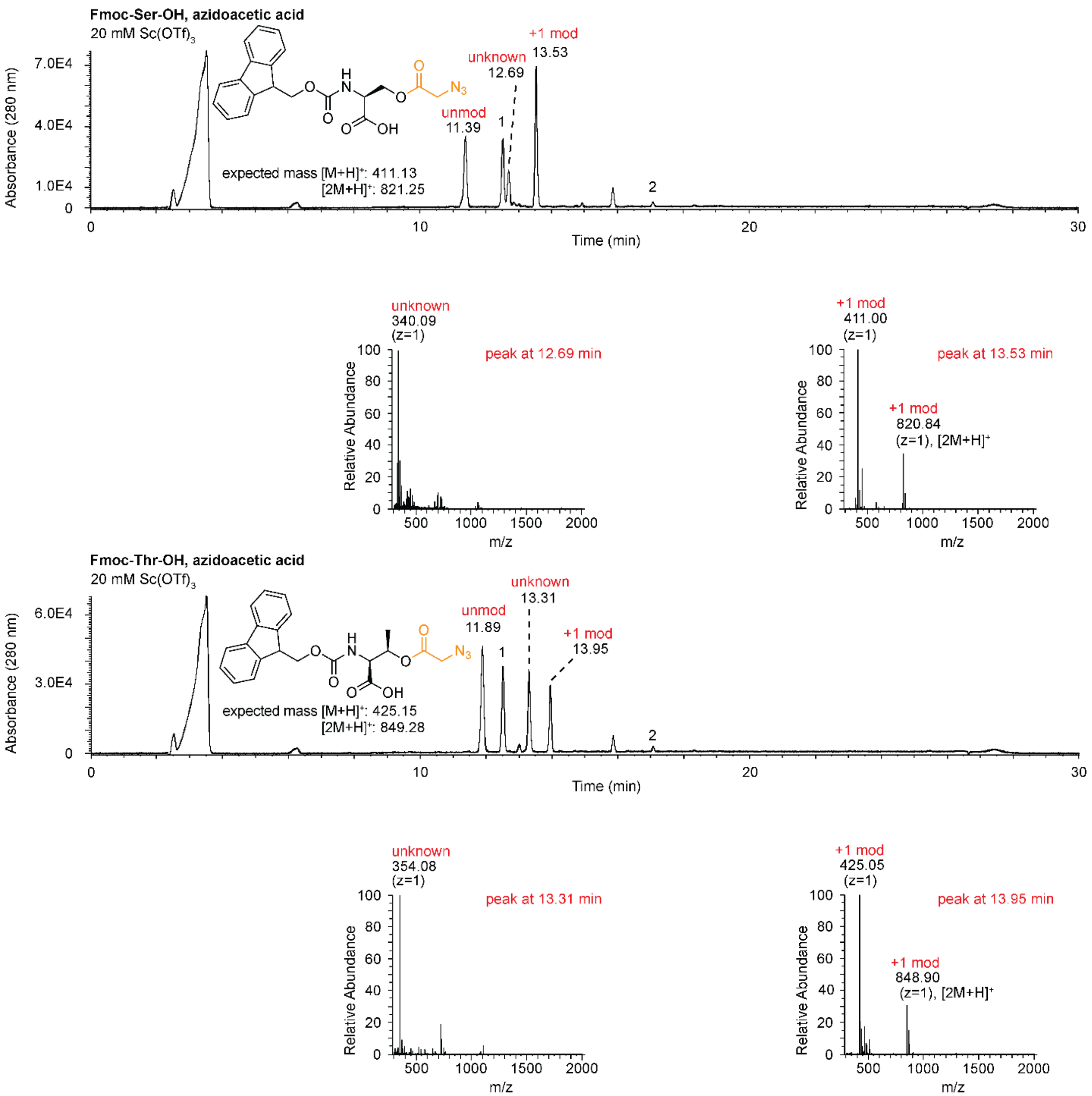

Figure S11. Liquid-chromatography mass spectrometry (LC-MS) analysis of reaction mixtures of modification of Fmoc-Ser-OH and Fmoc-Thr-OH with azidoacetic acid **1c**. Amino acid (5 mM final concn from 500 mM stock solution in 1,4-dioxane), scandium (III) triflate (20 mM final concn from 500 mM stock solution in 1,4-dioxane), and azidoacetic acid **1c** (4750 mM final concn from 5 M stock solution in MeCN) were incubated in MeCN for 24 h at 50 °C. Labels 1 and 2 in the chromatograms indicate peaks observed even in a blank sample shown in the *Liquid-chromatography mass spectrometry (LC-MS) analysis of acetic acid treated with TFA (column oven temperature: rt)* figure. 1=~12.5 min, 2=~17.1 min when LC column oven temperature is at rt.

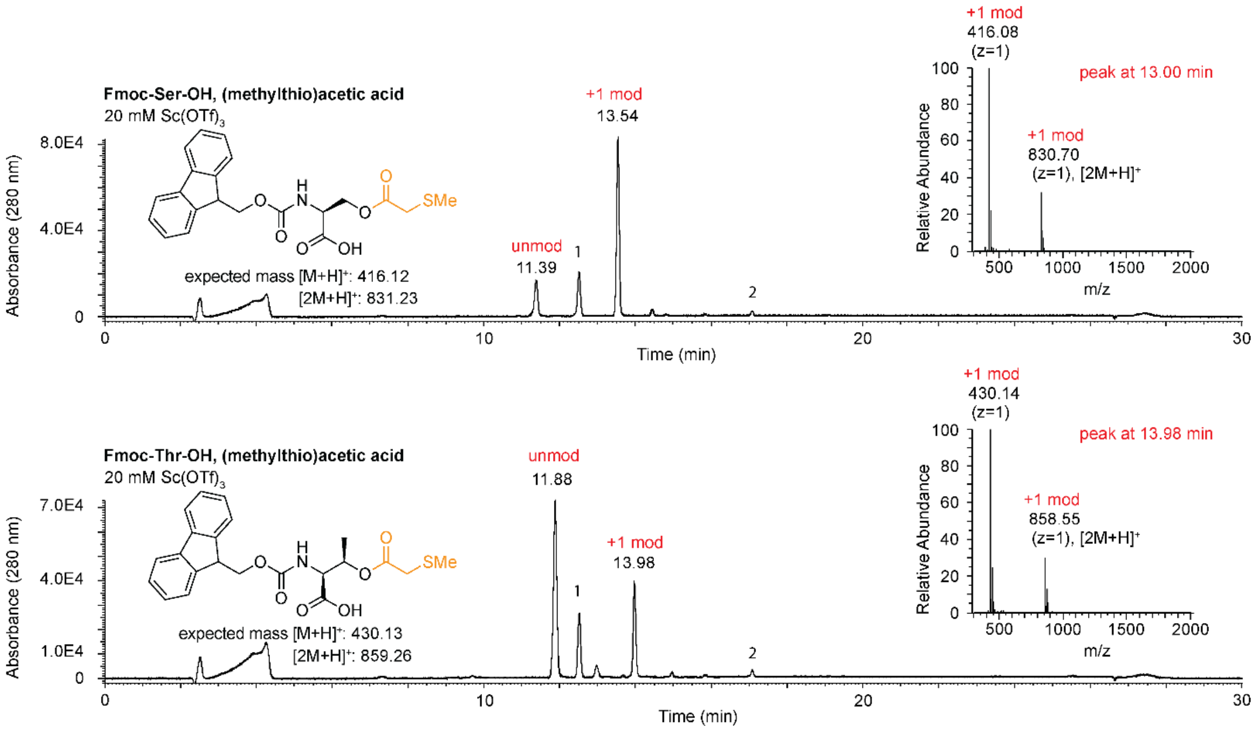

Figure S12. Liquid-chromatography mass spectrometry (LC-MS) analysis of reaction mixtures of modification of Fmoc-Ser-OH and Fmoc-Thr-OH with (methylthio)acetic acid **1d**. Reaction conditions: amino acid (5 mM final concn from 500 mM stock solution in 1,4-dioxane), scandium (III) triflate (20 mM final concn from 500 mM stock solution in 1,4-dioxane), and (methylthio)acetic acid **1d** (4750 mM final concn from 5 M stock solution in MeCN) were incubated in MeCN for 24 h at 50 °C. Labels 1 and 2 in the chromatograms indicate peaks observed even in a blank sample shown in the *Liquid-chromatography mass spectrometry (LC-MS) analysis of acetic acid treated with TFA (column oven temperature: rt)* figure. 1=~12.5 min, 2=~17.1 min when LC column oven temperature is at rt.

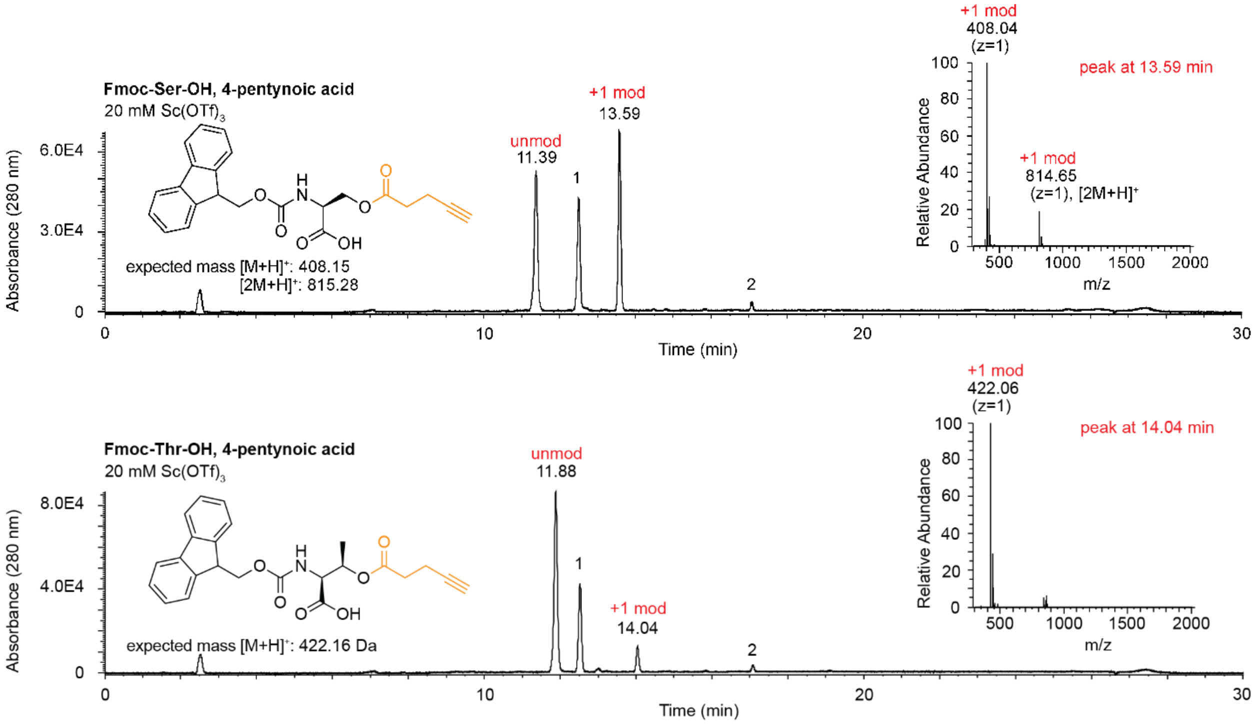

Figure S13. Liquid-chromatography mass spectrometry (LC-MS) analysis of reaction mixtures of modification of Fmoc-Ser-OH and Fmoc-Thr-OH with 4-pentynoic acid **1e**. Reaction conditions: amino acid (5 mM final concn from 500 mM stock solution in 1,4-dioxane), scandium (III) triflate (20 mM final concn from 500 mM stock solution in 1,4-dioxane), and 4-pentynoic acid **1e** (4750 mM final concn from 5 M stock solution in MeCN) were incubated in MeCN for 24 h at 50 °C. Labels 1 and 2 in the chromatograms indicate peaks observed even in a blank sample shown in the *Liquid-chromatography mass spectrometry (LC-MS) analysis of acetic acid treated with TFA (column oven temperature: rt)* figure. 1=~12.5 min, 2=~17.1 min when LC column oven temperature is at rt.

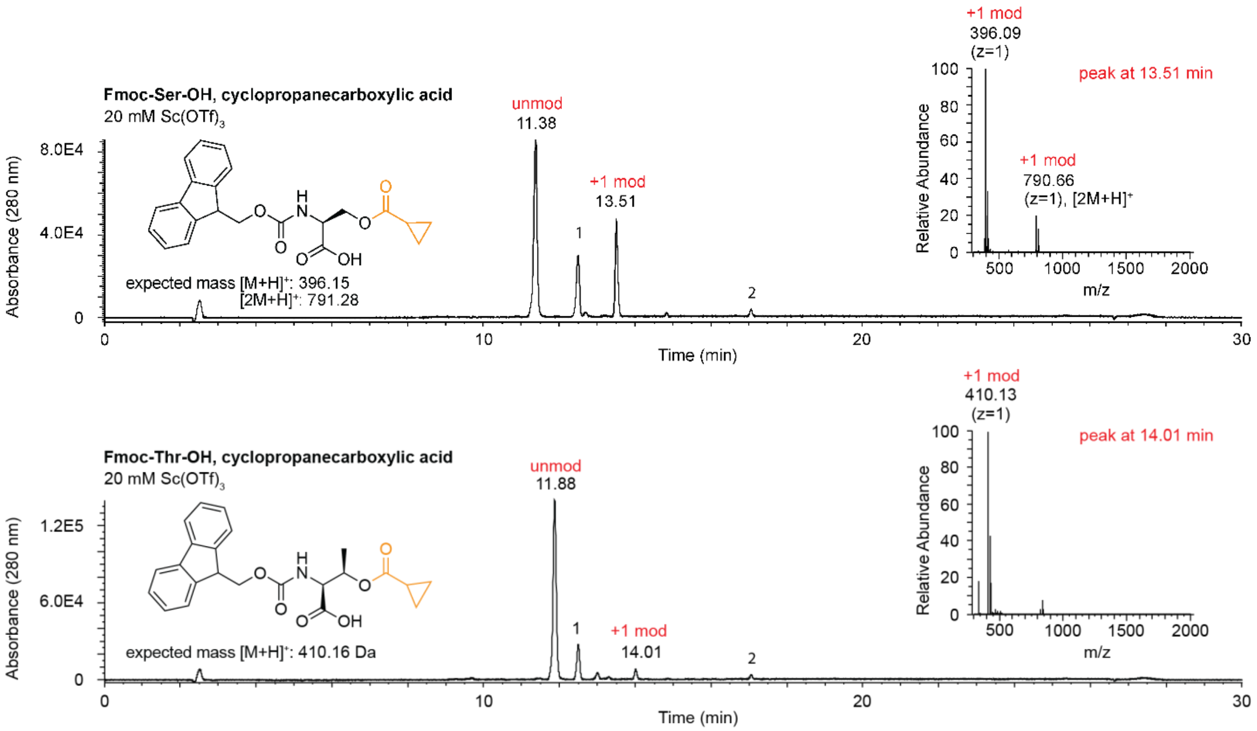

Figure S14. Liquid-chromatography mass spectrometry (LC-MS) analysis of reaction mixtures of modification of Fmoc-Ser-OH and Fmoc-Thr-OH with cyclopropanecarboxylic acid **1f**. Reaction conditions: amino acid (5 mM final concn from 500 mM stock solution in 1,4-dioxane), scandium (III) triflate (20 mM final concn from 500 mM stock solution in 1,4-dioxane), and cyclopropanecarboxylic acid **1f** (4750 mM final concn from 5 M stock solution in MeCN) were incubated in MeCN for 24 h at 50 °C. Labels 1 and 2 in the chromatograms indicate peaks observed even in a blank sample shown in the *Liquid-chromatography mass spectrometry (LC-MS) analysis of acetic acid treated with TFA (column oven temperature: rt)* figure. 1=~12.5 min, 2=~17.1 min when LC column oven temperature is at rt.

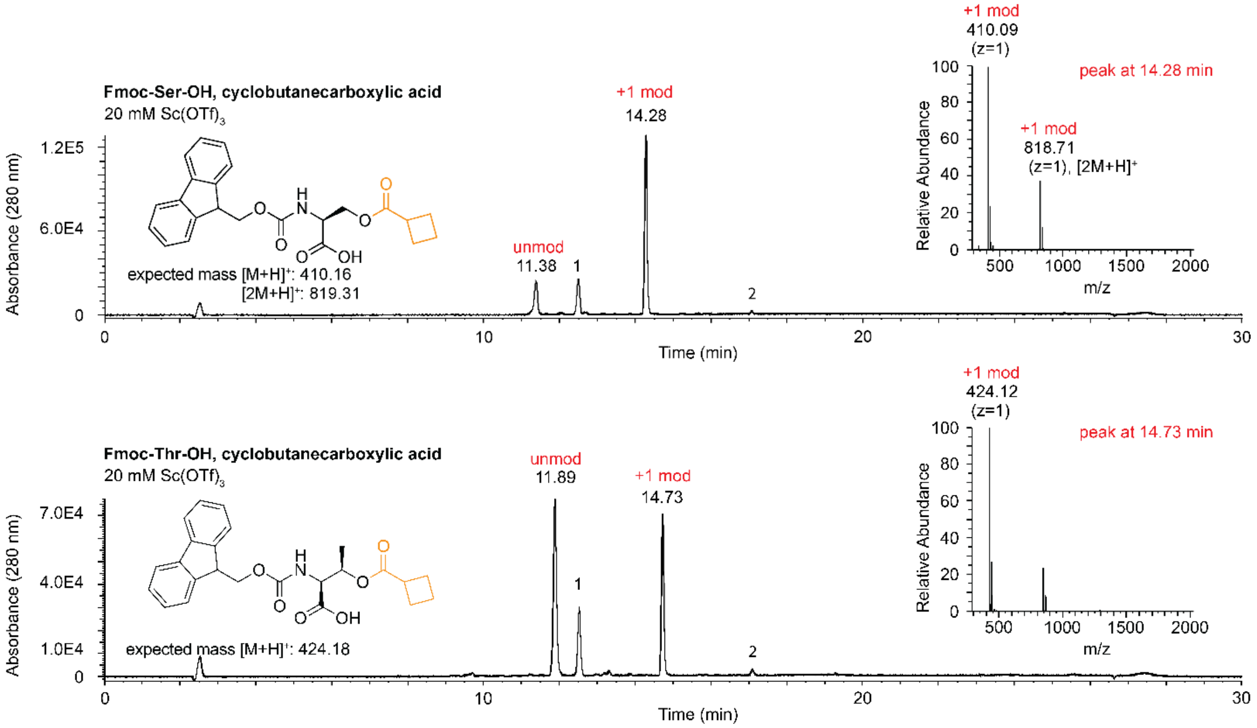

Figure S15. Liquid-chromatography mass spectrometry (LC-MS) analysis of reaction mixtures of modification of Fmoc-Ser-OH and Fmoc-Thr-OH with cyclobutanecarboxylic acid **1g**. Reaction conditions: amino acid (5 mM final concn from 500 mM stock solution in 1,4-dioxane), scandium (III) triflate (20 mM final concn from 500 mM stock solution in 1,4-dioxane), and cyclobutanecarboxylic acid **1g** (4750 mM final concn from 5 M stock solution in MeCN) were incubated in MeCN for 24 h at 50 °C. Labels 1 and 2 in the chromatograms indicate peaks observed even in a blank sample shown in the *Liquid-chromatography mass spectrometry (LC-MS) analysis of acetic acid treated with TFA (column oven temperature: rt)* figure.. 1=~12.5 min, 2=~17.1 min when LC column oven temperature is at rt.

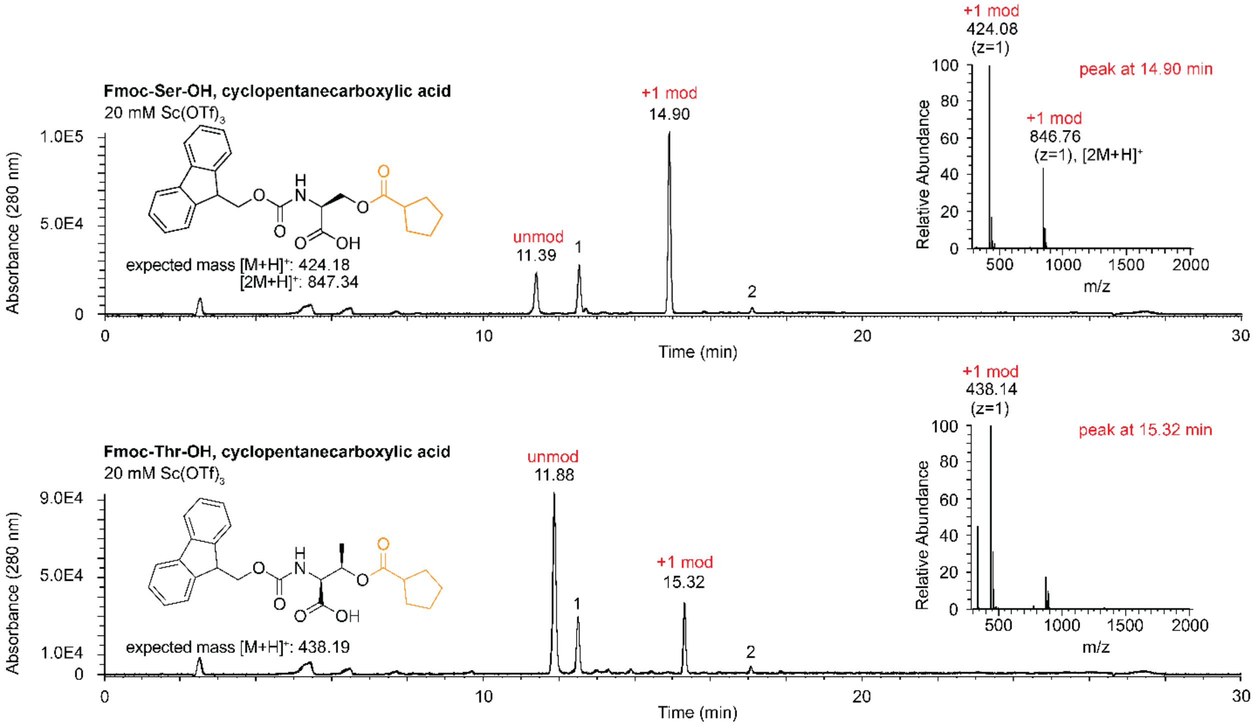

Figure S16. Liquid-chromatography mass spectrometry (LC-MS) analysis of reaction mixtures of modification of Fmoc-Ser-OH and Fmoc-Thr-OH with cyclopentanecarboxylic acid **1h**. Reaction conditions: amino acid (5 mM final concn from 500 mM stock solution in 1,4-dioxane), scandium (III) triflate (20 mM final concn from 500 mM stock solution in 1,4-dioxane), and cyclopentanecarboxylic acid **1h** (4750 mM final concn from 5 M stock solution in MeCN) were incubated in MeCN for 24 h at 50 °C. Labels 1 and 2 in the chromatograms indicate peaks observed even in a blank sample shown in the *Liquid-chromatography mass spectrometry (LC-MS) analysis of acetic acid treated with TFA (column oven temperature: rt)* figure. 1=~12.5 min, 2=~17.1 min when LC column oven temperature is at rt.

solvent	conversion (%)
neat acetic acid	87
acetonitrile (MeCN)	81

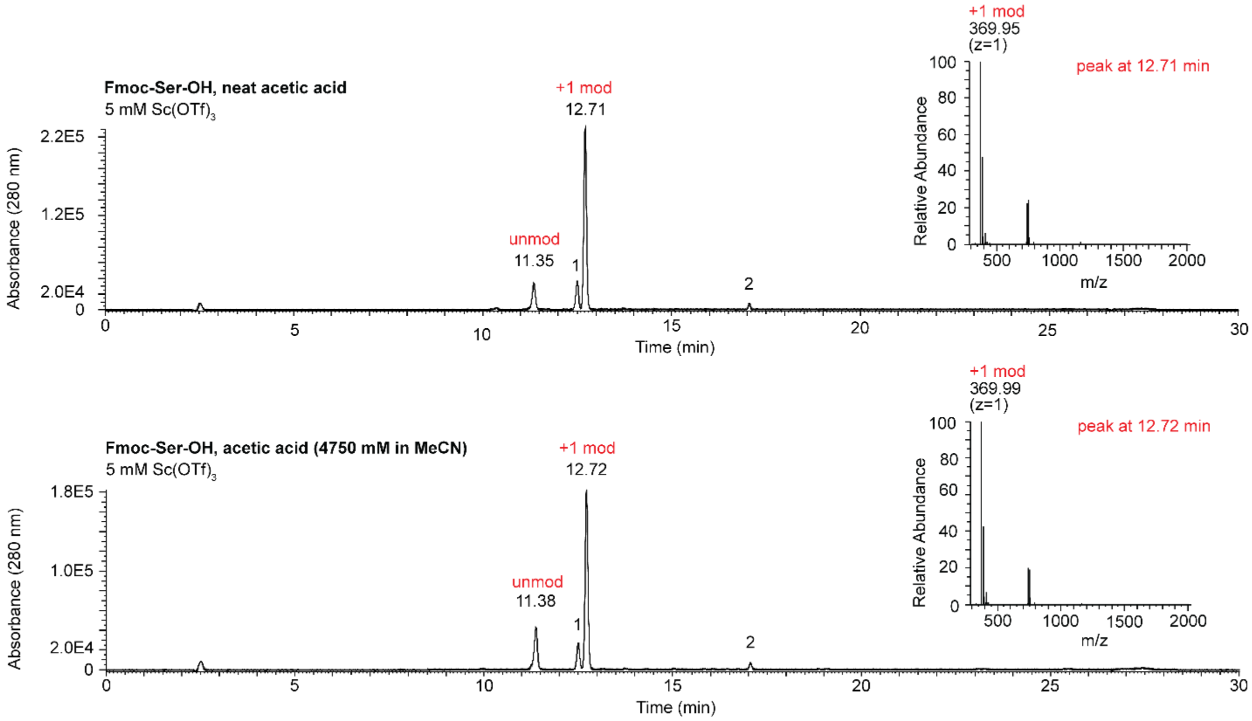

Figure S17. Summary of reaction conversions and liquid-chromatography mass spectrometry (LC-MS) analysis of reaction mixtures of modification of Fmoc-Ser-OH. Reaction conditions: Fmoc-Ser-OH (5 mM final concn from 500 mM stock solution in 1,4-dioxane) and scandium (III) triflate (5 mM final concn from 500 mM stock solution in 1,4-dioxane) were incubated in either neat acetic acid or acetic acid (4750 mM final concn) in acetonitrile (MeCN) for 16 h 40 min at 50 °C. After the incubation, the mixture was analyzed by following the procedure in the *analysis of amino acid samples* section. Labels 1 and 2 in the chromatograms indicate peaks observed even in a blank sample shown in the *Liquid-chromatography mass spectrometry (LC-MS) analysis of acetic acid treated with TFA (column oven temperature: rt)* figure. 1=~12.5 min, 2=~17.1 min when LC column oven temperature is at rt. The conversions were calculated by dividing the product peak area by the sum of the product peak area and starting material peak area of UV chromatograms (liquid chromatography).

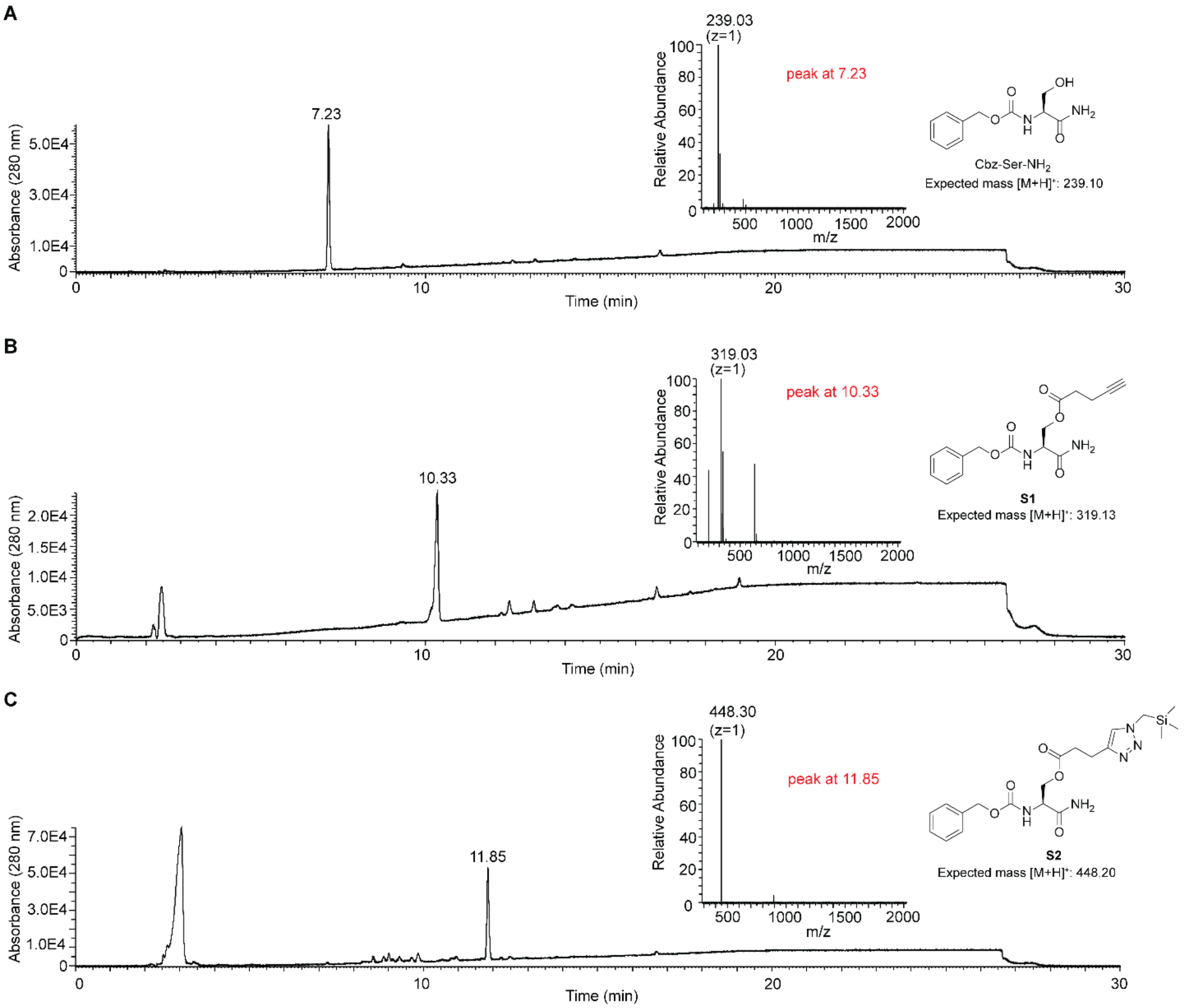

Figure S18. Liquid-chromatography mass spectrometry (LC-MS) analysis of Cbz-Ser-NH_2 and synthesized derivatives of Cbz-Ser-NH_2. (A) LC-MS analysis of commercial Cbz-Ser-NH_2 (Chem-Impex 12325). (B) LC-MS analysis of **S1** purified with methods described in the *synthesis of **S1*** section. (C) LC-MS analysis of **S2** purified with methods described in the *synthesis of **S2*** section.

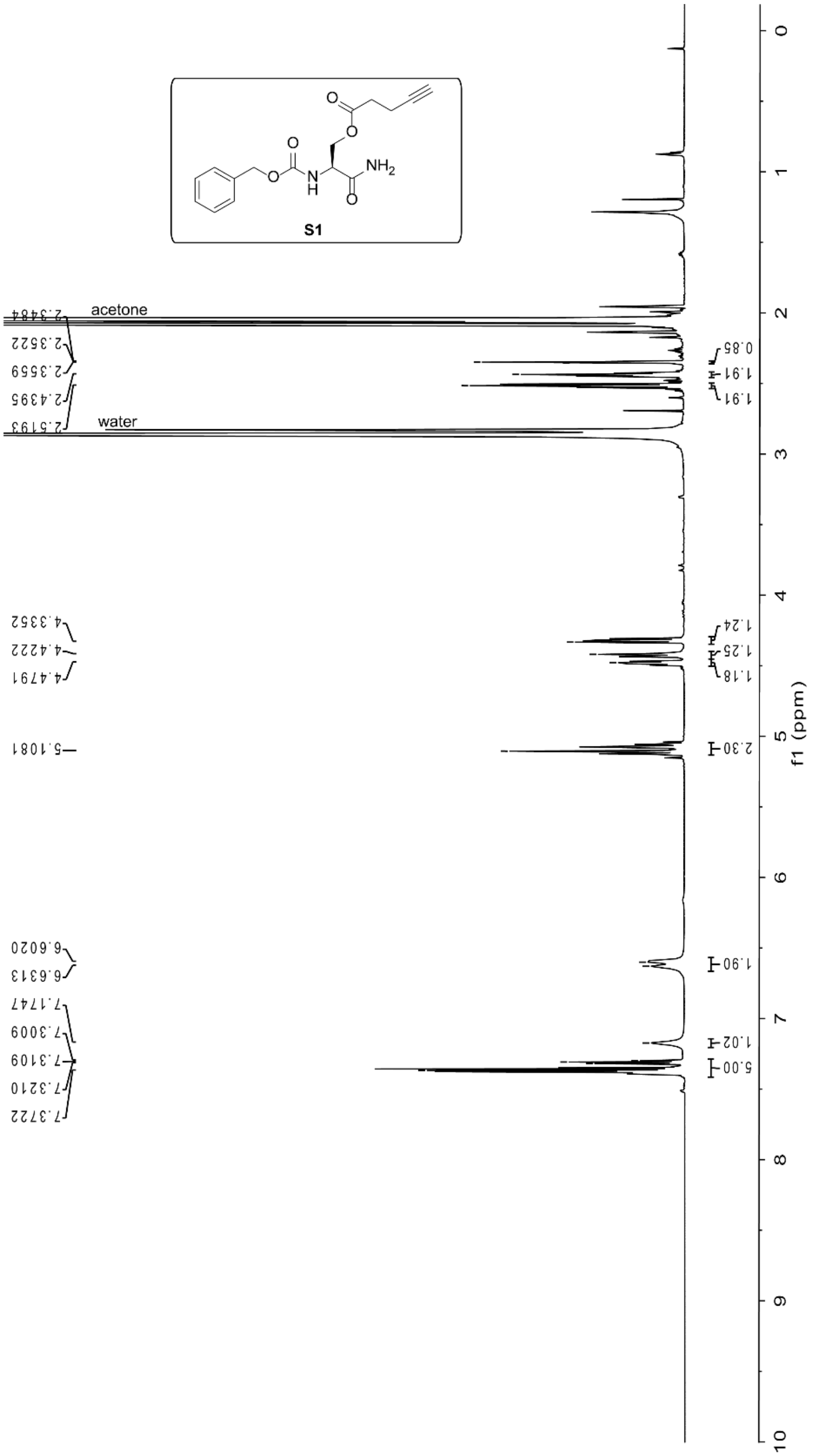

Figure S19. ^1H NMR spectrum of **S1** in acetone-d_6.

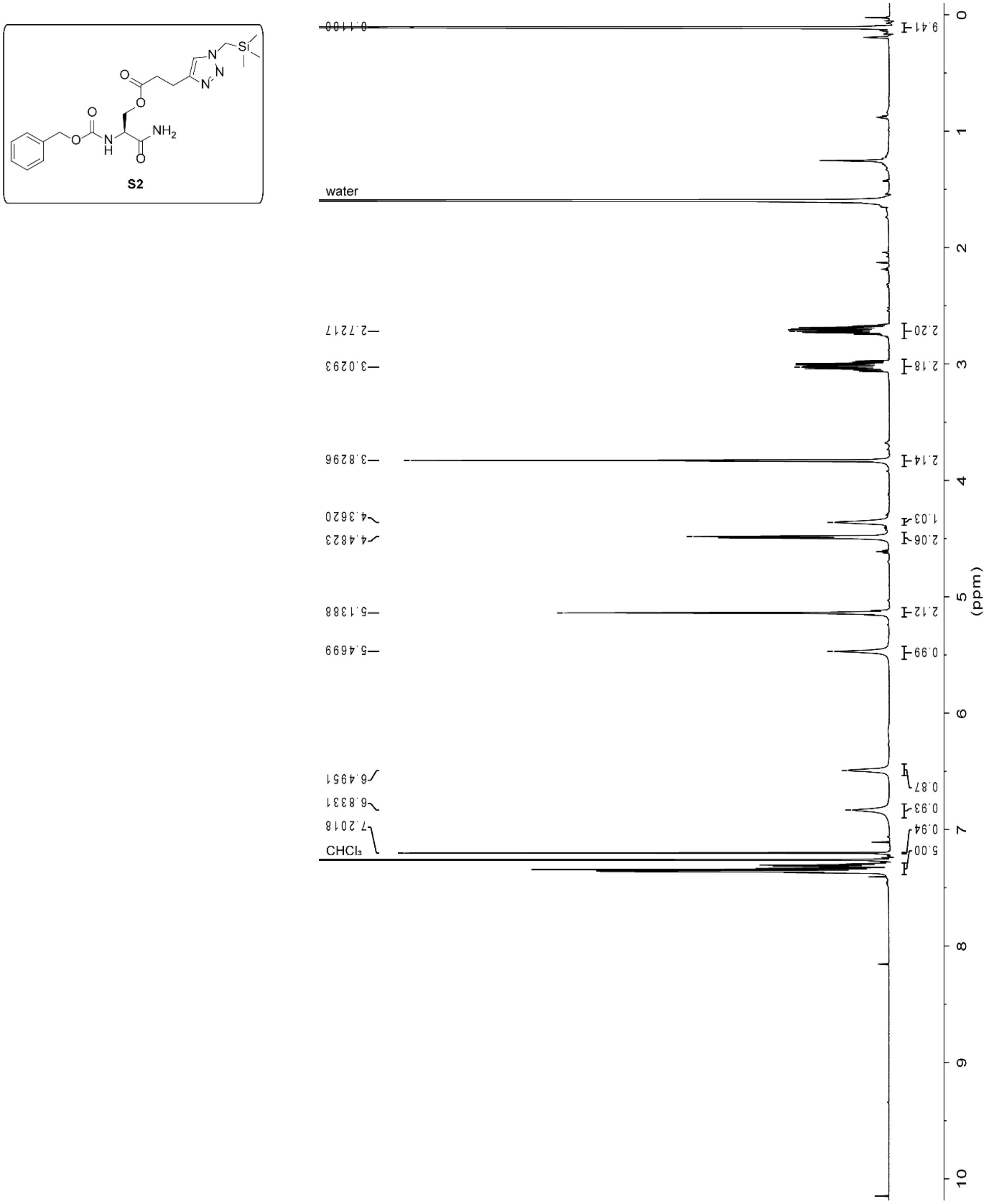

Figure S20. ^{1}H NMR spectrum of **S2** in $CDCl_3$.

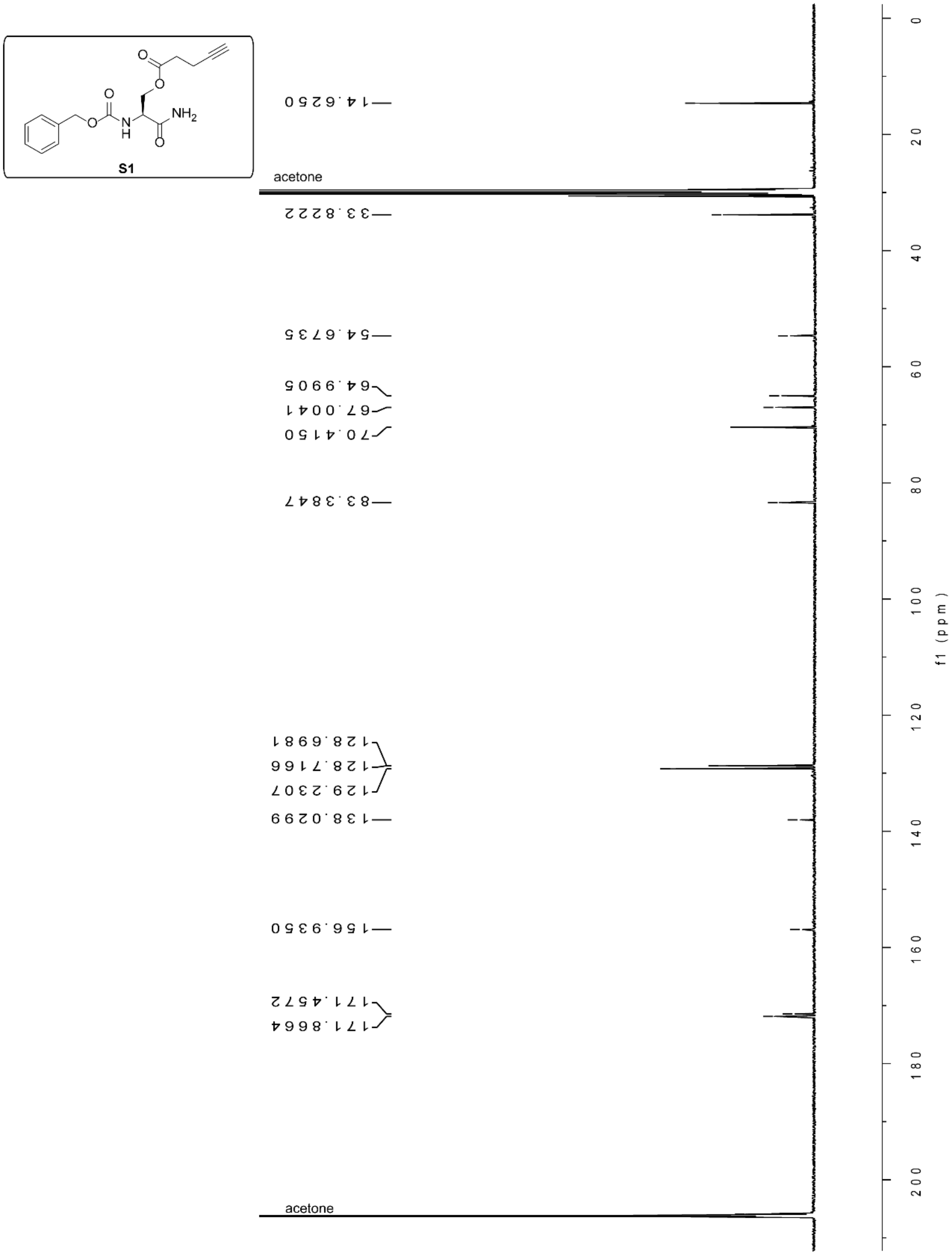

Figure S21. ^{13}C NMR spectrum of **S1** in acetone-d_6.

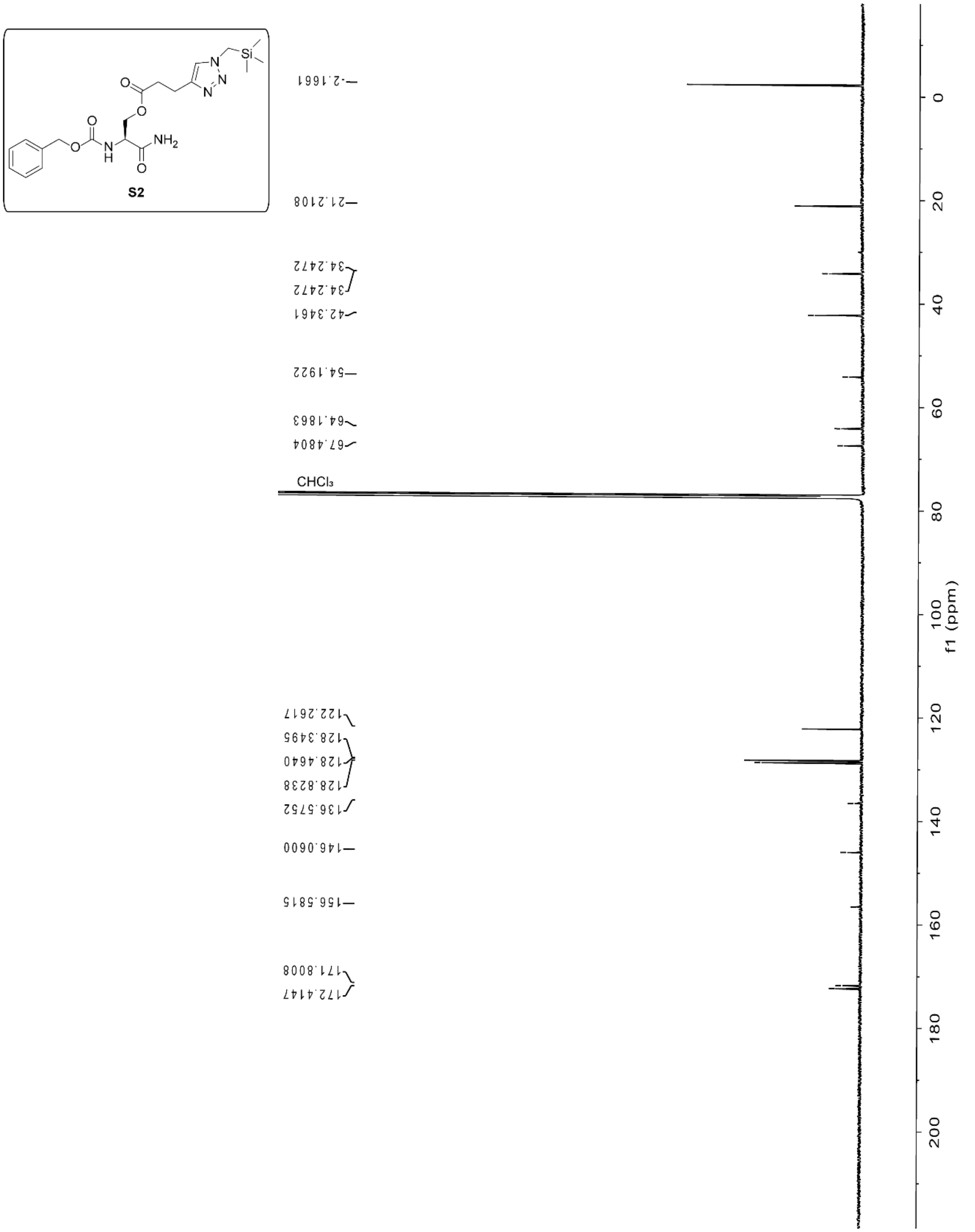

Figure S22. ^{13}C NMR spectrum of **S2** in $CDCl_3$.

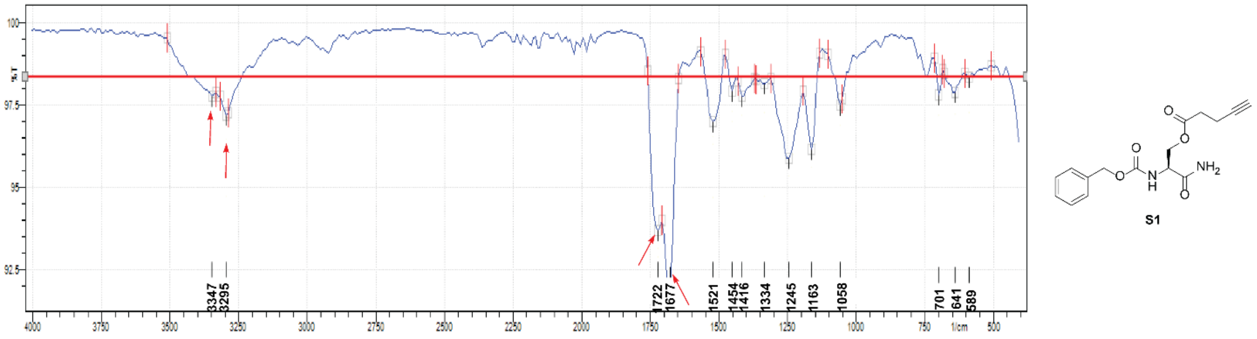

Figure S23. FT-IR spectrum of **S1**.

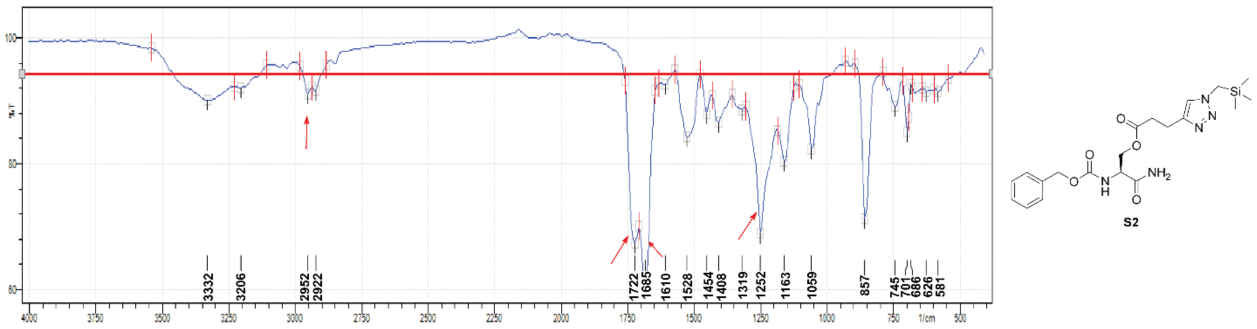

Figure S24. FT-IR spectrum of **S2**.

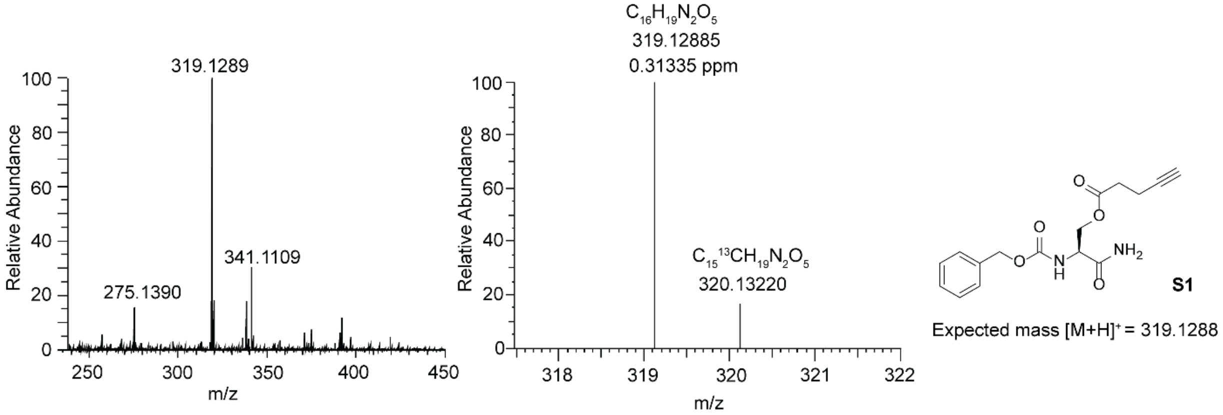

Figure S25. HRMS-ESI spectra of **S1**.

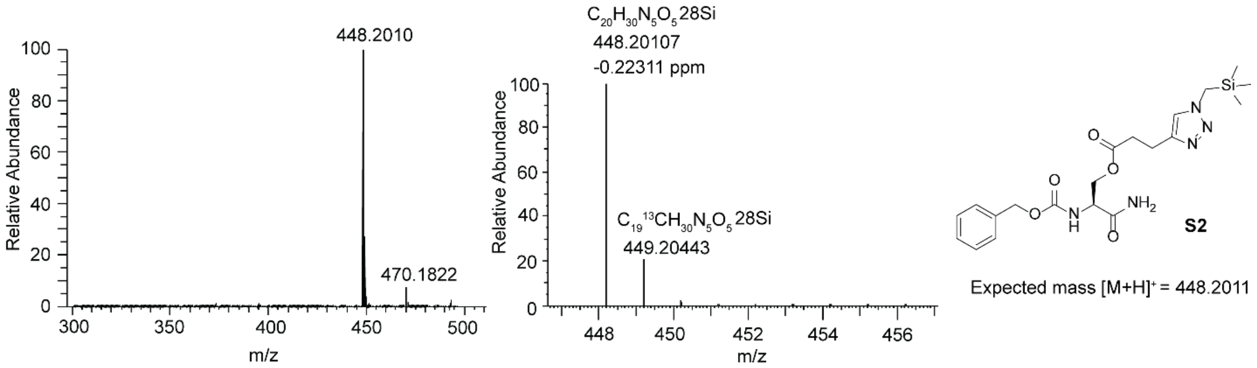

Figure S26. HRMS-ESI spectra of **S2**.

Acid catalyst screening

Acid catalyst screening studies with peptides and proteins shown below revealed optimal catalysts for the acid-catalyzed bioconjugation method. A catalyst screening with a model peptide was performed with acetic acid as a solvent and somatostatin-14 as a model substrate. H_2SO_4, $Bi(OTf)_3$, $In(OTf)_3$, $Sc(OTf)_3$, and sulfonic acid ionic liquid showed very small or no peptide peaks on UV chromatograms in LC-MS analyses, which might be due to the degradation of peptide substrates. In contrast, aq. HCl, TFA, $Dy(OTf)_3$, and the bisulfate ionic liquid showed decent reaction conversions without apparent loss of peptide peaks on UV chromatograms. A catalyst screening with protein was performed with acetic acid as a solvent and α-chymotrypsinogen as a model substrate. The result showed a similar trend to the screening with the peptide, where some catalysts showed no peak on the UV chromatograms on LC-MS analysis whereas aq. HCl, TFA, and $Dy(OTf)_3$ gave decent modifications. We decided to mainly use TFA as an acid catalyst in this study because of the reported peptide compatibilities,[1,2] and low boiling point which makes removing the catalyst after the reaction easier. It should be noted that TFA adduct (+96 Da) was not observed when TFA (6% v/v, 0.79 M) was used as a catalyst.

Table S1. Summary of catalyst screening for protein modification. Typical modification conditions: Somatostatin-14 (1.0 mM, sequence: AGCKNFFWKTFTSC), catalyst (given concentration) in acetic acid for 24 h at 50 °C. The conversions were calculated by dividing the product peak area by the sum of the product peak area and starting material peak area of UV chromatograms (liquid chromatography). Somatostatin-14: cyclic somatostatin-14 with a disulfide bond between the two cysteine residues. OTf: triflate, bisulfate ionic liquid: 1-butyl-3-methylimidazolium hydrogen sulfate, TFA: trifluoroacetic acid, sulfonic acid ionic liquid: 1-methyl-3-(4-sulfobutyl) imidazolium hydrogen sulfate. *Peaks of peptides have low S/N ratios on UV chromatograms.

catalyst	conversion (%)
no catalyst	13
HCl 100 mM	63
H_2SO_4 100 mM	<5%
TFA 100 mM	23
TFA 6% (v/v), 0.78 M	61
$Bi(OTf)_3$ 100 mM	56*
$Cp_2Zr(OTf)_2$ 100 mM	<5%
$Dy(OTf)_3$ 100 mM	64
$In(OTf)_3$ 100 mM	77*
$Sc(OTf)_3$ 100 mM	96*
Bisulfate ionic liquid 100 mM	77
Sulfonic acid ionic liquid 100 mM	74*

Somatostatin-14
AGCKNFFWKTFTSC

no catalyst (column temperature: 60 °C)

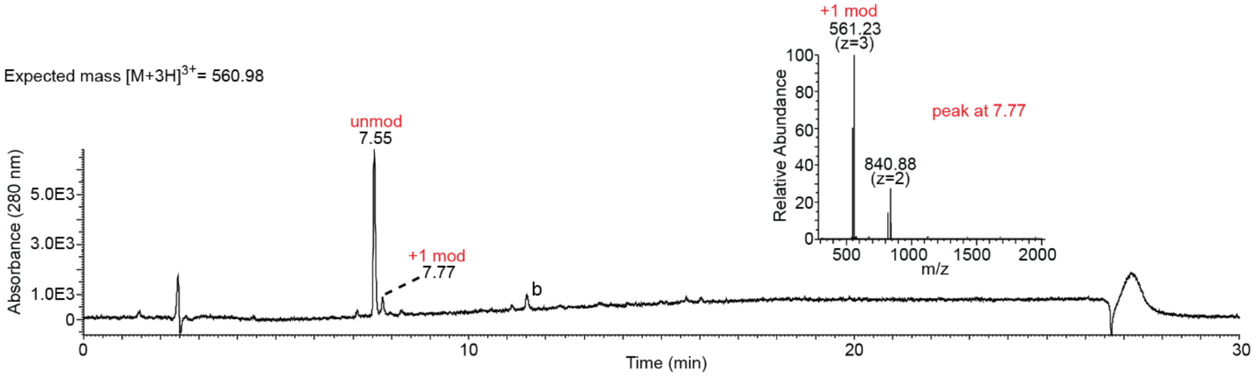

Figure S27. Liquid chromatography-mass spectrometry (LC-MS) analysis of reaction mixtures of modification of somatostatin-14. Typical modification conditions: somatostatin-14 (1 mM final concn from 100 mM stock solution in acetic acid) in acetic acid for 24 h at 50 °C. Label b in the chromatogram indicates a peak observed even in a blank sample shown in the *Liquid-chromatography mass spectrometry (LC-MS) analysis of acetic acid treated with TFA (column oven temperature: 60 °C)* figure. b=11.5 min when LC column oven temperature is 60 °C.

HCl 100 mM (column temperature: 60 °C)

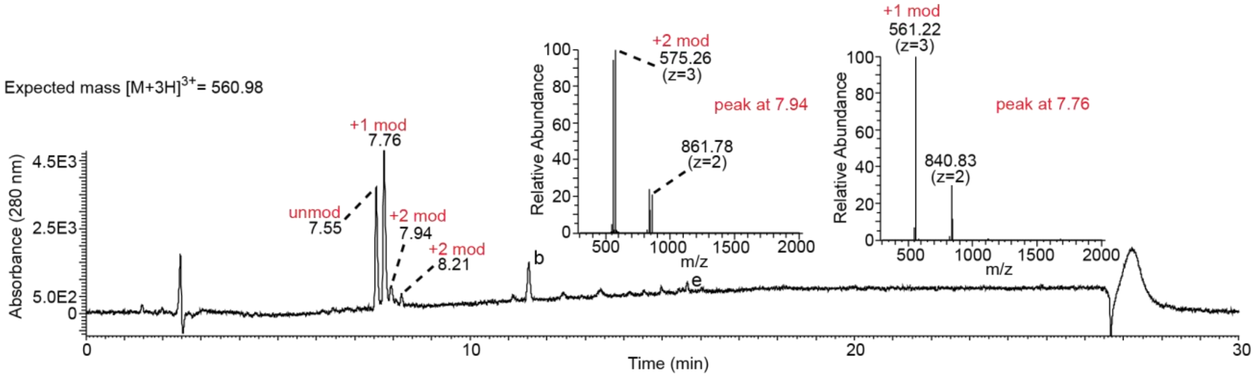

Figure S28. Liquid chromatography-mass spectrometry (LC-MS) analysis of reaction mixtures of modification of somatostatin-14. Typical modification conditions: somatostatin-14 (1 mM final concn from 100 mM stock solution in acetic acid), hydrochloric acid (100 mM final concn from 200 mM stock solution in acetic acid) in acetic acid for 24 h at 50 °C. Labels b and e in the chromatograms indicate peaks observed even in a blank sample shown in the *Liquid-chromatography mass spectrometry (LC-MS) analysis of acetic acid treated with TFA (column oven temperature: 60 °C)* figure. b=11.5 min, e=16.0 min when LC column oven temperature is 60 °C.

H_2SO_4 (column temperature: 60 °C)

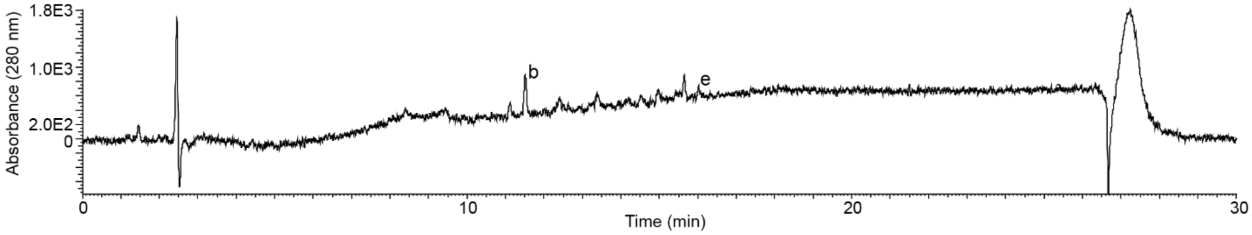

Figure S29. Liquid chromatography-mass spectrometry (LC-MS) analysis of reaction mixtures of modification of somatostatin-14. Typical modification conditions: somatostatin-14 (1 mM final concn from 100 mM stock solution in acetic acid), sulfuric acid (100 mM final concn from 200 mM stock solution in acetic acid) in acetic acid for 24 h at 50 °C. Labels b and e in the chromatograms indicate peaks observed even in a blank sample shown in the *Liquid-chromatography mass spectrometry (LC-MS) analysis of acetic acid treated with TFA (column oven temperature: 60 °C)* figure. b=11.5 min, e=16.0 min when LC column oven temperature is 60 °C.

TFA 100 mM (column temperature: 60 °C)

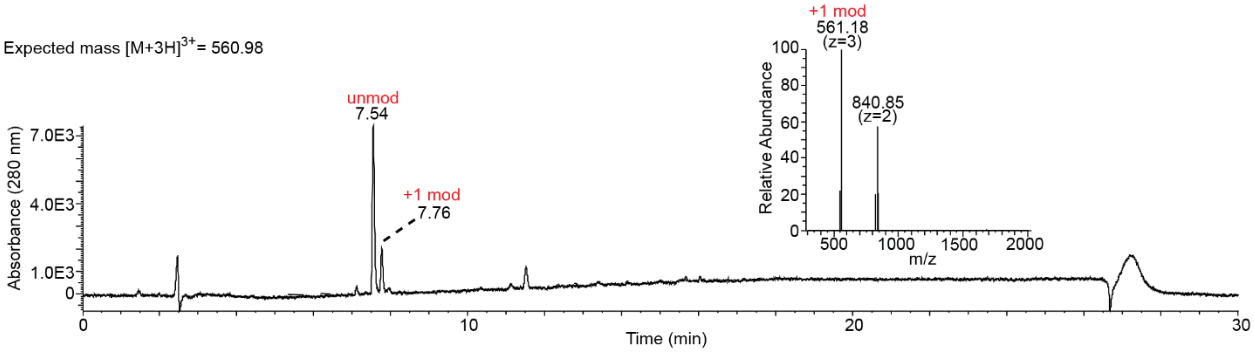

Figure S30. Liquid chromatography-mass spectrometry (LC-MS) analysis of reaction mixtures of modification of somatostatin-14. Typical modification conditions: somatostatin-14 (1 mM final concn from 100 mM stock solution in acetic acid), trifluoroacetic acid (100 mM final concn from 200 mM stock solution in acetic acid) in acetic acid for 24 h at 50 °C.

TFA 6% (column temperature: 60 °C)

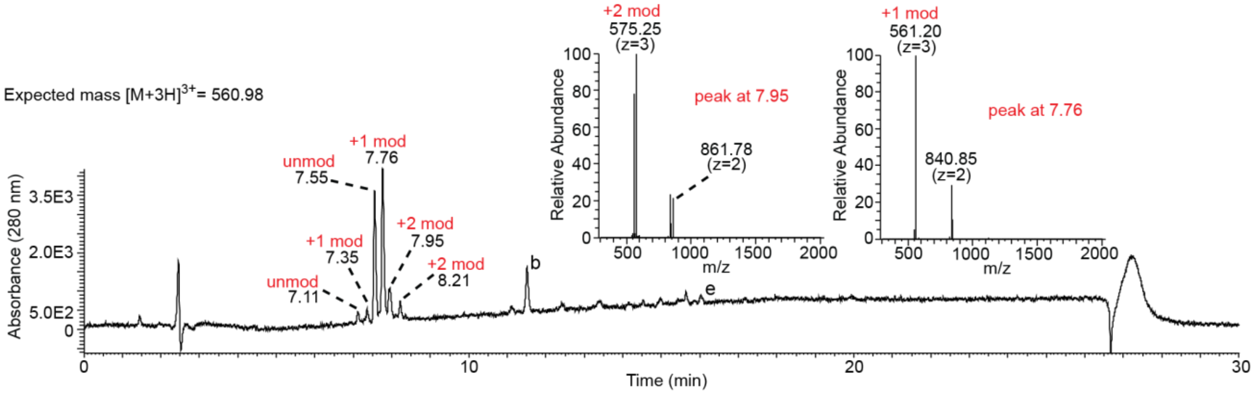

Figure S31. Liquid chromatography-mass spectrometry (LC-MS) analysis of reaction mixtures of modification of somatostatin-14. Typical modification conditions: somatostatin-14 (1 mM final concn from 100 mM stock solution in acetic acid), trifluoroacetic acid (6% v/v, 0.78 M final concn from 12% v/v stock solution in acetic acid) in acetic acid for 24 h at 50 °C. Labels b and e in the chromatograms indicate peaks observed even in a blank sample shown in the *Liquid-chromatography mass spectrometry (LC-MS) analysis of acetic acid treated with TFA (column oven temperature: 60 °C)* figure. b=11.5 min, e=16.0 min when LC column oven temperature is 60 °C.

$Bi(OTf)_3$ 100 mM (column temperature: 60 °C)

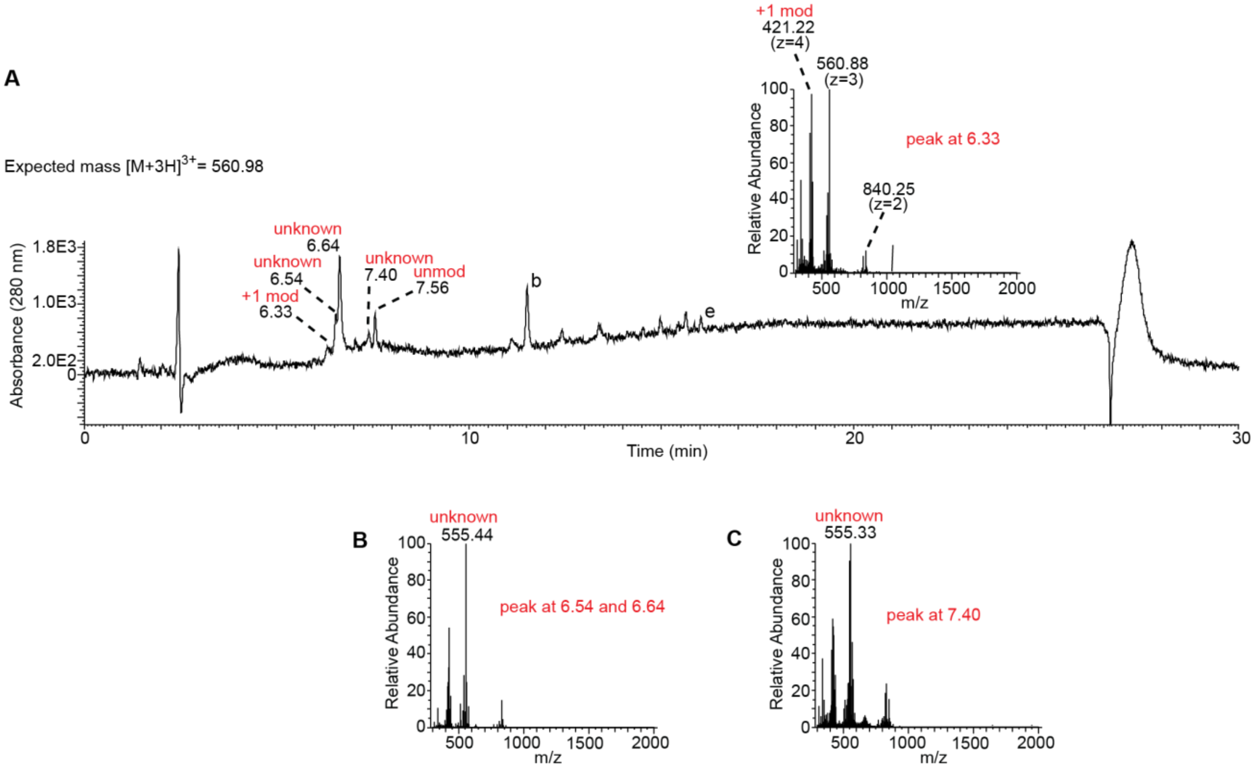

Figure S32. Liquid chromatography-mass spectrometry (LC-MS) analysis of reaction mixtures of modification of somatostatin-14. (A) LC chromatogram and MS spectra for the reaction with the catalyst. (B) MS spectrum for peaks at 6.54 min and 6.64 min in the UV chromatogram. (C) MS spectrum for a peak at 7.40 min in the UV chromatogram. Typical modification conditions: somatostatin-14 (1 mM final concn from 100 mM stock solution in acetic acid), bismuth (III) triflate (100 mM final concn from 200 mM stock solution in acetic acid) in acetic acid for 24 h at 50 °C. Labels b and e in the chromatograms indicate peaks observed even in a blank sample shown in the *Liquid-chromatography mass spectrometry (LC-MS) analysis of acetic acid treated with TFA (column oven temperature: 60 °C)* figure. b=11.5 min, e=16.0 min when LC column oven temperature is 60 °C.

$Cp_2Zr(OTf)_2$ 100 mM (column temperature: 60 °C)

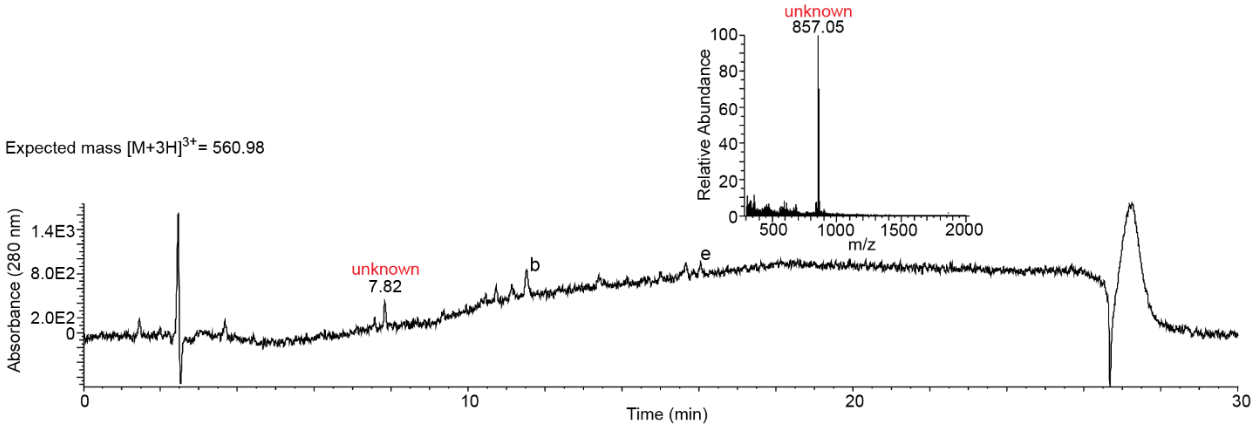

Figure S33. Liquid chromatography-mass spectrometry (LC-MS) analysis of reaction mixtures of modification of somatostatin-14. Typical modification conditions: somatostatin-14 (1 mM final concn from 100 mM stock solution in acetic acid), $Cp_2Zr(OTf)_2$ (100 mM final concn from 200 mM stock solution in acetic acid) in acetic acid for 24 h at 50 °C. Labels b and e in the chromatograms indicate peaks observed even in a blank sample shown in the *Liquid-chromatography mass spectrometry (LC-MS) analysis of acetic acid treated with TFA (column oven temperature: 60 °C)* figure. b=11.5 min, e=16.0 min when LC column oven temperature is 60 °C.

$Dy(OTf)_3$ 100 mM (column temperature: 60 °C)

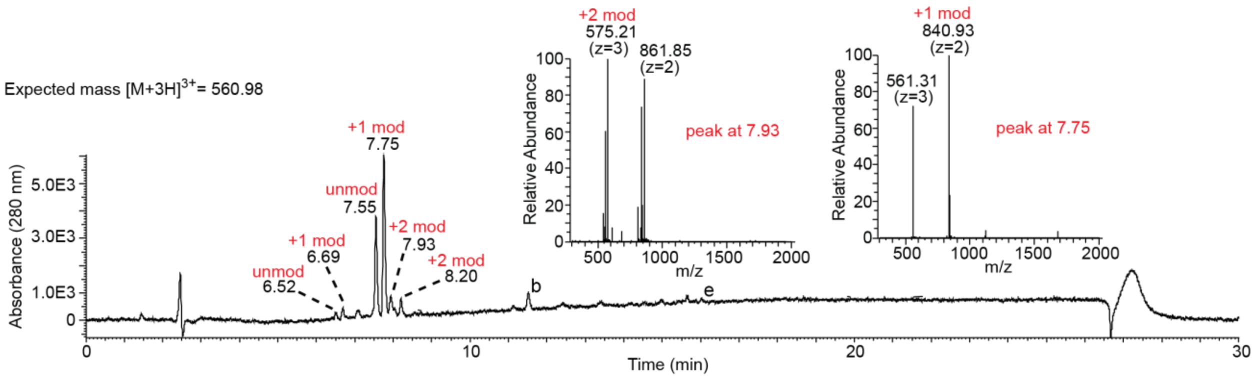

Figure S34. Liquid chromatography-mass spectrometry (LC-MS) analysis of reaction mixtures of modification of somatostatin-14. Typical modification conditions: somatostatin-14 (1 mM final concn from 100 mM stock solution in acetic acid), dysprosium (III) triflate (100 mM final concn from 200 mM stock solution in acetic acid) in acetic acid for 24 h at 50 °C. Labels b and e in the chromatograms indicate peaks observed even in a blank sample shown in the *Liquid-chromatography mass spectrometry (LC-MS) analysis of acetic acid treated with TFA (column oven temperature: 60 °C)* figure. b=11.5 min, e=16.0 min when LC column oven temperature is 60 °C.

$In(OTf)_3$ 100 mM (column temperature: 60 °C)

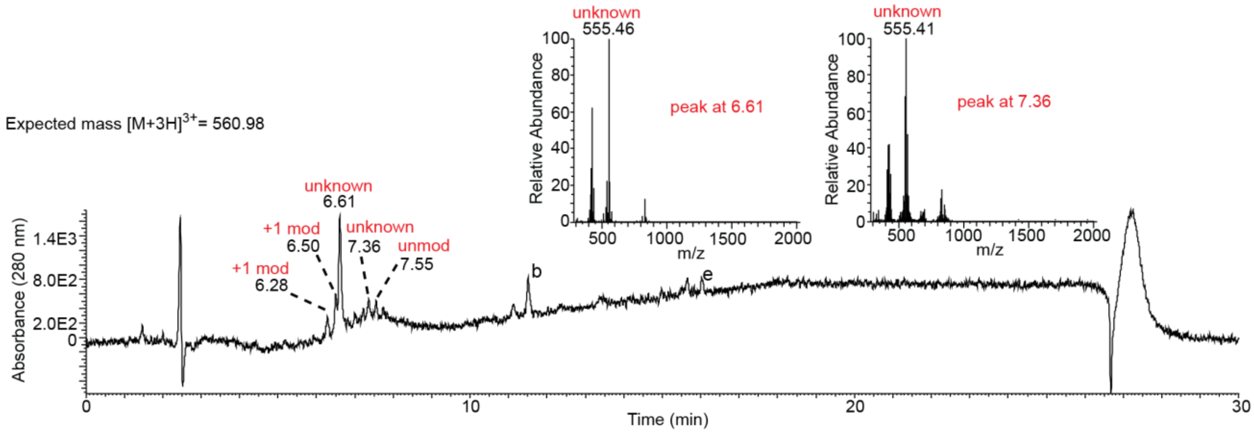

Figure S35. Liquid chromatography-mass spectrometry (LC-MS) analysis of reaction mixtures of modification of somatostatin-14. Typical modification conditions: somatostatin-14 (1 mM final concn from 100 mM stock solution in acetic acid), indium (III) triflate (100 mM final concn from 200 mM stock solution in acetic acid) in acetic acid for 24 h at 50 °C. Labels b and e in the chromatograms indicate peaks observed even in a blank sample shown in the *Liquid-chromatography mass spectrometry (LC-MS) analysis of acetic acid treated with TFA (column oven temperature: 60 °C)* figure. b=11.5 min, e=16.0 min when LC column oven temperature is 60 °C.

Sc(OTf)$_3$ 100 mM (column temperature: 60 °C)

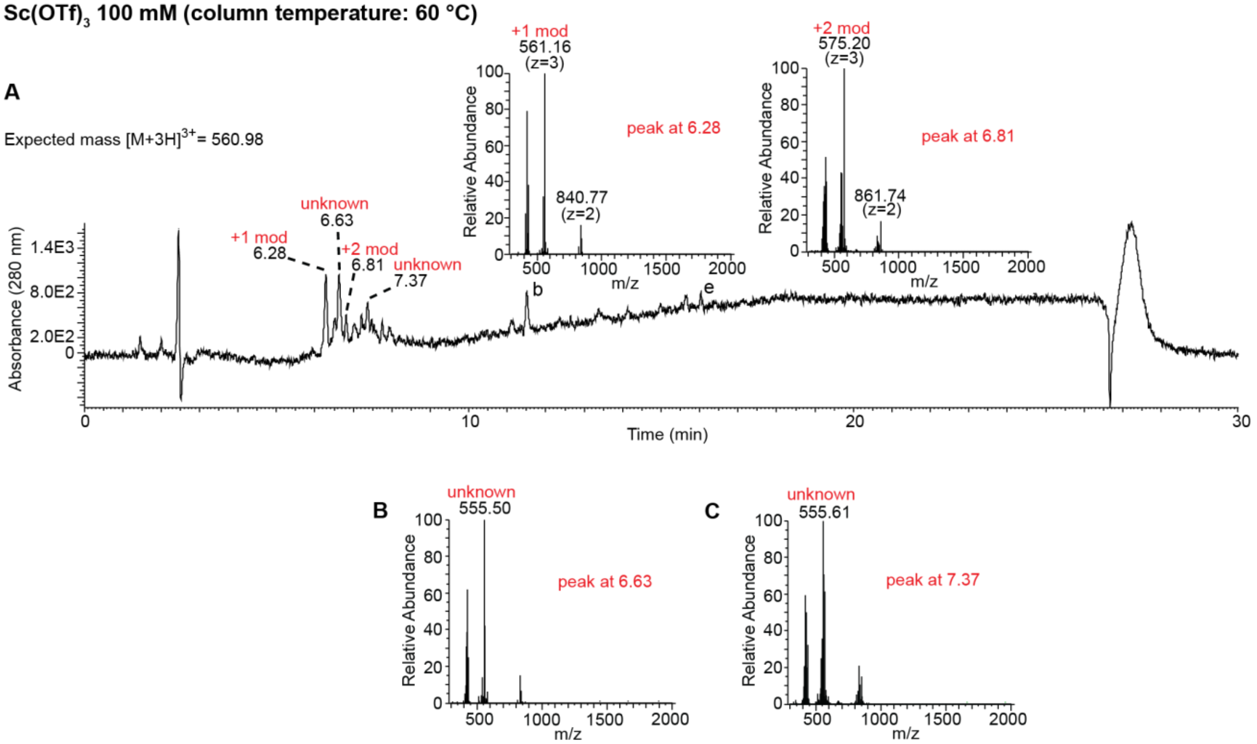

Figure S36. Liquid chromatography-mass spectrometry (LC-MS) analysis of reaction mixtures of modification of somatostatin-14. (A) LC chromatogram and MS spectra for the reaction with the catalyst. (B) MS spectrum for a peak at 6.63 min in the UV chromatogram. (C) MS spectrum for a peak at 7.37 min in the UV chromatogram. Typical modification conditions: somatostatin-14 (1 mM final concn from 100 mM stock solution in acetic acid), scandium (III) triflate (100 mM final concn from 200 mM stock solution in acetic acid) in acetic acid for 24 h at 50 °C. Labels b and e in the chromatograms indicate peaks observed even in a blank sample shown in the *Liquid-chromatography mass spectrometry (LC-MS) analysis of acetic acid treated with TFA (column oven temperature: 60 °C)* figure. b=11.5 min, e=16.0 min when LC column oven temperature is 60 °C.

Bisulfate ionic liquid 100 mM (column temperature: 60 °C)

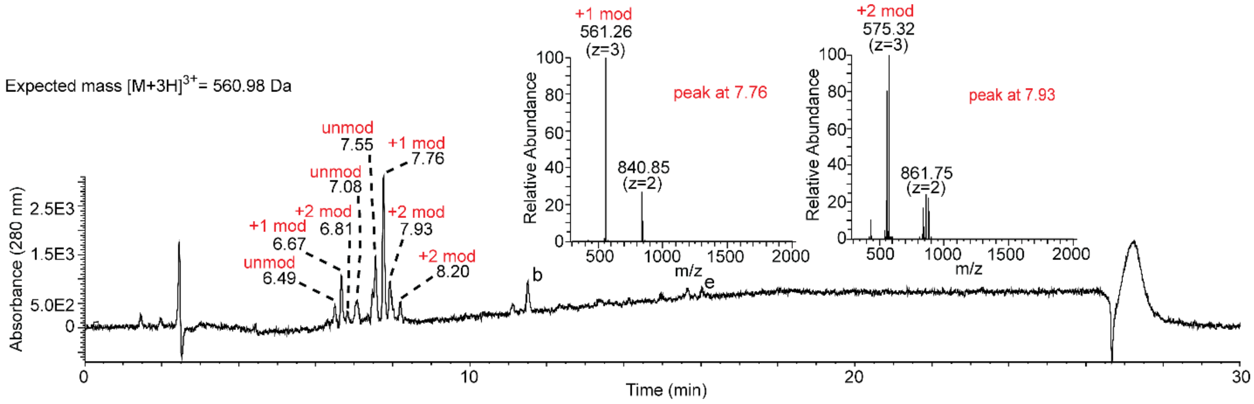

Figure S37. Liquid chromatography-mass spectrometry (LC-MS) analysis of reaction mixtures of modification of somatostatin-14. Typical modification conditions: somatostatin-14 (1 mM final concn from 100 mM stock solution in acetic acid), bisulfate ionic liquid (100 mM final concentration from neat liquid) in acetic acid for 24 h at 50 °C. Labels b and e in the chromatograms indicate peaks observed even in a blank sample shown in the *Liquid-chromatography mass spectrometry (LC-MS) analysis of acetic acid treated with TFA (column oven temperature: 60 °C)* figure. b=11.5 min, e=16.0 min when LC column oven temperature is 60 °C.

Sulfonic acid ionic liquid 100 mM (column temperature: 60 °C)

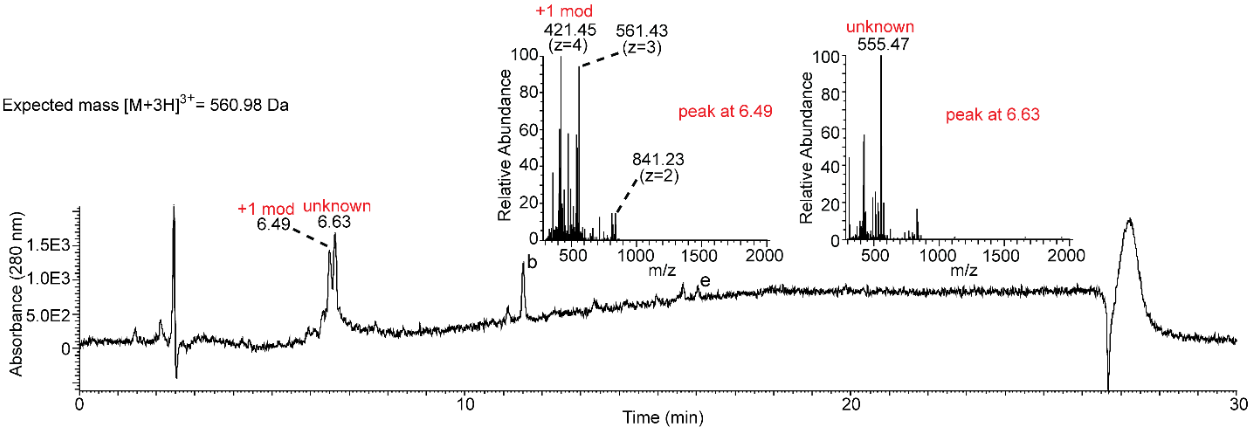

Figure S38. Liquid chromatography-mass spectrometry (LC-MS) analysis of reaction mixtures of modification of somatostatin-14. Typical modification conditions: somatostatin-14 (1 mM final concn from 100 mM stock solution in acetic acid), sulfonic acid ionic liquid (100 mM final concn from neat liquid) in acetic acid for 24 h at 50 °C. Labels b and e in the chromatograms indicate peaks observed even in a blank sample shown in the *Liquid-chromatography mass spectrometry (LC-MS) analysis of acetic acid treated with TFA (column oven temperature: 60 °C)* figure. b=11.5 min, e=16.0 min when LC column oven temperature is 60 °C.

Table S2. Summary of catalyst screening for protein modification.

catalyst	conversion (%)	average (mod.)
No catalyst	52	0.6
HCl (10 mM)	96	2.9
TFA (10 mM)	63	1.0
TFA (1%, 0.13 M)	97	3.2
$Dy(OTf)_3$ (10 mM)	100	4.8

The conversions were calculated by dividing the product peak intensity by the sum of the product peak intensity and the starting material peak intensity of the deconvoluted ESI mass spectra shown in the *ESI mass spectrometry analysis of α-chymotrypsinogen A modified in acetic acid with various catalysts* figure. H_2SO_4 (10 mM final concn from 20 mM stock solution in acetic acid), $Bi(OTf)_3$ (10 mM final concn from 20 mM stock solution in acetic acid), $Cp_2Zr(OTf)_2$ (10 mM final concn from 20 mM stock solution in acetic acid), $In(OTf)_3$ (10 mM final concn from 20 mM stock solution in acetic acid), $Sc(OTf)_3$ (10 mM final concn from 20 mM stock solution in acetic acid), 1-methyl-3-(4-sulfobutyl)imidazolium hydrogen sulfate (10 mM final concn from 1 M stock solution in EMIM BF_4), and 1-butyl-3-methylimidazolium hydrogen sulfate (10 mM final concn from 1 M stock solution in EMIM BF_4) were also screened as catalysts but no significant peak was observed in UV and mass chromatograms for the LCMS analysis.

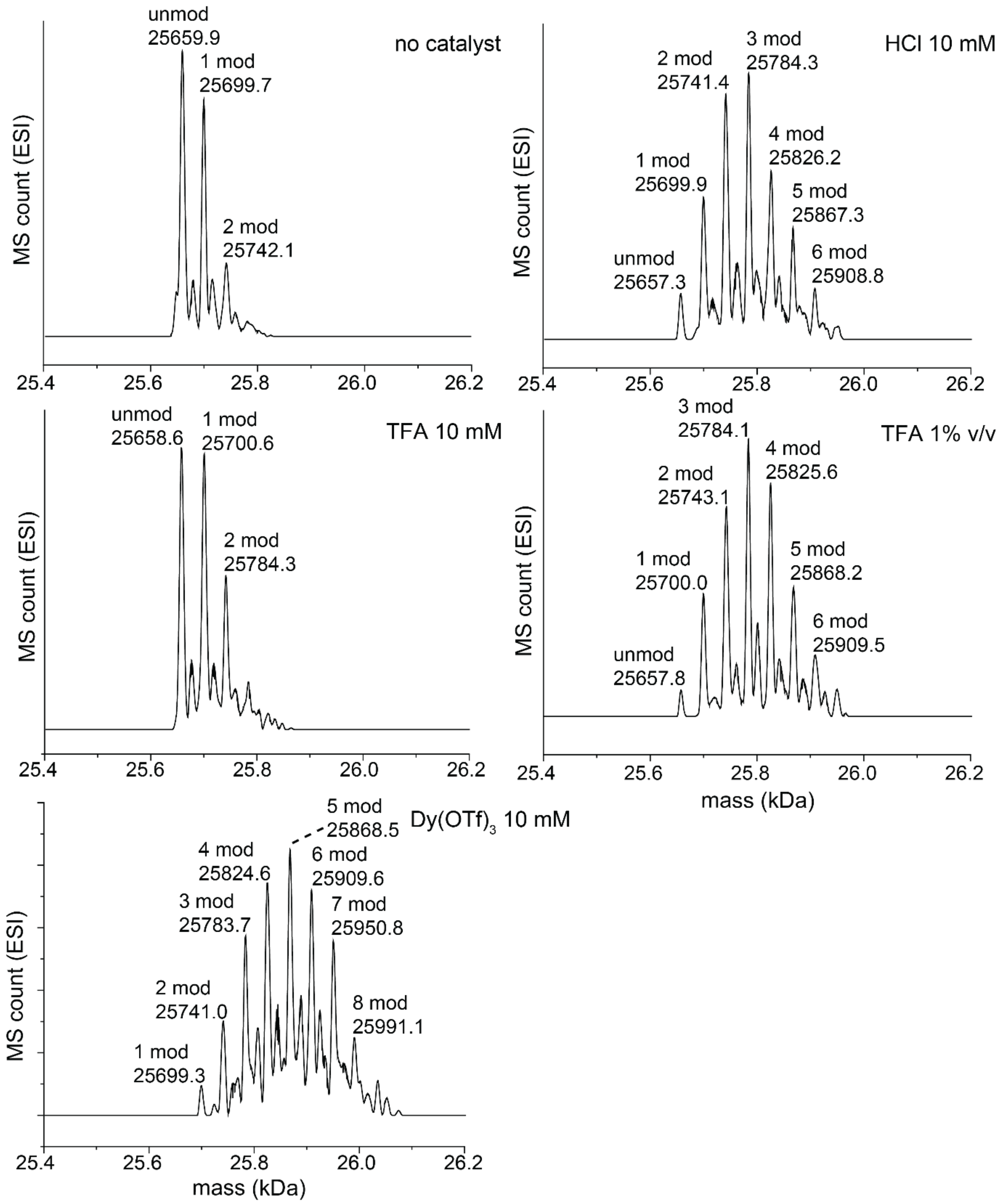

Figure S39. ESI mass spectrometry analysis of α-chymotrypsinogen A modified in acetic acid with various catalysts. Summary of the modification efficiency by each catalyst is shown in *summary of catalyst screening for protein modification* table. Reaction conditions: α-chymotrypsinogen A (0.072 mM final concn from 7.2 mM stock solution in 50 mM MES buffer pH 7.4) and catalysts (see below for the concentration) were incubated in acetic acid for 24 h at 37 °C. HCl and $Dy(OTf)_3$: 10 mM final concn from 20 mM stock solution in acetic acid. TFA: 10 mM final concn from 20 mM stock solution in acetic acid or 1% v/v, 0.13 M final concn from neat liquid.

The peptide scope investigation and tandem mass spectrometry analysis: The peptide scope investigation results shown below indicated chemoselectivity toward the alkyl hydroxyl groups. The LC-MS analysis showed the acylation products with just serine- and threonine-containing peptides, but not with peptides that contain tyrosine, lysine, arginine, and tryptophan. With TFA (6% v/v, 0.80 M) as a catalyst, serine-containing peptides showed higher conversions than peptides that contain threonine without serine, implying serine-selectivity of the acid-catalyzed acylation method over threonine at the peptide level. The amino acid selectivity trend was not compromised even with higher TFA loading (12% v/v, 1.6 M), and up to 85% conversion was observed for a serine-containing peptide. Tandem mass spectrometry (MS/MS) suggested reactions on serine or threonine.

Table S3. Scope of labeling of peptide substrates.

name	sequence	TFA 6% (v/v) conversion %	TFA 12% (v/v) conversion %
Allatostatin I	H-APSGAQRLYGFGL-NH_2	66	82
TRAP-14	H-SFLLRNPNDKYEPF-OH	40	43
LHRH	Glp-HWSYGLRPG-NH_2	64	74
Somatostatin-14	H-AGCKNFFWKTFTSC-OH	59	78
GHRF 1-29	H-HADAIFTSSYRRILGQLYARKLLHEIMNR-NH_2	54	100
PMAP-23	H-RIIDLLWRVRRPQKPKFVTVWVR-OH	26	50
PANP-004	H-RTRPLWVRME-OH	35	55
BAMP-001	H-YGGFMRRVGRPE-OH	<5%	<5%
IDR-1018	H-VRLIVAVRIWRR-NH_2	<5%	<5%
Osteocalcin (37-49)	H-GFQEAYRRFYGPV-OH	<5%	<5%
Dynorphin A	H-YGGFLRRIRPKLK-OH	<5%	<5%

Typical modification conditions: peptide (0.05-1.0 mM), trifluoroacetic acid (6% v/v, 0.78 M or 12% v/v, 1.6 M) in acetic acid for 24 h at 50 °C. The conversions were calculated by dividing the product peak area by the sum of the product peak area and starting material peak area of UV chromatograms (liquid chromatography). TRAP-14: Thrombin receptor agonist peptide-14. LHRH: Luteinizing hormone-releasing hormone human acetate salt. Somatostatin-14: cyclic somatostatin-14 with a disulfide bond between the two cysteine residues. GHRF 1-29: Growth hormone releasing factor 1-29. PMAP-23: Porcine myeloid antibacterial peptide-23. PANP-004: BDC2.5 mimotope peptide 1040-63. BAMP-001: Precursor of bovine adrenomedullary Met-enkephalin. IDR-1018: Innate defense regulator 1018.

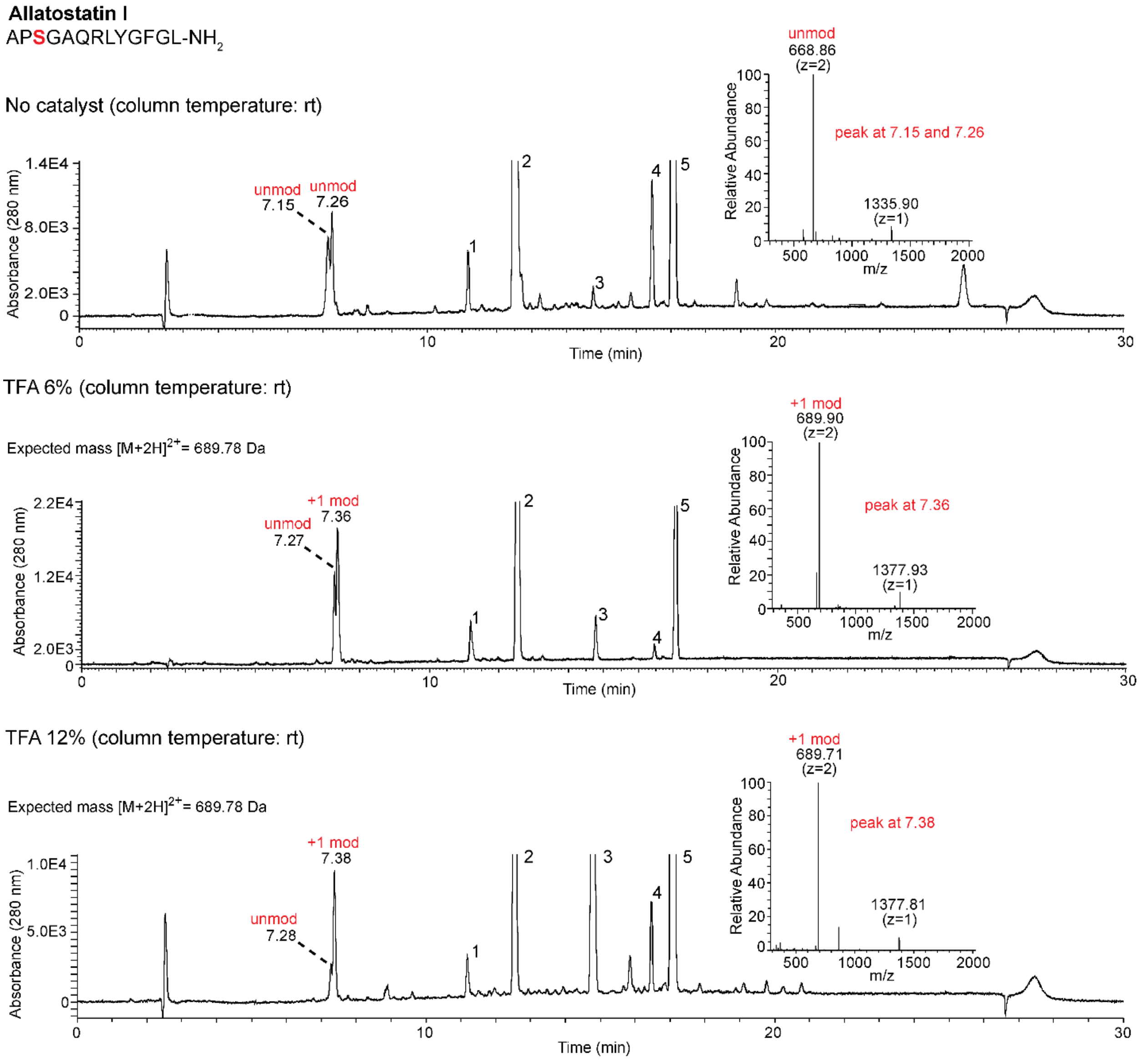

Figure S40. Liquid chromatography-mass spectrometry (LC-MS) analysis of reaction mixtures of modification of allatostatin I. Reaction conditions: allatostatin I (0.05 mM final concn from 5 mM stock solution in H_2O), trifluoroacetic acid (0–12% v/v, 0–1.6 M final concn from 0%–24% v/v stock solution in acetic acid) in acetic acid for 24 h at 50 °C. Labels 1, 2, 3, 4, and 5 in the chromatograms indicate peaks observed even in a blank sample shown in the *Liquid-chromatography mass spectrometry (LC-MS) analysis of acetic acid treated with TFA (column oven temperature: rt)* figure. 1=11.2 min, 2=12.5 min, 3=14.8 min, 4=16.5 min, 5=17.1 min when LC column oven temperature is at room temperature (rt).

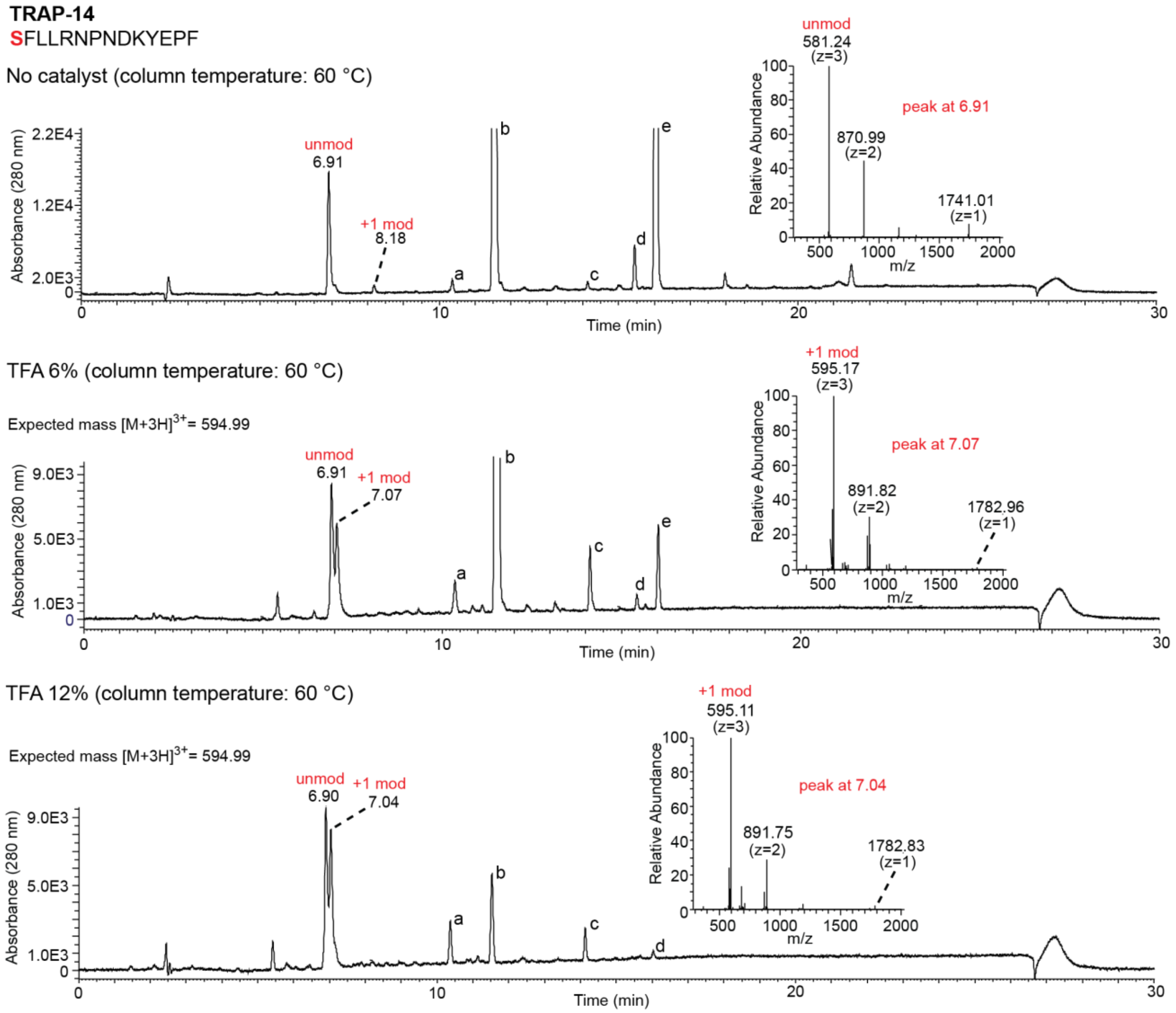

Figure S41. Liquid chromatography-mass spectrometry (LC-MS) analysis of reaction mixtures of modification of TRAP-14. Reaction conditions: TRAP-14 (0.05 mM final concn from 5 mM stock solution in H_2O), trifluoroacetic acid (0–12% v/v, 0–1.6 M final concn from 0%–24% v/v stock solution in acetic acid) in acetic acid for 24 h at 50 °C. Labels a, b, c, d, and e in the chromatograms indicate peaks observed even in a blank sample shown in the *Liquid-chromatography mass spectrometry (LC-MS) analysis of acetic acid treated with TFA (column oven temperature: 60 °C)* figure. a=10.4 min, b=11.5 min, c=14.1 min, d=15.4 min, e=16.0 min when LC column oven temperature is 60 °C.

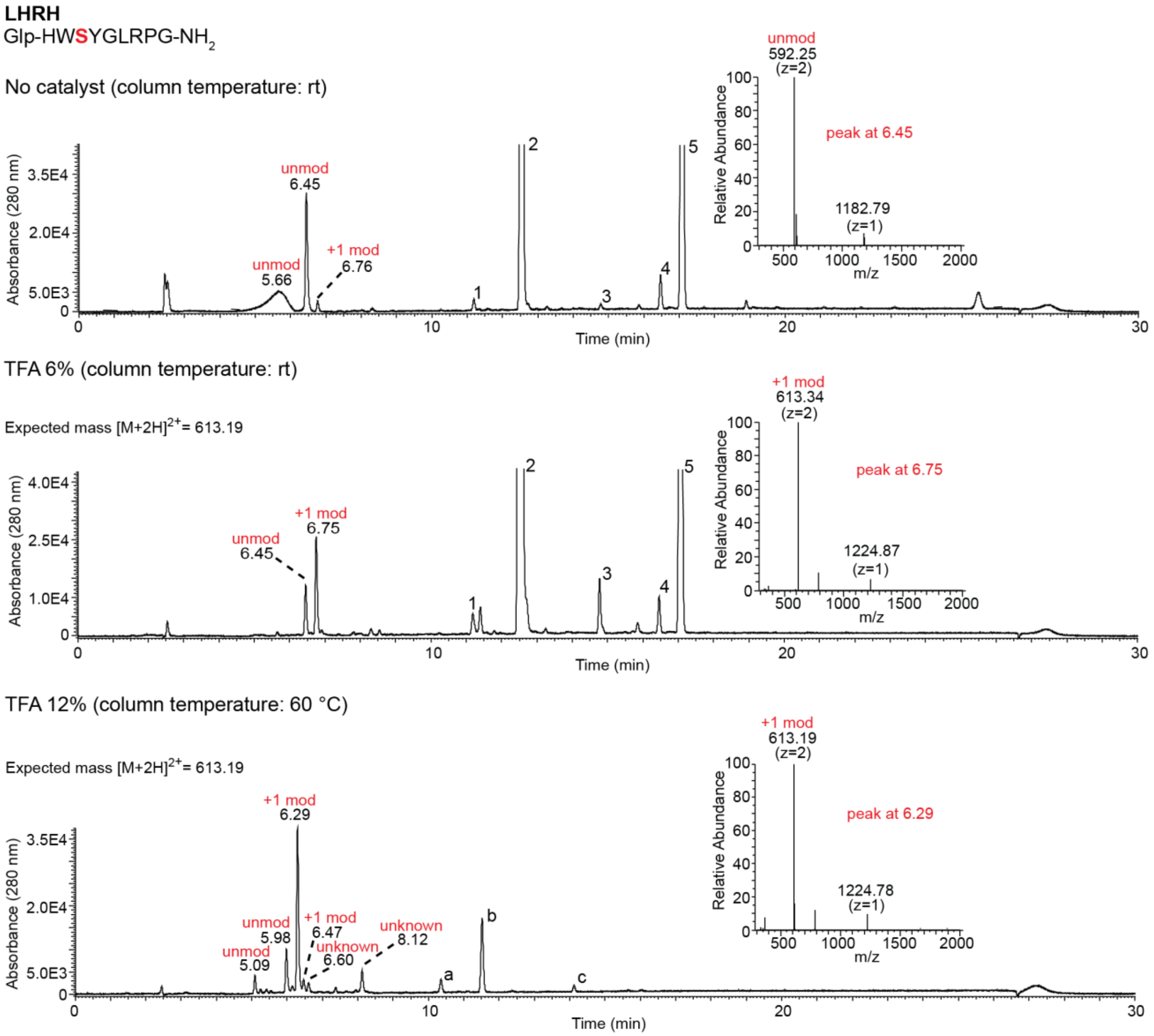

Figure S42. Liquid chromatography-mass spectrometry (LC-MS) analysis of reaction mixtures of modification of LHRH. Reaction conditions: LHRH (0.05 mM final concn from 5 mM stock solution in H_2O), trifluoroacetic acid (0–12% v/v, 0–1.6 M final concn from 0%–24% v/v stock solution in acetic acid) in acetic acid for 24 h at 50 °C. Labels 1, 2, 3, 4, and 5 in the chromatograms indicate peaks observed even in a blank sample shown in the *Liquid-chromatography mass spectrometry (LC-MS) analysis of acetic acid treated with TFA (column oven temperature: rt)* figure. 1=11.2 min, 2=12.5 min, 3=14.8 min, 4=16.5 min, 5=17.1 min when LC column oven temperature is at room temperature (rt). Labels a, b, and c in the chromatograms indicate peaks observed even in a blank sample shown in the *Liquid-chromatography mass spectrometry (LC-MS) analysis of acetic acid treated with TFA (column oven temperature: 60 °C)* figure. a=10.4 min, b=11.5 min, c=14.1 min when LC column oven temperature is 60 °C.

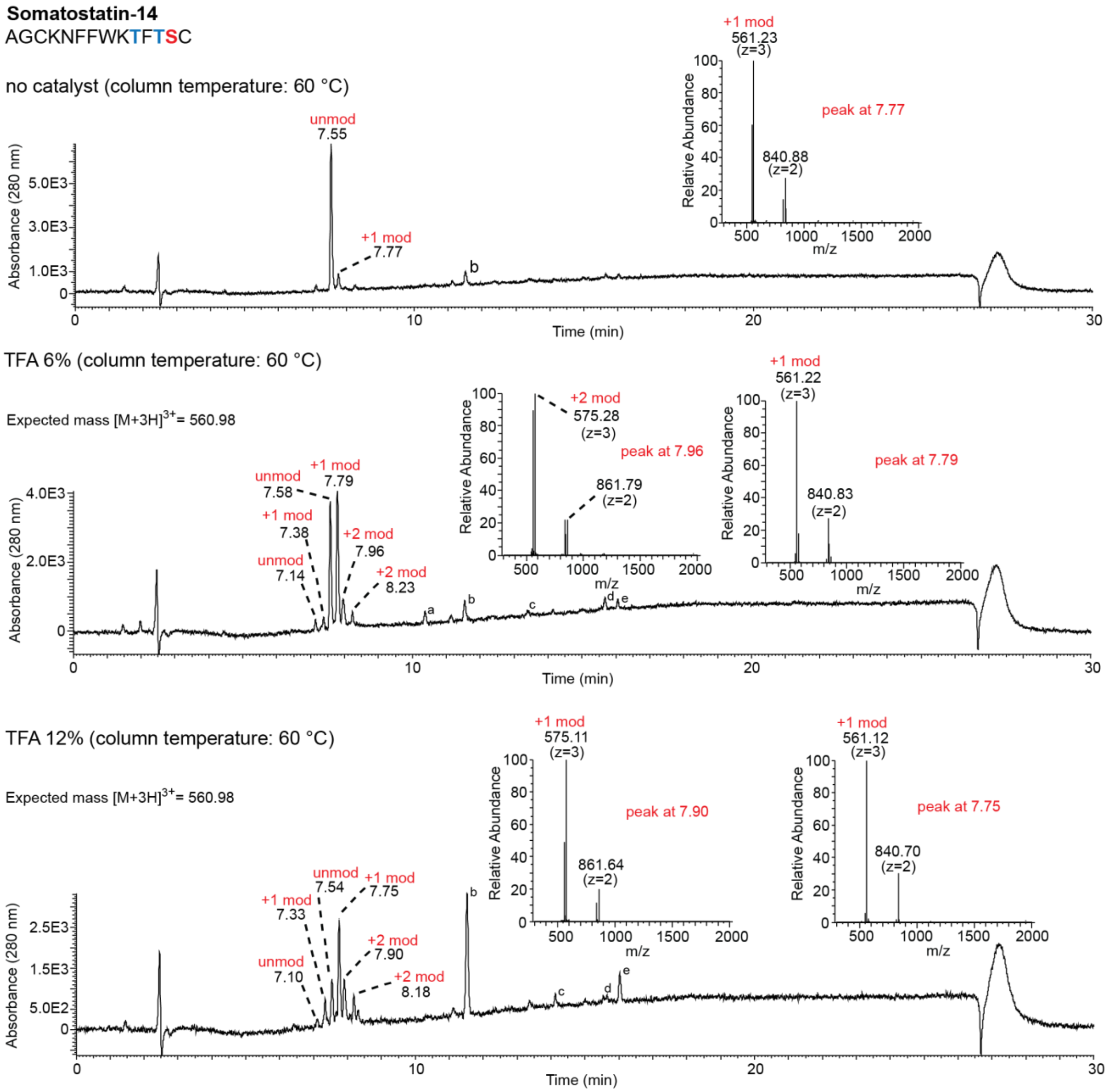

Figure S43. Liquid chromatography-mass spectrometry (LC-MS) analysis of reaction mixtures of modification of somatostatin-14. Reaction conditions: somatostatin-14 (1 mM final concn from 100 mM stock solution in acetic acid), trifluoroacetic acid (0–12% v/v, 0–1.6 M final concn from 0%–24% v/v stock solution in acetic acid) in acetic acid for 24 h at 50 °C. Labels a, b, c, d, and e in the chromatograms indicate peaks observed even in a blank sample shown in the *Liquid-chromatography mass spectrometry (LC-MS) analysis of acetic acid treated with TFA (column oven temperature: 60 °C)* figure. a=10.4 min, b=11.5 min, c=14.1 min, d=15.4 min, e=16.0 min when LC column oven temperature is 60 °C.

GHRF (1-29)

HADAIFTSSYRRILGQLYARKLLHEIMNR-NH_2

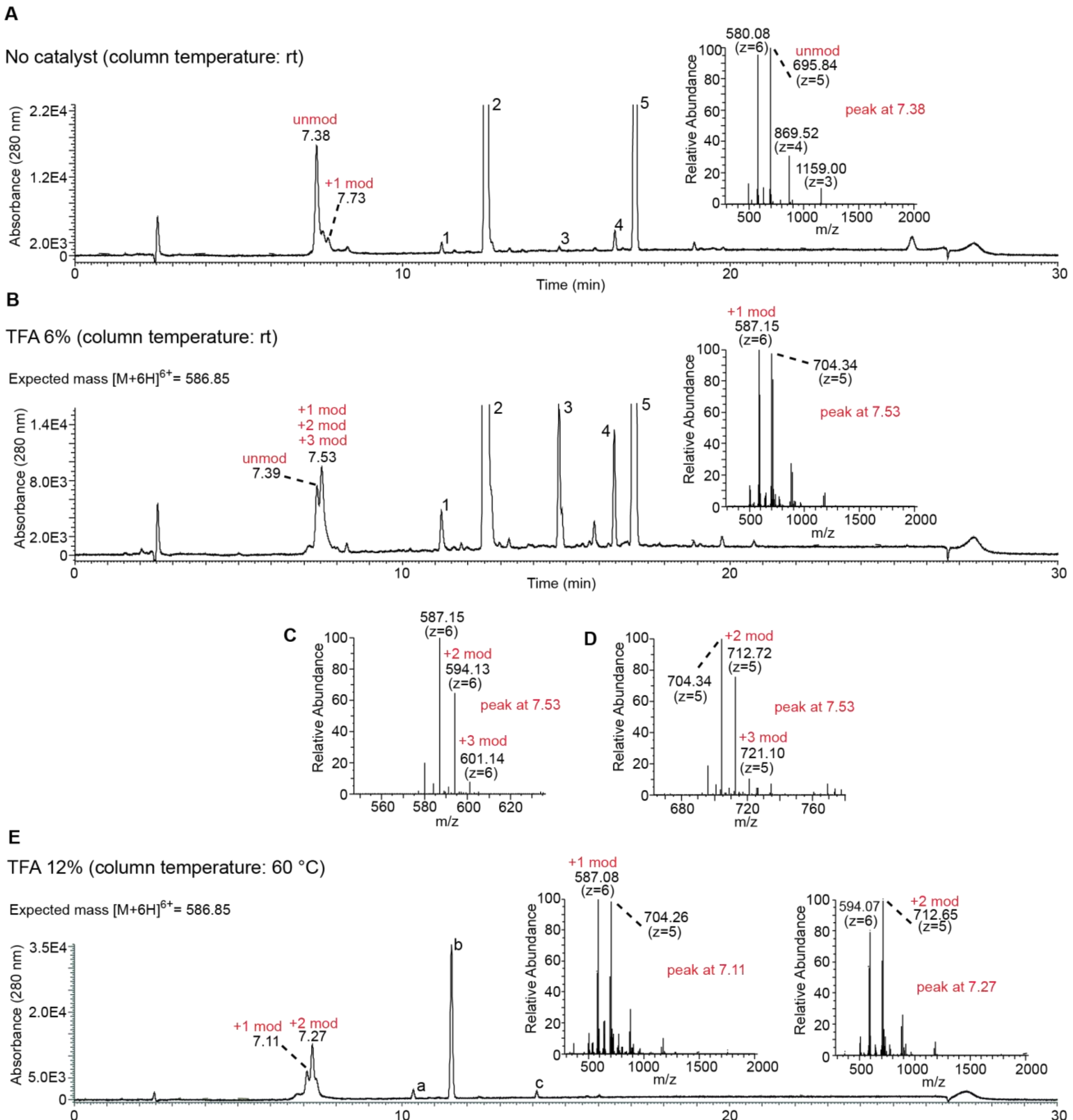

Figure S44. Liquid chromatography-mass spectrometry (LC-MS) analysis of reaction mixtures of modification of GHRF 1-29. (A) LC chromatogram and MS spectrum for the reaction without the catalyst. (B) LC chromatograms and MS spectra for the reaction with 6% v/v TFA. +2 mod and +3 mod for 6% v/v TFA are shown in separate panels (C, D). (C) MS spectrum for a peak at 7.53 min in the UV chromatogram (z=6). (D) MS spectrum for a peak at 7.53 min in the UV chromatogram (z=5). (E) LC chromatogram and MS spectra for the reaction with 12% v/v TFA. Reaction conditions: GHRF (1-29) (0.05 mM final concn from 5 mM stock solution in H_2O), trifluoroacetic acid (0–12% v/v, 0–1.6 M final concn from 0%–24% v/v stock solution in acetic acid) in acetic acid for 24 h at 50 °C. Labels 1, 2, 3, 4, and 5 in the chromatograms indicate peaks observed even in a blank sample shown in the *Liquid-chromatography mass spectrometry (LC-MS) analysis of acetic acid treated with TFA (column oven temperature: rt)* figure. 1=11.2 min, 2=12.5 min, 3=14.8 min, 4=16.5 min, 5=17.1 min when LC column oven temperature is at room temperature (rt). Labels a, b, and c in the chromatograms indicate peaks observed even in a blank sample shown in the *Liquid-chromatography mass*

spectrometry (LC-MS) analysis of acetic acid treated with TFA (column oven temperature: 60 °C) figure. a=10.4 min, b=11.5 min, c=14.1 min when LC column oven temperature is 60 °C.

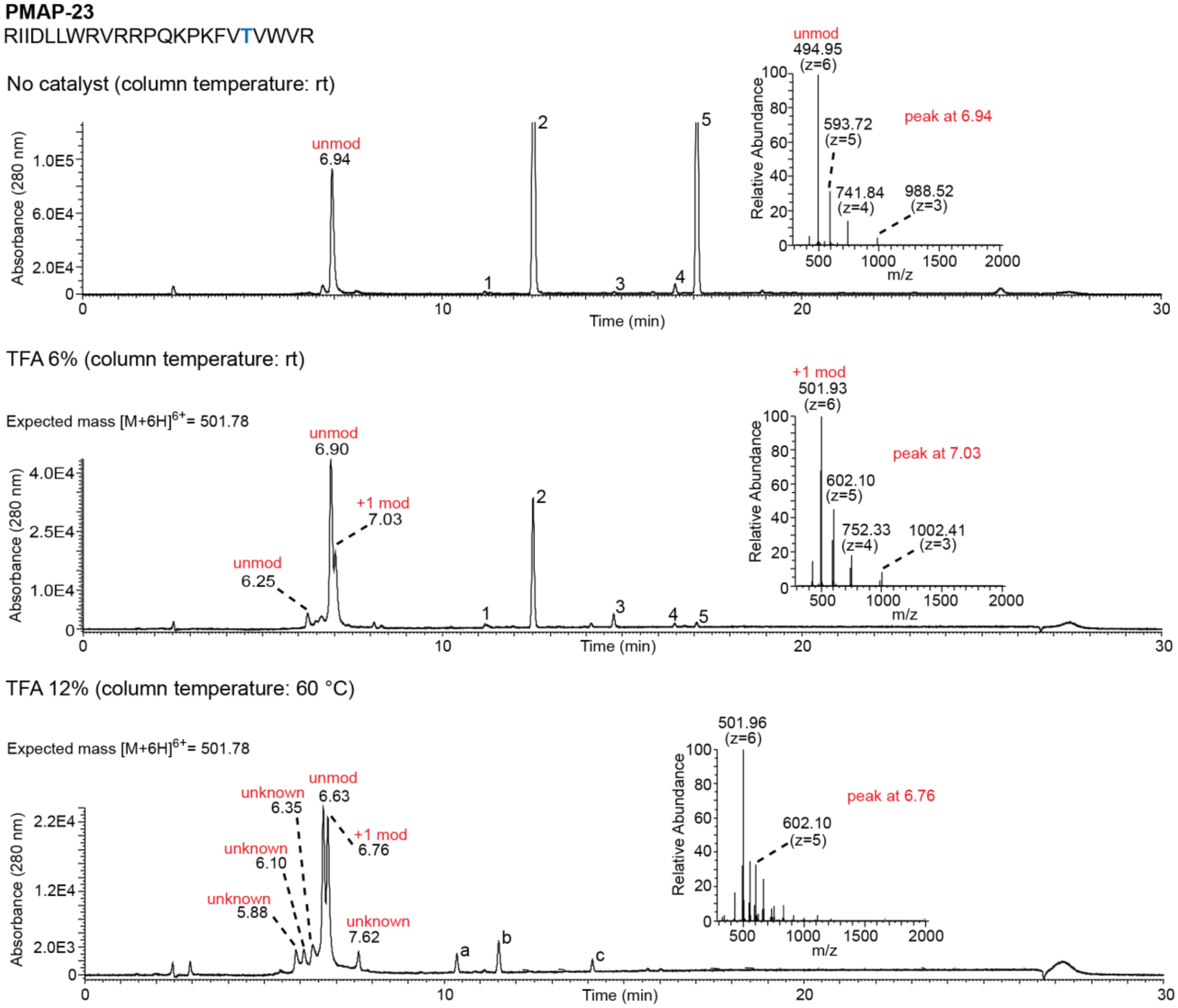

Figure S45. Liquid chromatography-mass spectrometry (LC-MS) analysis of reaction mixtures of modification of PMAP-23. Reaction conditions: PMAP-23 (0.05 mM final concn from 5 mM stock solution in H_2O), trifluoroacetic acid (0–12% v/v, 0–1.6 M final concn from 0%–24% v/v stock solution in acetic acid) in acetic acid for 24 h at 50 °C. Labels 1, 2, 3, 4, and 5 in the chromatograms indicate peaks observed even in a blank sample shown in the *Liquid-chromatography mass spectrometry (LC-MS) analysis of acetic acid treated with TFA (column oven temperature: rt)* figure. 1=11.2 min, 2=12.5 min, 3=14.8 min, 4=16.5 min, 5=17.1 min when LC column oven temperature is at room temperature (rt). Labels a, b, and c in the chromatograms indicate peaks observed even in a blank sample shown in the *Liquid-chromatography mass spectrometry (LC-MS) analysis of acetic acid treated with TFA (column oven temperature: 60 °C)* figure. a=10.4 min, b=11.5 min, c=14.1 min when LC column oven temperature is 60 °C.

PANP-004

RTRPLWVRME

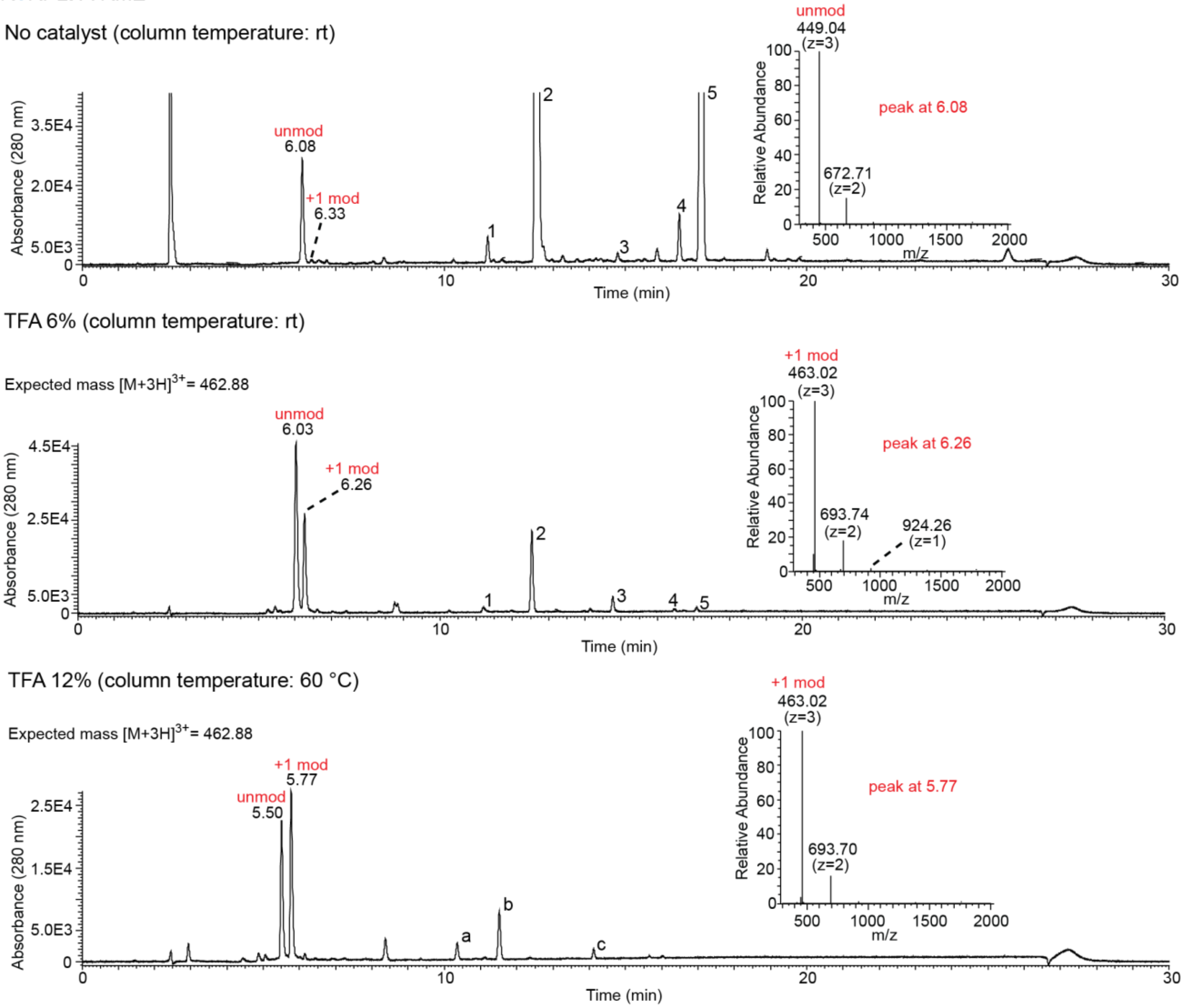

Figure S46. Liquid chromatography-mass spectrometry (LC-MS) analysis of reaction mixtures of modification of PANP-004. Reaction conditions: PANP-004 (0.05 mM final concn from 5 mM stock solution in H_2O), trifluoroacetic acid (0–12% v/v, 0–1.6 M final concn from 0%–24% v/v stock solution in acetic acid) in acetic acid for 24 h at 50 °C. Labels 1, 2, 3, 4, and 5 in the chromatograms indicate peaks observed even in a blank sample shown in the *Liquid-chromatography mass spectrometry (LC-MS) analysis of acetic acid treated with TFA (column oven temperature: rt)* figure. 1=11.2 min, 2=12.5 min, 3=14.8 min, 4=16.5 min, 5=17.1 min when LC column oven temperature is at room temperature (rt). Labels a, b, and c in the chromatograms indicate peaks observed even in a blank sample shown in the *Liquid-chromatography mass spectrometry (LC-MS) analysis of acetic acid treated with TFA (column oven temperature: 60 °C)* figure. a=10.4 min, b=11.5 min, c=14.1 min when LC column oven temperature is 60 °C.

BAMP-001
YGGFMRRVGRPE

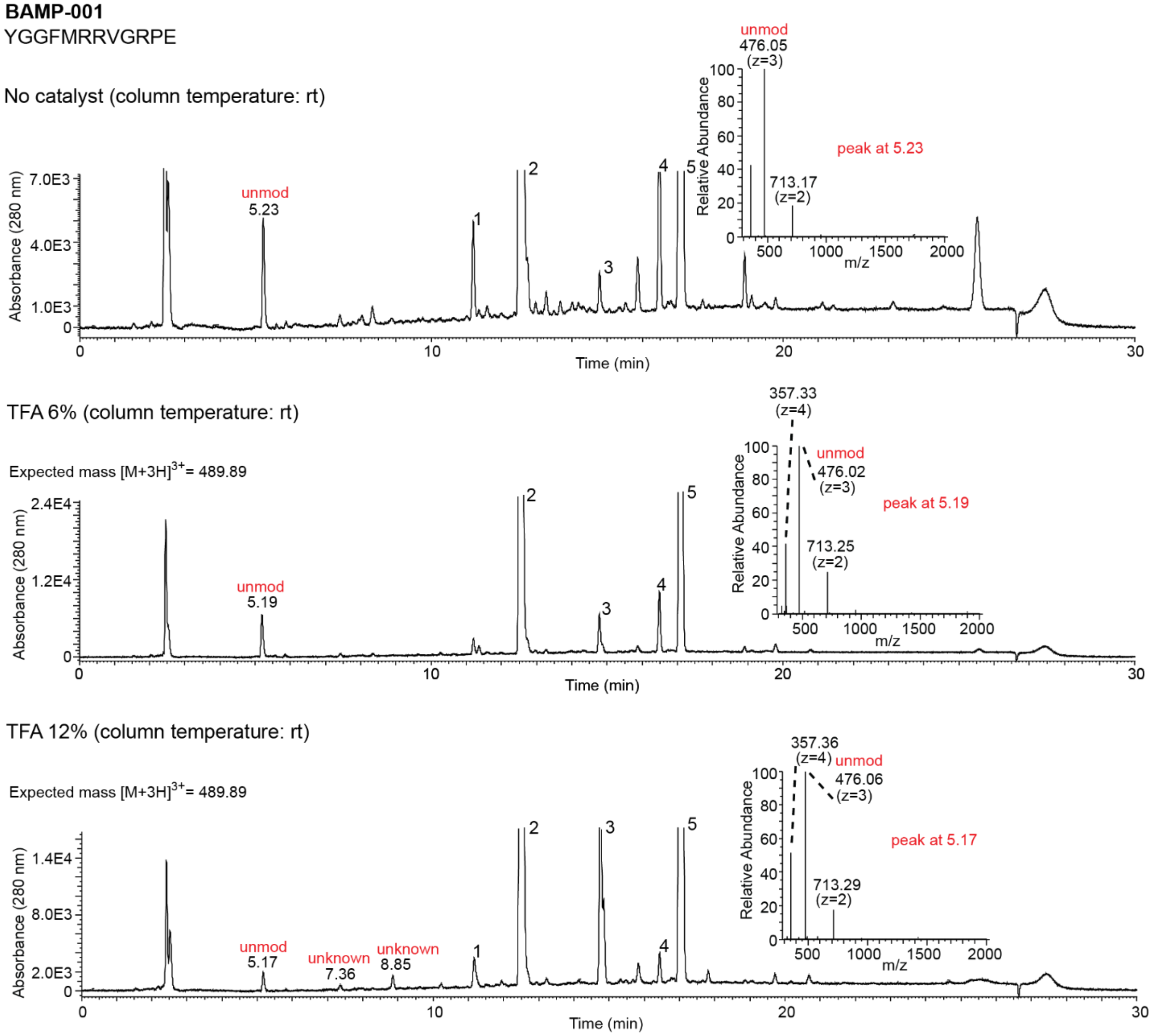

Figure S47. Liquid chromatography-mass spectrometry (LC-MS) analysis of reaction mixtures of modification of BAMP-001. Reaction conditions: BAMP-001 (0.05 mM final concn from 5 mM stock solution in H_2O), trifluoroacetic acid (0–12% v/v, 0–1.6 M final concn from 0%–24% v/v stock solution in acetic acid) in acetic acid for 24 h at 50 °C. Labels 1, 2, 3, 4, and 5 in the chromatograms indicate peaks observed even in a blank sample shown in the *Liquid-chromatography mass spectrometry (LC-MS) analysis of acetic acid treated with TFA (column oven temperature: rt)* figure. 1=11.2 min, 2=12.5 min, 3=14.8 min, 4=16.5 min, 5=17.1 min when LC column oven temperature is at room temperature (rt).

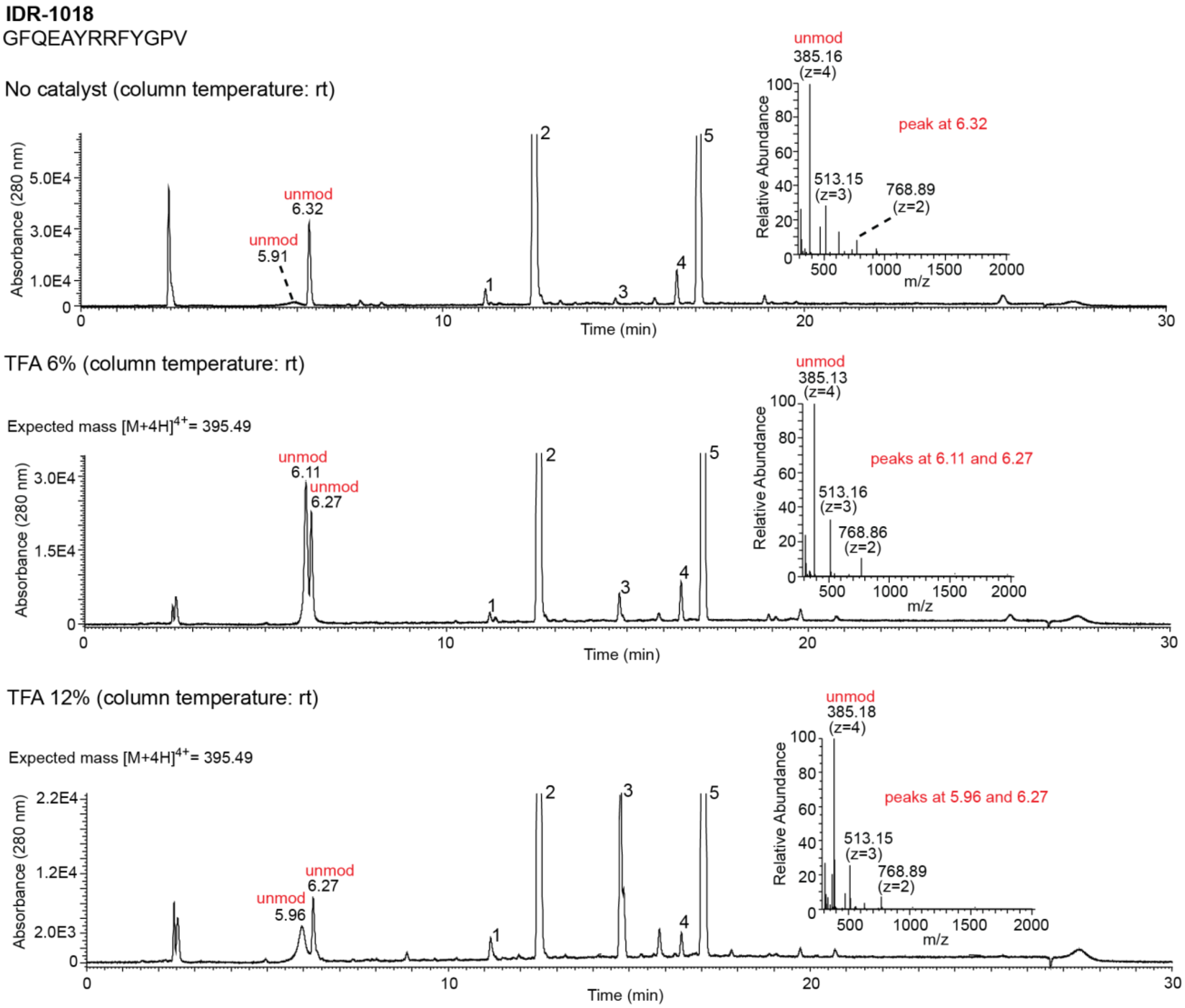

Figure S48. Liquid chromatography-mass spectrometry (LC-MS) analysis of reaction mixtures of modification of IDR-1018. Reaction conditions: IDR-1018 (0.05 mM final concn from 5 mM stock solution in H_2O), trifluoroacetic acid (0–12% v/v, 0–1.6 M final concn from 0%–24% v/v stock solution in acetic acid) in acetic acid for 24 h at 50 °C. Labels 1, 2, 3, 4, and 5 in the chromatograms indicate peaks observed even in a blank sample shown in the *Liquid-chromatography mass spectrometry (LC-MS) analysis of acetic acid treated with TFA (column oven temperature: rt)* figure. 1=11.2 min, 2=12.5 min, 3=14.8 min, 4=16.5 min, 5=17.1 min when LC column oven temperature is at room temperature (rt).

Osteocalcin (37-49)
GFQEAYRRFYGPV

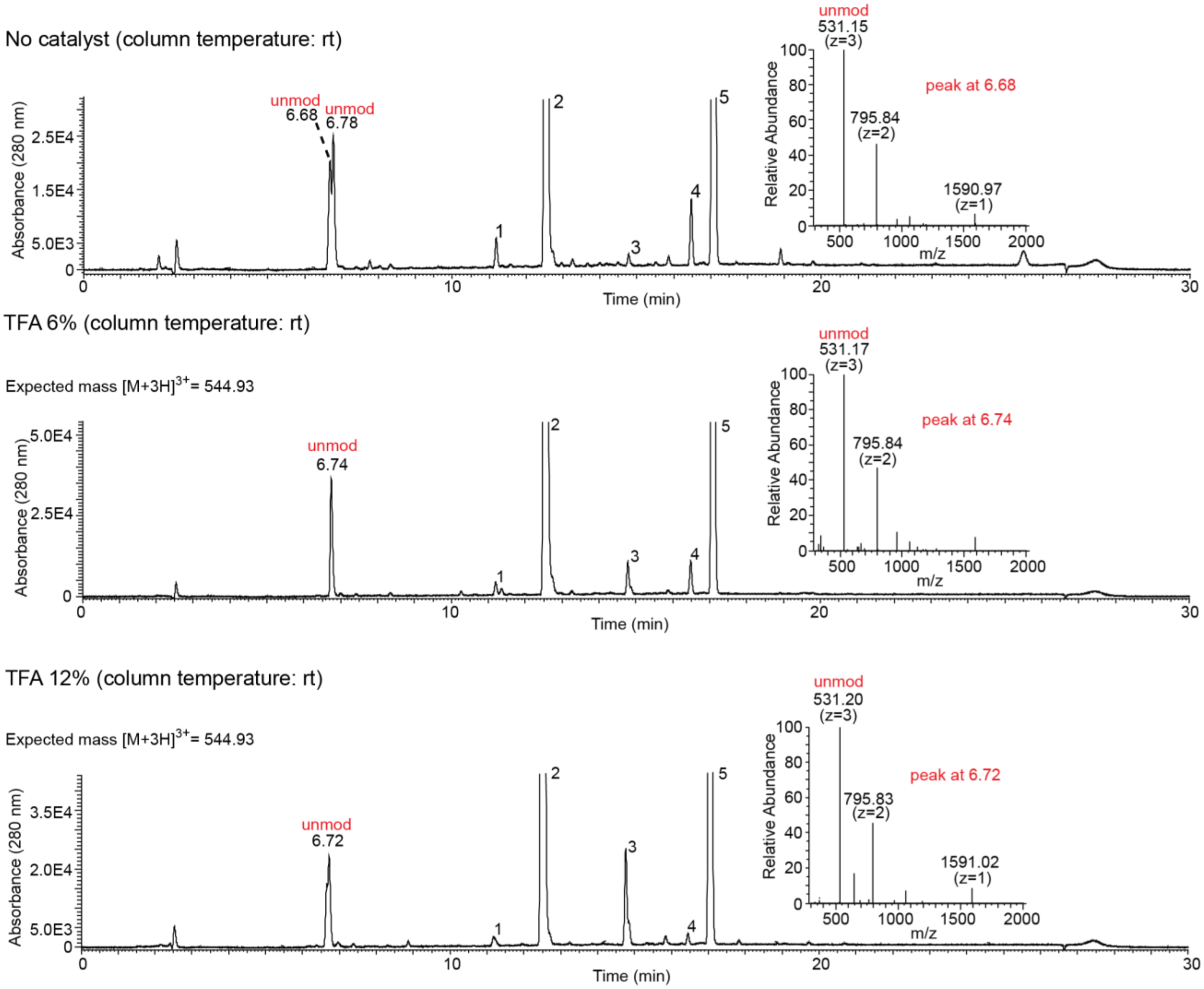

Figure S49. Liquid chromatography-mass spectrometry (LC-MS) analysis of reaction mixtures of modification of Osteocalcin (37-49). Reaction conditions: Osteocalcin (37-49) (0.05 mM final concn from 5 mM stock solution in H_2O), trifluoroacetic acid (0–12% v/v, 0–1.6 M final concn from 0%–24% v/v stock solution in acetic acid) in acetic acid for 24 h at 50 °C. Labels 1, 2, 3, 4, and 5 in the chromatograms indicate peaks observed even in a blank sample shown in the *Liquid-chromatography mass spectrometry (LC-MS) analysis of acetic acid treated with TFA (column oven temperature: rt)* figure. 1=11.2 min, 2=12.5 min, 3=14.8 min, 4=16.5 min, 5=17.1 min when LC column oven temperature is at room temperature (rt).

Dynorphin A

YGGFLRRIRPKLK

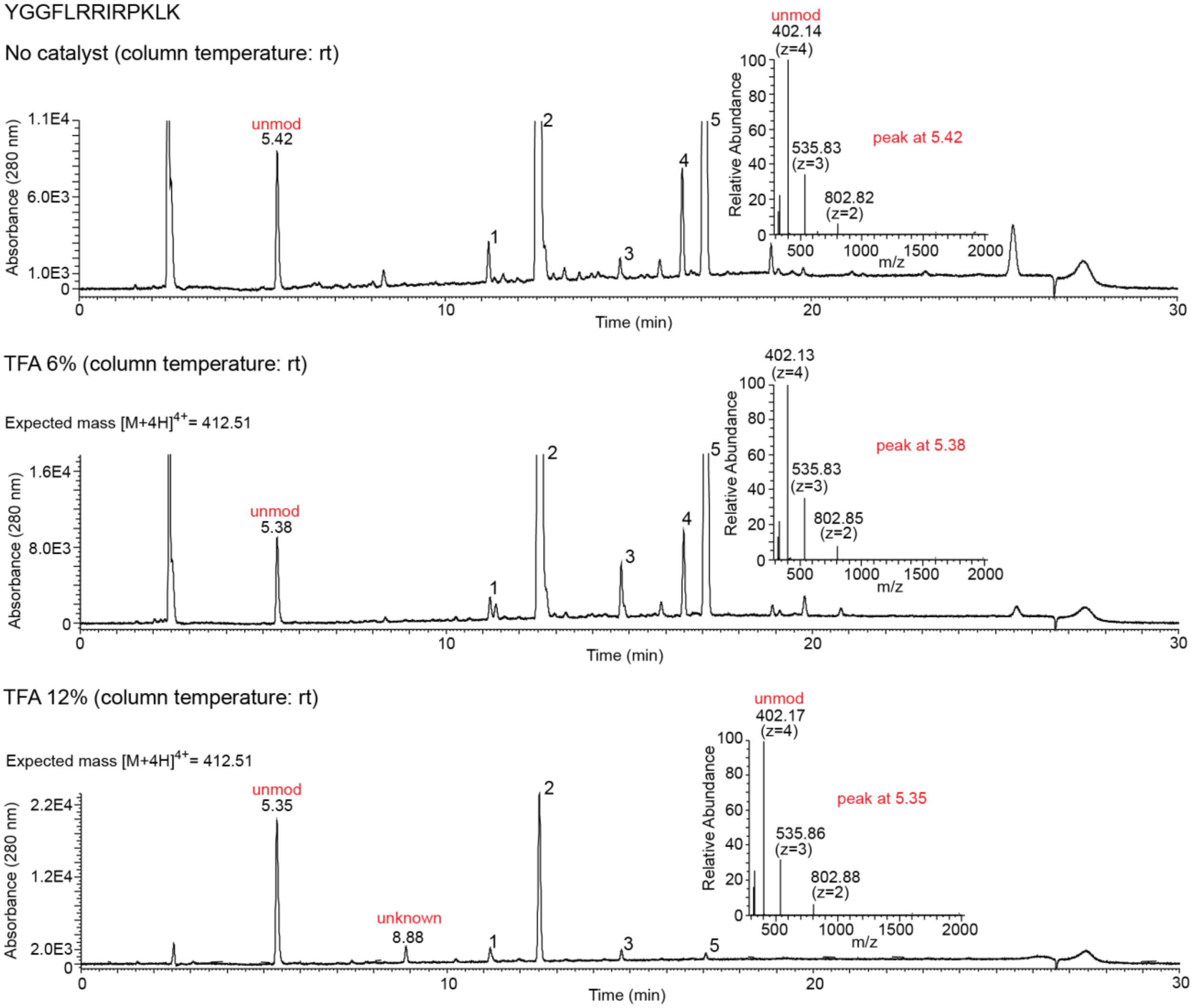

Figure S50. Liquid chromatography-mass spectrometry (LC-MS) analysis of reaction mixtures of modification of Dynorphin A. Reaction conditions: Dynorphin A (0.05 mM final concn from 5 mM stock solution in H_2O), trifluoroacetic acid (0–12% v/v, 0–1.6 M final concn from 0%–24% v/v stock solution in acetic acid) in acetic acid for 24 h at 50 °C. Labels 1, 2, 3, 4, and 5 in the chromatograms indicate peaks observed even in a blank sample shown in the *Liquid-chromatography mass spectrometry (LC-MS) analysis of acetic acid treated with TFA (column oven temperature: rt)* figure. 1=11.2 min, 2=12.5 min, 3=14.8 min, 4=16.5 min, 5=17.1 min when LC column oven temperature is at room temperature (rt).

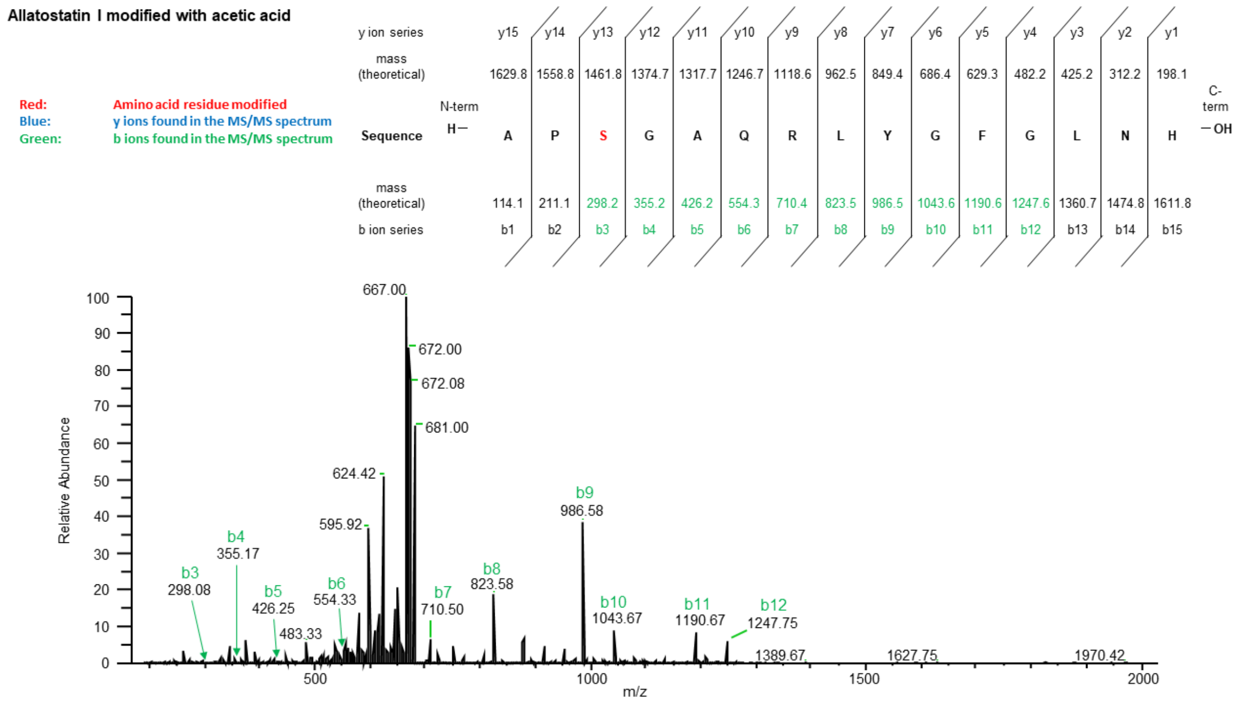

Figure S51. Tandem mass spectrum (MS/MS) analysis of allatostatin I modified with acetic acid. Reaction conditions: allatostatin I (0.05 mM final concn from 5 mM stock solution in H_2O) and trifluoroacetic acid (6% v/v, 0.78 M final concn from 12% v/v stock solution in acetic acid) were incubated in acetic acid overnight at 50 °C.

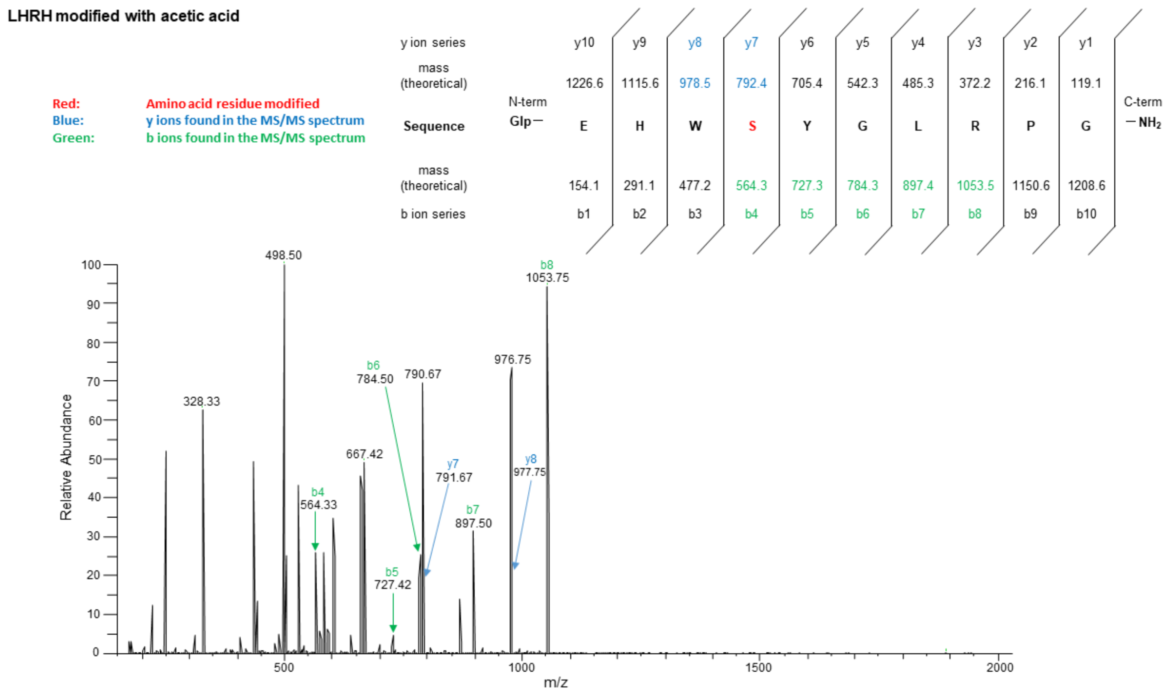

Figure S52. Tandem mass spectrum (MS/MS) analysis of LHRH modified with acetic acid. Reaction conditions: LHRH (0.05 mM final concn from 5 mM stock solution in H_2O), and trifluoroacetic acid (6% v/v, 0.78 M final concn from 12% v/v stock solution in acetic acid) were incubated in acetic acid overnight at 75 °C.

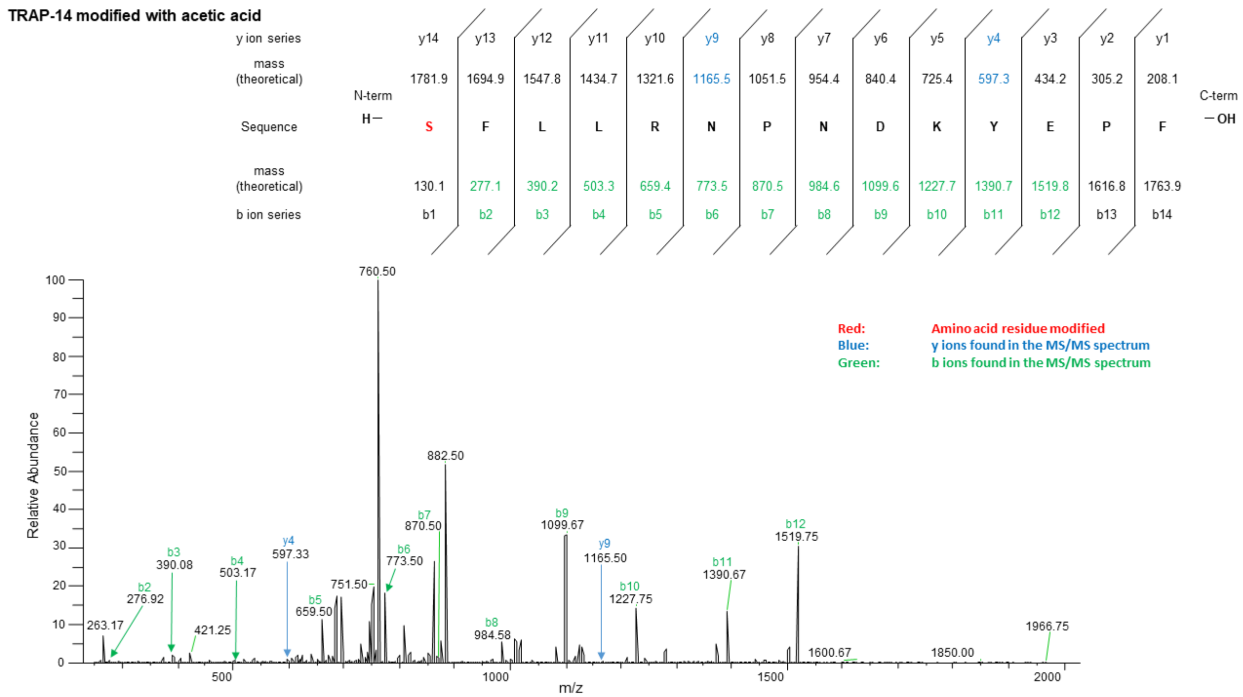

Figure S53. Tandem mass spectrum (MS/MS) analysis of TRAP-14 modified with acetic acid. Reaction conditions: TRAP-14 (0.05 mM final concn from 5 mM stock solution in H_2O), and trifluoroacetic acid (6% v/v, 0.78 M final concn from 12% v/v stock solution in acetic acid) were incubated in acetic acid for 24 h at 50 °C.

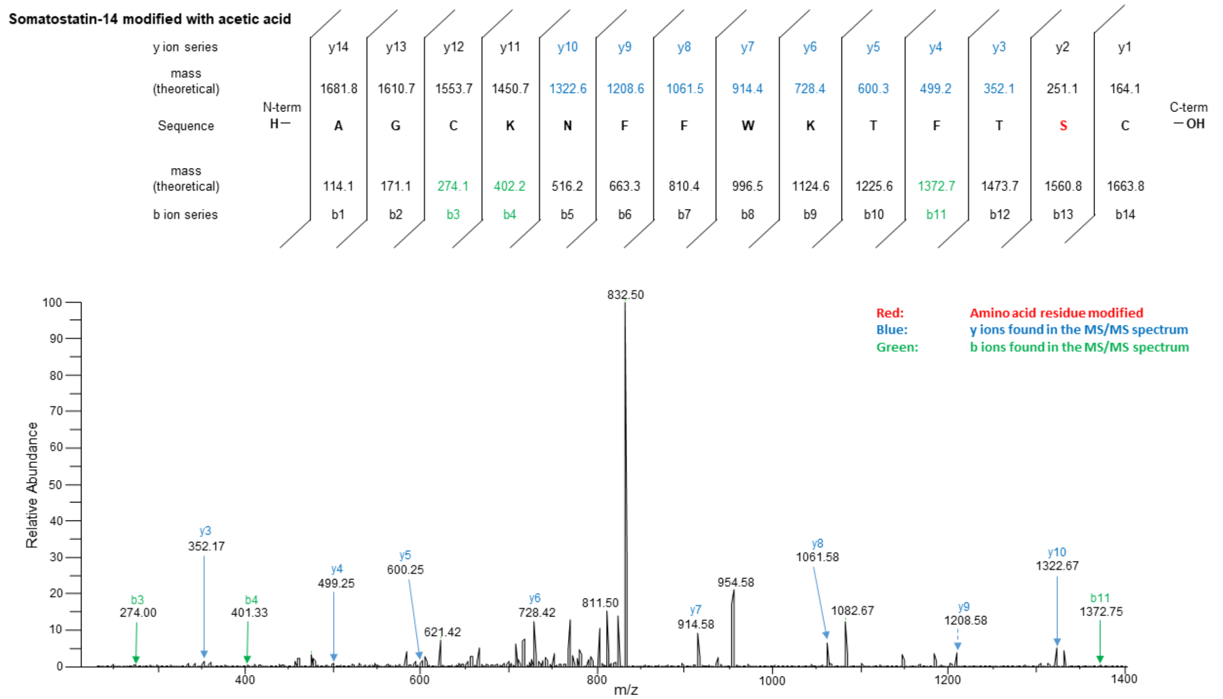

Figure S54. Tandem mass spectrum (MS/MS) analysis of somatostatin-14 modified with acetic acid. Reaction conditions: somatostatin-14 (1 mM final concentration from 100 mM stock solution in acetic acid), and trifluoroacetic acid (6% v/v, 0.78 M final concentration from 12% v/v stock solution in acetic acid) were incubated in acetic acid for 24 h at 50 °C.

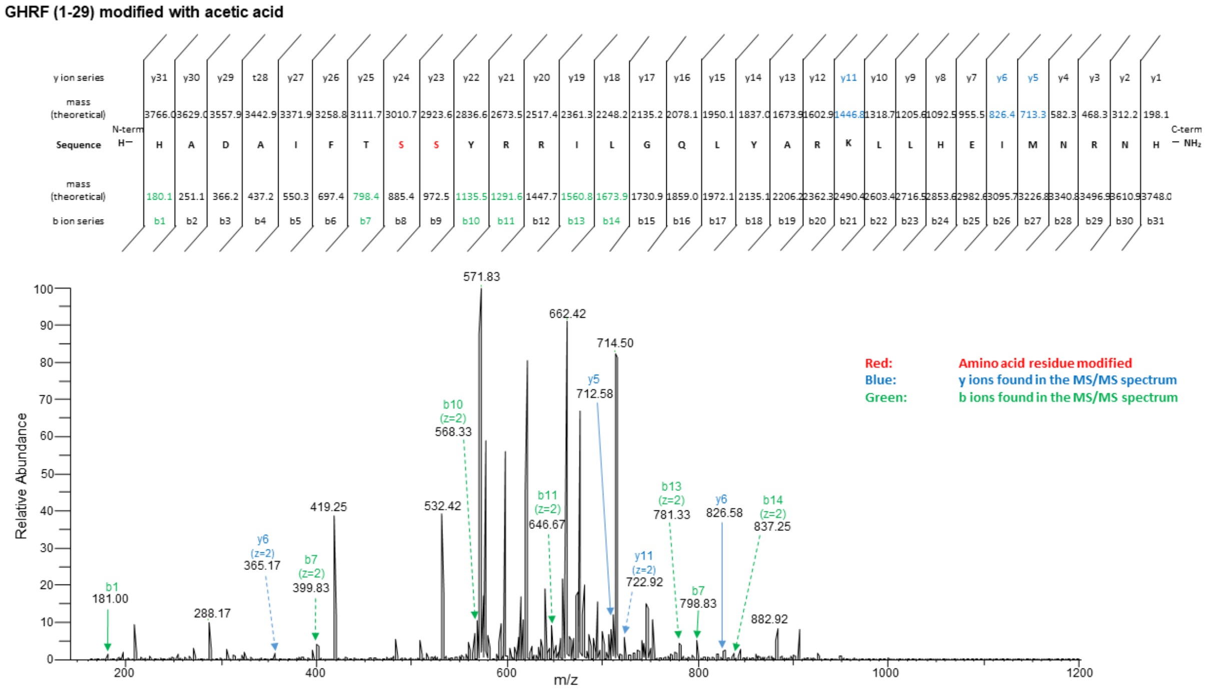

Figure S55. Tandem mass spectrum (MS/MS) analysis of GHRF (1-29) modified with acetic acid. Reaction conditions: GHRF (1-29) (0.05 mM final concn from 5 mM stock solution in H_2O), and trifluoroacetic acid (6% v/v, 0.78 M final concn from 12% v/v stock solution in acetic acid) in acetic acid for 24 h at 50 °C.

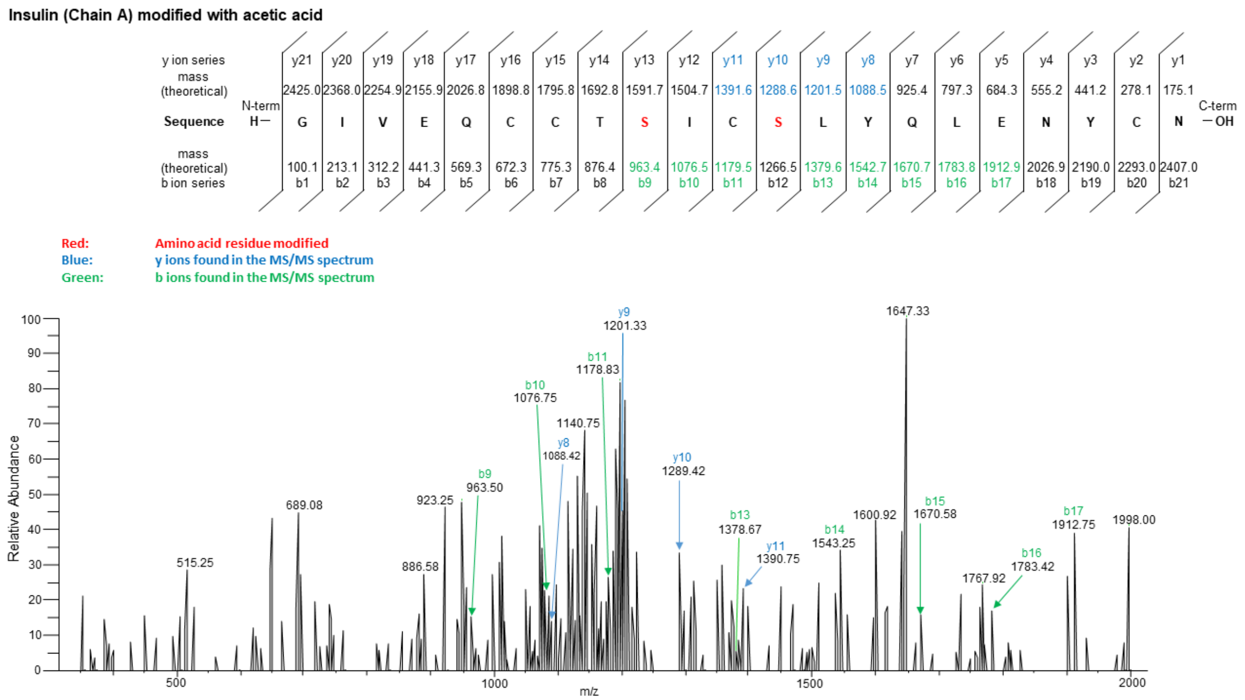

Figure S56. Tandem mass spectrum (MS/MS) analysis of Insulin (chain A) modified with acetic acid. Reaction conditions: insulin (0.1 mM final concn from 10 mM stock solution in acetic acid), and trifluoroacetic acid (3% v/v, 0.39 M final concn from 6% v/v stock solution in acetic acid) in acetic acid for 24 h at 50 °C.

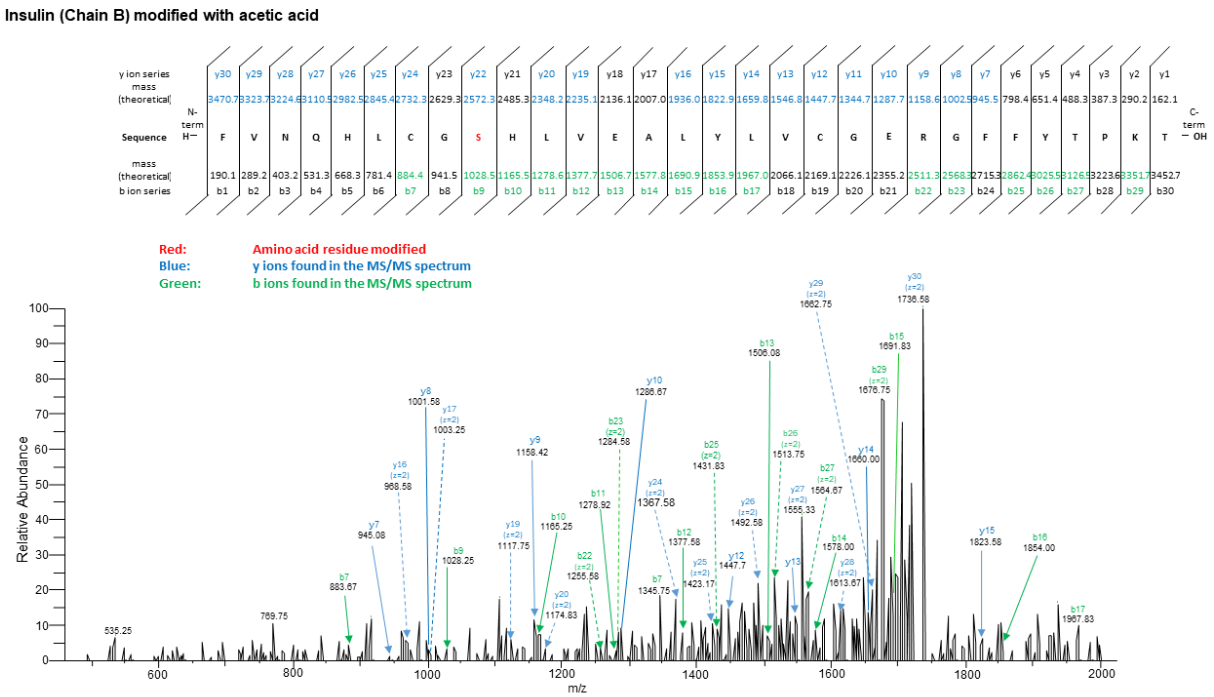

Figure S57. Tandem mass spectrum (MS/MS) analysis of Insulin (chain B) modified with acetic acid. Reaction conditions: peptide (0.1 mM final concn from 10 mM stock solution in acetic acid), and trifluoroacetic acid (3% v/v, 0.39 M final concn from 6% v/v stock solution in acetic acid) in acetic acid for 24 h at 50 °C.

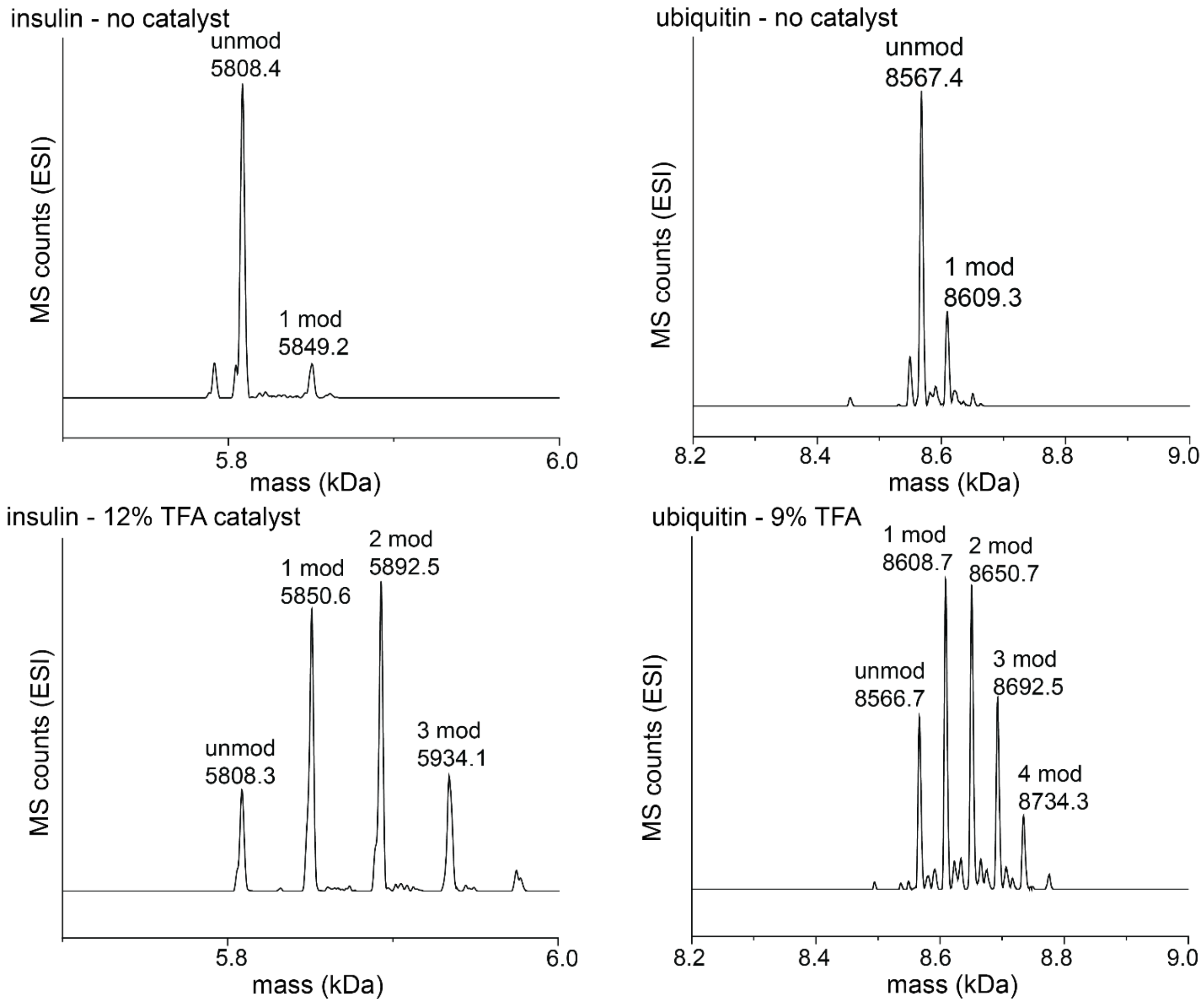

Figure S58. ESI mass spectrometry analysis of insulin and ubiquitin modified in acetic acid with TFA. Reaction conditions: proteins (see below for the concentration) and TFA (0–12% v/v, 0–1.6 M final concn from neat liquid) were incubated in acetic acid for 24 h at 37 °C. Insulin: 0.1 mM final concn from 10 mM stock solution in acetic acid. Ubiquitin: 0.05 mM final concn from 5 mM stock solution in water.

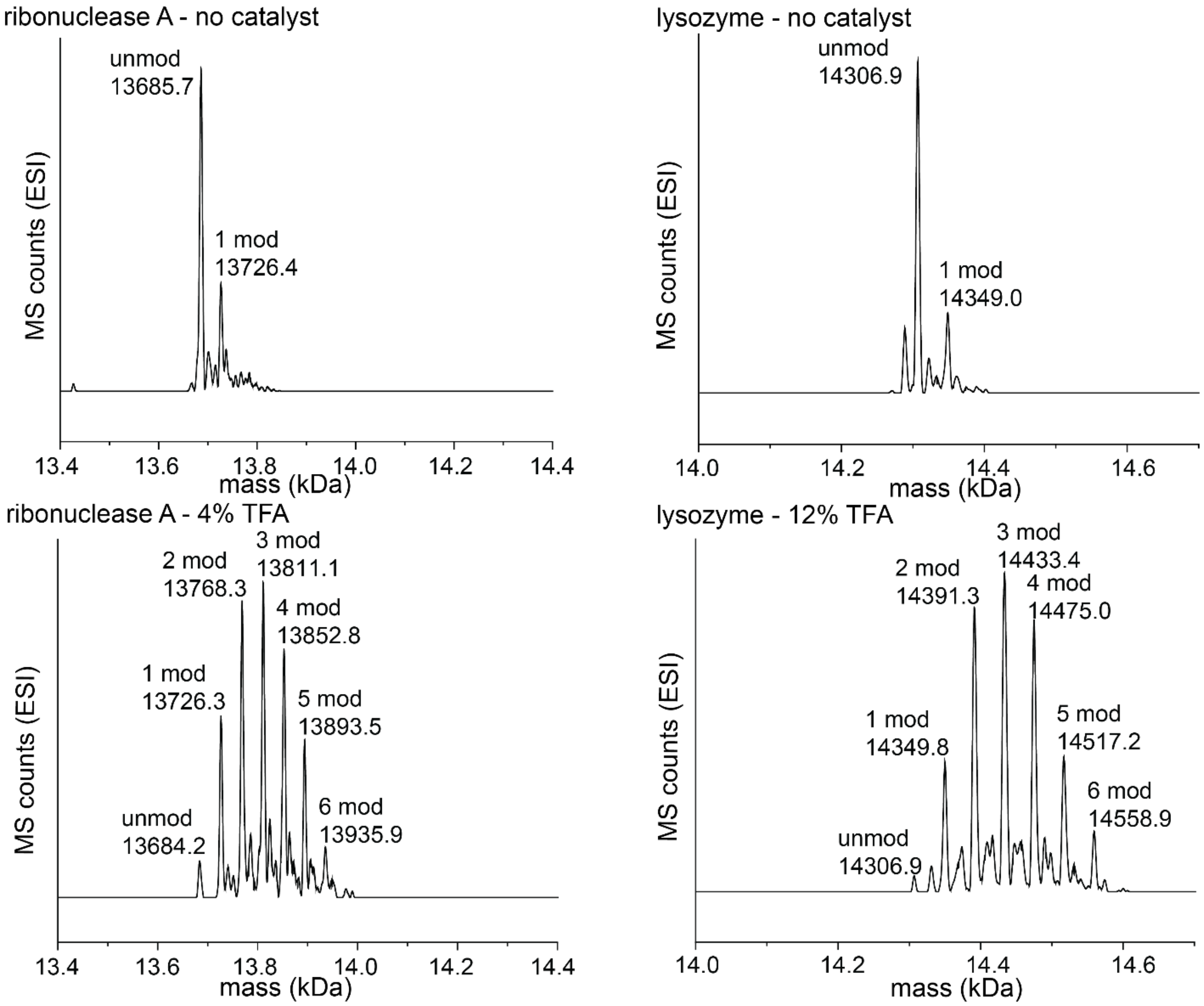

Figure S59. ESI mass spectrometry analysis of ribonuclease A and lysozyme modified in acetic acid with TFA. Reaction conditions: proteins (see below for the concentration) and TFA (0–12% v/v, 0–1.6 M final concn from neat liquid) were incubated in acetic acid for 24 h at 37 °C. ribonuclease A: 0.02 mM final concn from 2 mM stock solution in 50 mM MES buffer pH 7.4. lysozyme: 0.1 mM final concn from 10 mM stock solution in 50 mM MES buffer pH 7.4.

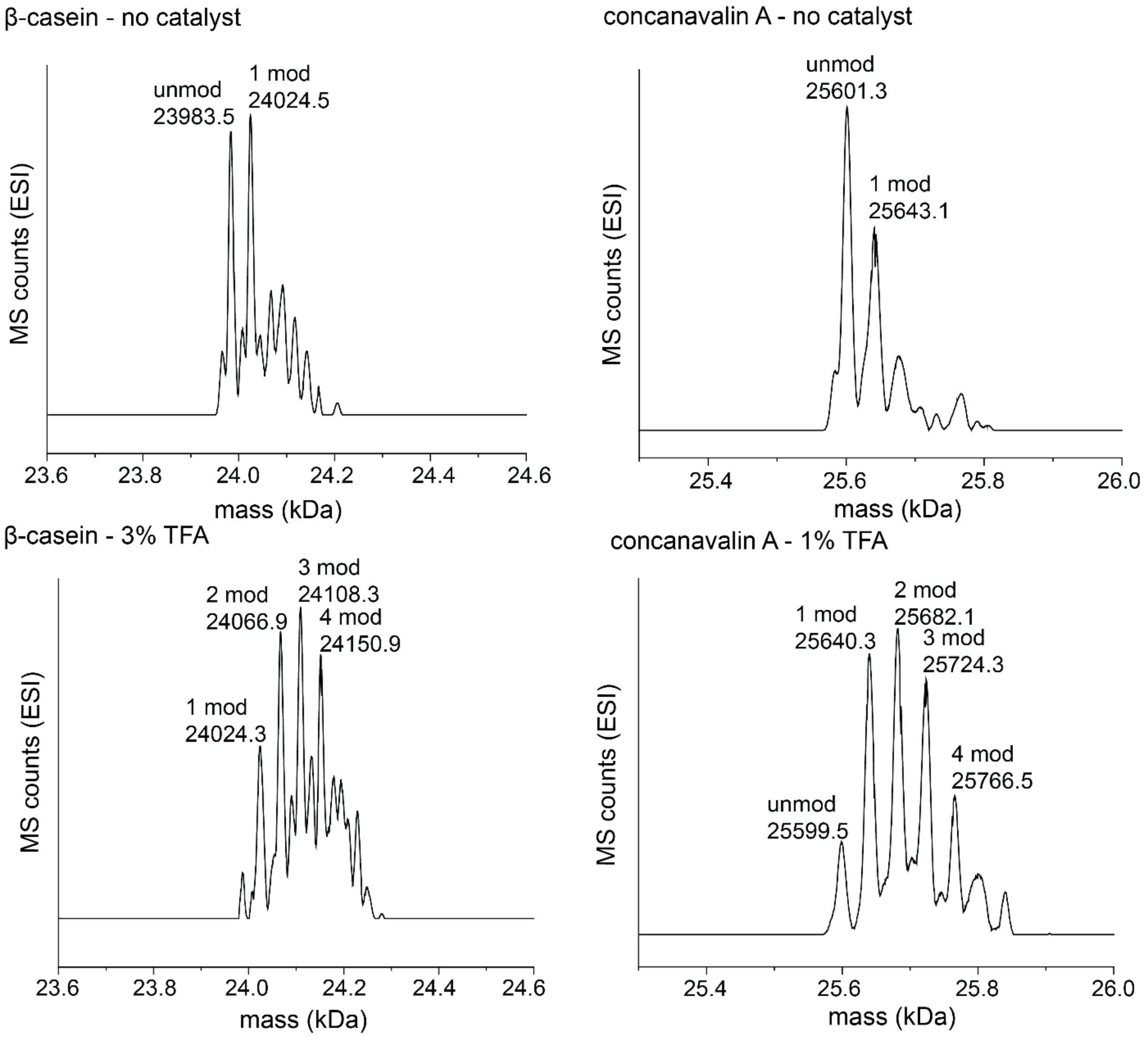

Figure S60. ESI mass spectrometry analysis of β-casein and concanavalin A modified in acetic acid with TFA. Reaction conditions: proteins (see below for the concentration) and TFA (0–3% v/v, 0–0.39 M final concn from neat liquid) were incubated in acetic acid for 24 h at 37 °C. β-casein: 0.02 mM final concn from 2 mM stock solution in 50 mM MES buffer pH 7.4. concanavalin A: 0.064 mM final concn from 6.4 mM stock solution in 50 mM N-methyl morpholine buffer pH 7.4.

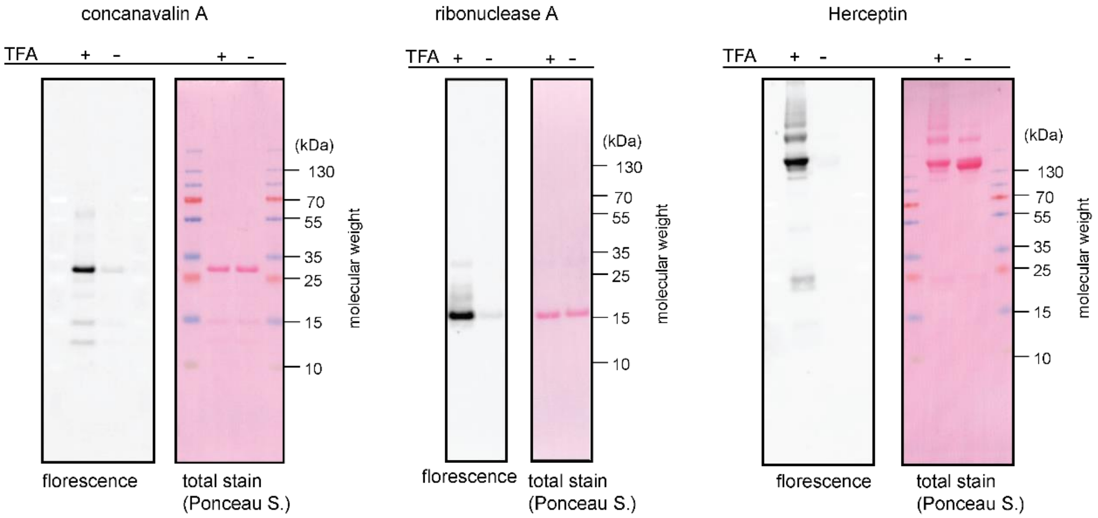

Figure S61. Chemical blot analysis (detection of an alkyne tag in proteins on a blot membrane with fluorogenic coumarin azide) of concanavalin A, ribonuclease A, and Herceptin modified with 4-pentynoic acid. Reaction conditions: Protein (see below for the concentration) and TFA (3% v/v, 0.39 M final concn from neat liquid) were incubated in 4-pentynoic acid (4 M in EMIM BF_4) at 37 °C for 24 h. Concentrations of proteins: concanavalin A (192 µM final concn from 6.4 mM stock solution in 50 mM MES buffer pH 7.4), ribonuclease A (60 µM final concn from 2 mM stock solution in 50 mM MES buffer pH 7.4), Herceptin (46 µM final concn from 766 µM stock solution in 50 mM MES buffer pH 7.4). Fluorescence imaging was performed by 460 nm excitation with 525 nm bandpass filter (±20 nm). Molecular weight marker: Thermo Scientific 26619.

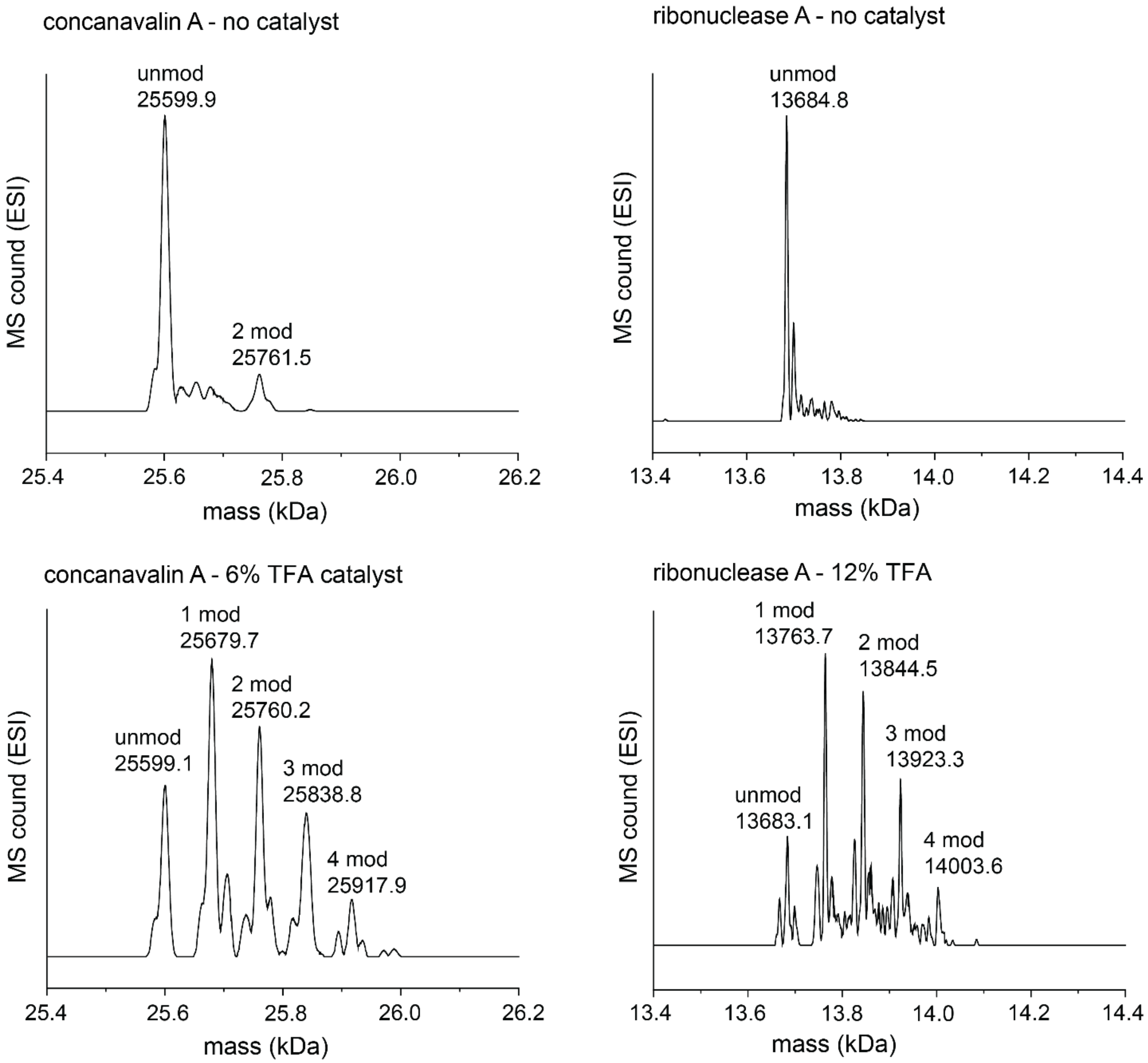

Figure S62. ESI mass spectrometry analysis of concanavalin A and ribonuclease A modified with 4-pentynoic acid. Reaction conditions: Protein (see below for the concentration) and TFA (0–12% v/v, 0–1.6 M final concn from neat liquid) were incubated in 4-pentynoic acid (4 M in EMIM BF_4) at 37 °C for 24 h. Concentrations of proteins: Concanavalin A (192 µM final concn from 6.4 mM stock solution in 50 mM MES buffer pH 7.4), ribonuclease A (60 µM final concn from 2 mM stock solution in 50 mM MES buffer pH 7.4).

Table S4. MS counts for peptide fragments that contain expected modifications (either acetic acid +42.0370 or TFA +96.0082 for lysozyme, and 4-pentynoic acid +80.0860 or TFA +96.0082 for α-chymotrypsinogen A and ribonuclease A). Ser, Thr, Tyr, and Lys residues were set as possible modifications. The number of these residues of each protein is shown in *the number of Ser, Thr, Tyr, and Lys residues of lysozyme, α-chymotrypsinogen A, and ribonuclease A* table.

lysozyme	modification with acetic acid				modification with TFA			
residues	Ser	Thr	Tyr	Lys	Ser	Thr	Tyr	Lys
MS counts	1910	631	110	107	29	6	0	19

α-chymotrypsinogen A	modification with 4-pentynoic acid				modification with TFA			
residues	Ser	Thr	Tyr	Lys	Ser	Thr	Tyr	Lys
MS counts	1007	96	13	98	20	6	3	12

ribonuclease A	modification with 4-pentynoic acid				modification with TFA			
residues	Ser	Thr	Tyr	Lys	Ser	Thr	Tyr	Lys
MS counts	265	161	30	44	1	1	0	2

Table S5. The number of Ser, Thr, Tyr, and Lys residues of lysozyme, α-chymotrypsinogen A, and ribonuclease A.

residues	Ser	Thr	Tyr	Lys
lysozyme	10	7	3	6
α-chymotrypsinogen A	28	23	4	14
ribonuclease A	15	10	6	10

Proteomics analysis of lysozyme modified with acetic acid

Uniprot ID: P00698

Sequence
KVFGRCELAAAMKRHGLDNYRGYSLGNWVCAAKFESNFNTQA
TNRNTDGSTDYGILQINSRWWCNDGRTPGSRNLCNIPCSALLSSDITASVNCAKKIVSDG
NGMNAWVAWRNRCKGTDVQAWIRGCRL

Sequence
KVFGRCELAAAMKRHGLDNYRGYSLGNWVCAAKFESNFNTQA
TNRNTDGSTDYGILQINSRWWCNDGRTPGSRNLCNIPCSALLSSDITASVNCAKKIVSDG
NGMNAWVAWRNRCKGTDVQAWIRGCRL

Coverage 100%
Modification site of **acetic acid**

Modification site of **TFA**

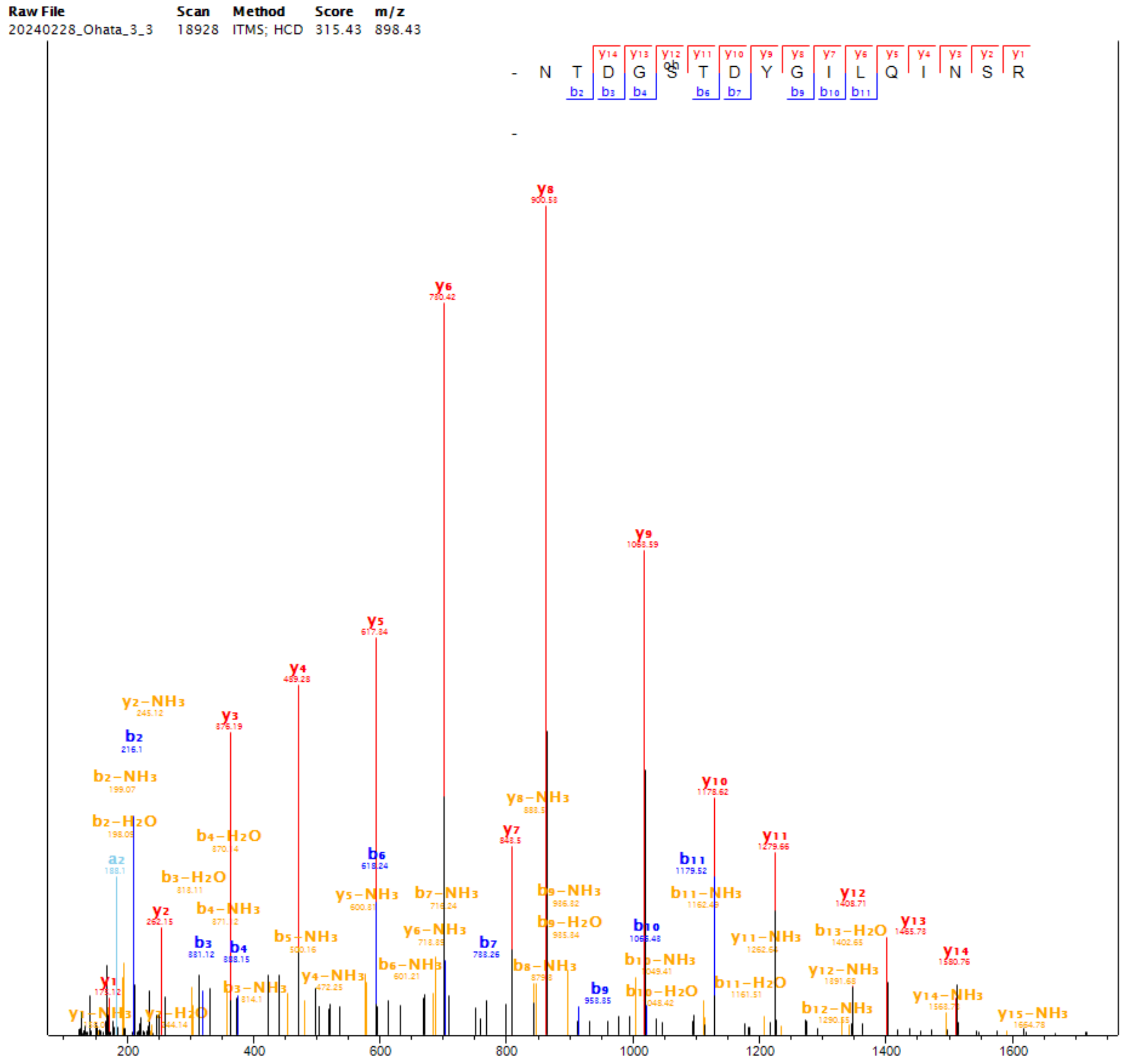

Figure S63. MS/MS analysis of the peak corresponding to labeled Ser50 of lysozyme (NTDGS*TDYGILQINSR) as a representative Ser-labeled fragment with acetic acid.

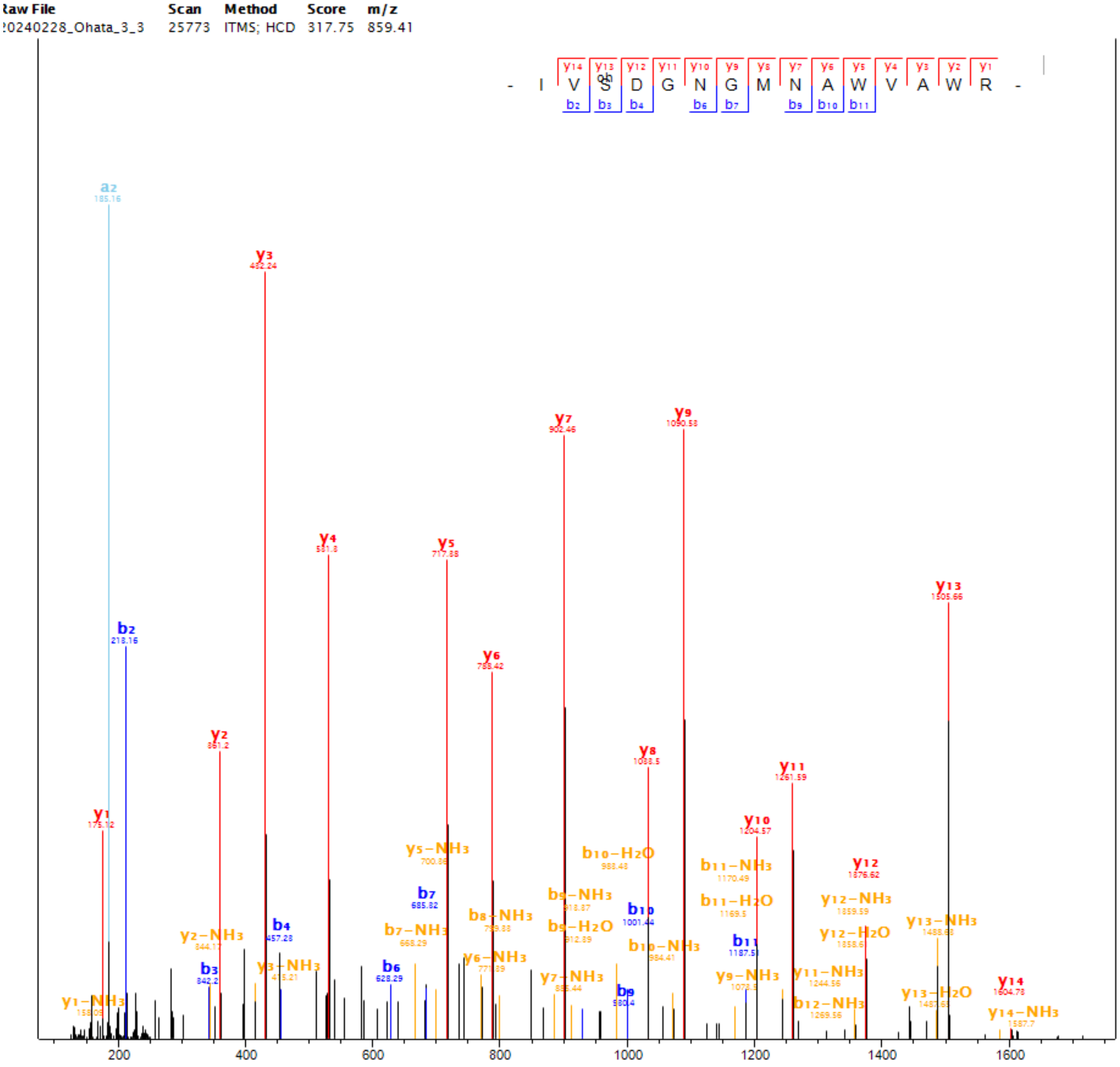

Figure S64. MS/MS analysis of the peak corresponding to labeled Ser100 of lysozyme (IVS*DGNGMNAWVAWR) as a representative Ser-labeled fragment with acetic acid.

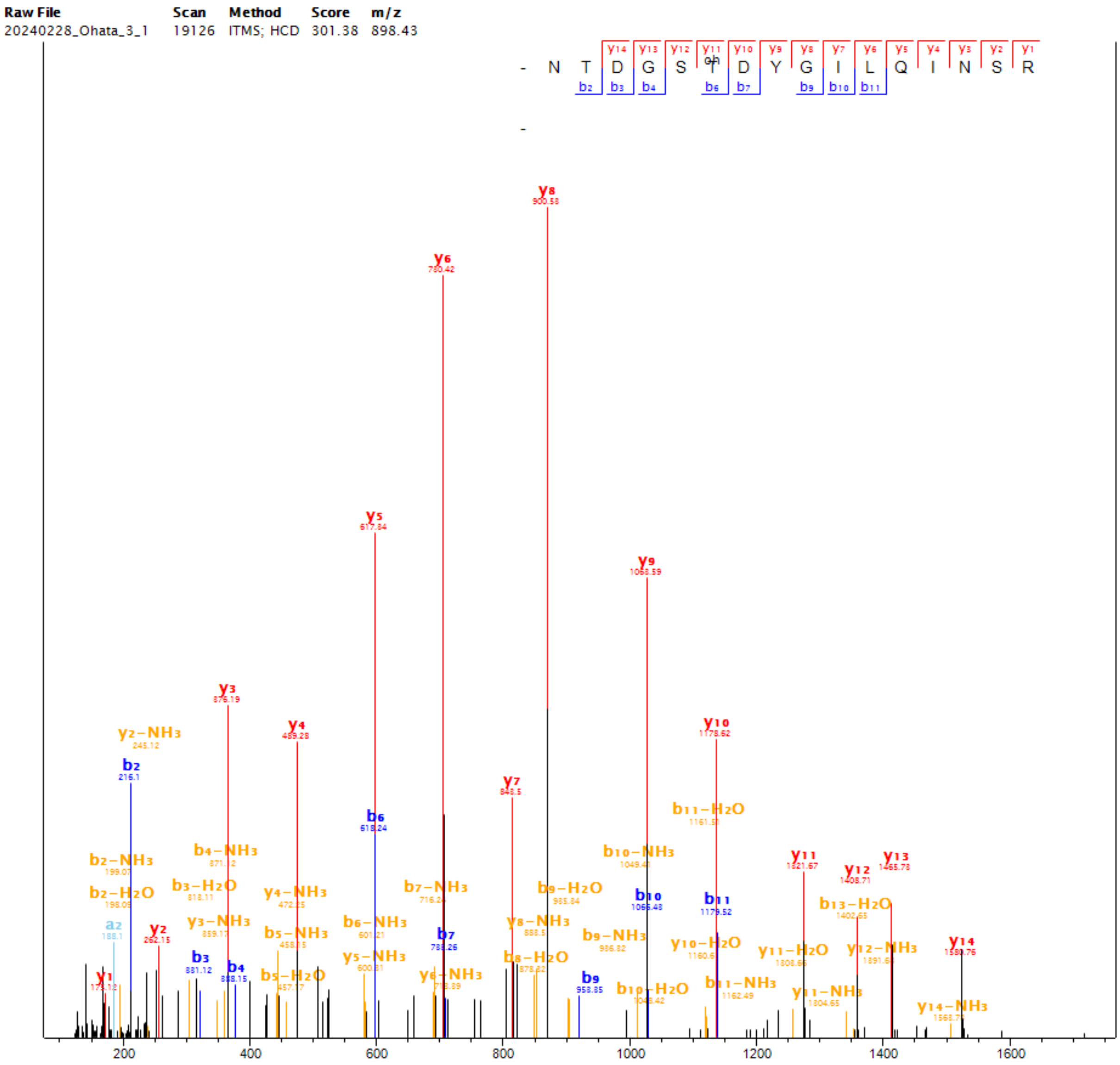

Figure S65. MS/MS analysis of the peak corresponding to labeled Thr51 of lysozyme (NTDGST*DYGILQINSR) as a representative Thr-labeled fragment with acetic acid.

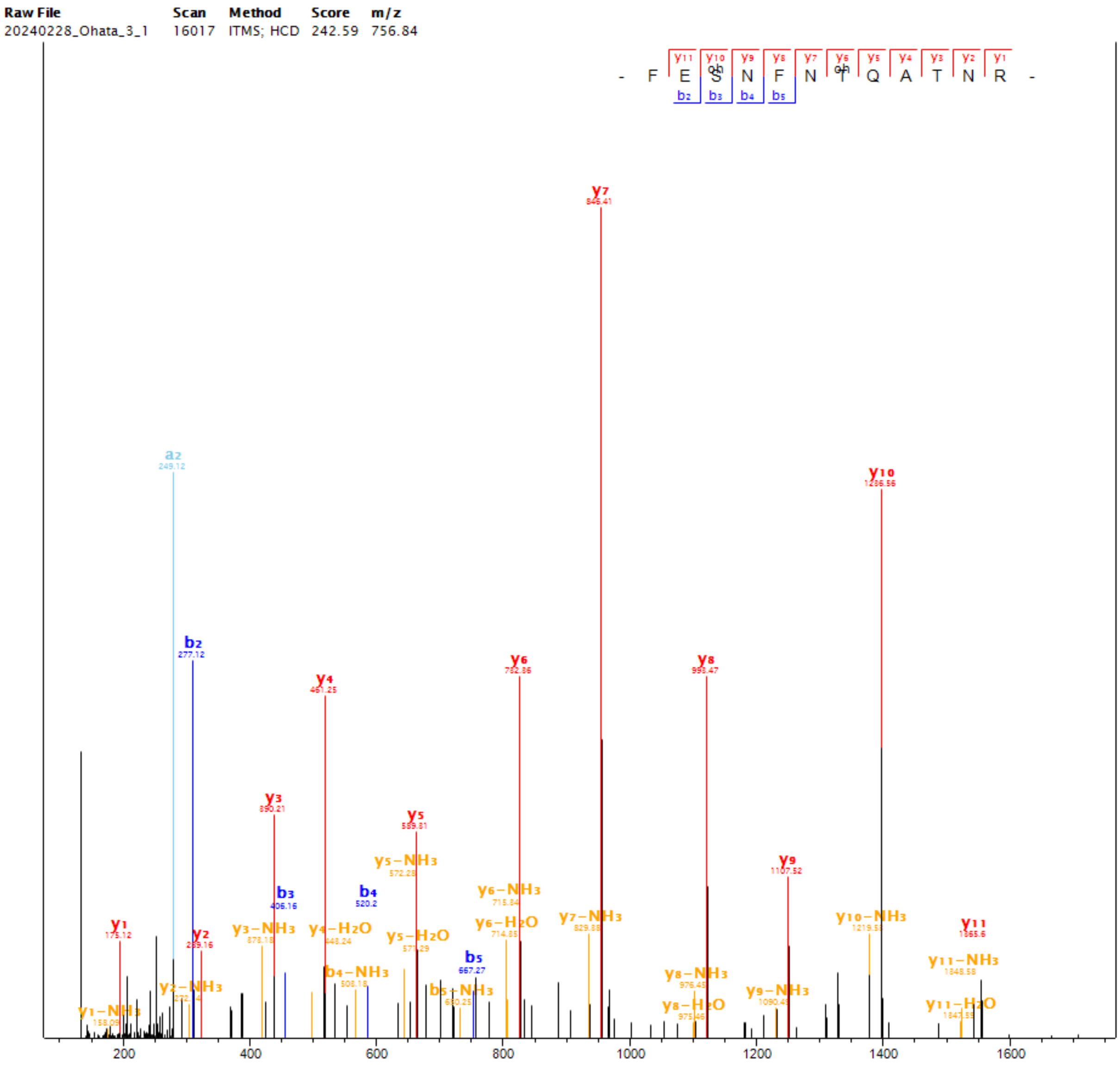

Figure S66. MS/MS analysis of the peak corresponding to labeled Ser36 and Thr40 of lysozyme (FES*NFNT*QATNR) as a representative Ser- and Thr-labeled fragment with acetic acid.

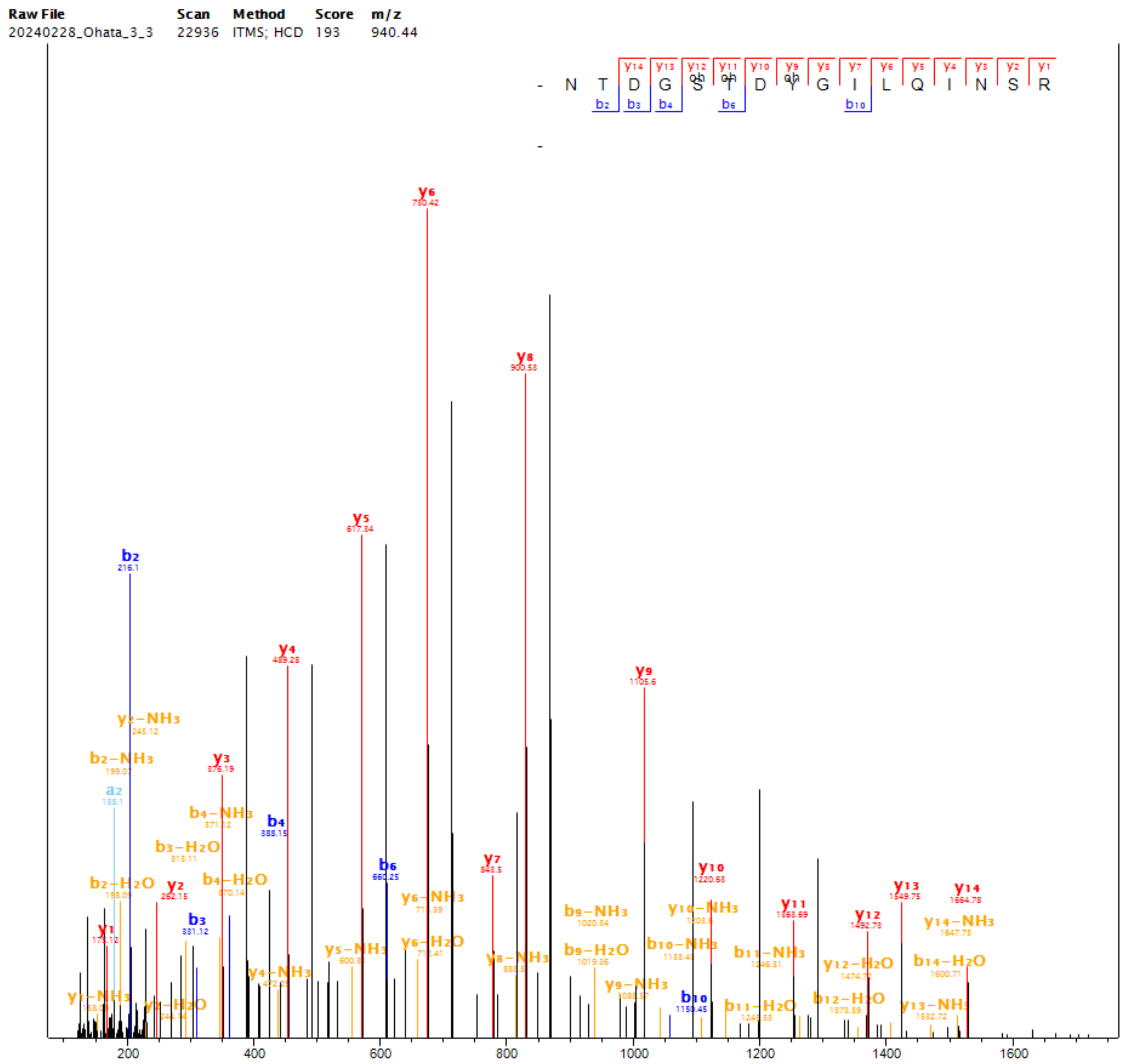

Figure S67. MS/MS analysis of the peak corresponding to labeled Ser50, Thr51, and Tyr53 of lysozyme (NTDGS*T*DY*GILQINSR) as a representative Ser-, Thr- and Tyr-labeled fragment with acetic acid.

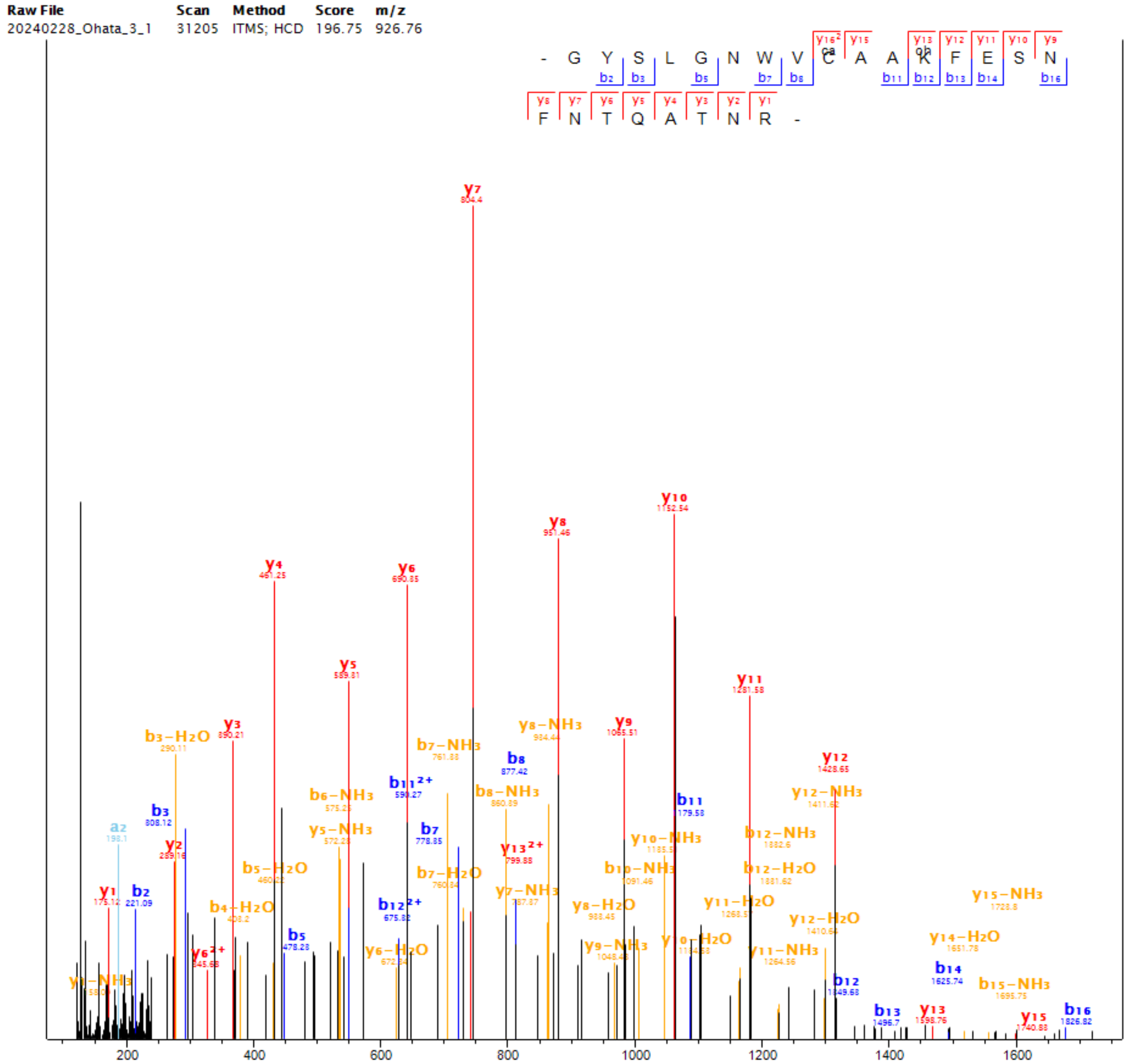

Figure S68. MS/MS analysis of the peak corresponding to labeled Lys33 of lysozyme (GYSLGNWVC^{CAM}AAK*FESNFNTQATNR) as a representative Lys-labeled fragment with acetic acid.

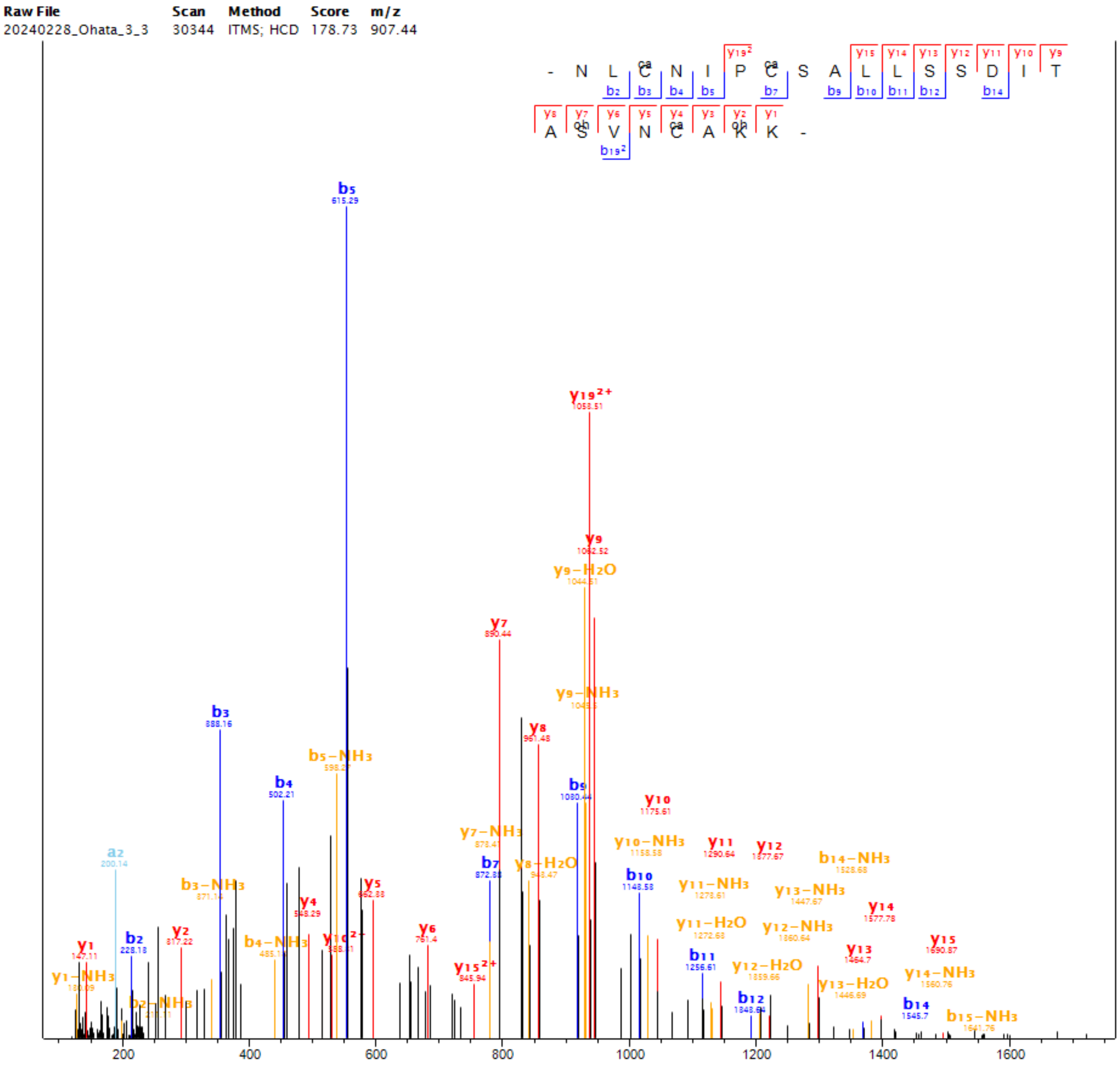

Figure S 69. MS/MS analysis of the peak corresponding to labeled Ser91 and Lys96 of lysozyme (NLC^{CAM}NIPC^{CAM}SALLSSDITAS*VNC^{CAM}K*K) as a representative Ser- and Lys-labeled fragment with acetic acid.

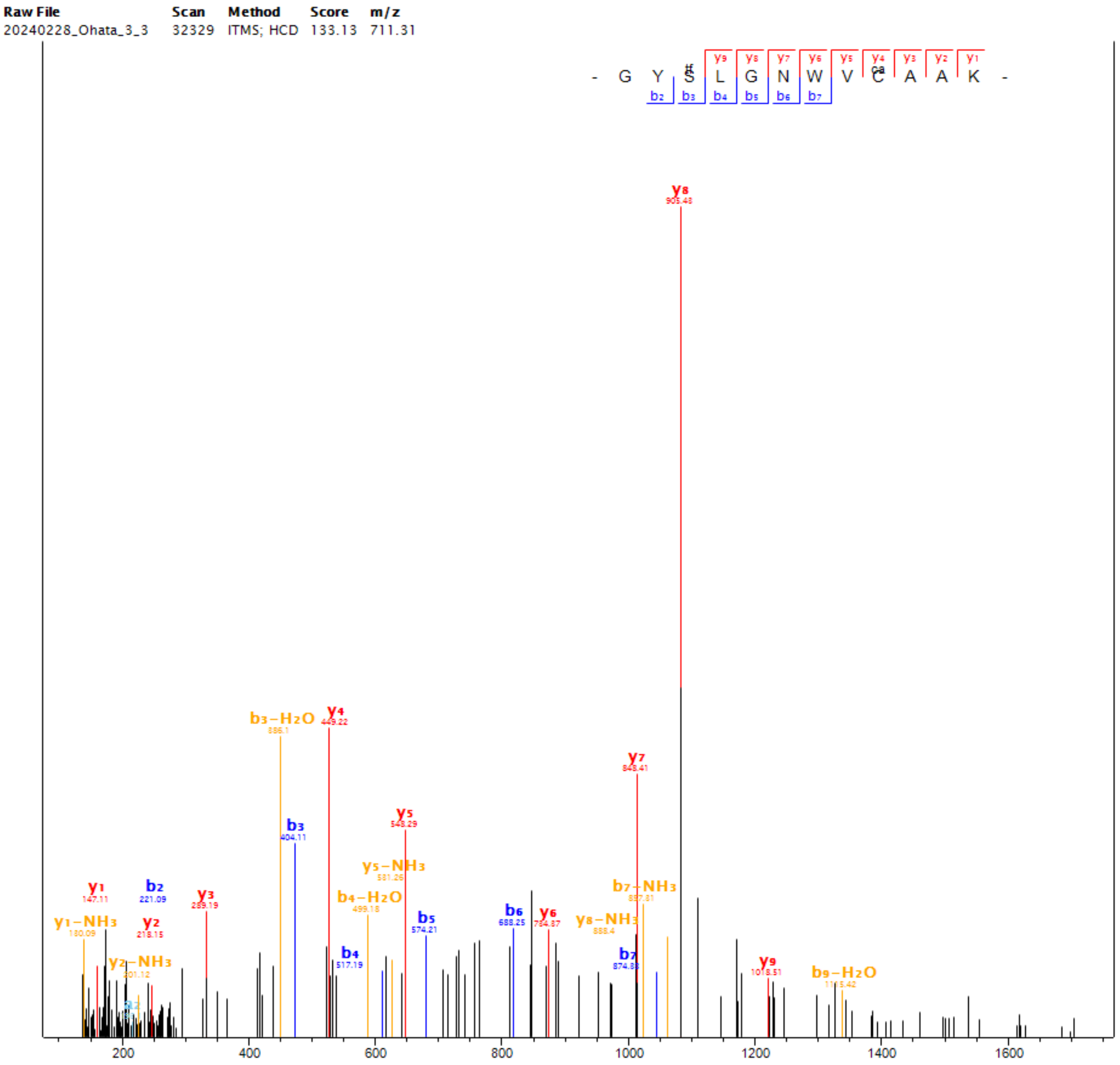

Figure S 70. MS/MS analysis of the peak corresponding to labeled Ser24 of lysozyme (GYS*LGNWVC^{CAM}AAK) as a representative Ser-labeled fragment with TFA.

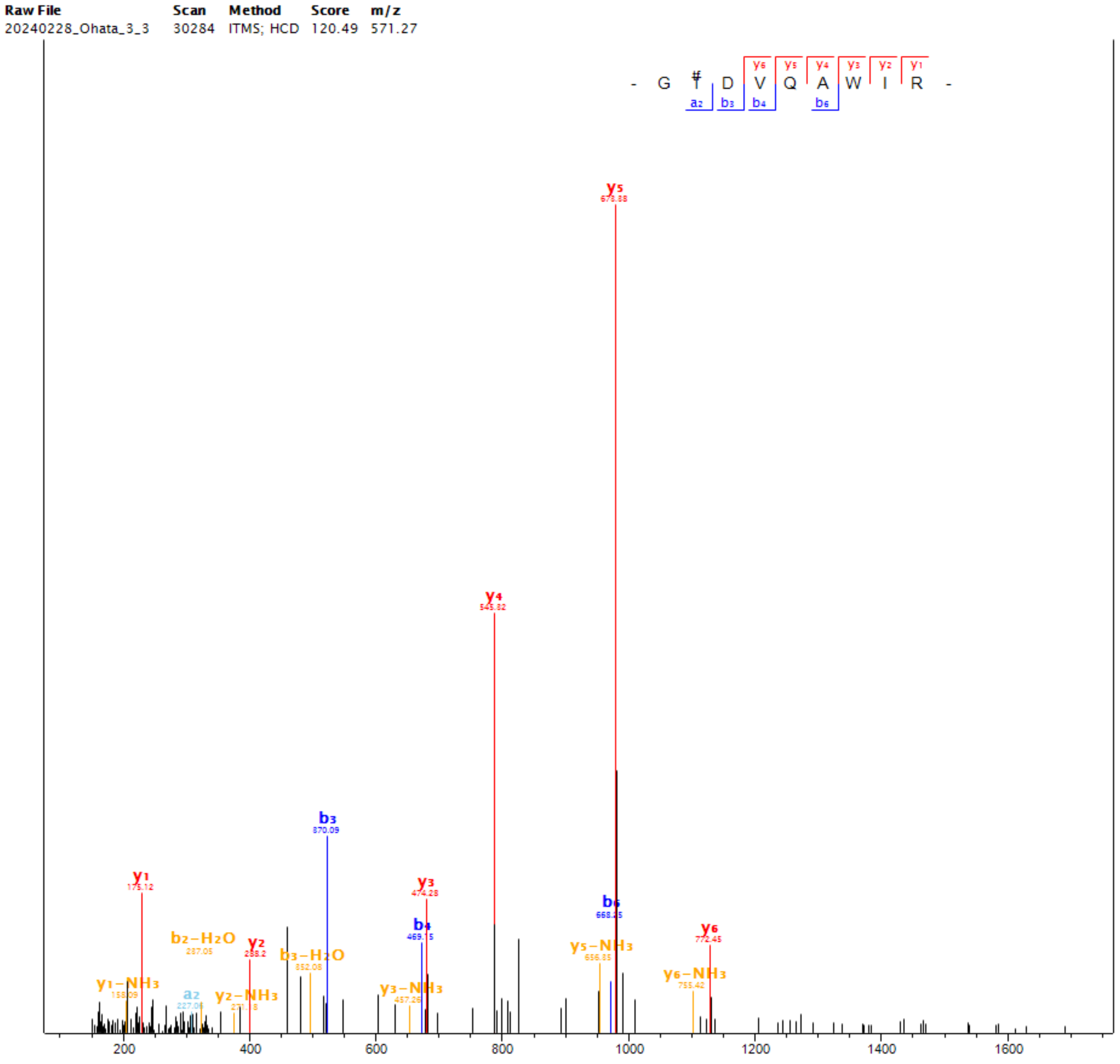

Figure S71. MS/MS analysis of the peak corresponding to labeled Thr118 of lysozyme (GT*DVQAWIR) as a representative Thr-labeled fragment with TFA.

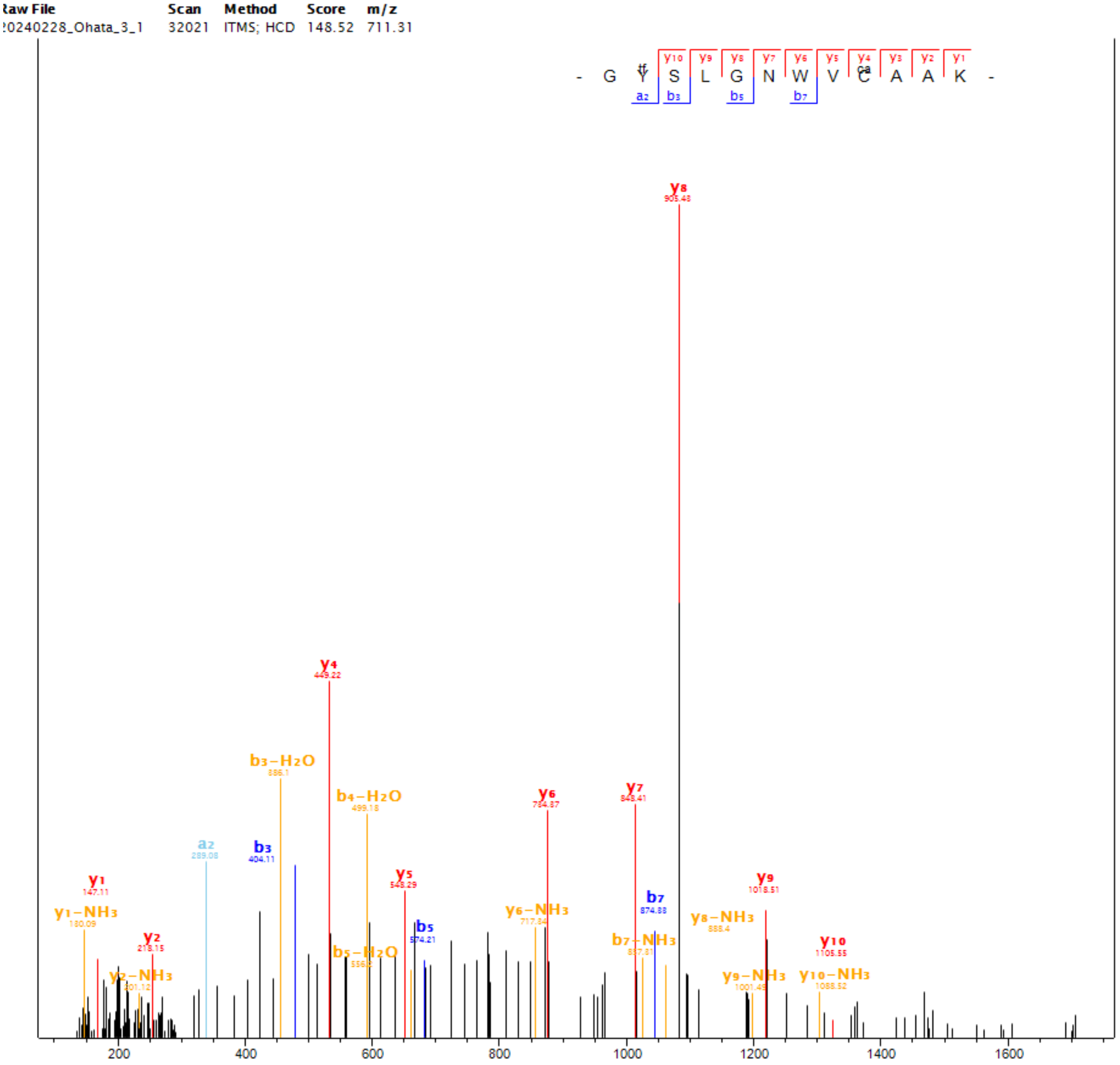

Figure S72. MS/MS analysis of the peak corresponding to labeled Tyr23 of lysozyme (GY*SLGNWVC^{CAM}AAK) as a representative Tyr-labeled fragment with TFA.

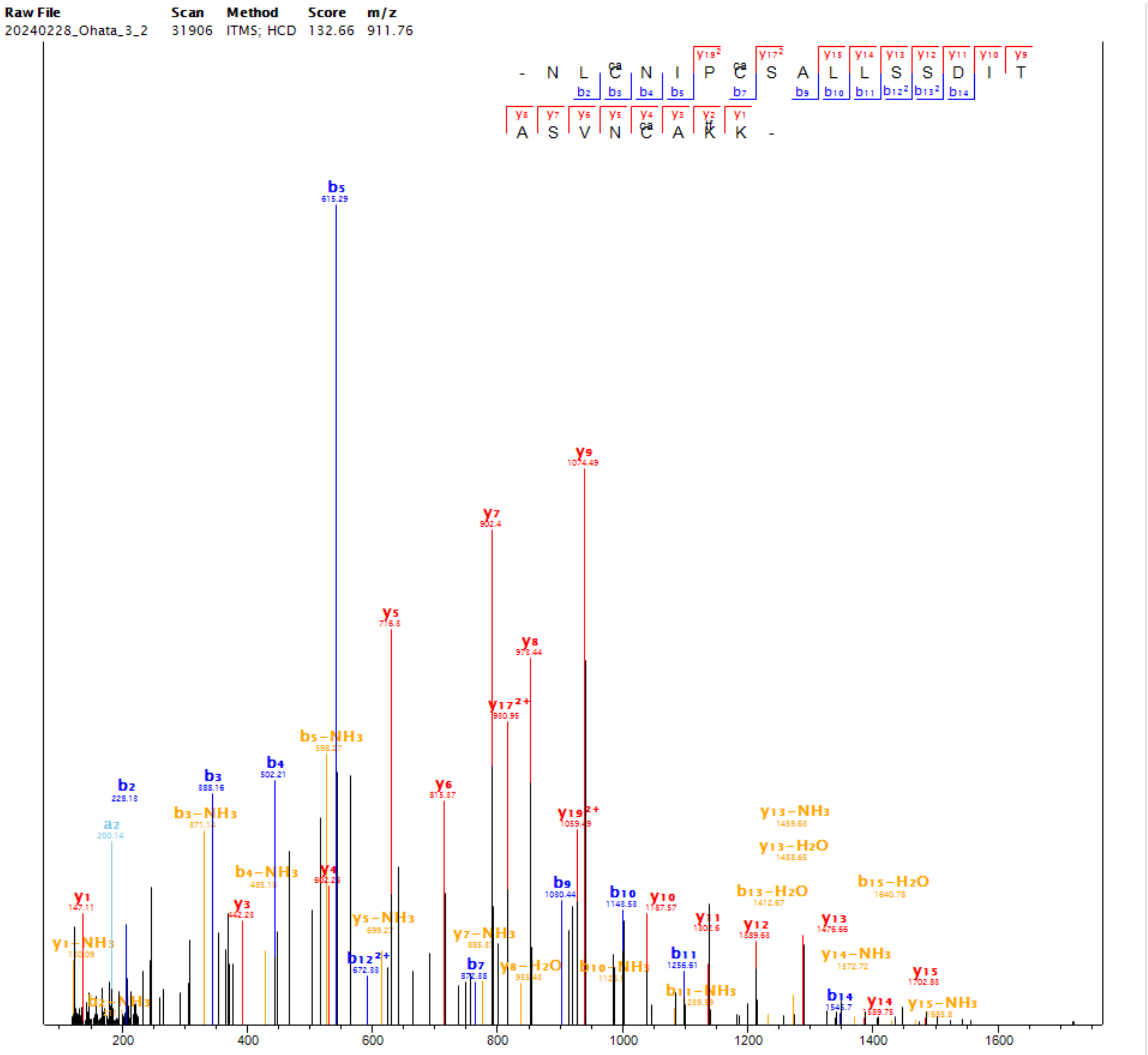

Figure S73. MS/MS analysis of the peak corresponding to labeled Lys96 of lysozyme (NLC^{CAM}NIPC^{CAM}SALLSSDITASVNC^{CAM}AK*K) as a representative Lys-labeled fragment with TFA.

Proteomics analysis of α-chymotrypsinogen A modified with 4-pentynoic acid

Uniprot ID: P00766

Sequence

CGVPAIQPVLSGLSRIVNGEEAVPGSWPWQVSLQDKTGFHFCGGSLINENWVVTAAHCGV
TTSDVVVAGEFDQGSSSEKIQKLKIAKVFKNSKYNSLTINNDITLLKLSTAASFSQTVSA
VCLPSASDDFAAGTTCVTTGWGLTRYTNANTPDRLQQASLPLLSNTNCKKYWGTKIKDAM
ICAGASGVSSCMGDSGGPLVCKKNGAWTLVGIVSWGSSTCSTSTPGVYARVTALVNWVQQ
TLAAN

Sequence

CGVPAIQPVLSGLSRIVNGEEAVPGSWPWQVSLQDKTGFHFCGGSLINENWVVTAAHCGV
TTSDVVVAGEFDQGSSSEKIQKLKIAKVFKNSKYNSLTINNDITLLKLSTAASFSQTVSA
VCLPSASDDFAAGTTCVTTGWGLTRYTNANTPDRLQQASLPLLSNTNCKKYWGTKIKDAM
ICAGASGVSSCMGDSGGPLVCKKNGAWTLVGIVSWGSSTCSTSTPGVYARVTALVNWVQQ
TLAAN

Coverage 98.8%

Modification site of **4-pentynoic acid**

Modification site of **TFA**

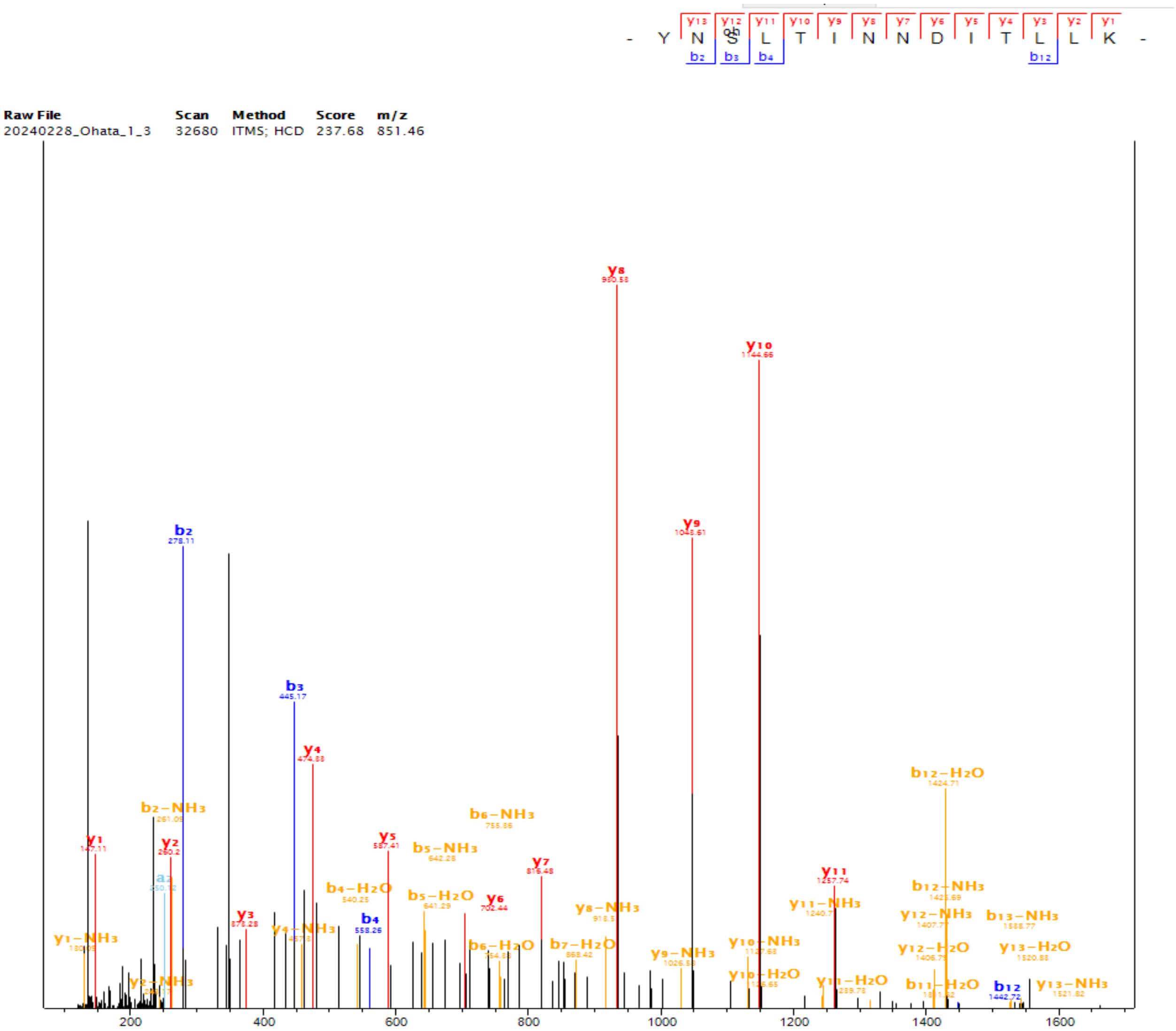

Figure S74. MS/MS analysis of the peak corresponding to labeled Ser96 of alpha-chymotrypsinogen A (YNS*LTINNDITLLK) as a representative Ser-labeled fragment with 4-pentynoic acid.

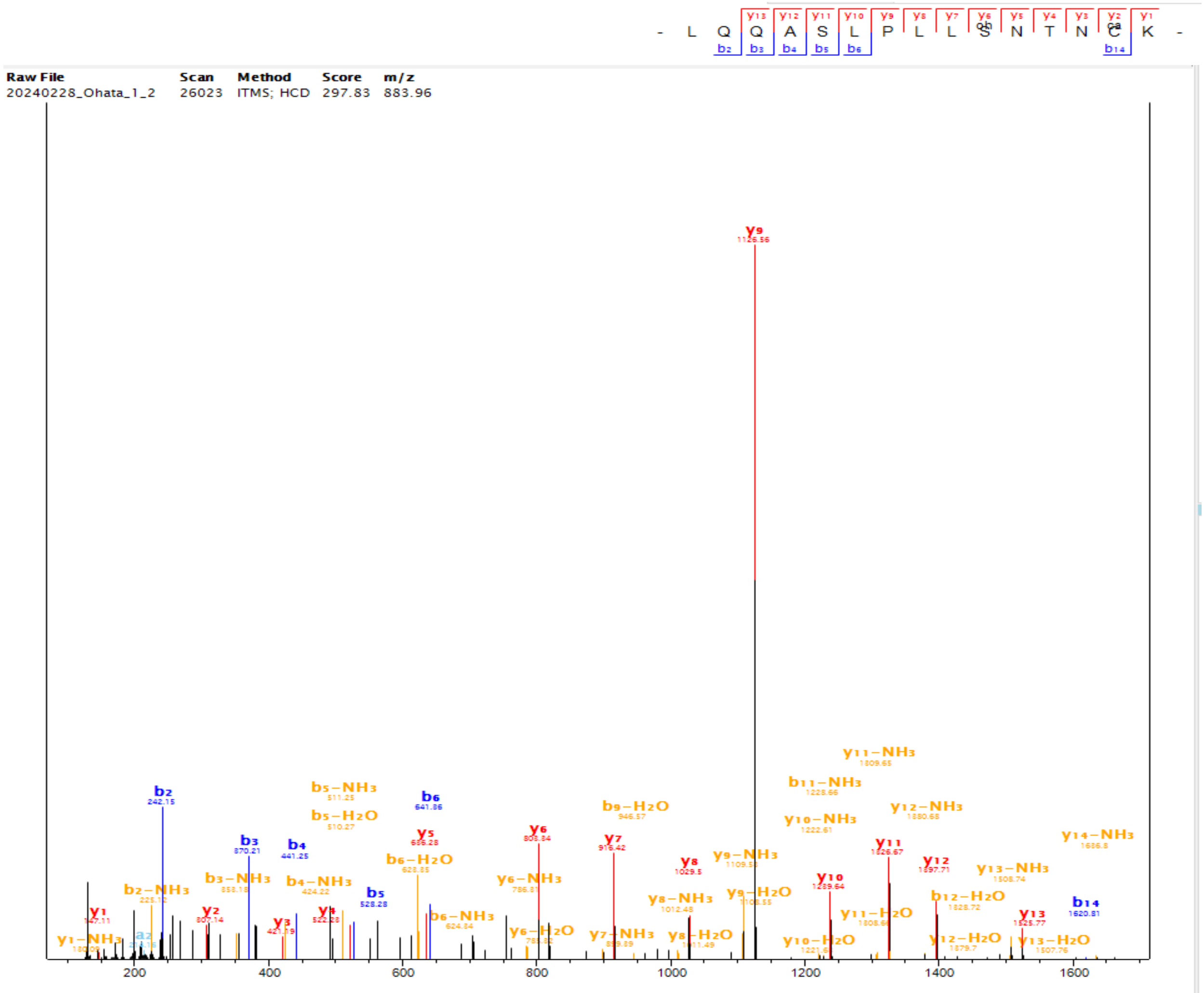

Figure S75. MS/MS analysis of the peak corresponding to labeled Ser164 of alpha-chymotrypsinogen A (LQQASLPLLS*NTNC^{CAM}K) as a representative Ser-labeled fragment with 4-pentynoic acid.

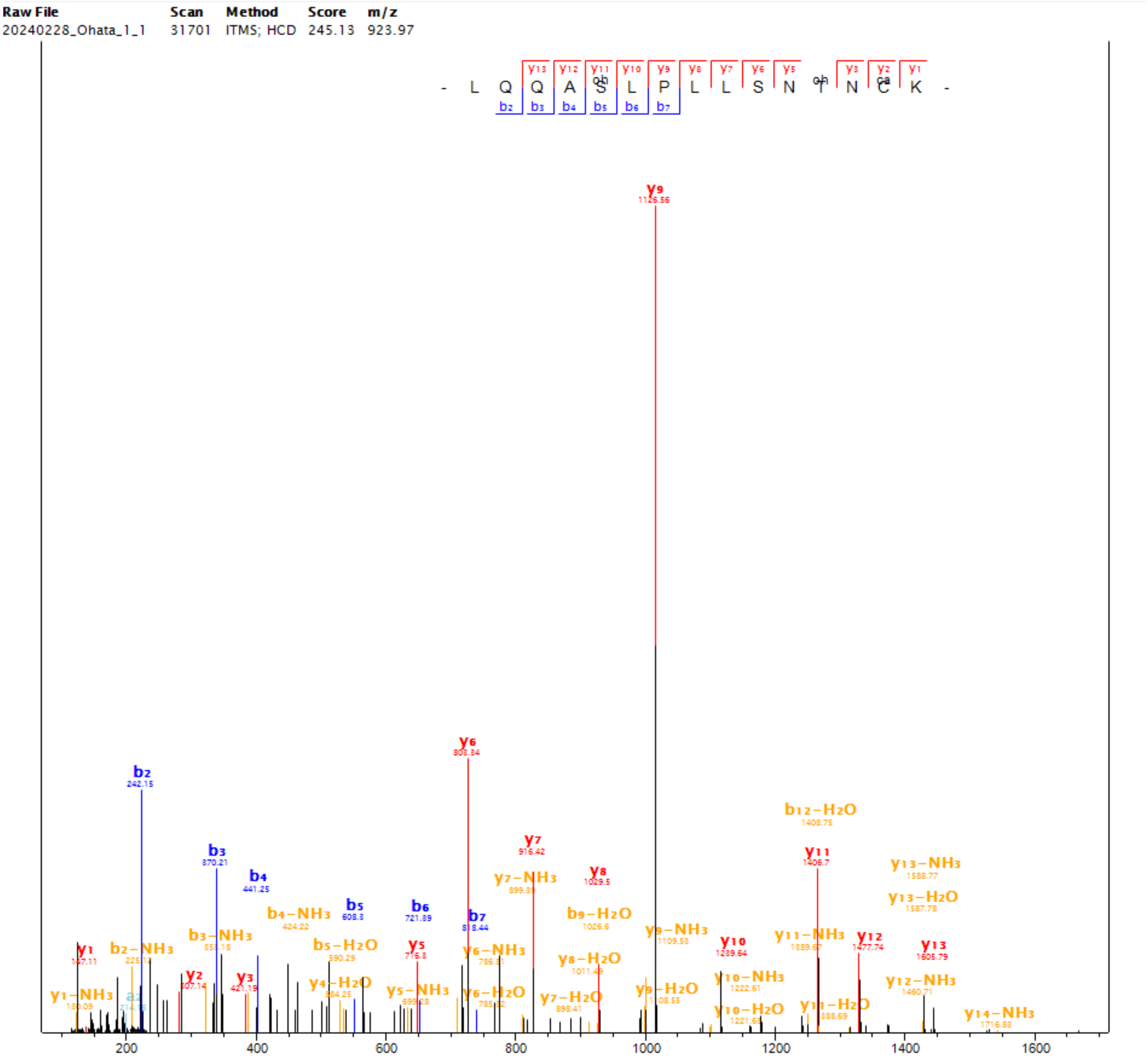

Figure S76. MS/MS analysis of the peak corresponding to labeled Ser159 and Thr166 of alpha-chymotrypsinogen A (LQQAS*LPLLSNT*NC^{CAM}K) as a representative Ser- and Thr-labeled fragment with 4-pentynoic acid.

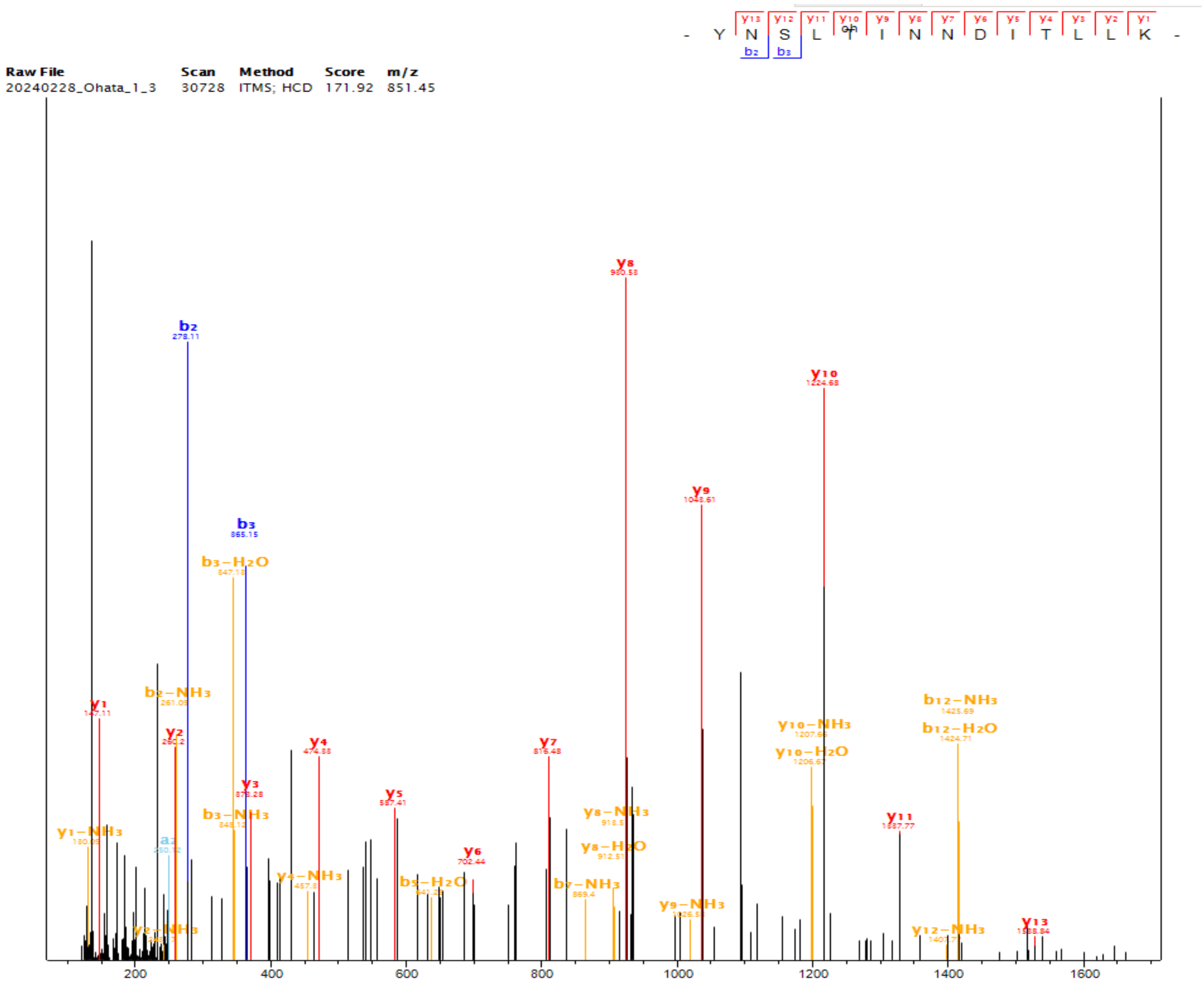

Figure S77. MS/MS analysis of the peak corresponding to labeled Thr98 of alpha-chymotrypsinogen A (YNSLT*INNDITLLK) as a representative Thr-labeled fragment with 4-pentynoic acid.

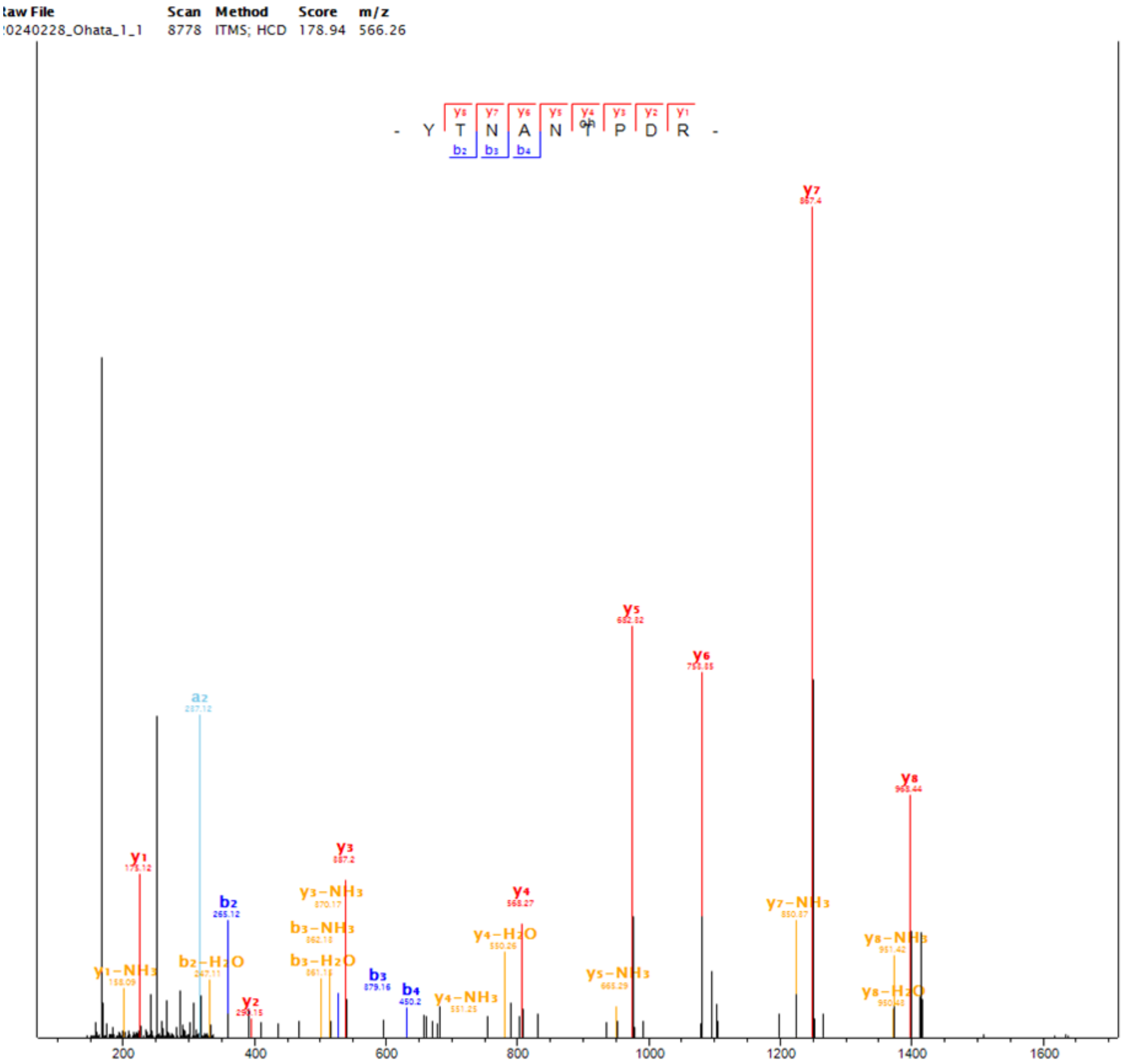

Figure S78. MS/MS analysis of the peak corresponding to labeled Thr151 of alpha-chymotrypsinogen A (YTNANT*PDR) as a representative Thr-labeled fragment with 4-pentynoic acid.

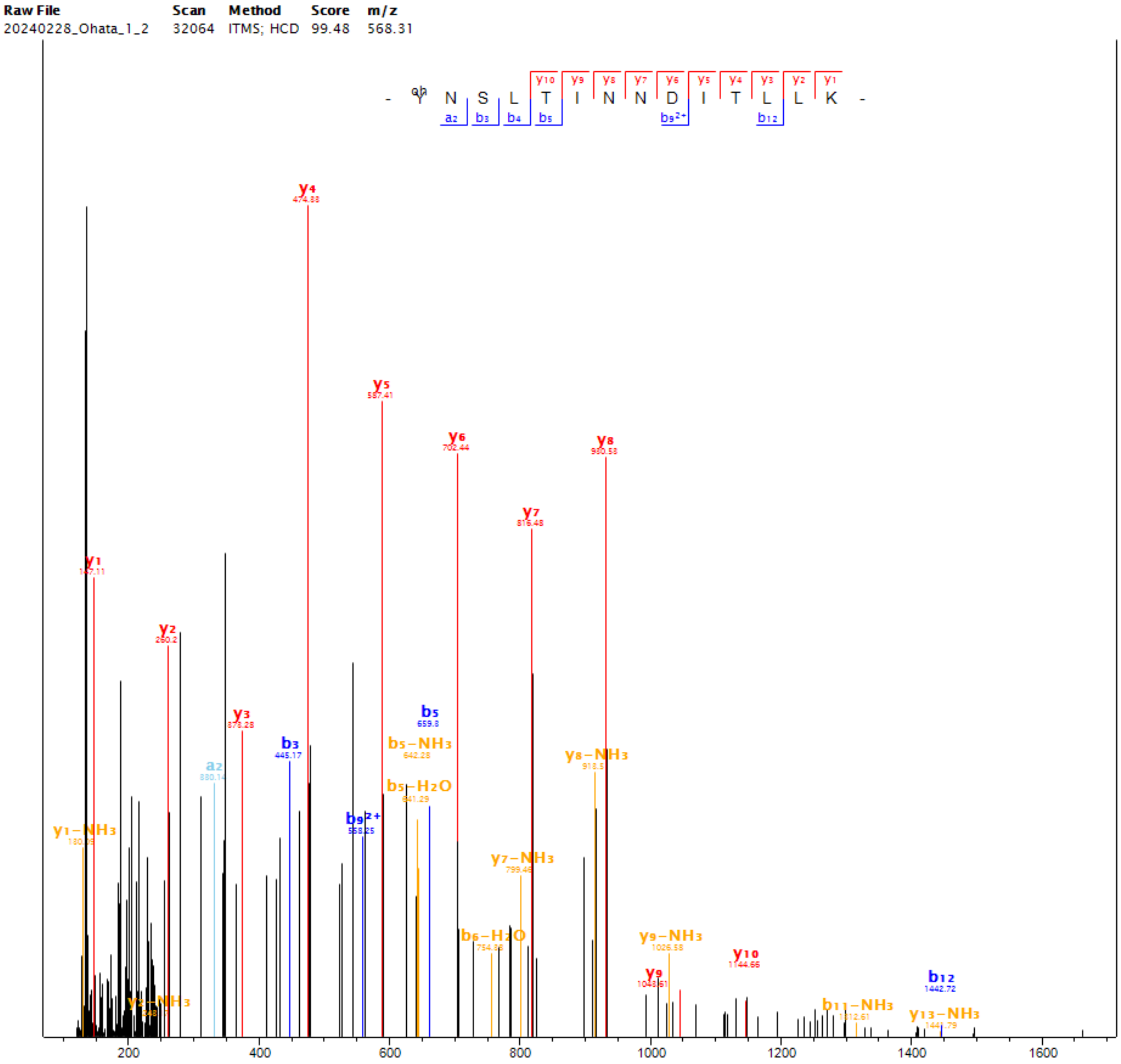

Figure S79. MS/MS analysis of the peak corresponding to labeled Tyr94 of alpha-chymotrypsinogen A (Y*NSLTINNDITLLK) as a representative Tyr-labeled fragment with 4-pentynoic acid.

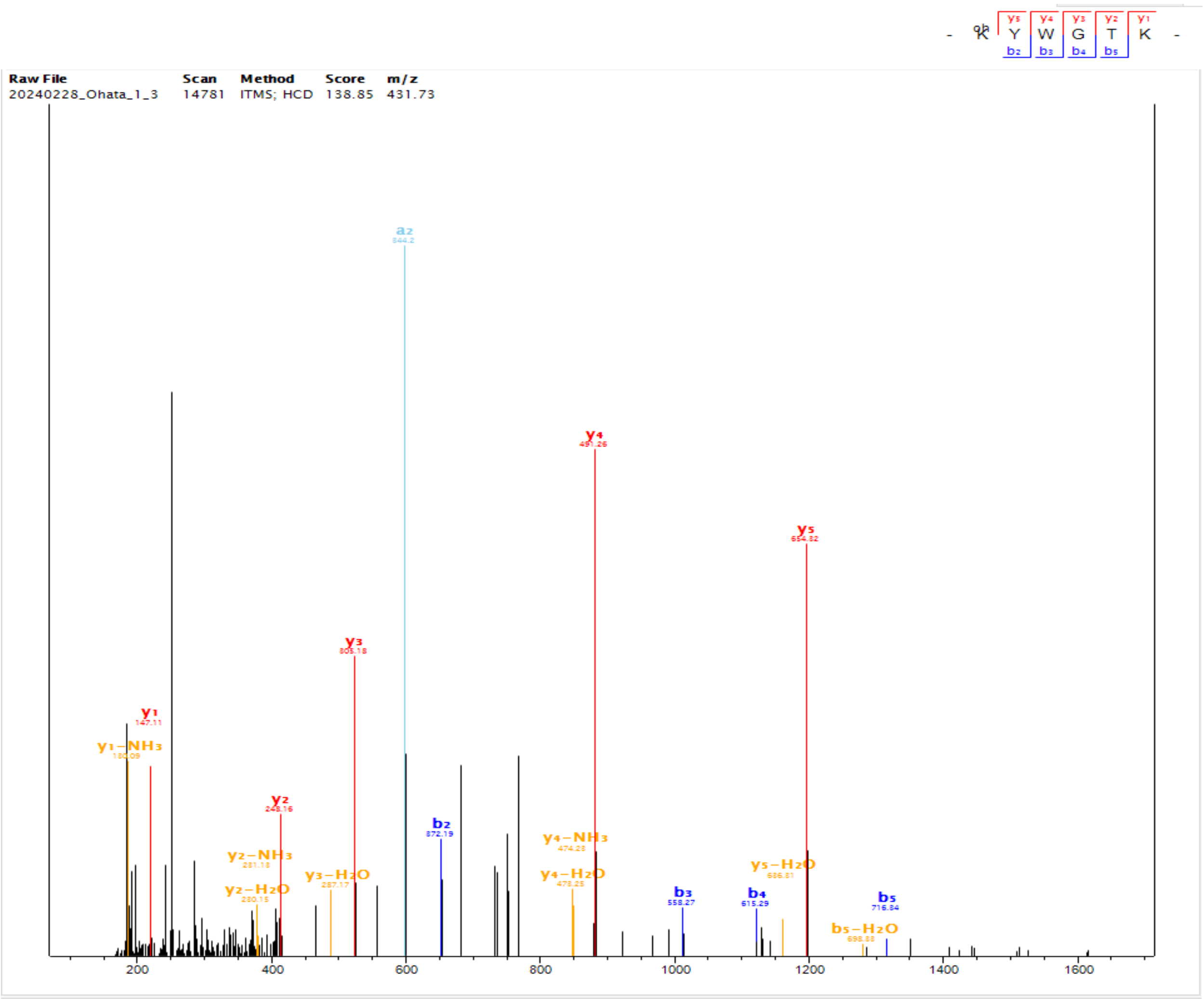

Figure S80. MS/MS analysis of the peak corresponding to labeled Lys170 of alpha-chymotrypsinogen A (K*YWGTK) as a representative Lys-labeled fragment with 4-pentynoic acid.

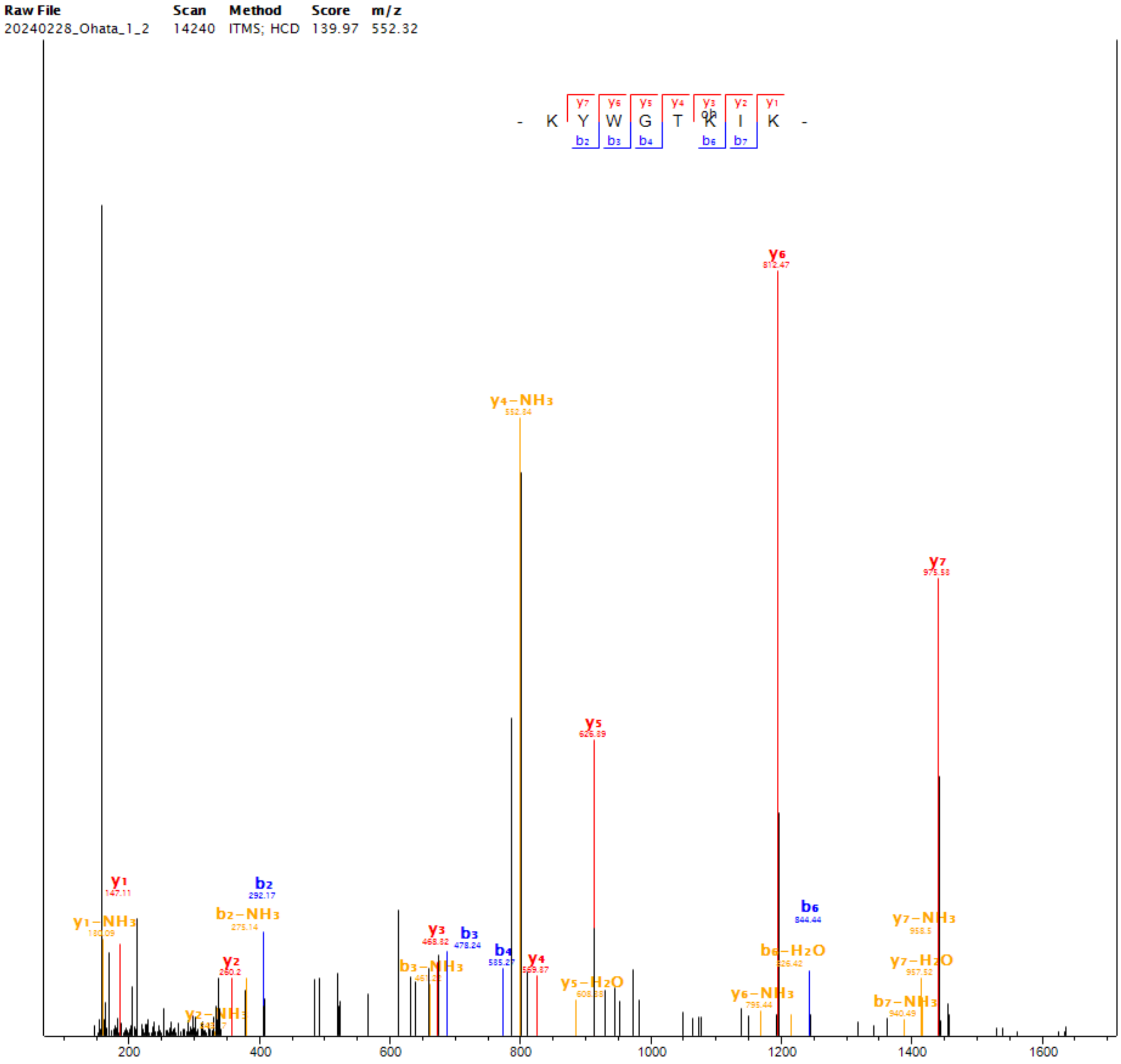

Figure S81. MS/MS analysis of the peak corresponding to labeled Lys175 of alpha-chymotrypsinogen A (KYWGTK*IK) as a representative Lys-labeled fragment with 4-pentynoic acid.

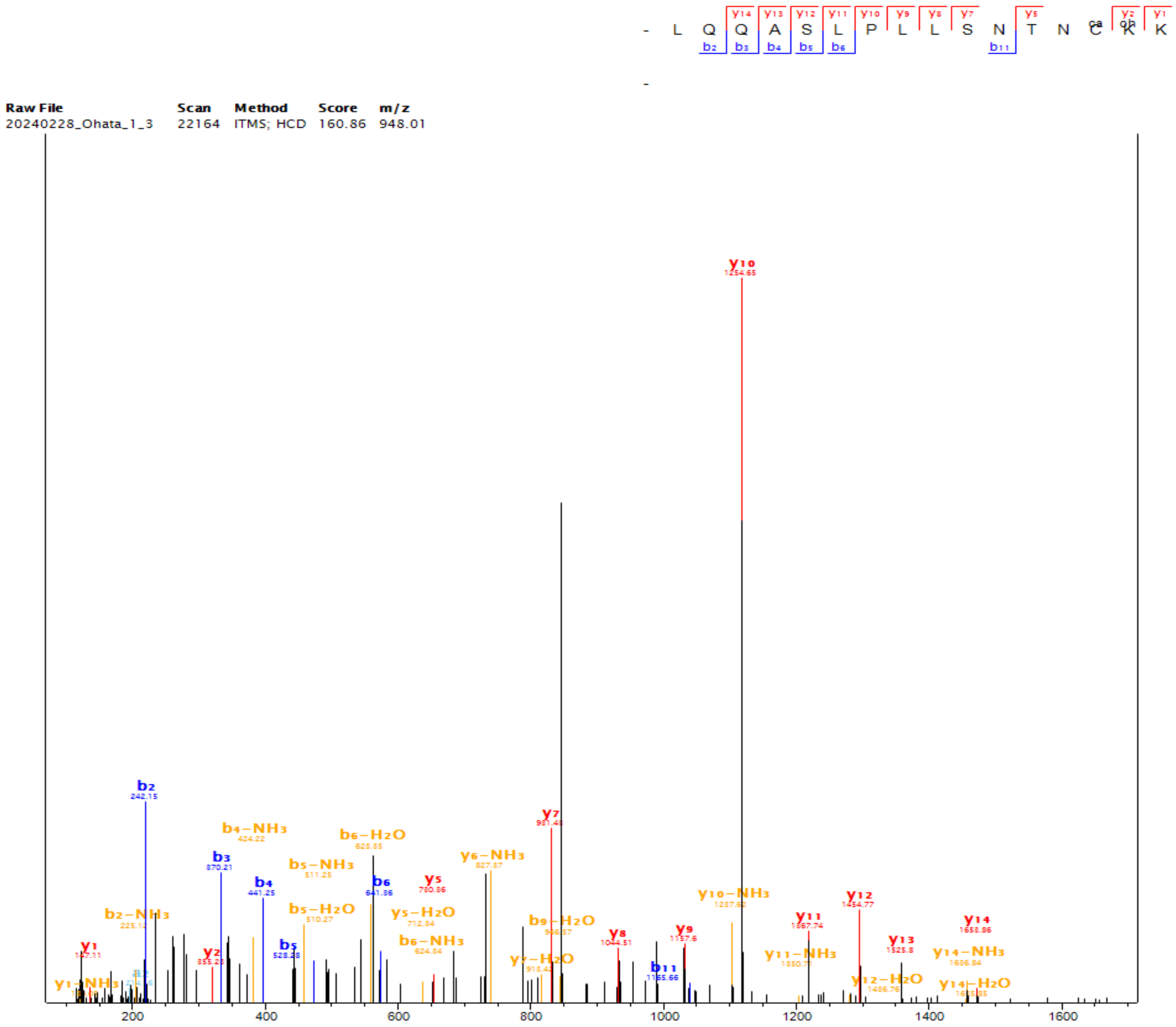

Figure S82. MS/MS analysis of the peak corresponding to labeled Lys169 of alpha-chymotrypsinogen A (LQQASLPLLSNTNC^{CAM}K*K) as a representative Lys-labeled fragment with 4-pentynoic acid.

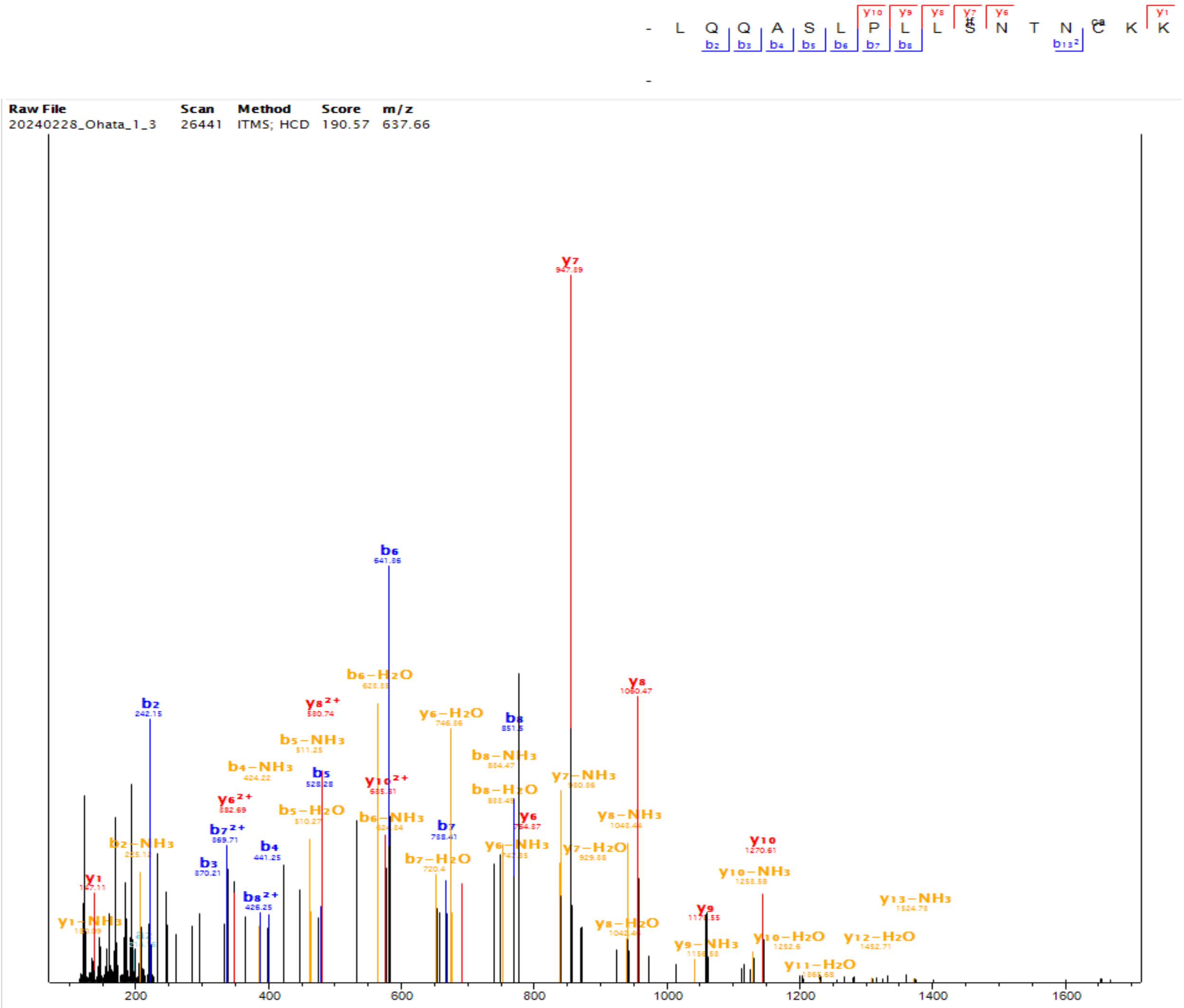

Figure S83. MS/MS analysis of the peak corresponding to labeled Ser164 of alpha-chymotrypsinogen A (LQQASLPLLS*NTNC^{CAM}KK) as a representative Ser-labeled fragment with TFA.

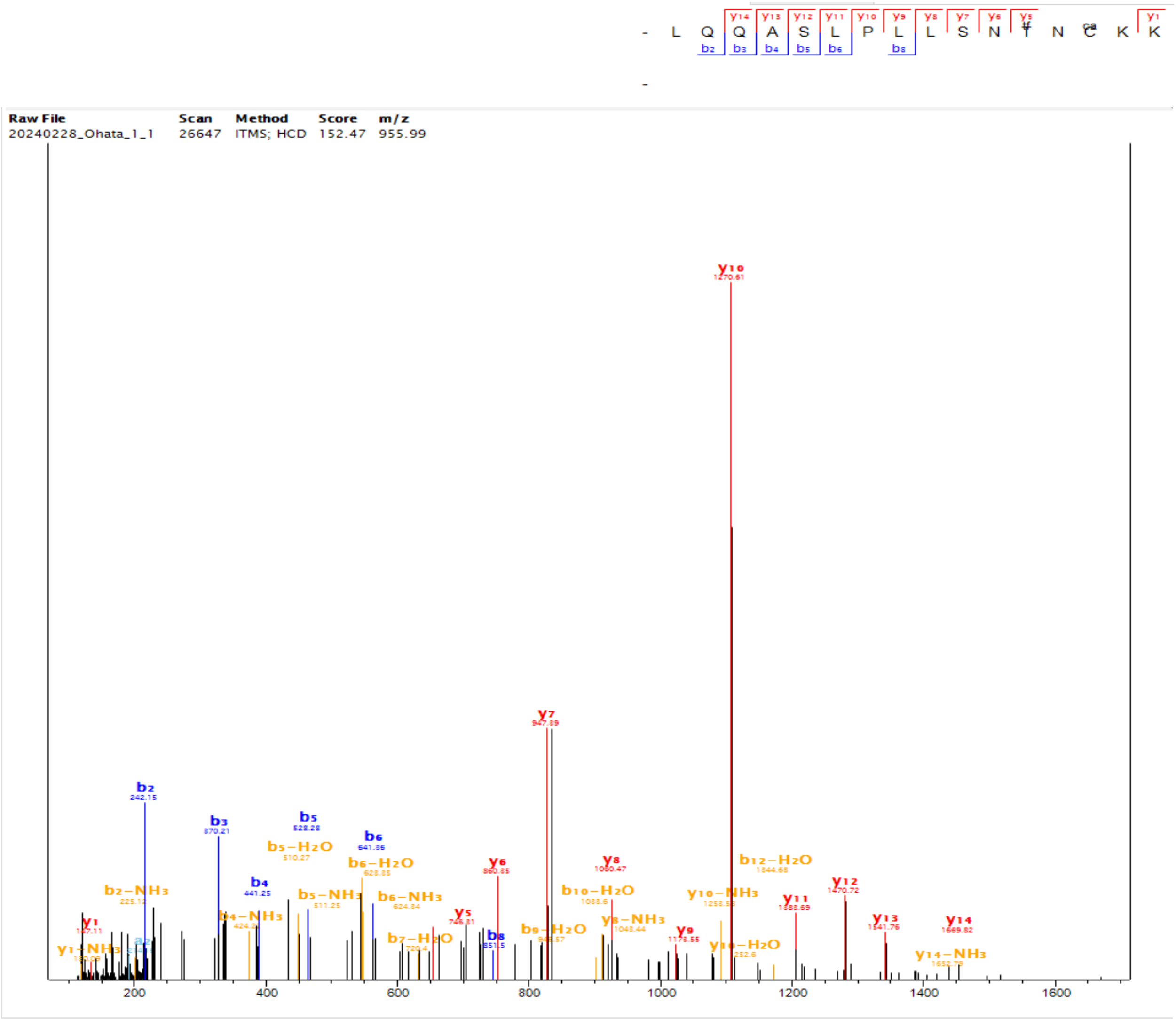

Figure S84. MS/MS analysis of the peak corresponding to labeled Thr166 of alpha-chymotrypsinogen A (LQQASLPLLSNT*NC^{CAM}KK) as a representative Thr-labeled fragment with TFA.

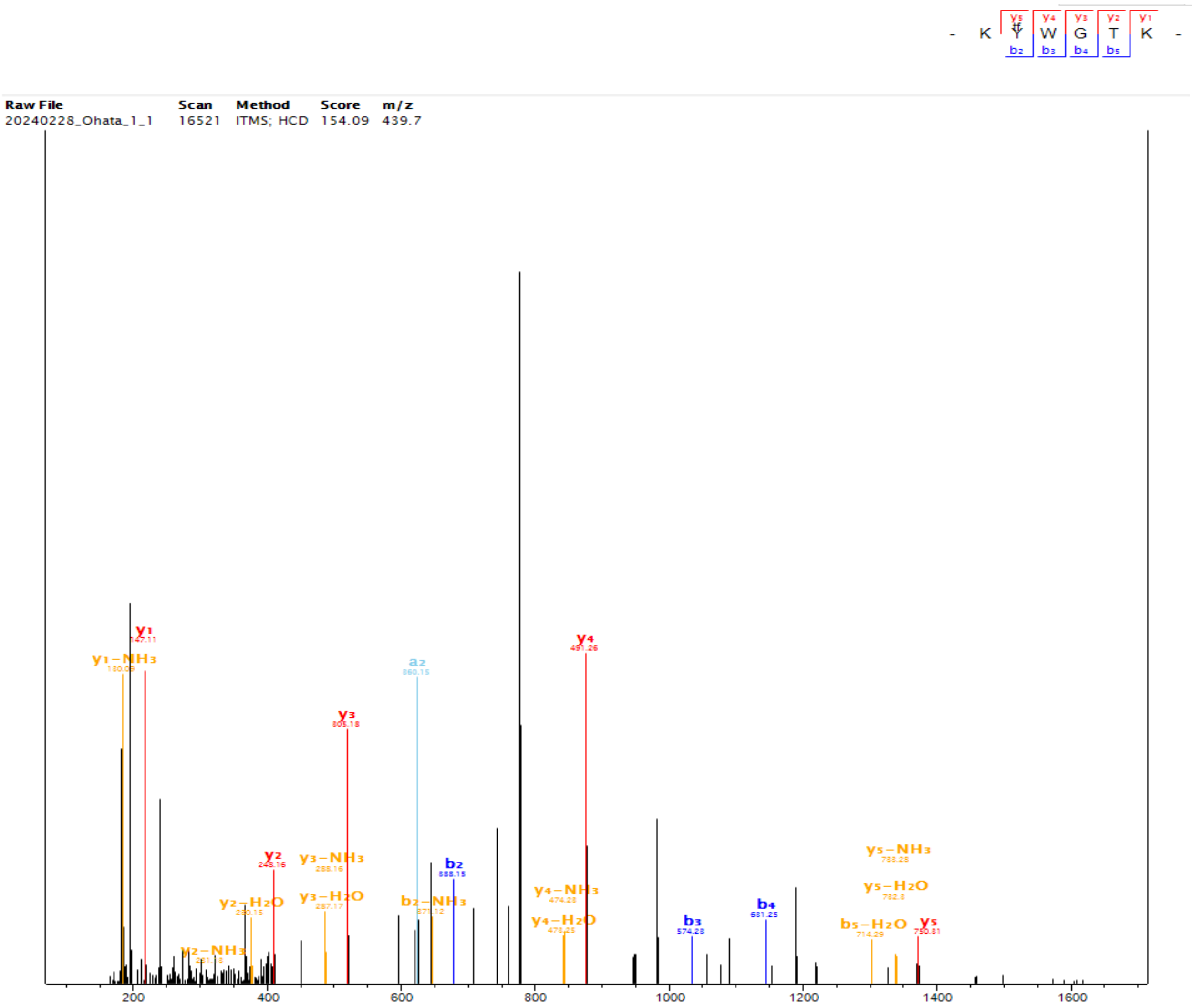

Figure S85. MS/MS analysis of the peak corresponding to labeled Tyr171 of alpha-chymotrypsinogen A (KY*WGTK) as a representative Tyr-labeled fragment with TFA.

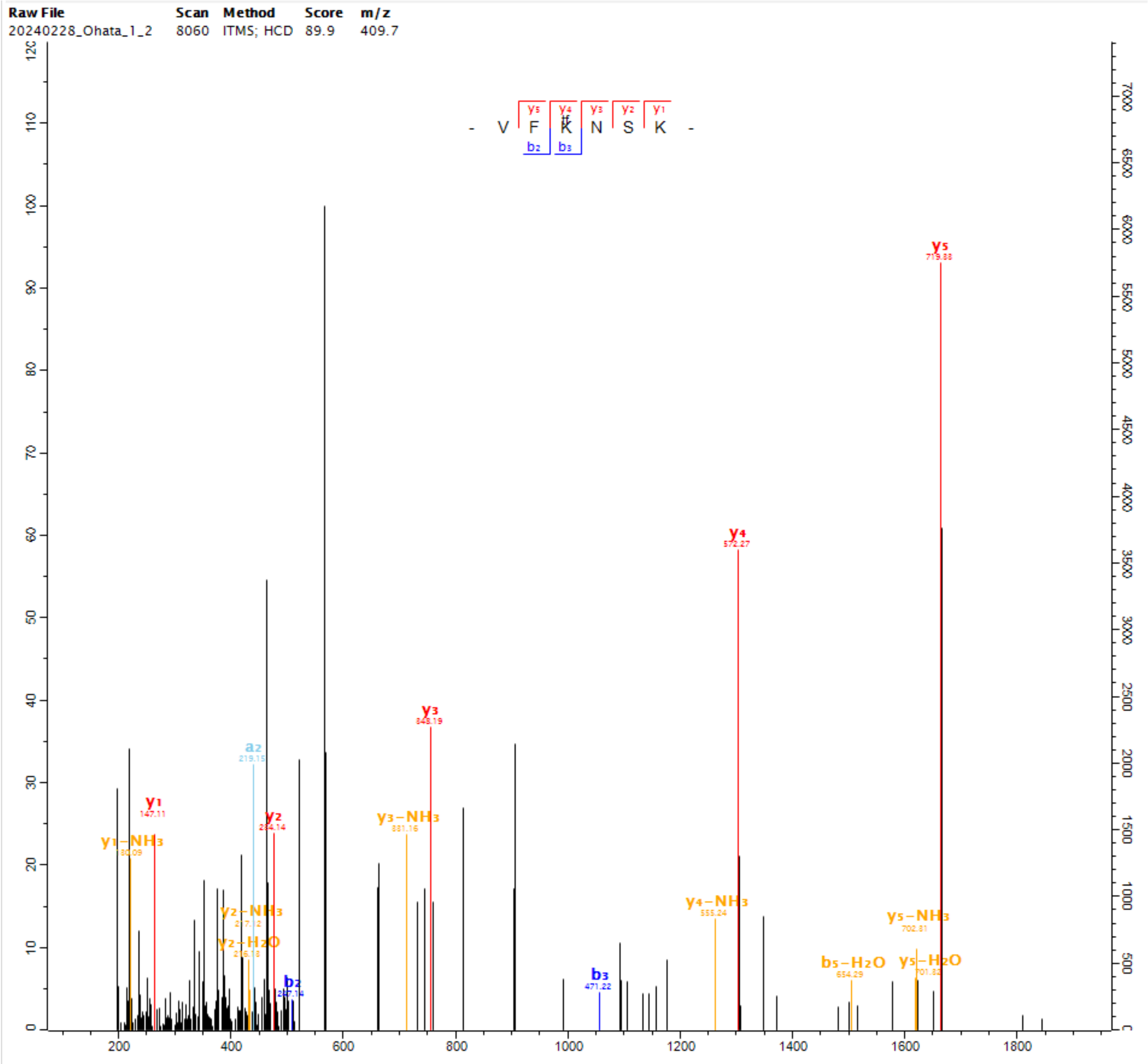

Figure S86. MS/MS analysis of the peak corresponding to labeled Lys90 of alpha-chymotrypsinogen A (VFK*NSK) as a representative Lys-labeled fragment with TFA.

Proteomics analysis of ribonuclease A modified with 4-pentynoic acid

Uniprot ID: P61823

Sequence
KETAAAKFERQHMDSSTSAASSSNYCNQMMKSRNLTKDRCKPVNTFVHESLADVQAVCSQKNVACKNGQTNCYQSYSTM
SITDCRETGSSKYPNCAYKTTQANKHIIVACEGNPYVPVHFDASV

Sequence
KETAAAKFERQHMDSSTSAASSSNYCNQMMKSRNLTKDRCKPVNTFVHESLADVQAVCSQKNVACKNGQTNCYQSYSTM
SITDCRETGSSKYPNCAYKTTQANKHIIVACEGNPYVPVHFDASV

Coverage 100%
Modification site of **4-pentynoic acid**

Modification site of **TFA**
The addition peak of TFA to ribonuclease A had a weak signal for any residue and did not yield reliable MS/MS results.

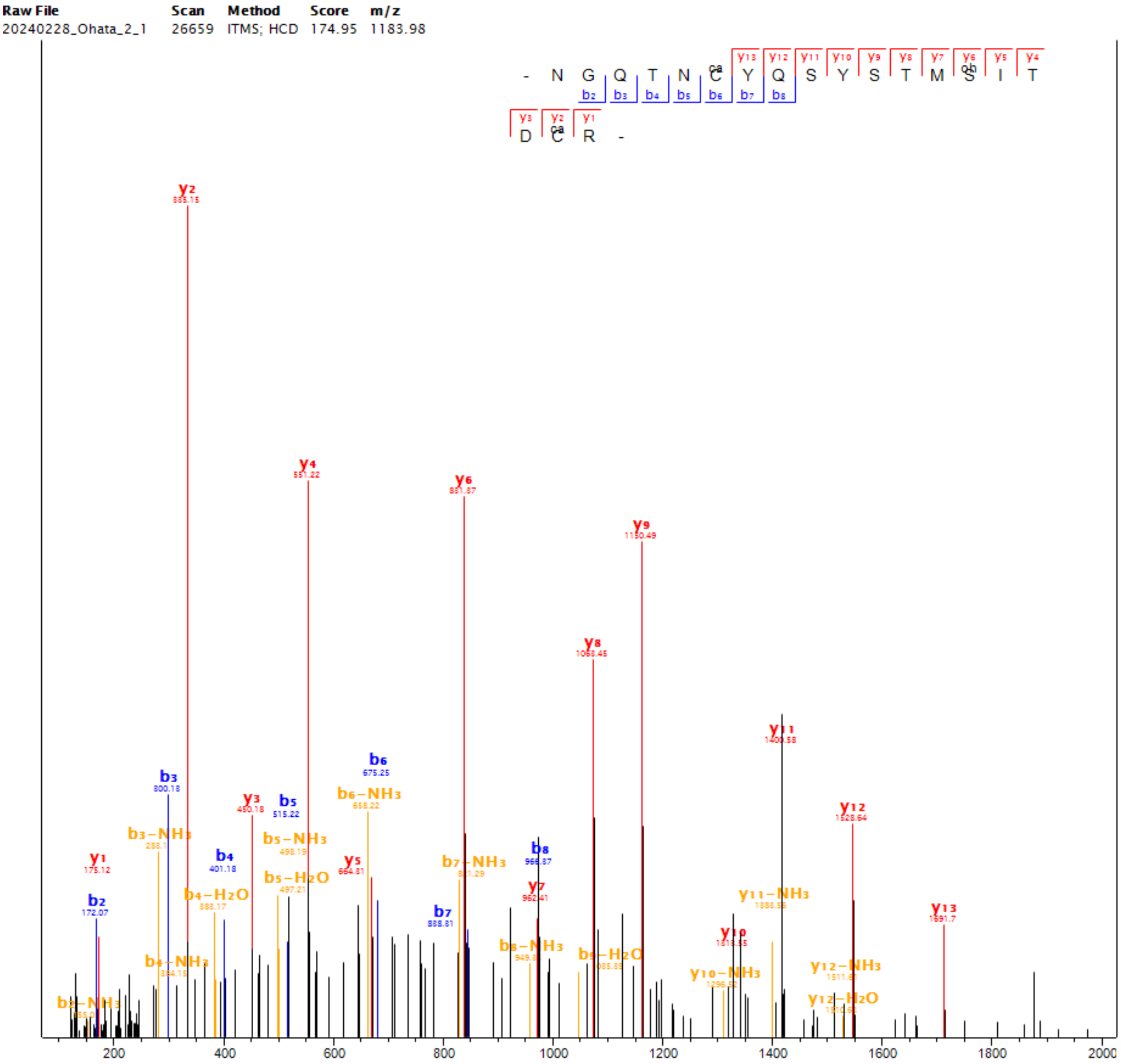

Figure S87. MS/MS analysis of the peak corresponding to labeled Ser80 of ribonuclease A (NGQTNC^{CAM}YQSYSTMS*ITDC^{CAM}R) as a representative Ser-labeled fragment with 4-pentynoic acid.

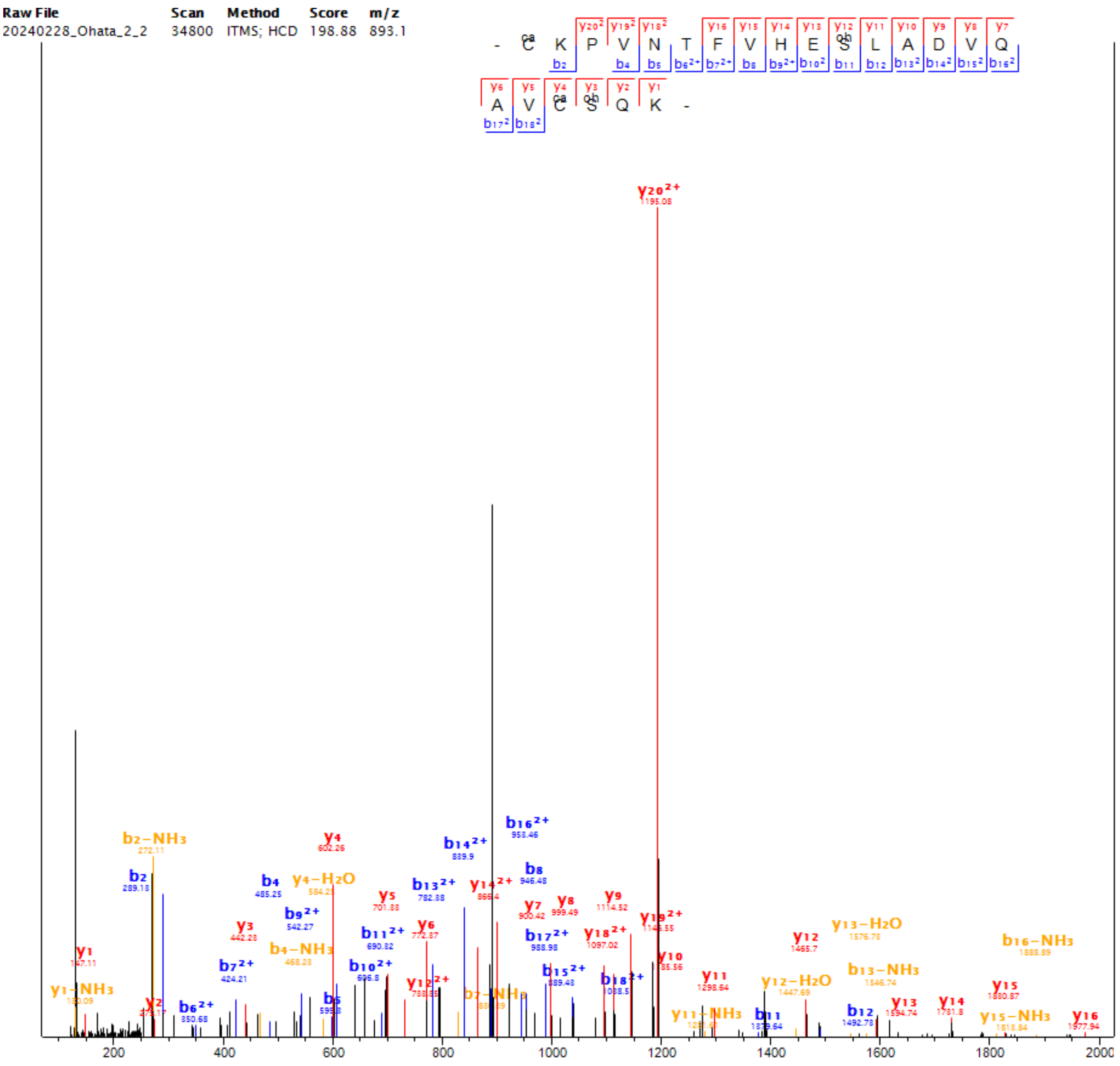

Figure S88. MS/MS analysis of the peak corresponding to labeled Ser50 and Ser59 of ribonuclease A (C^{CAM}KPVNTFVHES*LADVQAVC^{CAM}S*QK) as a representative Ser-labeled fragment with 4-pentynoic acid.

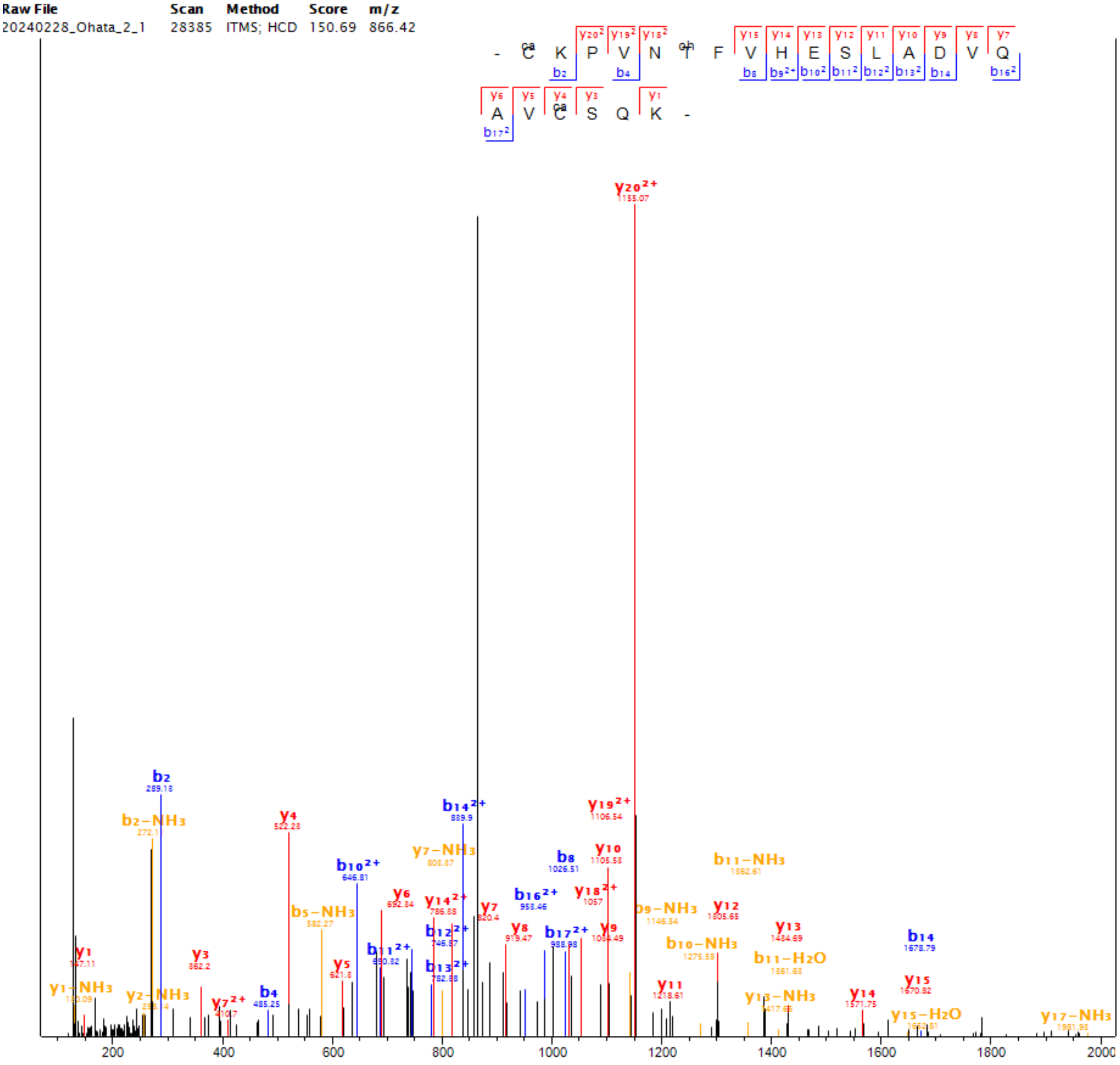

Figure S89. MS/MS analysis of the peak corresponding to labeled Thr45 of ribonuclease A (C^{CAM}KPVNT*FVHES*LADVQAVC^{CAM}SQK) as a representative Thr-labeled fragment with 4-pentynoic acid.

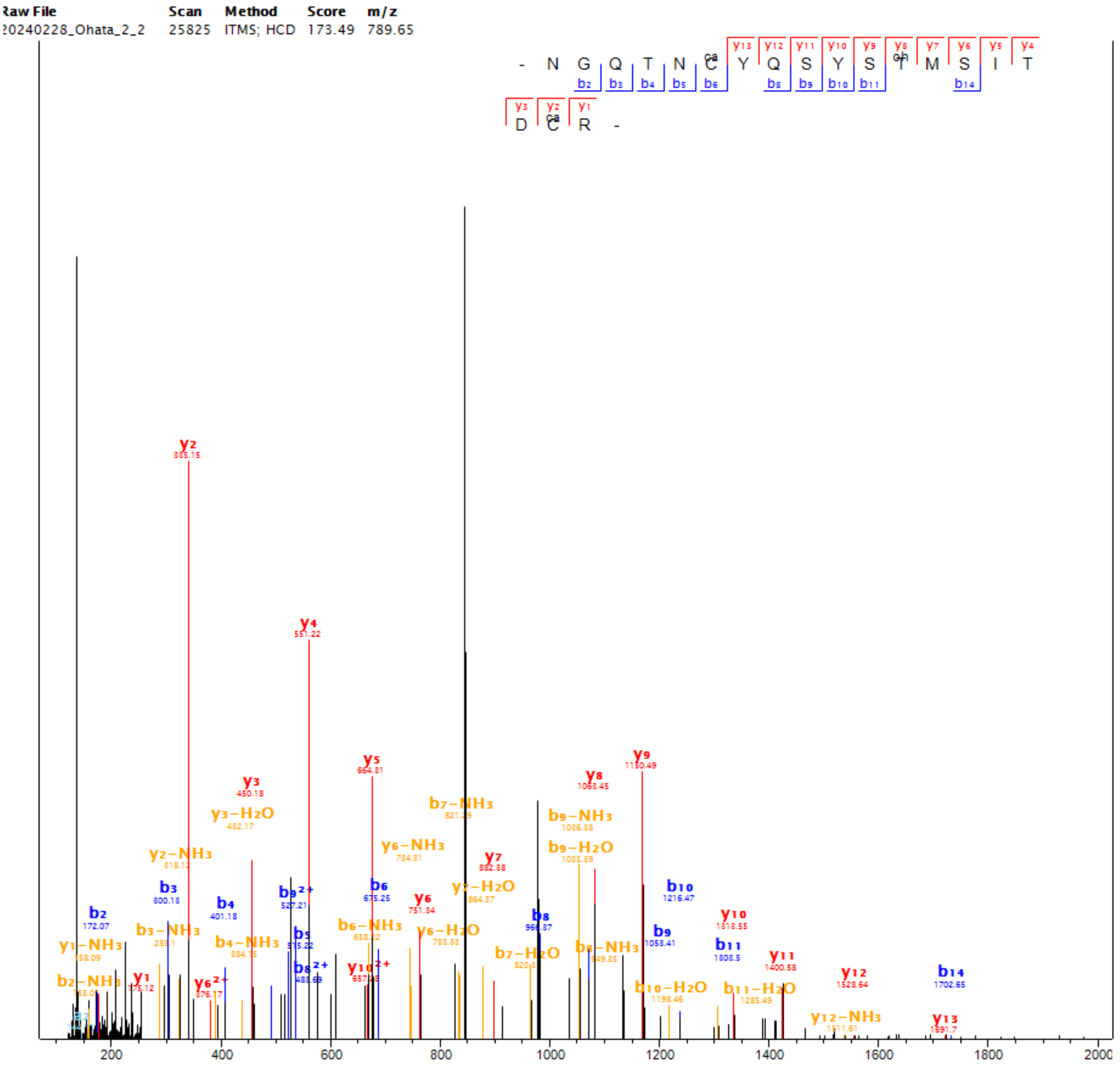

Figure S90. MS/MS analysis of the peak corresponding to labeled Thr78 of ribonuclease A (NGQTNC^{CAM}YQSYST*MSITDC^{CAM}R) as a representative Thr-labeled fragment with 4-pentynoic acid.

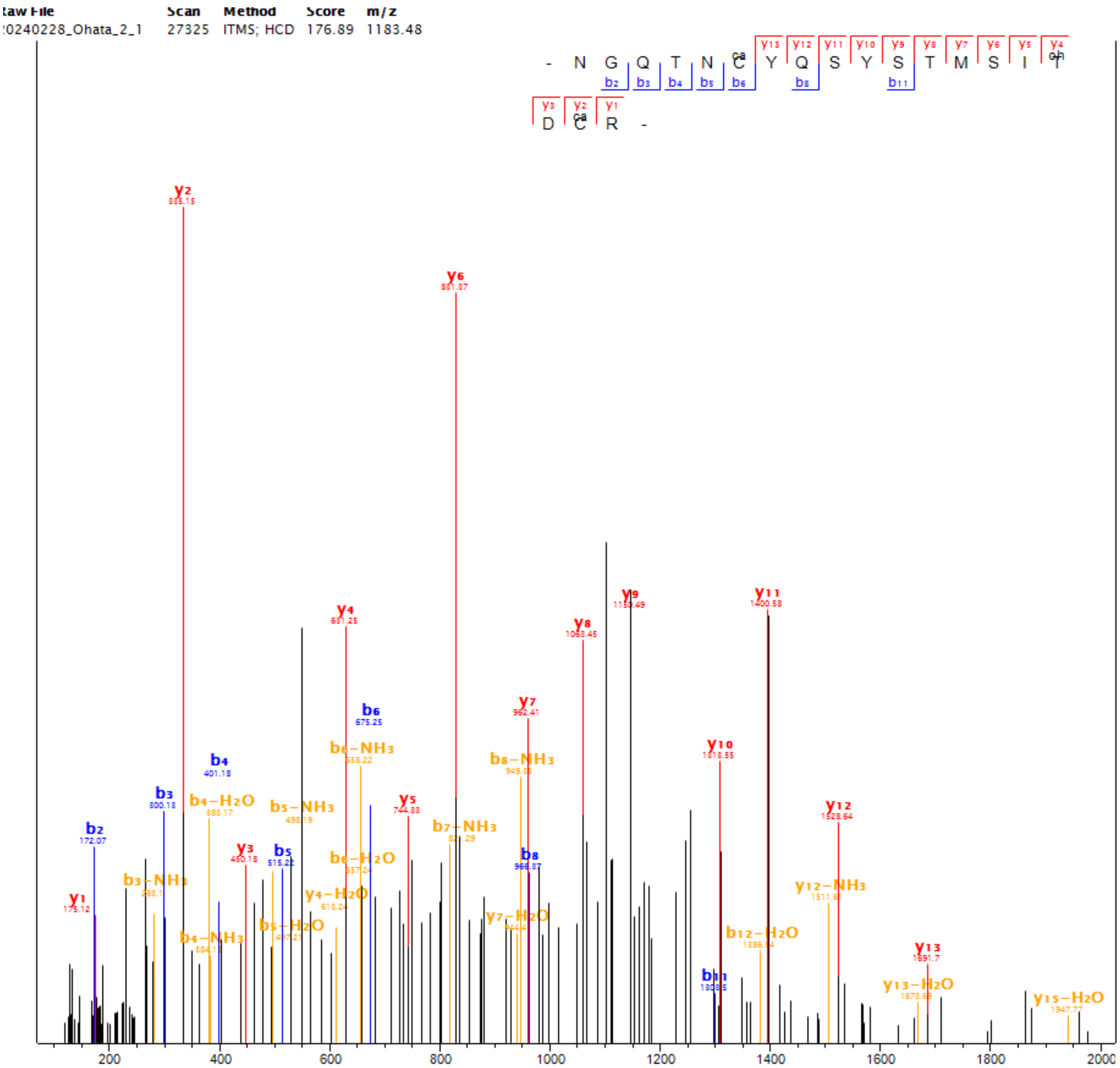

Figure S91. MS/MS analysis of the peak corresponding to labeled Thr82 of ribonuclease A (NGQTNC^{CAM}YQSYSTMSIT*DC^{CAM}R) as a representative Thr-labeled fragment with 4-pentynoic acid.

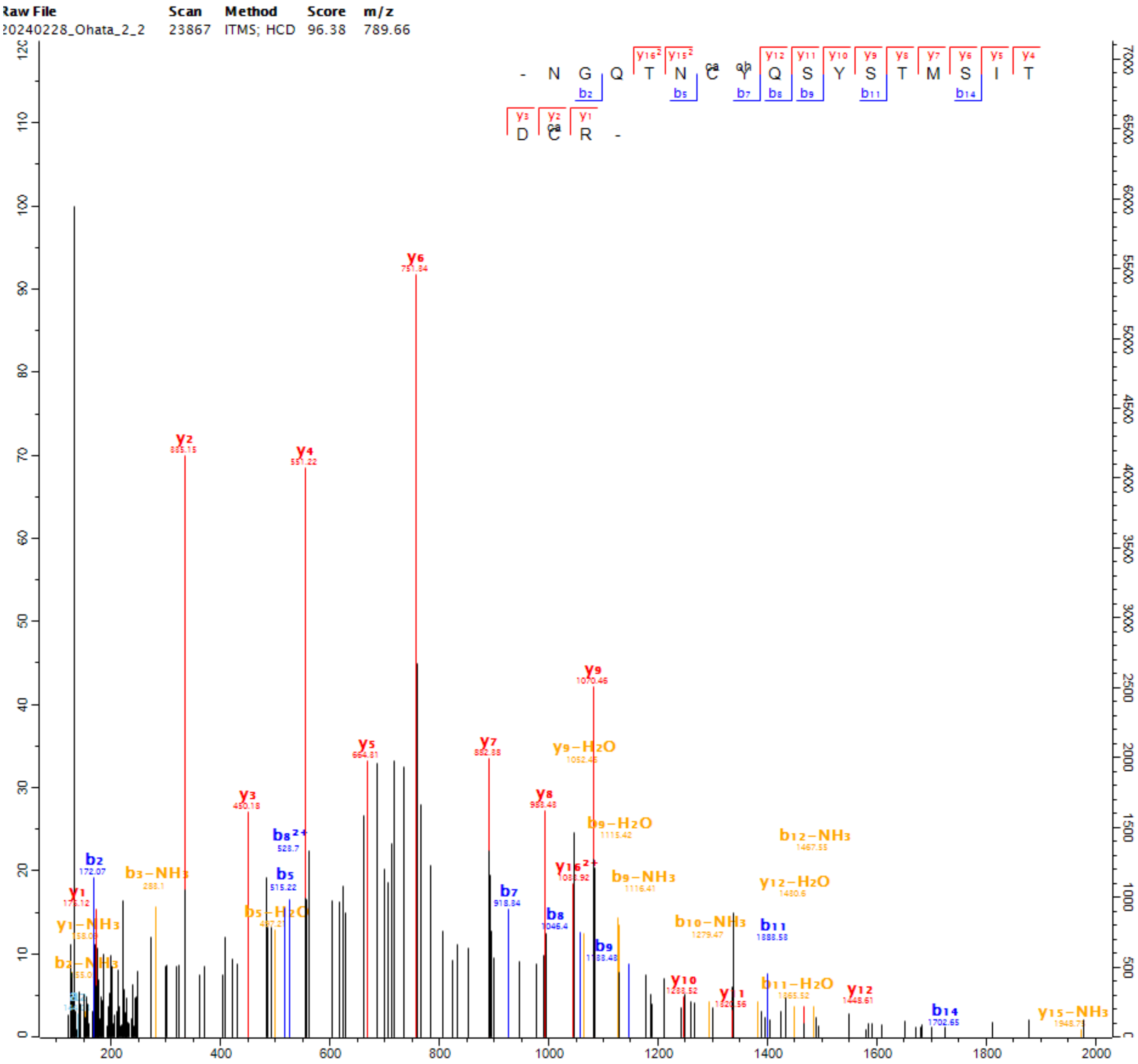

Figure S92. MS/MS analysis of the peak corresponding to labeled Tyr73 of ribonuclease A (NGQTNC^{CAM}Y*QSYSTMSITDC^{CAM}R) as a representative Tyr-labeled fragment with 4-pentynoic acid.

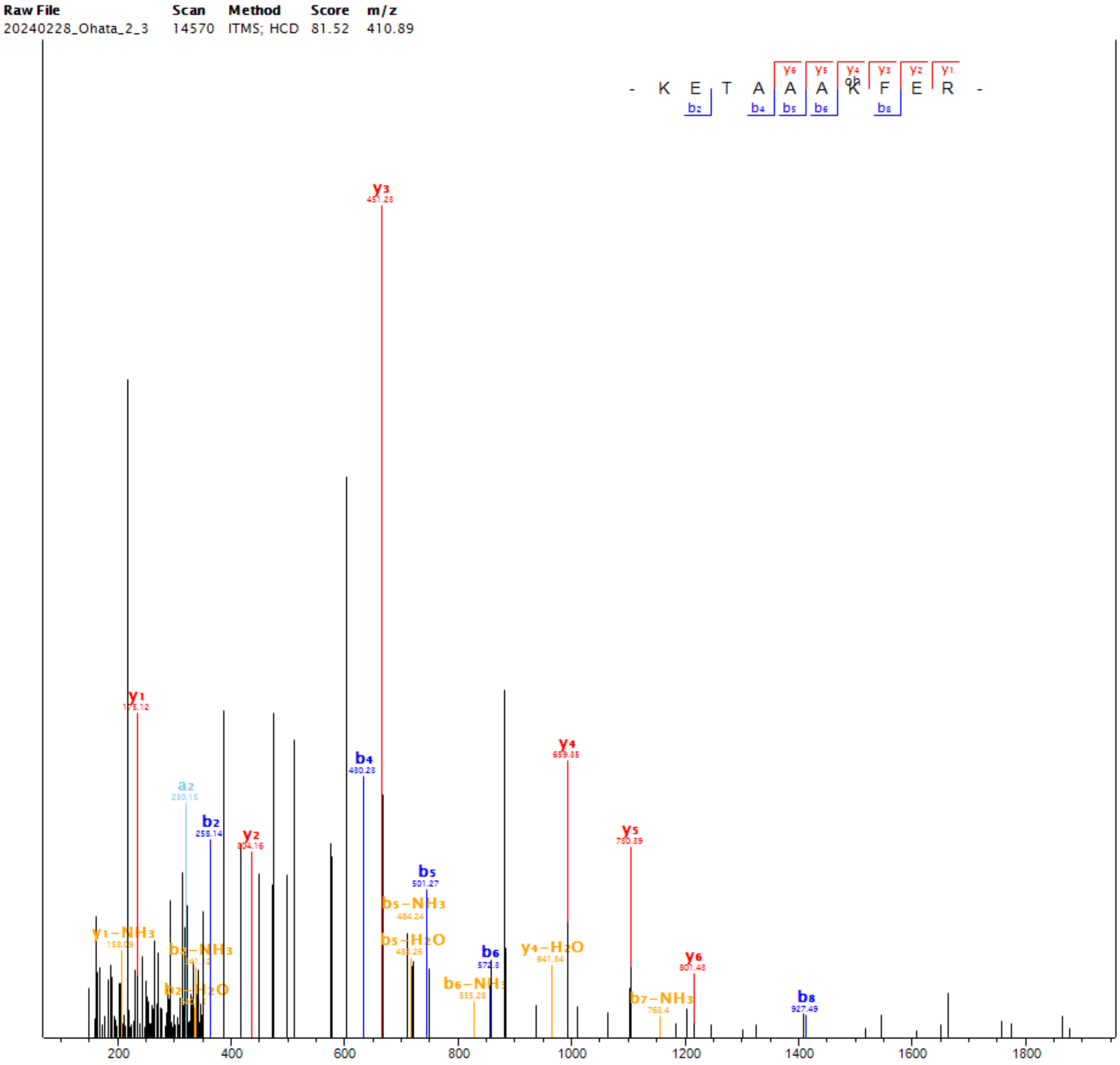

Figure S93. MS/MS analysis of the peak corresponding to labeled Lys7 of ribonuclease A (KETAAAK*FER) as a representative Lys-labeled fragment with 4-pentynoic acid.

Fluorescence imaging of HEK293T cells with WGA-fluorophore conjugate (comparison with reported data)[3]

To validate the quality of the commercial WGA-fluorophore conjugate for cell staining experiments , we first replicated a reported procedure using the commercial conjugate (WGA CF®633).[3] The reported protocol involved coating cell culture flask with poly-D-lysine/laminin solution before seeding the HEK293T cells. The procedure also showed live staining of HEK293T cells prior to fixation with 4% paraformaldehyde.

Experiments were performed in our hand using a laminin/poly-D-lysine mixture (Sigma Aldrich #127-2.5) or just poly-D-lysine solution for culture flask coating and cell fixation before and after WGA staining While similar fluorescence patterns were observed compared to the published report, we observed a significant loss of cells using the reported procedure with the laminin coating as evidenced in Figure S95 (top and middle images), and therefore, the other experiments for this manuscript used only poly-D-lysine coating (without laminin).

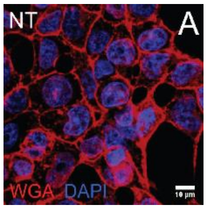

Figure S94. A confocal microscope image of HEK293T cells stained with WGA-Rhodamine in published literature, which is used as a comparison/standard of our control experiment.[3] The cell image was reproduced from Garita-Hernandez et al., licensed by CC BY 4.0.

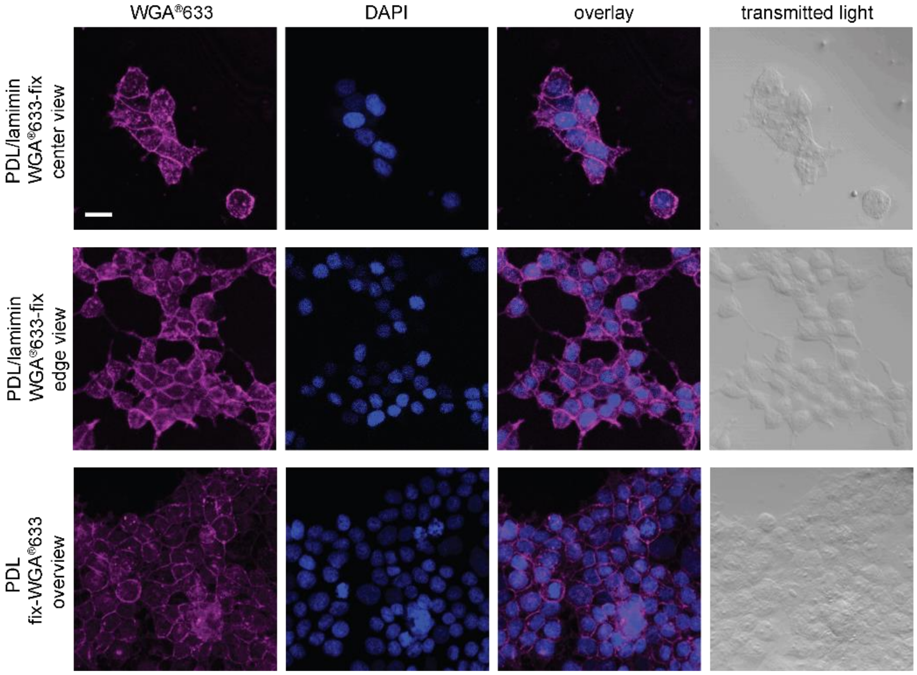

Figure S95. Confocal microscopy images of HEK293T cells stained with commercial wheat germ agglutinin (WGA) CF®633. Conditions: cell culture flask was coated with poly-D-lysine/Laminin solution, WGA CF®633 was introduced before cell fixation (top and middle), cell culture flask was coated with poly-D-lysine solution, WGA CF®633 was introduced after cell fixation (bottom). Blue: nuclear stain with DAPI. Scale bar: 20 µm. WGA CF®633 concentration (2.5 µg/mL final concn from 1 mg/mL stock solution in water), DAPI (0.1 µg/mL final concn from 0.1 mg/mL stock solution in water).

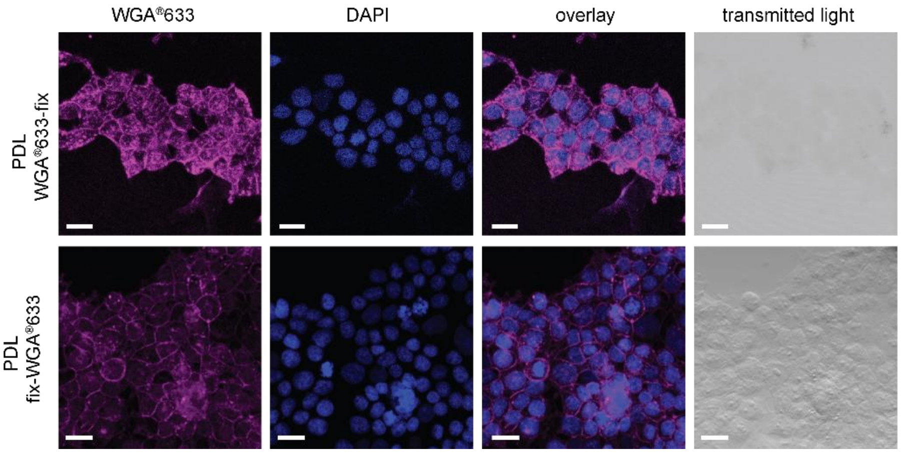

Figure S96. Confocal microscopy images of HEK293T cells stained with commercial wheat germ agglutinin (WGA) CF®633. Conditions: cell culture flask was coated with poly-D-lysine solution, WGA CF®633 was introduced before (top) or after (bottom) cell fixation. Blue: nuclear stain with DAPI. Scale bar: 20 µm. WGA CF®633 concentration (2.5 µg/mL final concn from 1 mg/mL stock solution in water), DAPI (0.1 µg/mL final concn from 0.1 mg/mL stock solution in water).

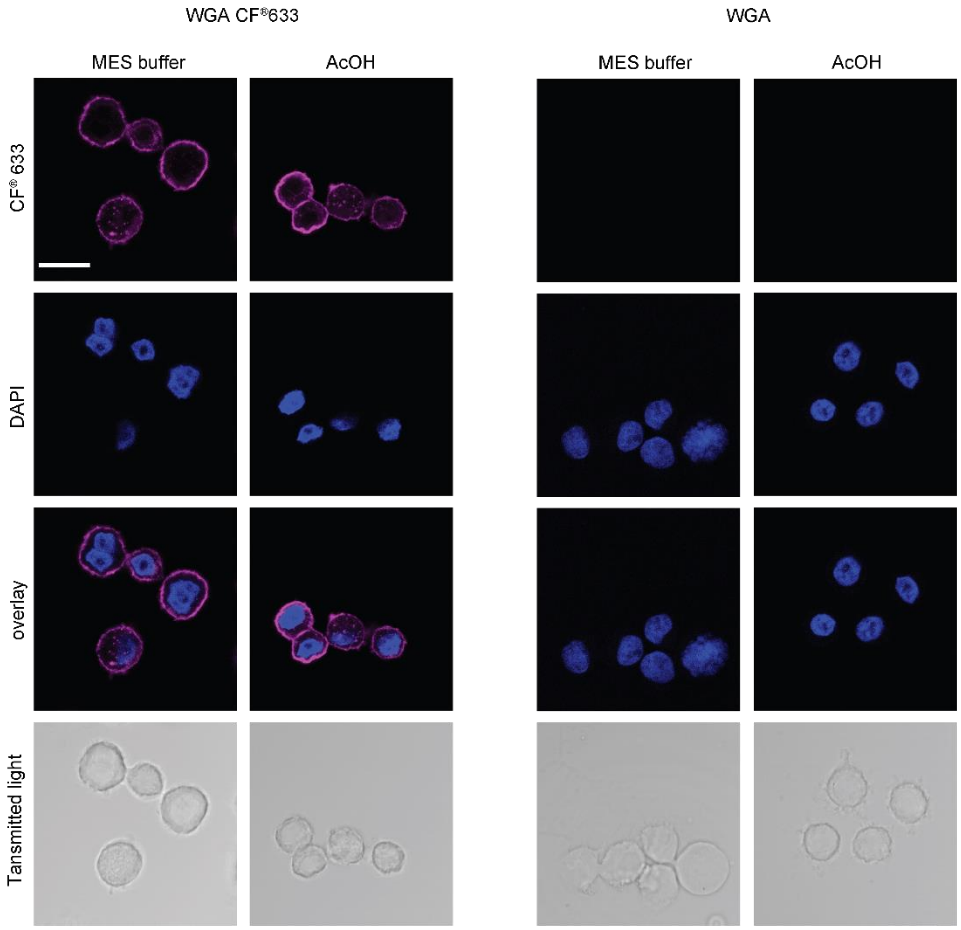

Figure S97. Confocal microscopy images of SK-BR-3 cells stained with commercial wheat germ agglutinin (WGA) CF®633 (left) or WGA (right) treated in either 50 mM MES buffer (pH 7.4) or neat acetic acid. Blue: nuclear stain with DAPI. Scale bar: 20 µm. WGA CF®633/WGA concentration (5 µg/mL final concn from 1 mg/mL stock solution in water), DAPI (0.1 µg/mL final concn from 0.1 mg/mL stock solution in water).

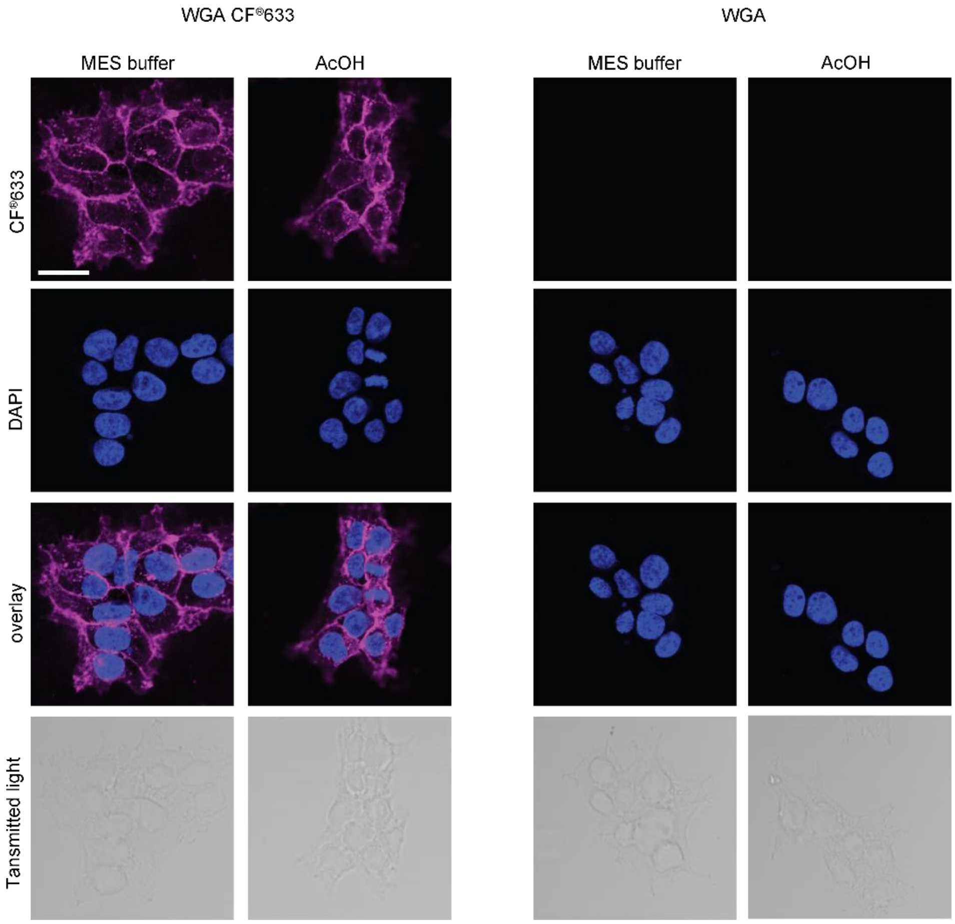

Figure S98. Confocal microscopy images of HEK293T cells stained with commercial wheat germ agglutinin (WGA) CF®633 (left) or WGA (right) treated in either 50 mM MES buffer or neat acetic acid. Blue: nuclear stain with DAPI. Scale bar: 20 µm. WGA CF®633/WGA concentration (5 µg/mL final concn from 1 mg/mL stock solution in water), DAPI (0.1 µg/mL final concn from 0.1 mg/mL stock solution in water).

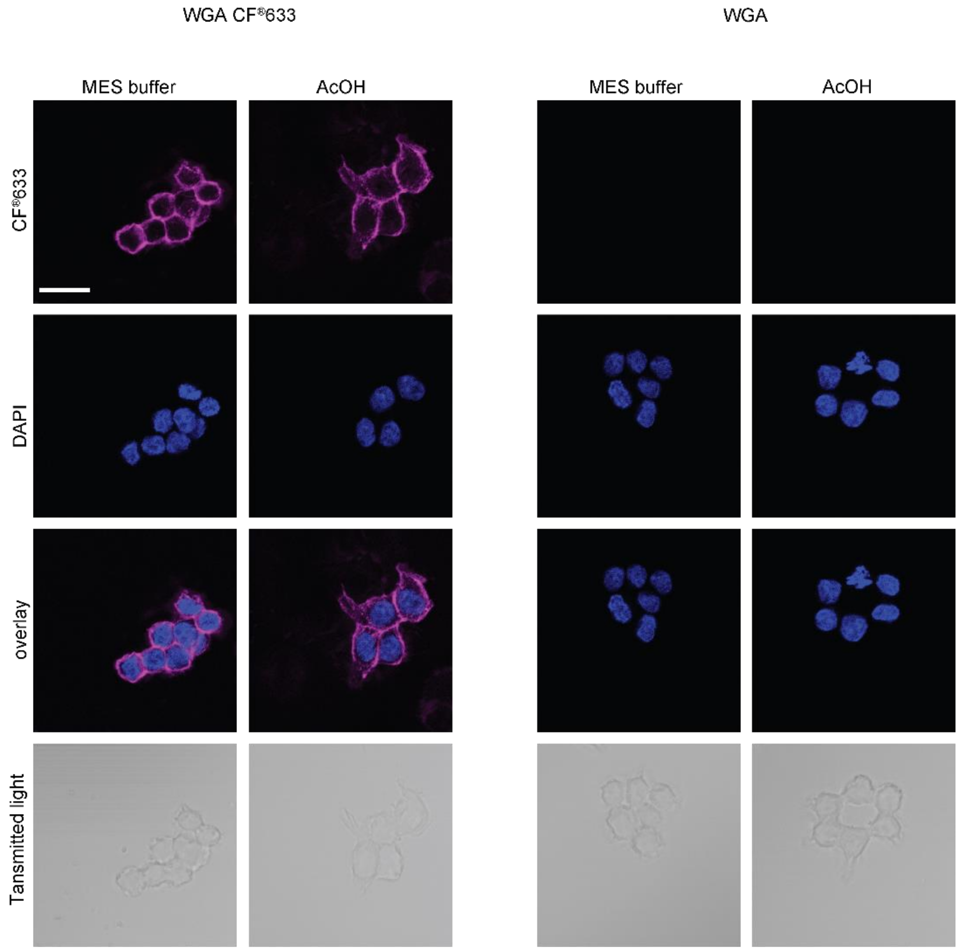

Figure S99. Confocal microscopy images of HCT-116 cells stained with commercial wheat germ agglutinin (WGA) CF®633 (left) or WGA (right) treated in either 50 mM MES buffer or neat acetic acid. Blue: nuclear stain with DAPI. Scale bar: 20 µm. WGA CF®633/WGA concentration (5 µg/mL final concn from 1 mg/mL stock solution in water), DAPI (0.1 µg/mL final concn from 0.1 mg/mL stock solution in water).

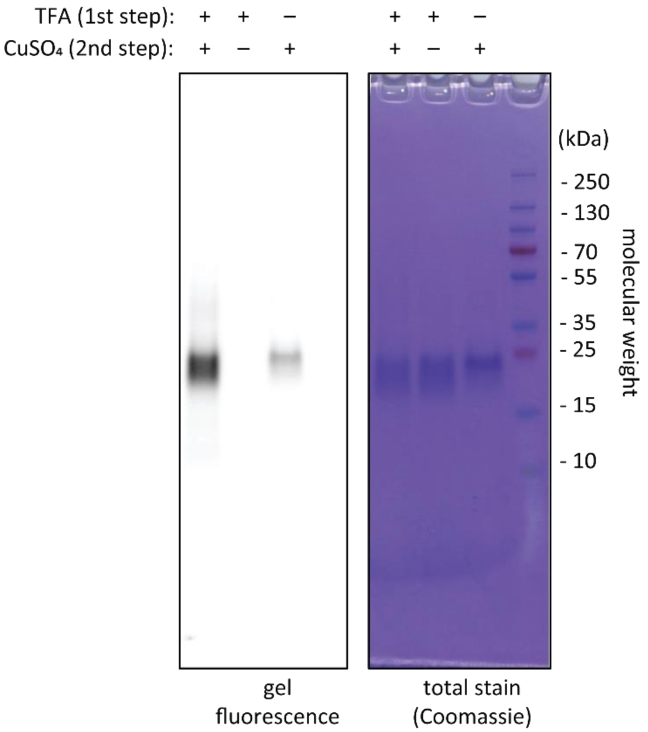

Figure S100. Fluorescence gel analysis of WGA modified with 4-pentynoic acid (acid-catalyzed acylation) followed by BODIPY-N_3 (copper-catalyzed azide-alkyne cycloaddition). ESI-MS analysis was not performed, as the commercial WGA (Simga-Aldrich L 9640) was a mixture of different isoforms. Fluorescence imaging was performed by 460 nm excitation with a 525 nm bandpass filter (±20 nm). Molecular weight marker: Thermo Scientific 26619. The *WGA modification, purification, and analysis* section describes the procedures for the preparations of WGA-BODIPY conjugate.

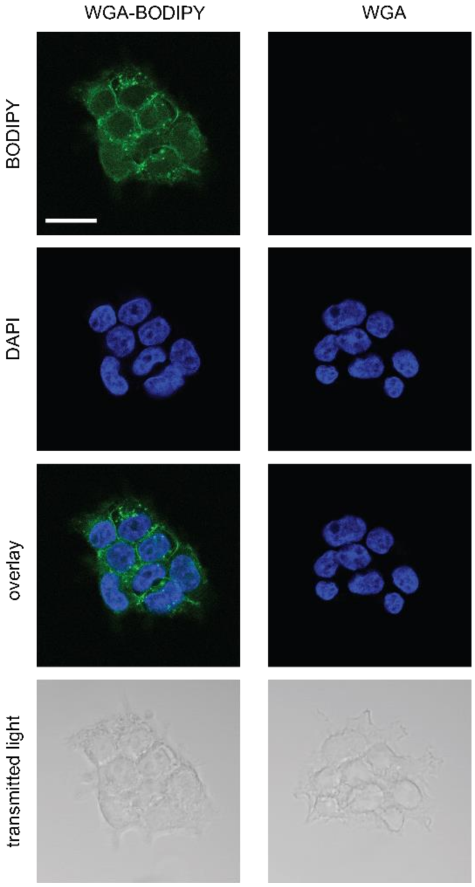

Figure S101. Confocal microscopy images of HEK293T cells stained with wheat germ agglutinin (WGA)-BODIPY (left) or WGA (right, no Cu catalyst control in the conjugation process). Blue: nuclear stain with DAPI. Scale bar: 20 µm. WGA-BODIPY/WGA (5 µg/mL final concn from 1 mg/mL stock solution in water), DAPI (0.1 µg/mL final concn from 0.1 mg/mL stock solution in water). The *WGA modification, purification, and analysis* section describes the procedures for the preparations of WGA-BODIPY conjugate.

Reactivity of fluorophosphonate toward a non-enzyme active site serine

To confirm the selectivity of the synthesized fluorophosphonate toward non-enzyme active site serine, *N*-protected serine (Fmoc-Ser-OH) was treated with fluorophosphonate **2**. As we expected, a modification was not observed in the liquid-chromatography mass spectrometry (LC-MS) analysis shown below.

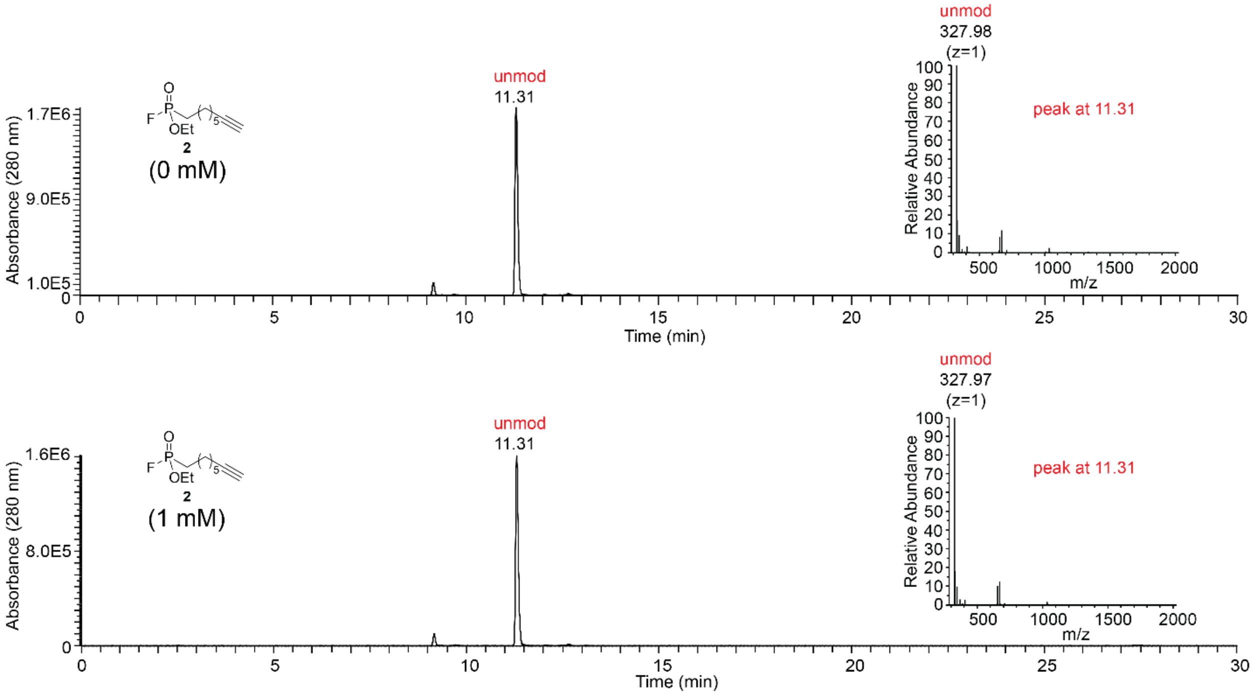

Figure S102. Liquid-chromatography mass spectrometry (LC-MS) analysis of reaction mixtures of Fmoc-Ser-OH treated with fluorophosphonate-alkyne **2**. Reaction conditions: Fmoc-Ser-OH (50 µM final concentration from 5 mM stock solution in 1,4-dioxane), fluorophosphonate-alkyne **2** (1 mM final concentration from 100 mM stock solution in DMSO), and *N*-methyl morpholine buffer (5 mM final concn from 50 mM pH 7.4 stock solution in H_2O) were incubated in H_2O at 50 °C for 24 h. After the reaction, the reaction mixture was centrifuged (15 krcf, rt, 15 min), and the resulting supernatant was analyzed by LC-MS with hypersil gold column (Thermo Fisher, 25005-259070A, particle size 5 µm, diameter 10 mm, length 250 mm).

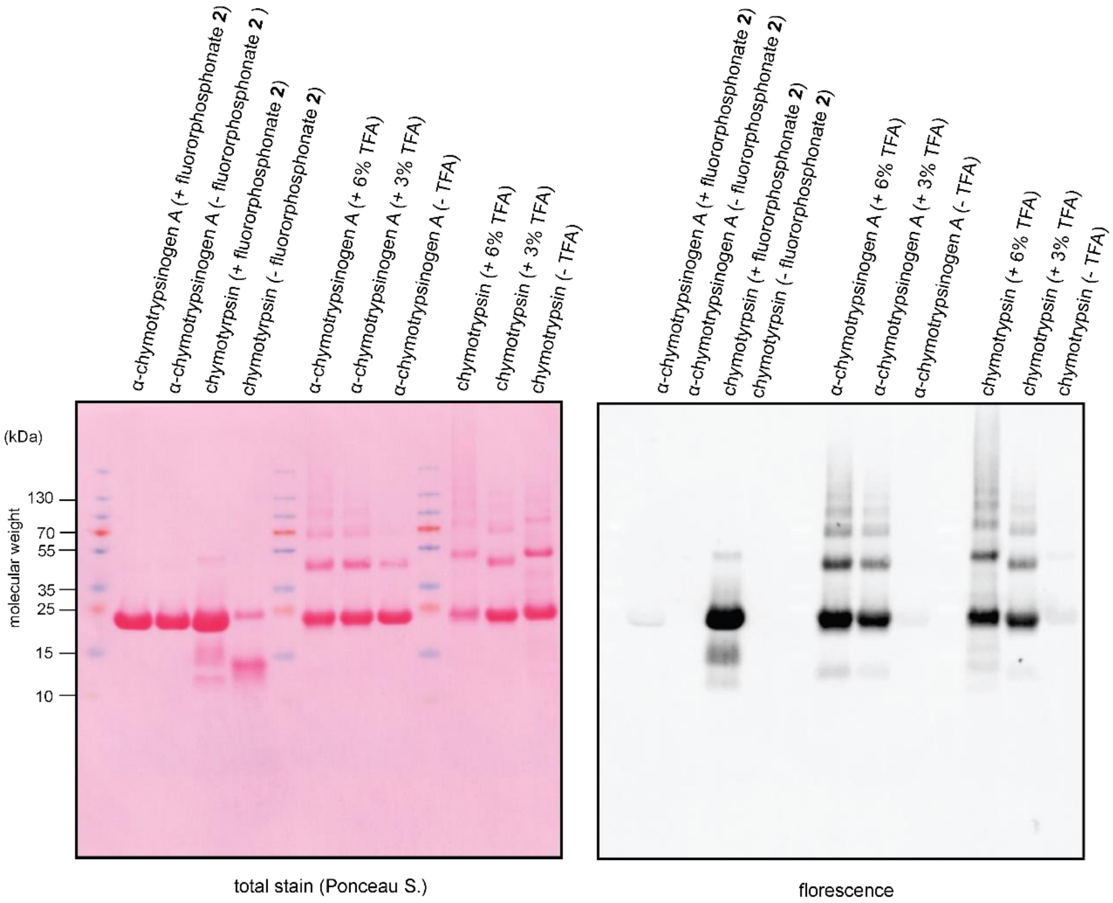

Figure S103. Chemical blot analysis (detection of an alkyne tag in proteins on a blot membrane with fluorogenic coumarin azide) of α-chymotrypsinogen A and chymotrypsin modified with either 4-pentynoic acid (acid-catalyzed acylation) or alkyne-tagged fluorophosphonate **2** (active-site specific labeling). Reaction conditions of acid-catalyzed acylation: Proteins (see below for the concentration) and TFA (0–6% v/v, 0–0.78 M final concn from neat liquid) were incubated in 4-pentynoic acid (4 M in EMIM BF_4). Reaction conditions of active-site specific labeling: proteins (see below for the concentration), alkyne-tagged fluorophosphonate **2** (360 µM final concn from 36 mM stock solution in DMSO), and *N*-methyl morpholine (NMM) buffer (5 mM final concn from 50 mM stock solution in H_2O, pH 7.4) were incubated in H_2O at rt for 1 h. Concentrations of proteins: α-Chymotrypsinogen A (240 µM final concn from freshly prepared 8 mM (by mass) stock solution in cold 50 mM MES buffer, pH 7.4), chymotrypsin (240 µM final concn from freshly prepared 8 mM (by mass) stock solution in 1 mM aq. HCl). Fluorescence imaging was performed by 460 nm excitation with 525 nm bandpass filter (±20 nm). Molecular weight marker: Thermo Scientific 26619.

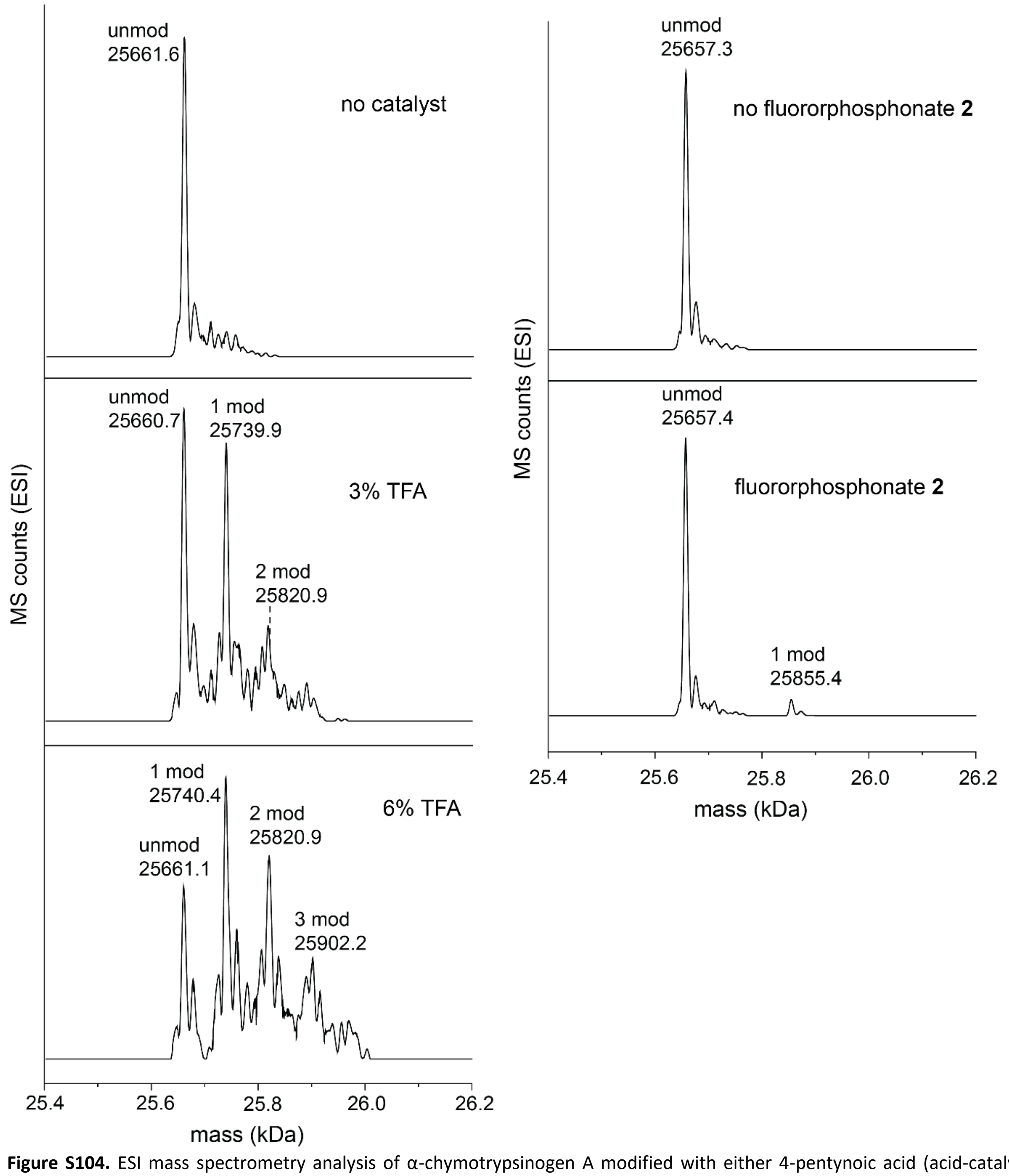

Figure S104. ESI mass spectrometry analysis of α-chymotrypsinogen A modified with either 4-pentynoic acid (acid-catalyzed acylation) or alkyne-tagged fluorophosphonate **2** (active-site specific labeling). Reaction conditions of acid-catalyzed acylation: α-Chymotrypsinogen A (240 µM final concn from freshly prepared 8 mM (by mass) stock solution in cold 50 mM MES buffer, pH 7.4) and TFA (0–6% v/v, 0–0.78 M final concn from neat liquid) were incubated in 4-pentynoic acid (4 M in EMIM BF_4). Reaction conditions of active-site specific labeling: α-Chymotrypsinogen A (240 µM final concn from freshly prepared 8 mM (by mass) stock solution in cold 50 mM MES buffer, pH 7.4), alkyne-tagged fluorophosphonate **2** (360 µM final concn from 36 mM stock solution in DMSO), and *N*-methyl morpholine (NMM) buffer (5 mM final concn from 50 mM stock solution in H_2O, pH 7.4) were incubated in H_2O at rt for 1 h.

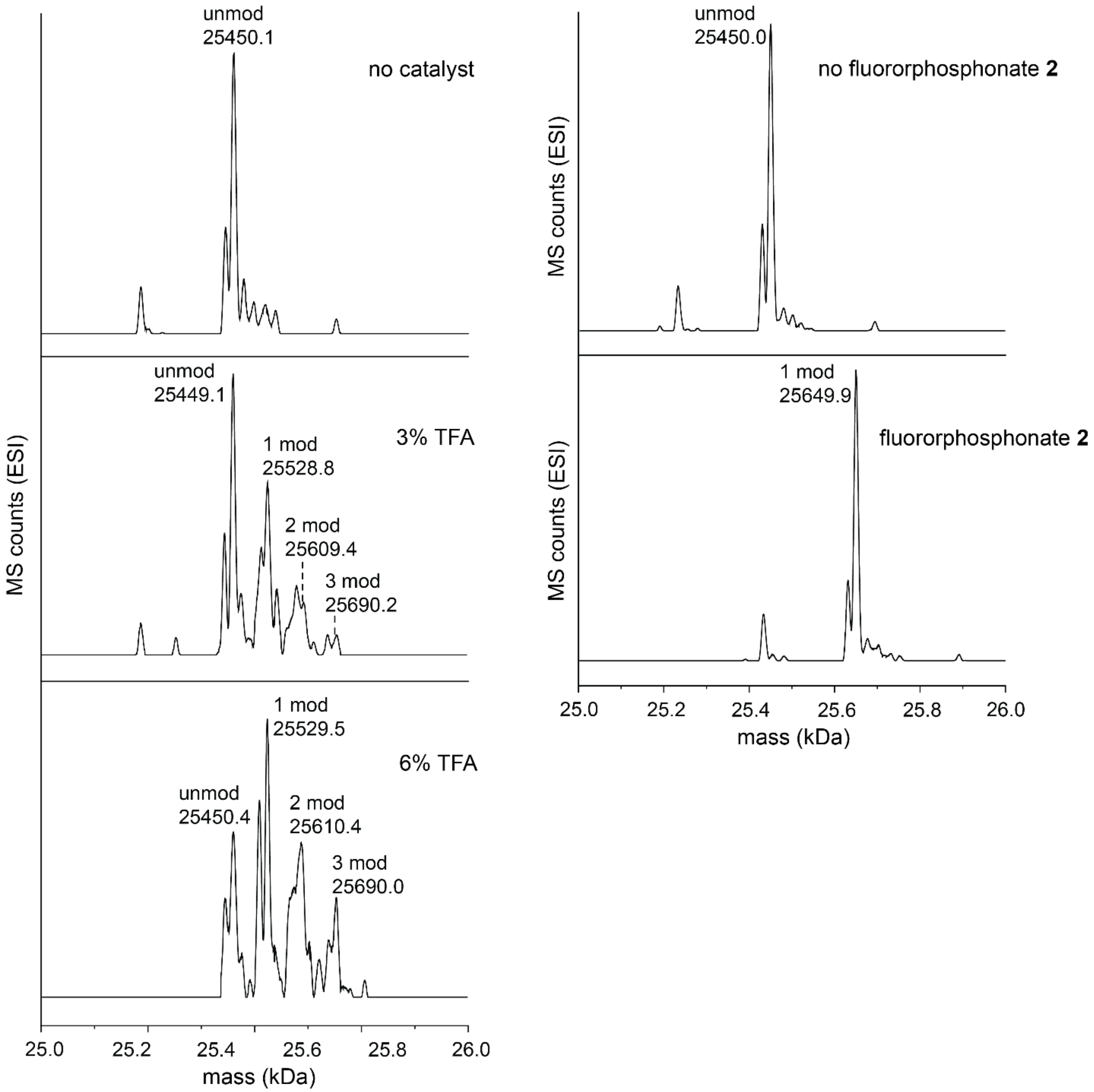

Figure S105. ESI mass spectrometry analysis of chymotrypsin modified with either 4-pentynoic acid (acid-catalyzed acylation) or alkyne-tagged fluorophosphonate **2** (active-site specific labeling). Reaction conditions of the acid-catalyzed acylation: chymotrypsin (240 µM final concn from freshly prepared 8 mM (by mass) stock solution in 1 mM aq. HCl) and TFA (0–6% v/v, 0–0.78 M final concn from neat liquid) were incubated in 4-pentynoic acid (4 M in EMIM BF_4). Reaction conditions of the active-site specific labeling: Chymotrypsin (240 µM final concn from freshly prepared 8 mM (by mass) stock solution in 1 mM aq. HCl), alkyne-tagged fluorophosphonate **2** (360 µM final concn from 36 mM stock solution in DMSO), and *N*-methyl morpholine (NMM) buffer (5 mM final concn from 50 mM stock solution in H_2O, pH 7.4) were incubated in H_2O at rt for 1 h.

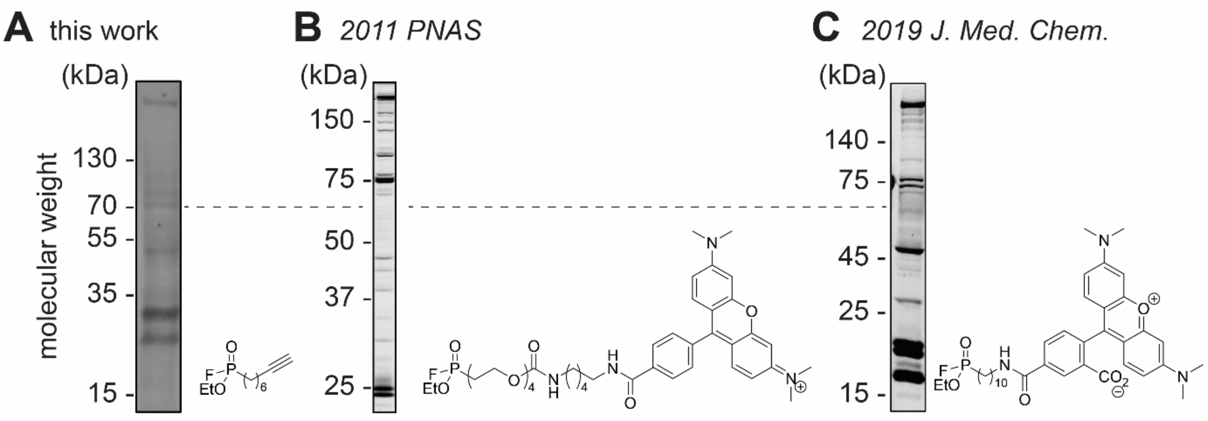

Figure S106. Comparison of the labeling of lysates of HEK293T with fluorophosphonate reagents with reported data. (A) HEK293T lysate labeled with fluorophosphonate-alkyne **2**, then analyzed by chemical blotting with fluorogenic coumarin azide (this work). A full-size membrane image is shown in Figure 5. (B) Reported image of HEK293T lysate labeled with **S3**, then analyzed by in-gel fluorescence scanning. The image of gel-imaging was reproduced with permission. [4] (C) Reported image of HEK293T lysate labeled with **S4**, then analyzed by in-gel fluorescence scanning. The image of gel-imaging was adapted with permission.[5] Copyright 2024, American Chemical Society.

General information

Materials and reagents

All chemicals including peptides and proteins were purchased from commercial suppliers unless otherwise noted. All the chemical syntheses were performed under air unless otherwise noted. Fluorophosphonate-alkyne **2** was synthesized according to a reported procedure.[6] 1X phosphate-buffered saline (PBS) pH 7.4 was purchased from Corning (21-040-CM). Protein gel/blot molecular weight marker was purchased from Thermo Scientific (26619). 4X loading buffer for gel electrophoresis was purchased from Thermo Scientific (NP0007). Ionic liquid, 1-ethyl-3-methylimidazolium tetrafluoroborate (EMIM BF_4) was purchased from Ambeed (A724626). Acetic acid was purchased from Thermo Scientific (010994). Trifluoroacetic acid (TFA) was purchased from Sigma Aldrich (TX1275). 4-pentynoic acid was purchased from Ambeed (A391230). Cbz-Ser-NH_2 was purchased from Chem-Impex (12325). 4-Pentynoic acid solution (5 M in acetonitrile and 4 M in EMIM BF_4) was prepared by sonication in a sonic bath.

Protein structures shown in the main manuscript were generated by using PyMol and Protein Data Bank (PDB). PDB ID: 1CLL (calmodulin in Figure 1), 1GJN (myoglobin in Figure 1), 1IJ8 (avidin in Figure 1), 3I40 (insulin in Figure 4), 1UBQ (ubiquitin in Figure 4), 1A5P (ribonuclease A in Figure 4), 1HEL (lysozyme in Figure 4), 7TTR (β-casein in Figure 4), 1APN (concanavalin A in Figure 4), 1EX3 (α-chymotrypsinogen A in Figure 5).

The molar concentrations of stock solutions of peptides and proteins were determined by nanodrop (Thermo Fisher) using their absorbance at 280 nm and theoretical extinction coefficient (obtained by Expasy Protparam) unless otherwise noted.

Cell culture

HEK293T cells (ATCC, CRL-3216), HCT-116 cells, and SK-BR-3 cells were cultured in Dulbecco's Modified Eagle Medium (DMEM) with Glutamax, 10% fetal bovine serum (FBS), and 1% penicillin-streptomycin. Cells were kept under 5% CO_2 at 37 °C. In a 24-well cell culture plate (Thermo Scientific 142475), sterilized coverslips (12 mm diameter, 0.09-0.12 mm thickness. Carolina Science & Math, #633009. Autoclaved through dry cycles before use) were coated with the poly-D-lysine solution for 1 h. Cells were seeded in the 24-well plates and fixed with 4% paraformaldehyde at 40% or 60% confluency, washed with 1X PBS three times, and used for the fluorescence imaging experiment.

List of materials

Table S6. A list of peptides and proteins used in the study.

Name	Sequence or Uniprot ID	MW (Da)	Supplier (cat #)
Somatostatin-14	H-AGCKNFFWKTFTSC-OH	1637.9	Bachem (4033009)
Allatostatin I	H-AP**S**GAQRLYGFGL-NH_2	1335.5	CPC Scientific (ALST-001)
IDR-1018	H-VRLIVAVRIWRR-NH_2	1535.92	CPC Scientific (IMMO-006A)
LHRH	Glp-HWSYGLRPG-NH_2	1182.3	Sigma-Aldrich (L7134)
TRAP-14	H-**S**FLLRNPNDKYEPF-OH	1740.0	CPC Scientific (PARP-022A)
GHRF 1-29	H-HADAIF**TSS**YRRILGQLYARKLLHEIMNR-NH_2	3473.1	CPC Scientific (GHRF-007)
PMAP-23	H-RIIDLLWRVRRPQKPKFVTVWVR-OH	2962.6	CPC Scientific (ATMP-012A)
PANP-004	H-R**T**RPLWVRME-OH	1343.6	CPC Scientific (PANP-004A)
BAMP-001	H-YGGFMRRVGRPE-OH	1424.6	CPC Scientific (BAMP-001A)
Osteocalcin (37-49)	H-GFQEAYRRFYGPV-OH	1589.8	CPC Scientific (OSTP-002A)
Dynorphin A	H-YGGFLRRIRPKLK-OH	1604.0	CPC Scientific (DYNP-007A)
β-Casein	P02666	24k	Sigma-Aldrich (C6905)
Lysozyme	P00698	14k	Sigma-Aldrich (L4919)
Wheat germ agglutinin (WGA) CF®633 conjugate	N/A	N/A	Biotium (29024)
Wheat germ agglutinin (WGA)	N/A	35k (dimer)	Sigma-Aldrich (L9640)
Concanavalin A	P02866	25k	Sigma-Aldrich (C2010)
Chymotrypsin	P00767	25k	Sigma-Aldrich (C4129)
α-Chymotrypsinogen A	P00766	26k	Thermo Scientific (J64741.03)
Insulin	P01308	6k	Sigma-Aldrich (91077C)
Ubiquitin	P0CG48	9k	Sigma-Aldrich (U6253)
Ribonuclease A	P61823	14k	Sigma-Aldrich (R6513)
Herceptin	N/A	150k	Genentech

Instrumentation

NMR

NMR was performed on Bruker AVANCE NEO 700.

LC-MS

LC-MS analysis was performed on Thermo Vanquish LC system and LTQ-XL linear ion trap MS system. A C18 reverse-phase column (hypersil gold column, Thermo Fisher, 25005-259070A, particle size 5 µm, diameter 10 mm, length 250 mm) was used for the analysis of small molecules and peptides by using 280 nm UV detection at 0.2 mL/min flow rate with the gradient of acetonitrile (10–90% for 15 min, and then 90% for 8 min, 10% for 7 min) containing 0.1% formic acid in 0.1% formic acid aqueous solution. A phenyl reverse-phase column (MAbPac 088648, particle size 4 µm, diameter 2.1 mm, length 50 mm) was used for analysis of proteins by using positive MS ion detection at 0.2 mL/min flow rate with the gradient of acetonitrile (10–90% for 3 min, and then 90% for 2 min, 10% for 2 min) containing 0.1% formic acid in 0.1% formic acid aqueous solution.
The conversion of the LC-MS-based experiments shown in the manuscript was calculated by dividing the product peak area by the sum of the product peak area and the starting material peak area.

Tandem mass spectrometry

Tandem mass spectrometry (MS/MS) for peptide substances was performed on Thermo Vanquish LC system and LTQ-XL linear ion trap MS system with the same setup described in LC-MS.

Proteomics analysis

To acetic acid (for lysozyme, 30 µL scale) or a 4 M solution of 4-pentynoic acid in EMIM BF_4 (for α-chymotrypsinogen A and ribonuclease A, 30 µL scale) in a 1.7 mL Eppendorf tube, protein and TFA (see below for the concentration) were added. Concentrations of proteins and TFA: Lysozyme (0.1 mM final concn from 10 mM stock solution in 50 mM MES buffer pH 7.4) and TFA (4% v/v, 0.52 M final concentration from neat liquid); α-chymotrypsinogen A (0.02 mM final concn from 2 mM stock solution in 50 mM MES buffer pH 7.4) and TFA (6% v/v, 0.78 M final concn from neat liquid); ribonuclease A (0.02 mM final concn from 2 mM stock solution) and TFA (12% v/v, 1.6 M final concn from neat liquid). The resulting solution was incubated at 37 °C overnight. After the reaction, to the reaction mixture, cold acetone (600 µL, –20 °C) was added in one portion. The mixture was mixed by upside-down shaking and sat at –80 °C for 1 h or overnight. The precipitates were collected by centrifugation (15,000 rcf, 15 min, 4 °C), and the supernatant was removed. The pellet was further washed by one additional cycle of acetone (600 µL) addition and centrifugation. After removing the supernatant, the pellet was air-dried on the bench at rt for 15 min. The dried pellet was reconstituted in 8.5 µL of water. 7.5 µL of the protein solution in H_2O (lysozyme: ~0.32 mM, α-chymotrypsinogen A, and ribonuclease A: ~0.062 mM) was mixed with 4X loading buffer (2.5 µL). Three identical replicates of the protein solutions were prepared and subjected to gel electrophoresis using Tris-Glycine gel (Invitrogen XP04205BOX, 4–20%) at 140 V for 45 min.
After Coomassie Blue Brilliant R-250 staining and destaining processes, the gel was cut into pieces using a razor (approximately 1 mm^2 pieces), and the three identical replicates were combined in a single 1.7 mL Eppendorf tube. The gel pieces were washed with water (3 × 1 mL, 10 min incubation at 37 °C for each wash process), followed by a wash with 1:1 100 mM ammonium bicarbonate (ABC) buffer/ MeCN and

100% MeCN (1 mL each, 10 min incubation at 37 °C for each wash process). After the 100% MeCN was removed, 100 mM dithiothreitol (DTT) in 100 mM ABC buffer was added for cysteine reduction, and the mixture was incubated at 37 °C for 30 min. The DTT solution was removed, and alkylation was performed with a solution containing 250 mM iodoacetamide in 100 mM ABC buffer at 37 °C for 30 min in the dark (i.e. with an aluminum foil cover). After the removal of the alkylation solution, the gel pieces were washed with 100 mM ABC buffer, 1:1 100 mM ammonium bicarbonate (ABC) buffer/MeCN, and 100% MeCN (1 mL each, 10 min incubation at 37 °C for each wash process). To the dehydrated gel pieces after the MeCN wash, trypsin solution (50 µL, 20 µg/ml) in 50 mM ABC buffer was added, and the mixture was incubated at 37 °C overnight. The mixture was quenched with the addition of trifluoroacetic acid solution (5 µL from 5% v/v stock solution) and lyophilized.

The resulting peptide fragments were dissolved in 5% MeCN/0.1% trifluoro acetic acid aq. And desalted using C18 pipette tips (Nikkyo Technos Co., Ltd., Tokyo, Japan). The desalted solution was applied for LC-MS analysis. NanoLC-MS/MS analysis was performed using an LC-nano-ESI-MS comprising a quadrupole, orbitrap, and ion trap Tribrid mass spectrometer (Orbitrap Fusion; Thermo Fisher Scientific) equipped with a nanospray ion source and a nano HPLC system (Easy-nLC 1000; Thermo Fisher Scientific). The trap column used for the nano HPLC was a 2 cm × 75 µm capillary column packed with 3 µm C18-silica particles (Nikkyo Technos Co., Ltd.). The micropump (flow rate: 300 nL/min) gradient method was used as follows: mobile phase A: 0.1% formic acid; mobile phase B: 80% acetonitrile, 0.1% formic acid aq. 0–1 min: 0–5% B, 1–61 min: 5–40% B, 61–63 min: 40–95% B, 63–80 min: 95% B. The LC-MS/MS data were acquired in a data-dependent acquisition mode controlled by xcalibur 4.3 (Thermo Fisher Scientific). The settings for the data-dependent acquisition were as follows: maximum injection time, 0.05 s; full MS (MS1, orbitrap) scan range, 375–1500 *m/z*, excluding the former target ion for 20 s; and mass tolerance, 10 ppm. The top speed cycle time was set to 3 s and the maximum MS2 scan could be acquired within that time. The MS2 (isolation: quadrupole, detection: ion trap) scan maximum injection time and range were 0.035 s and the *m/z* range defined based on the MS1 scan, respectively. The measurement was performed three times for each sample. MS/MS spectra were searched against the respective amino acid sequence using MaxQuant (Freeware) with default settings. A FASTA file corresponds to lysozyme, α-chymotrypsinogen A, and ribonuclease A was used. For labeling settings, oxidation (+O, +15.9949) for Met residues, carbamidomethylation (+C_2H_3NO, +57.0215) for Cys residues, acetylation (+C_2H_2O, +42.0106) for the N-terminus, acetylation (for lysozyme, +C_2H_2O, +42.0106), acylation with 4-pentynoic acid (for α-chymotrypsinogen A and ribonuclease A, +C_5H_4O, +80.0262), and acylation with trifluoroacetic acid (for all proteins, +$C_2HF_3O_2$–H_2O, +95.9823) for Ser, Thr, Tyr, and Lys residues were set as possible modifications.

Gel and blot imaging

Gel and blot imaging was performed on Amersham ImageQuant 800 (Cytiva) using 360 nm, 535 nm, and 635 nm light sources with corresponding emission bandpass filters at 525 nm (±20 nm), 605 nm (±40 nm), and 705 nm (±40 nm), respectively.

Confocal microscope

Fluorescence microscope imaging was performed on a Zeiss LSM880 confocal microscope. General imaging was performed with a 40x water immersion C-Apochromat objective lens (NA 1.1) while the imaging in an 8 well culture slide was performed with a 10x objective lens. Excitation at 405 nm (DAPI), 488 (BODIPY), and 633 (Cy5) were used with the filter settings below. Experiments with Wheat germ agglutinin (WGA) ®CF633: 415–490 nm (DAPI) and 634–758 nm (CF633). Experiments with the prepared

WGA-BODIPY conjugate: 504–631 nm (DAPI and BODIPY). Image J software was used to generate images suitable for publication.

Experimental procedures

Amino acid screening for Figure 1A

To acetic acid (30 µL scale) in a 1.7 mL Eppendorf tube, scandium (III) triflate (1.25 mM final concn from 125 mM stock solution in 1,4-dioxane) and Fmoc amino acid (5 mM final concn from 500 mM stock in acetic acid, acetonitrile, or 1,4-dioxane) were added. Specific conditions of each substrate are described in figure captions of the MS data in the Supporting Information. The resulting solution was incubated at 50 °C for 24 h. After the reaction, the reaction mixture was analyzed by following the procedure in the *analysis of amino acid samples* section.

Analysis of amino acid samples

0.3 µL of the reaction mixture was diluted with 29.7 µL of MeCN, and the diluted solution was centrifuged (15 krcf, rt, 15 min). The resulting supernatant was analyzed by LC-MS with hypersil gold column (Thermo Fisher, 25005-259070A, particle size 5 µm, diameter 10 mm, length 250 mm).

Carboxylic acid screening for Figure 1B

To different carboxylic acid (4.75 M final concn from 5 M stock solution in MeCN, 30 µL scale) in a 1.7 mL Eppendorf tube, scandium (III) triflate (20 mM final concn from 500 mM stock solution in 1,4-dioxane) and Fmoc-Ser-OH (5 mM final concn from 500 mM stock solution in 1,4-dioxane) or Fmoc-Thr-OH (5 mM final concn from 500 mM stock solution in 1,4-dioxane) were added. The resulting solution was incubated at 50 °C for 24 h. 0.3 µL of the reaction mixture was diluted with 29.7 µL of MeCN, and the diluted solution was centrifuged (15 krcf, rt, 15 min). The resulting supernatant was analyzed by LC-MS with hypersil gold column (Thermo Fisher, 25005-259070A, particle size 5 µm, diameter 10 mm, length 250 mm).

General procedures for modifications of peptides

To acetic acid (30 µL scale) in a 1.7 mL Eppendorf tube, catalyst (100 mM final concn from 200 mM stock solution in acetic acid or neat liquid, or 6–12% v/v, 0.78–1.6 M final concn of TFA from 12–24% v/v stock solution in acetic acid) and peptide (0.05–1 mM final concn from 5–100 mM stock solution in H_2O or acetic acid) were added. Specific conditions of each catalyst and peptide are described in figure captions of the LC-MS data in the Supporting Information. The reaction mixture was incubated at 50 °C for 24 h. After the reaction, the reaction mixture was analyzed by following the procedure in the *analysis of modified somatostatin-14* section or *analysis of peptide samples* section (all other peptides).

Analysis of peptide samples

All volatiles were removed by blowing the nitrogen gas for 15–30 minutes, and the residue was reconstituted in 1:1 H_2O/MeCN (30 µL). The reconstituted solution was centrifuged (15 krcf, rt, 15 min), then the resulting supernatant was analyzed by LC-MS with hypersil gold column (Thermo Fisher, 25005-259070A, particle size 5 µm, diameter 10 mm, length 250 mm).

Analysis of modified somatostatin-14

All volatiles were removed by blowing the nitrogen gas for 15–30 minutes, and the residue was reconstituted in 1:1 H_2O/MeCN (30 µL). To the solution, aq. NH_4HCO_3 (3 mM, 160 µL) was added. 2.4 µL of the resulting solution was diluted with 27.6 µL of aq. NH_4HCO_3 (3 mM), and the diluted solution was centrifuged (15 krcf, rt, 15 min). The resulting supernatant was analyzed by LC-MS with hypersil gold column (Thermo Fisher, 25005-259070A, particle size 5 µm, diameter 10 mm, length 250 mm).

Preparation of modified somatostatin-14 and insulin for tandem mass spectrometry (MS/MS)

To acetic acid (30 µL scale) in a 1.7 mL Eppendorf tube, catalyst (3–6% v/v, 0.39–0.78 M final concn of TFA from 6–12% v/v stock solution in acetic acid) and peptide (0.1–1 mM final concn from 10–100 mM stock solution in acetic acid) were added. Specific conditions of each peptide are described in figure captions of the LC-MS data in the Supporting Information. The reaction mixture was incubated at 50 °C for 24 h. After the reaction, all volatiles were removed by blowing the nitrogen gas for 15–30 minutes, and the residue was reconstituted in 1:1 H_2O/MeCN (30 µL). To the solution, aq. NH_4HCO_3 (3 mM, 160 µL) was added. 2.4 µL of the resulting solution was diluted with 27.6 µL of TCEP (0.1 mg/mL in 3 mM aq. NH_4HCO_3), and the solution was centrifuged (15 krcf, rt, 15 min). The resulting supernatant was analyzed by LC-MS with hypersil gold column (Thermo Fisher, 25005-259070A, particle size 5 µm, diameter 10 mm, length 250 mm).

Catalyst screening with α-chymotrypsinogen A

To acetic acid (30 µL scale) in a 1.7 mL Eppendorf tube, catalyst (10 mM final concn from 20 mM stock solution in acetic acid, or 1% v/v, 0.13 M final concn from neat TFA) and α-chymotrypsinogen A (0.072 mM final concn from 7.2 mM stock solution in 50 mM MES buffer, pH 7.4) were added. Specific conditions of each catalyst are described in figure captions of the *ESI mass spectrometry analysis of α-chymotrypsinogen A modified in acetic acid with various catalysts* figure. The reaction mixture was incubated at 37 °C for 24 h. 0.3 µL of the reaction mixture was diluted with 29.7 µL of H_2O. The diluted solution was centrifuged (15 krcf, rt, 15 min), and the resulting supernatant was analyzed by LC-MS (MAbPac 088648, particle size 4 µm, diameter 2.1 mm, length 50 mm). In addition to catalysts shown in the *Summary of catalyst screening for protein modification* table, 10 mM final concn of H_2SO_4, $Bi(OTf)_3$, $Cp_2Zr(OTf)_2$, $Dy(OTf)_3$, $In(OTf)_3$, $Sc(OTf)_3$, 1-methyl-3-(4-sulfobutyl)imidazolium hydrogn sulfate, and 1-butyl-3-methylimidazolium hydrogen sulfate were also screened as catalysts but no significant peak was observed in UV and mass chromatograms for the LC-MS analysis.

Preparation of acid-treated lysozyme (sample prep for the enzyme activity assay)

To acetic acid (30 µL scale) or MES buffer (50 mM, pH 7.4, 30 µL scale) in a 1.7 mL Eppendorf tube, lysozyme solution (100 µM final concn from 10 mM stock solution in 50 mM MES buffer pH 7.4) was added. The resulting solution was incubated at 37 °C for 24 h. To the mixture in a 1.7 mL Eppendorf tube, cold acetone (1200 µL, –20 °C) was added in one portion. The mixture was mixed by upside-down shaking and sat at –80 °C for 1 h or overnight. The precipitates were collected by centrifugation (15,000 rcf, 15 min, 4 °C), and the supernatant was removed. The pellet was further washed by one additional cycle of acetone

(600 µL) addition and centrifugation. After removing the supernatant, the pellet was air-dried on the bench at rt for 15 min. The dried pellet was reconstituted in 30 µL of 1X PBS (samples incubated with acetic acid) or water (samples incubated with MES buffer). The concentration of lysozyme in the solution was determined by nanodrop (Thermo Fisher) and diluted to 0.40 µM with 1X PBS. The resulting solution was used as a stock solution for the activity assay below. For the negative control experiment of denatured lysozyme, the lysozyme solution preincubated in MES buffer (0.40 µM final concn from 6.1 µM stock solution) was heated at 95 °C for 1 min in 1X PBS buffer with SDS (1% v/v final concn from 10% v/v aqueous stock solution), then cooled to room temperature.

Lysozyme activity assay

The lysozyme stock solution (50 nM final concn from 0.40 µM stock solution in 1X PBS) obtained from the *Preparation of acid-treated lysozyme* section was mixed with 1X reaction buffer (Thermo Fisher, EnzChek lysozyme assay kit, E-22013) and the DQ™ lysozyme substrate, fluorescein conjugate (50 µL, 25 µg/mL final concn from 50 µg/ml stock solution in 1X reaction buffer, Thermo Fisher, EnzChek lysozyme assay kit, E-22013) in a 96-well plate. The resulting mixture was incubated at 37 °C for 30 min. Fluorescence was measured using a fluorescence microplate reader (Biotek Synergy H1, Agilent). The fluorescence was measured using excitation/emission at 485/524 nm. The experiment was repeated three times on different days. The average and standard deviation of fluorescence intensity are shown in Figure 4C. In each experiment on different days, each condition was duplicated.

Modification of concanavalin A, ribonuclease A, and Herceptin with 4-pentynoic acid

To 4-pentynoic acid (4 M in EMIM BF_4, 10 µL scale) in a 1.7 mL Eppendorf tube, trifluoro acetic acid (0–12% v/v, 0–1.6 M final concn from neat liquid) and concanavalin A (192 µM final concn from 6.4 mM stock solution in 50 mM MES buffer pH 7.4), ribonuclease A (60 µM final concn from 2 mM stock solution in 50 mM MES buffer pH 7.4) or Herceptin (46 µM final concn from 766 µM stock solution in 50 mM MES buffer pH 7.4) were added. The specific conditions of each catalyst (TFA) are described in the figure captions of the MS data in the Supporting Information section. The reaction mixture was incubated at 37 °C overnight to 24 h. To the reaction mixture in a 1.7 mL Eppendorf tube, cold acetone (600 µL, –20 °C) was added in one portion, mixed upside-down and placed at –80 °C for 1 h or overnight. The precipitates were collected by centrifugation (15,000 rcf, 15 min, 4 °C), and the supernatant was removed. The pellet was further washed by one additional cycle of acetone (600 µL) addition and centrifugation. After removing the supernatant, the pellet was air-dried on the bench at rt for 15 min.

<u>LC-MS analysis</u>: The dried pellet was reconstituted in water (30 µL), and a further 10X dilution of the ribonuclease A solution was prepared while concanavalin A was not diluted further for LC-MS The diluted solution was centrifuged (15 krcf, rt, 15 min), and the resulting supernatant was analyzed by LC-MS (MAbPac 088648, particle size 4 µm, diameter 2.1 mm, length 50 mm) analysis.

<u>Chemical blotting analysis</u>: To the dried pellet, 1X loading buffer (40 µL, loading buffer prepared from 4X loading buffer, Thermo BN2003, and water) was added and heated at 95 °C in a sand bath. A 6X dilution of the concanavalin A sample or a 3X dilution of the Herceptin samples were prepared. The diluted samples (10 µL each) were subjected to SDS-PAGE at 140 V for 45 min. The modified proteins on the gel were transferred to a PVDF membrane through a semi-dry transfer process. The membrane was subjected to chemical blotting (alkyne detection) according to the *chemical blotting for the detection of alkyne handles* procedure.

Modification of α-chymotrypsinogen A, and chymotrypsin with 4-pentynoic acid

To 4-pentynoic acid (4 M in EMIM BF_4, 10 µL scale) in a 1.7 mL Eppendorf tube, trifluoro acetic acid (0–6% v/v, 0–0.78 M final concn from neat liquid) and α-chymotrypsinogen A (240 µM final concn from freshly prepared 8 mM (by mass) stock solution in cold 50 mM MES buffer, pH 7.4) or chymotrypsin (240 µM final concn from freshly prepared 8 mM (by mass) stock solution in cold 1 mM HCl) was added. The specific conditions of each catalyst (TFA) are described in the figure captions of the MS data in the Supporting Information section. The reaction mixture was incubated at 37 °C for 24 h. To the reaction mixture in a 1.7 mL Eppendorf tube, cold acetone (600 µL, –20 °C) was added in one portion, mixed upside-down and placed at –80 °C for 1 h or overnight. The precipitates were collected by centrifugation (15,000 rcf, 15 min, 4 °C), and the supernatant was removed. The pellet was further washed by one additional cycle of acetone (600 µL) addition and centrifugation. After removing the supernatant, the pellet was air-dried on the bench at rt for 15 min.

LC-MS analysis: The dried pellet was reconstituted in water (30 µL). A 4X dilution in water was prepared for LC-MS. The diluted solution was centrifuged (15,000 rcf, rt, 15 min), and the resulting supernatant was analyzed by LC-MS (MAbPac 088648, particle size 4 µm, diameter 2.1 mm, length 50 mm) analysis.

Chemical blotting analysis: To the dried pellet, 1X loading buffer (40 µL, loading buffer prepared from 4X loading buffer, Thermo BN2003 and water) was added. A 2X dilution in 1X loading buffer of the sample was prepared and heated at 95 °C in a sand bath. The heated samples (10 µL each) were subjected to SDS-PAGE at 140 V for 45 min. The modified proteins on the gel were transferred to a PVDF membrane through a semi-dry transfer process. The membrane was subjected to chemical blotting (alkyne detection) according to the *chemical blotting for the detection of alkyne handles* procedure.

Modification of α-chymotrypsinogen A, and chymotrypsin with alkyne-tagged fluorophosphonate 2

To water (30 µL scale) in a 1.7 mL Eppendorf tube, *N*-methyl morpholine (NMM) buffer (5 mM final concn from 50 mM stock solution in H_2O, pH 7.4),alkyne-tagged fluorophosphonate **2** (360 µM final concn from 36 mM stock solution in DMSO), and α-chymotrypsinogen A (240 µM final concn from freshly prepared 8 mM by mass stock solution in cold 50 mM MES buffer, pH 7.4) or chymotrypsin (240 µM final concn from freshly prepared 8 mM by mass stock solution in cold 1mM aq. HCl) was added. The reaction mixture was incubated at room temperature for 1 h.

LC-MS analysis: A 15X dilution in water of the reaction mixture was prepared for LC-MS. The diluted solution was centrifuged (15,000 rcf, rt, 15 min), and the resulting supernatant was analyzed by LC-MS (MAbPac 088648, particle size 4 µm, diameter 2.1 mm, length 50 mm) analysis.

Chemical blotting analysis: To the reaction mixture in a 1.7 mL Eppendorf tube, cold acetone (1200 µL, –20 °C) was added in one portion, mixed upside-down and placed at –80 °C for 1 h or overnight. The precipitates were collected by centrifugation (15,000 rcf, 15 min, 4 °C), and the supernatant was removed. The pellet was further washed by one additional cycle of acetone (600 µL) addition and centrifugation. After removing the supernatant, the pellet was air-dried on the bench at rt for 15 min. To the dried pellet, 1X loading buffer (40 µL, loading buffer prepared from 4X loading buffer, Thermo BN2003 and water) was added. A 6X dilution in 1X loading buffer of the sample was prepared and heated at 95 °C in a sand bath. The diluted samples (10 µL each) were subjected to SDS-PAGE at 140 V for 45 min. The modified proteins

on the gel were transferred to a PVDF membrane through a semi-dry transfer process. The membrane was subjected to chemical blotting (alkyne detection) according to the *chemical blotting for detection of alkyne handles* procedures.

Treatment of commercial wheat germ agglutinin (WGA) CF®633 with different media

To MES buffer (50 mM in water, pH 7.4), or neat acetic acid in a 1.7 mL Eppendorf tube, commercial wheat germ agglutinin (WGA) CF®633 (60 µg/mL final concn from 1 mg/mL stock solution in water) was added and incubated for 24 h at 37 °C. To the reaction mixture in a 1.7 mL Eppendorf tube, cold acetone (1200 µL, –20 °C) was added in one portion, mixed upside-down and placed at –80 °C for 1 h or overnight. The precipitates were collected by centrifugation (15,000 rcf, 15 min, 4 °C), and the supernatant was removed. The resulting pellet was air-dried on the bench at rt for 15 min. The dried pellet was reconstituted in water (10 µL) and used in the *fluorescence imaging experiment*.

General fluorescence imaging experiment

HEK293T cells (ATCC, CRL-3216), HCT-116 cells, and SK-BR-3 cells were cultured and fixed in a 24-well culture plate with coverslips, according to the procedure described in the *general cell culture procedure*. Cells were incubated with treated commercial wheat germ agglutinin (WGA) CF®633 (5 µg/mL final concn from 180 µg/mL stock solution in water) or prepared WGA-BODIPY (5 µg/mL final concn from 1 mg/mL stock solution in water) in 1X PBS (300 µL scale) at room temperature for 30 min. After the incubation, cells were washed with 1X PBS solution three times. The coverslips with stained cells were mounted onto a microscope slide with Prolong Gold Antifade Mountant (Thermo, P10144) and CoverGrip Coverslip Sealant (Biotium, 23005), and the cells were imaged using a confocal microscope. Nuclei were counterstained with 4',6-diamidino-2-phenylindole dihydrochloride (DAPI, 0.1 µg/mL final concn from 0.1 mg/mL stock solution in water).

Cell culture procedure for HEK293T stained with WGA CF®633 before fixing with PFA in culture slide coated with poly-D-lysine/laminin solution.

HEK293T cells were cultured and stained according to a modified protocol.[3] HEK293T cells (ATCC, CRL-3216) were cultured in Dulbecco's Modified Eagle Medium (DMEM) with Glutamax, 10% fetal bovine serum (FBS), and 1% penicillin-streptomycin. Cells were kept under 5% CO_2 at 37 °C. An 8 well cell culture slide (Lab-Tek II CC^2 chamber slide 154941) was coated with the poly-D-lysine/laminin solution (175 µL) at room temperature for 1 h. The solution was aspirated, and the flask was washed twice in 1X PBS. The culture slide was air-dried for 2 h. On day 1, 10,000 cells per well were seeded in the 8 well culture slide in DMEM with 10% FBS without antibiotics. After two days, the medium was replaced by DMEM with 10% FBS without antibiotics. On day 4, the cells were rinsed three times with 1X PBS and stained with commercial wheat germ agglutinin (WGA) CF®633 (2.5 µg/mL final concn from 1 mg/mL stock solution in water) in 1X PBS (300 µL scale) for 10 min before being fixed with 4% paraformaldehyde for 10 min at room temperature. Nuclei were counterstained with 4',6-diamidino-2-phenylindole dihydrochloride (DAPI, 0.1 µg/mL final concn from 0.1 mg/mL stock solution in water).

Cell culture procedure for HEK293T stained with WGA CF®633 after fixing with PFA in culture slide coated with poly-D-lysine solution.

HEK293T cells (ATCC, CRL-3216) were cultured in Dulbecco's Modified Eagle Medium (DMEM) with Glutamax, 10% fetal bovine serum (FBS), and 1% penicillin-streptomycin. Cells were kept under 5% CO_2 at 37 °C. An 8 well cell culture slide (Lab-Tek II CC^2 chamber slide 154941) was coated with the poly-D-lysine solution (175 µL) at room temperature for 1 h. The solution was aspirated, and the flask was washed three times with distilled water. The culture slide was air-dried for 2 h. On day 1, 10,000 cells per well were seeded in the 8 well culture slide in DMEM with 10% FBS and 1% penicillin-streptomycin (300 µL). After two days, the medium was replaced by DMEM with 10% FBS and 1% penicillin-streptomycin. On day 4, the cells were rinsed two times with HBBS and fixed with 4% paraformaldehyde for 10 min at room temperature before being stained with commercial wheat germ agglutinin (WGA) CF®633 (2.5 µg/mL final concn from 1 mg/mL stock solution in water) in 1X PBS (300 µL scale) for 10 min. Nuclei were counterstained with 4',6-diamidino-2-phenylindole dihydrochloride (DAPI, 0.1 µg/mL final concn from 0.1 mg/mL stock solution in water).

Fluorescence imaging experiment for HEK293T cells stained with WGA CF®633 before/after fixing in 8 well cell culture slide.

HEK293T cells (ATCC, CRL-3216) stained before or after fixing with 4% PFA in an 8 well culture slide (Lab-Tek II CC^2 chamber slide 154941) were washed three times (3 min, each time) in 1X PBS and kept in PBS for confocal microscope imaging.

Confocal microscope

Fluorescence microscope imaging was performed on Zeiss LSM800 confocal microscope. General imaging was performed with a 40x water immersion C-Apochromat objective lens (NA 1.1) while the imaging in an 8 well culture slide was performed with a 10x objective lens. Excitation at 405 nm (DAPI), 488 (BODIPY), and 633 (Cy5) were used with the filter settings below. Experiments with Wheat germ agglutinin (WGA) ®CF633: 415–490 nm (DAPI) and 634–758 nm (CF633). Experiments with the prepared WGA-BODIPY conjugate: 504–631 nm (DAPI and BODIPY). Image J software was used to generate images suitable for publication.

Cell lysate preparation

HEK 293T cells were allowed to reach 80–90% confluency in the T-75 flask (surface area: 75 cm^2) or T-25 flask (surface area: 25 cm^2). Old media was discarded, and cells were washed with PBS buffer. Cells were detached from the flask via trypsinization for five minutes at 37 °C (5% CO_2). After the incubation, supplemented DMEM (10% FBS, 1% penicillin/streptomycin) was added at two times the volume of trypsin and immediately centrifuged (3 min, 4 °C). The supernatant was discarded, and the pellet was resuspended in ice-cold PBS and centrifuged under the same conditions noted previously. This process was repeated two additional times. After the final wash-centrifuge-aspirate cycle, cells were resuspended and counted. Again, cells were centrifuged, supernatant was aspirated, and the pellet was placed in a –80 °C freezer for 30 minutes. 100 µL of 1X NP-40 lysis buffer (prepared from 2X buffer, Alfa Aesar #J62805 and 1X PBS) was added per every 1,000,000 cells and homogenized, placing the sample on ice every 30 seconds to ensure that the sample was not exposed to excessive heating. The crude mixture was

incubated for 30 minutes on ice and subsequently centrifuged (15,000 rcf, 15 min, 4 °C) to pellet cell debris and insoluble material. The supernatant was collected, and approximate concentrations were determined by comparing previously prepared lysate with known concentrations through Ponceau staining or by Bradford Assay.

Chemical blotting for the detection of alkyne handles

The blot membrane after the transfer process was soaked with methanol for 5 min and rinsed with water twice. Chemical blotting with fluorogenic azide-coumarin was performed following a reported procedure.[7] Conditions for the chemical blotting are as follows: To 19.7 mL of 1:1 H_2O/DMSO mixture, 300 µL of freshly prepared sodium ascorbate (1.5 mM final concn from 100 mM stock in water), 20 µL of THTPA (0.1 mM final concn from 100 mM stock in water), 15 µL of coumarin-azide (0.015 mM final concn from 20 mM stock in DMSO), and 15 µL of $CuSO_4$ (0.075 mM final concn from 100 mM stock in water) were added. The membrane in the solution was shaken at rt for 0.5–18 h. The reaction solution was discarded, and the membrane was rinsed with methanol and washed 1:1 DMSO/methanol mixture for 5 min. The rinsing and washing process was repeated three times in total. The membrane was rinsed with methanol and then with 70% aqueous ethanol, and the dried membrane was imaged with Amersham ImageQuant 800. Afterward, the membrane was reactivated with methanol and water before Ponceau S stain. at rt for 2 min.

Preparative synthesis of small molecules

Organic synthesis procedures

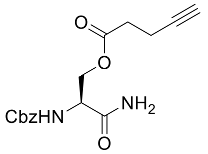

Synthesis of Cbz-Ser-NH_2 alkyne. Cbz-Ser-NH_2 (10 mg, 0.042 mmol) was added to a 4-mL vial equipped with a magnetic stir bar. TFA (12 µL, 0.157 mmol) and 4-pentynoic acid solution in MeCN (88 µL, 5 M, 0.440 mmol) were added. The mixture was heated for 24 h at 75 °C. After the incubation, the mixture was cooled to rt and sat. aq. $NaHCO_3$ (150 µL) was added. The product was extracted with ethyl acetate three times, and the resulting organic layer was washed with sat. aq. $NaHCO_3$ and water. The product was purified by TLC (silica gel 60, Sigma-Aldrich #1057150001) with ethyl acetate as a developing solvent. The product was recovered from the silica by the addition of ethyl acetate. The suspension was passed through filter paper to remove silica. Removal of the volatiles under vacuum afforded the Cbz-Ser-NH_2 alkyne as white solids (5.4 mg, 0.017 mmol, 40%). ^{1}H NMR (700 MHz, acetone-*d*6): δ 7.42-7.29 (m, 5H), 7.17 (s, 1H), 6.61 (m, 2H), 5.14-5.04 (m, 2H), 4.50-4.45 (m, 1H), 4.45-4.40 (m, 1H), 4.35-4.30 (m, 1H), 2.54-2.49 (m, 2H), 2.46-2.42 (m, 2H), 2.35 (t, *J* = 2.6 Hz, 1H). ^{13}C NMR (176 MHz, acetone-*d*6) δ 171.9, 171.5, 156.9, 138.0, 129.2,128.7, 128.6, 83.4, 70.4, 67.0, 64.9, 54.7, 33.8, 14.6. FT-IR: 3347, 3295, 1722, 1677. HRMS-ESI (m/z) $[M + H]^+$ calcd for $C_{16}H_{19}N_2O_5$, 319.1288; found 319.1289.

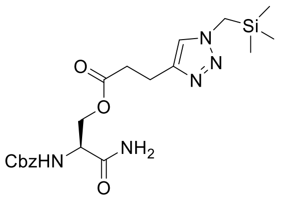

Synthesis of Cbz-Ser-NH_2 TMS ester. Cbz-Ser-NH_2 alkyne (3.5 mg, 0.011 mmol), sodium ascorbate (15.2 mg, 0.077 mmol), and THPTA (9.6 mg, 0.022 mmol) were added to a 4-mL vial equipped with a magnetic stir bar. MeCN (165 µL), water (45 µL), trimethylsilylmethyl azide solution (22 µL, 1 M in MeCN, 22 µmol) and $CuSO_4{\cdot}5H_2O$ (8.24 mg, 33 µmol) were added. The mixture was stirred at rt for 3.5 h, and additional trimethylsilylmethyl azide solution (44 µL, 1 M in MeCN, 44 µmol) was added. The mixture was stirred at rt overnight. After the incubation, the volatiles were removed by nitrogen gas blowing. The crude product was extracted from the remaining aqueous mixture with ethyl acetate three times. The crude product was purified by TLC (silica gel 60, Sigma-Aldrich #1057150001) with ethyl acetate as a developing solvent. The product was recovered from the silica by the addition of ethyl acetate. The suspension was passed through filter paper to remove silica. Removal of the volatiles under vacuum afforded the Cbz-Ser-NH_2 TMS ester as white solids (4.4 mg, 9.8 µmol, 89%). ^{1}H NMR (700 MHz, acetone-*d*6): δ 7.39-7.29 (m, 5H), 7.20 (s, 1H), 6.83 (s, 1H), 6.49 (s, 1H), 5.47 (s, 1H), 5.14 (s, 2H), 4.48 (m, 2H), 4.36 (m, 1H), 3.83(s, 2H), 3.06-2.96 (m, 2H), 2.77-2.65 (m, 2H), 0.11 (s, 9H). ^{13}C NMR (176 MHz, CDCl3) δ 172.4, 171.8, 156.6, 146.1, 136.6, 128.8, 128.5, 128.3, 122.3, 67.5, 64.2, 54.2, 42.3, 34.2, 21.2, -2.2. IR: 3332, 2952, 1722, 1684, 1252. HRMS-ESI (m/z) $[M + H]^+$ calcd for $C_{20}H_{30}N_5O_5Si$, 448.2011; found 448.2011.

References for Electronic Supplementary Information

(1) Killian, J. A.; Salemink, I.; de Planque, M. R. R.; Lindblom, G.; Koeppe, R. E.; Greathouse, D. V. Induction of Nonbilayer Structures in Diacylphosphatidylcholine Model Membranes by Transmembrane α-Helical Peptides: Importance of Hydrophobic Mismatch and Proposed Role of Tryptophans. *Biochemistry* **1996**, *35* (3), 1037–1045. https://doi.org/10.1021/bi9519258.

(2) Xiao, Y.; Zhou, H.; Shi, P.; Zhao, X.; Liu, H.; Li, X. Clickable Tryptophan Modification for Late-Stage Diversification of Native Peptides. *Sci. Adv.* **2024**, *10* (28), eadp9958. https://doi.org/10.1126/sciadv.adp9958.

(3) Garita-Hernandez, M.; Guibbal, L.; Toualbi, L.; Routet, F.; Chaffiol, A.; Winckler, C.; Harinquet, M.; Robert, C.; Fouquet, S.; Bellow, S.; Sahel, J.-A.; Goureau, O.; Duebel, J.; Dalkara, D. Optogenetic Light Sensors in Human Retinal Organoids. *Front. Neurosci.* **2018**, *12*. https://doi.org/10.3389/fnins.2018.00789.

(4) Bachovchin, D. A.; Mohr, J. T.; Speers, A. E.; Wang, C.; Berlin, J. M.; Spicer, T. P.; Fernandez-Vega, V.; Chase, P.; Hodder, P. S.; Schürer, S. C.; Nomura, D. K.; Rosen, H.; Fu, G. C.; Cravatt, B. F. Academic Cross-Fertilization by Public Screening Yields a Remarkable Class of Protein Phosphatase Methylesterase-1 Inhibitors. *Proc. Natl. Acad. Sci.* **2011**, *108* (17), 6811–6816. https://doi.org/10.1073/pnas.1015248108.

(5) Wang, Y.-L.; Liu, S.; Yu, Z.-J.; Lei, Y.; Huang, M.-Y.; Yan, Y.-H.; Ma, Q.; Zheng, Y.; Deng, H.; Sun, Y.; Wu, C.; Yu, Y.; Chen, Q.; Wang, Z.; Wu, Y.; Li, G.-B. Structure-Based Development of (1-(3′-Mercaptopropanamido)Methyl)Boronic Acid Derived Broad-Spectrum, Dual-Action Inhibitors of Metallo- and Serine-β-Lactamases. *J. Med. Chem.* **2019**, *62* (15), 7160–7184. https://doi.org/10.1021/acs.jmedchem.9b00735.

(6) Yang, J.; Korovesis, D.; Ji, S.; Kahler, J. P.; Vanhoutte, R.; Verhelst, S. H. L. Efficient Synthesis of an Alkyne Fluorophosphonate Activity-Based Probe and Applications in Dual Colour Serine Hydrolase Labelling. *Isr. J. Chem.* **2023**, *63* (3–4), e202200094. https://doi.org/10.1002/ijch.202200094.

(7) Ohata, J.; Vohidov, F.; Ball, Z. T. Convenient Analysis of Protein Modification by Chemical Blotting with Fluorogenic “Click” Reagents. *Mol. Biosyst.* **2015**, *11* (11), 2846–2849. https://doi.org/10.1039/c5mb00510h.
